# Assessing variation in faecal glucocorticoid concentrations in gray whales exposed to anthropogenic stressors

**DOI:** 10.1093/conphys/coad082

**Published:** 2023-11-11

**Authors:** Enrico Pirotta, Alejandro Fernandez Ajó, K C Bierlich, Clara N Bird, C Loren Buck, Samara M Haver, Joseph H Haxel, Lisa Hildebrand, Kathleen E Hunt, Leila S Lemos, Leslie New, Leigh G Torres

**Affiliations:** Centre for Research into Ecological and Environmental Modelling, University of St Andrews, The Observatory, Buchanan Gardens, St Andrews, Fife, Scotland KY16 9LZ, UK; Geospatial Ecology of Marine Megafauna Lab, Marine Mammal Institute, Department of Fisheries, Wildlife and Conservation Sciences, Oregon State University, 2030 SE Marine Science Drive, Newport, OR 97365, USA; Geospatial Ecology of Marine Megafauna Lab, Marine Mammal Institute, Department of Fisheries, Wildlife and Conservation Sciences, Oregon State University, 2030 SE Marine Science Drive, Newport, OR 97365, USA; Geospatial Ecology of Marine Megafauna Lab, Marine Mammal Institute, Department of Fisheries, Wildlife and Conservation Sciences, Oregon State University, 2030 SE Marine Science Drive, Newport, OR 97365, USA; Department of Biological Sciences, Northern Arizona University, 617 S. Beaver St., Flagstaff, AZ 86011, USA; Cooperative Institute for Marine Ecosystem and Resources Studies, Oregon State University, 2030 SE Marine Science Drive, Newport, OR 97365, USA; Department of Fisheries, Wildlife, and Conservation Sciences, Oregon State University, 104 Nash Hall, Corvallis, OR 97331, USA; Pacific Northwest National Laboratory, Coastal Sciences Division, 1529 W. Sequim Bay Rd., Sequim, WA 98362, USA; Geospatial Ecology of Marine Megafauna Lab, Marine Mammal Institute, Department of Fisheries, Wildlife and Conservation Sciences, Oregon State University, 2030 SE Marine Science Drive, Newport, OR 97365, USA; Smithsonian-Mason School of Conservation & Department of Biology, George Mason University, 1500 Remount Rd, Front Royal, VA 22630, USA; Institute of Environment, Florida International University, 3000 NE 151st St, North Miami, FL 33181, USA; Department of Mathematics, Computer Science and Statistics, Ursinus College, 601 E Main St, Collegeville, PA 19426, USA; Geospatial Ecology of Marine Megafauna Lab, Marine Mammal Institute, Department of Fisheries, Wildlife and Conservation Sciences, Oregon State University, 2030 SE Marine Science Drive, Newport, OR 97365, USA

**Keywords:** anthropogenic stressors, baleen whales, dose–response functions, faecal hormone metabolites, glucocorticoids, Pacific Coast Feeding Group gray whales, physiological variation

## Abstract

Understanding how individual animals respond to stressors behaviourally and physiologically is a critical step towards quantifying long-term population consequences and informing management efforts. Glucocorticoid (GC) metabolite accumulation in various matrices provides an integrated measure of adrenal activation in baleen whales and could thus be used to investigate physiological changes following exposure to stressors. In this study, we measured GC concentrations in faecal samples of Pacific Coast Feeding Group (PCFG) gray whales (*Eschrichtius robustus*) collected over seven consecutive years to assess the association between GC content and metrics of exposure to sound levels and vessel traffic at different temporal scales, while controlling for contextual variables such as sex, reproductive status, age, body condition, year, time of year and location. We develop a Bayesian Generalized Additive Modelling approach that accommodates the many complexities of these data, including non-linear variation in hormone concentrations, missing covariate values, repeated samples, sampling variability and some hormone concentrations below the limit of detection. Estimated relationships showed large variability, but emerging patterns indicate a strong context-dependency of physiological variation, depending on sex, body condition and proximity to a port. Our results highlight the need to control for baseline hormone variation related to context, which otherwise can obscure the functional relationship between faecal GCs and stressor exposure. Therefore, extensive data collection to determine sources of baseline variation in well-studied populations, such as PCFG gray whales, could shed light on cetacean stress physiology and be used to extend applicability to less-well-studied taxa. GC analyses may offer greatest utility when employed as part of a suite of markers that, in aggregate, provide a multivariate measure of physiological status, better informing estimates of individuals’ health and ultimately the consequences of anthropogenic stressors on populations.

## Teaser

Understanding animals’ responses to stressors is required to assess population consequences and inform management. We develop a flexible analytical approach to investigate the relationships between glucocorticoid concentrations in gray whale faeces and exposure to sound and vessels. Our results highlight that context (e.g., sex, body condition) modulates physiological disturbance responses.

## Introduction

Assessment of the effects of stressors resulting from human activities and environmental change is central to the effective conservation of wildlife populations (Tyack *et al.,* 2022). When animals are exposed to stressors, they often respond by changing behaviour and/or physiology. In recent decades, several conceptual frameworks have emerged to predict how these short-term individual responses can manifest to the population level in long-lived species, where direct observations of long-term effects are often unfeasible (Ankley *et al.,* 2010; Pirotta *et al.,* 2018; Wilson *et al.,* 2020). However, quantifying individual responses to stressors remains a fundamental starting point for assessing population-level consequences.

Most research in this field has focused on the behavioural-energetic pathway linking exposure to population effects via behavioural changes (Pirotta *et al.,* 2018): changes in behaviour can lead to either increased energy expenditure (e.g. due to evasive responses) or reduced energy intake (e.g. via reduced feeding time); as a result, an altered energy budget can affect the ability of an individual to allocate resources to survive, grow and reproduce. Behavioural responses have been measured in the context of observational or experimental studies, with the probability of individual behavioural change modelled as a function of the received dose of the stressor, resulting in a dose–response function (e.g. Harris *et al.,* 2018). Dose–response functions (also referred to as exposure-response or stressor-response functions, depending on the context) are a fundamental approach to address basic and applied questions in ecology (Rosenfeld *et al.,* 2022). For example, they can be used to simulate the occurrence of responses within mechanistic models to predict longer-term effects (e.g. Pirotta *et al.,* 2021).

Behavioural state is the manifestation of underpinning physiological mechanisms and emerges from a complex set of competing motivations, so that behavioural responses tend to be highly context-dependent (Gill *et al.,* 2001; Beale and Monaghan, 2004). Therefore, additional response pathways that are not necessarily reflected in behaviour can also lead to adverse effects (Bejder *et al.,* 2009). For example, across taxa individuals might tolerate a disturbance source when the benefit of remaining in a foraging patch outweighs the risks or when they are accustomed to its presence in their habitat, but aspects of their physiology (e.g. heart rate or hormone release) might still be affected. Moreover, responses to disturbance may alter an individual’s health, survival and reproduction independently of energetic status, e.g. alterations in the endocrine system, immune system and other organ systems (Pirotta *et al.,* 2022; Tyack *et al.,* 2022). Thus, understanding how exposure to stressors alters the physiology of individuals represents a critical next step towards the quantification of the effects of stressors on wildlife populations.

The hypothalamus–pituitary–adrenal (HPA) axis is one of the primary neuroendocrine pathways involved in animal responses to stressors, culminating in the secretion of glucocorticoid (GC) hormones, among a complex set of other physiological changes (Dickens and Romero, 2013; Dantzer *et al.,* 2014; MacDougall-Shackleton *et al.,* 2019). Stress-induced increases in circulating GCs promote adaptive processes, such as energy balance regulation, re-routing of blood flow and temporary inhibition of some physiological processes (e.g. digestion), that together prioritize short-term survival and facilitate coping with stressors. Activation of the HPA axis is thought to be adaptive in the short term, but sustained or intense activation can push this stress response system into a maladaptive state termed chronic stress, with detrimental consequences on individual health (Romero and Wingfield, 2015). GC accumulation in matrices such as faeces or hair can therefore provide an integrated measure of cumulative adrenal activation, and the timeline of incorporation varies across matrices (Dantzer *et al.,* 2014). However, potential sensitization, habituation or exhaustion of the system, together with the fact that GCs are involved in many other processes that relate to energy mobilization, make the interpretation of measured GC concentrations challenging (Dickens and Romero, 2013; MacDougall-Shackleton *et al.,* 2019). Interpreting GC concentrations and their variation requires extensive information on population and individual baselines to contextualize GC data within the large and normal variability resulting from the environment, life-history status, sex, or time (e.g. circadian and seasonal rhythms) (Goymann, 2012; Dickens and Romero, 2013; Madliger and Love, 2014; MacDougall-Shackleton *et al.,* 2019). Meta-analyses indicate that GC data derived from sample types that capture GC secretion over time (e.g. faecal or urinary GCs) provide a more integrated and accessible way to assess chronic stress than plasma-based measures (Dickens and Romero, 2013). However, even when using faecal GC metrics, distinguishing GC alterations that are specifically due to chronic stress from normal variation in baseline GCs can be challenging.

In baleen whales, studies of physiological stress responses were initially hampered by inability to collect blood samples. In the past two decades, advances in methodologies for assessing GC concentrations in non-plasma matrices (e.g. baleen plates, blubber, earplugs and faeces) have demonstrated that GC patterns in these sample types are associated with exposure to stressors over medium to long timescales (months to decades). For example, GC concentrations were lower in North Atlantic right whale (*Eubalaena glacialis*) faecal samples in association with a reduction in underwater sound levels following the events of 11 September 2001 (Rolland *et al.,* 2012), and in humpback whale (*Megaptera novaeangliae*) blubber samples during the 2021 foraging season associated with reduced tourism due to the COVID-19 pandemic (Pallin *et al.,* 2022). Increased GC concentrations in baleen, faeces and blubber are also associated with known cases of prolonged illness or injury, such as chronic entanglement in fishing gear (Hunt *et al.,* 2006, 2017*a*; Rolland *et al.,* 2017, 2019; Lysiak *et al.,* 2018; Lowe *et al.,* 2021) and chronic wounding (Fernández-Ajó *et al.,* 2018). On a longer timescale, Trumble *et al.* (2018) found a correlation of GC patterns in earplugs of fin (*Balaenoptera physalus*), blue (*Balaenoptera musculus*) and humpback whales with whaling counts and anomalies in sea surface temperature.

In contrast, less evidence exists for the ability of GCs to capture shorter-term responses of baleen whales exposed to stressors, an issue relevant for many management and policy decisions regarding regulation of relatively short-term disturbance events. Collecting data to fit physiological dose–response functions in large, free-ranging cetaceans is challenging because in most scenarios available technology prevents us from measuring GC concentrations before and after the experimental exposure to a stressor in a minimally invasive manner that allows replicate sampling. Moreover, while behavioural responses can be measured on short timescales, a temporal lag often exists between exposure to a stressor and our ability to collect a sample from a specific matrix where the corresponding change in GC concentration can be detected. However, measuring GC concentrations in association with varying stressor levels can function as a natural experiment to explore the relationships between exposure to a stressor and subsequent physiological changes. This assessment also has analytical ramifications: a suitable modelling approach is required to capture the potentially non-linear nature of these relationships, while controlling for the multitude of contextual variables that influence the short-term effects of stressors in wild vertebrates and accounting for laboratory detection abilities and sampling variability. An important variable that is not generally included in studies of GC concentrations in cetaceans is body condition. Recent reviews of GC patterns across vertebrate taxa indicate that the negative health and fitness effects associated with stressor exposure are likely to be strongly modulated by body condition, because animals are more vulnerable to stress when they have diminished energy reserves (e.g. Breuner and Berk, 2019).

A large body of research has documented the behavioural responses of cetaceans to sound (Nowacek *et al.,* 2007), specifically, shipping noise (Erbe *et al.,* 2019), whose effects are intertwined with and confounded by the potential disturbance from the physical presence of the vessels (e.g. Pirotta *et al.,* 2015). Conversely, relatively few studies have been able to link cetacean physiological variation to sound (e.g. Rolland *et al.,* 2012; Elmegaard *et al.,* 2021; Yang *et al.,* 2021; Williams *et al.,* 2022), and these studies were either on captive animals or not able to fully incorporate relevant contextual variables. In a preliminary analysis, Lemos *et al.* (2022*a*) identified a correlation between the levels of GC metabolite concentrations in faecal samples from gray whales (*Eschrichtius robustus*) and vessel counts in the day prior to sample collection, suggesting that such analysis is viable in observational studies.

The aim of this study is to use faecal samples collected from gray whales of the Pacific Coast Feeding Group (PCFG) to assess the relationships between faecal GC concentrations and individual exposure to varying levels of two stressors (underwater sound and vessel activity), while quantifying and controlling for the effect of several contextual variables that are known to modulate the HPA axis in mammals (sex, reproductive status, age, body condition, year and time of year). Our hypothesis is that GC relationships with the stressors under analysis may be context-dependent and vary as a function of these variables. To investigate these questions, we add data (2019–2022) to the 2016–2018 dataset of Lemos *et al.* (2022*a*), and propose an improved statistical methodology to estimate the relationships between varying stressor levels and GC concentration that accommodates the complexities of these data. Finally, we use our findings to identify future research priorities that will help refine and improve our ability to model complex multivariate physiological dose–response phenomena, which may be broadly applicable to ecological studies across marine and terrestrial taxa.

## Materials and Methods

### Ethics statement

All methods were carried out in accordance with relevant guidelines and regulations. This project was approved by the Oregon State University Institutional Animal Care and Use Committee (IACUC-2019-0008) and complies with the ARRIVE guidelines. Analysis of the data was also approved by the Animal Welfare and Ethics Committee (AWEC) of the University of St Andrews (UK). All gray whale data collection was carried out under a research permit from NOAA/NMFS (#16011 and #21678, issued to John Calambokidis). Drone operations were conducted by a Federal Aviation Authority (FAA) certified private pilot with a Part107 license or under a Certificate of Authorization (2016-WSA-101-COA).

### Overview

We collected faecal samples from free-ranging gray whales in a summer feeding ground, and paired the samples with information on the sex, age, reproductive status and body condition (from aerial photogrammetry) of the sampled individuals. We analysed the variation in faecal GC concentrations as a function of a set of metrics characterizing the soundscape (median, 95th percentile and variance of sound levels) and the levels of vessel traffic (counts of different vessel types) experienced by the whales, summarized over multiple temporal windows prior to sample collection. The models included all the ancillary information listed above, as well as the year, day of the year and the concentrations of the metabolites of other hormones, to control for their effect on GC concentrations. Moreover, we tested for specific interactions between the effect of the stressor metrics (sound levels and vessel counts) and some contextual variables (sex, body condition and sample location) that could alter an individual’s physiological response. The modelling approach accommodated potential non-linearity in the relationships, censored data and repeated sampling.

### Study site

Oregon coastal waters are a documented PCFG foraging ground between June and November each year (Newell and Cowles, 2006; Torres *et al.,* 2018). Here, these gray whales feed on a diversity of invertebrates (including mysids, amphipods and crab larvae; Hildebrand *et al.,* 2022) in a nearshore ecosystem (<3 km from shore) with a variable soundscape influenced by natural and anthropogenic sources, which include vessel traffic (Lemos *et al.,* 2022*a*; Haver *et al.,* 2023) that can influence gray whale behaviour (Sullivan and Torres, 2018).

### Data collection

**Figure 1:**
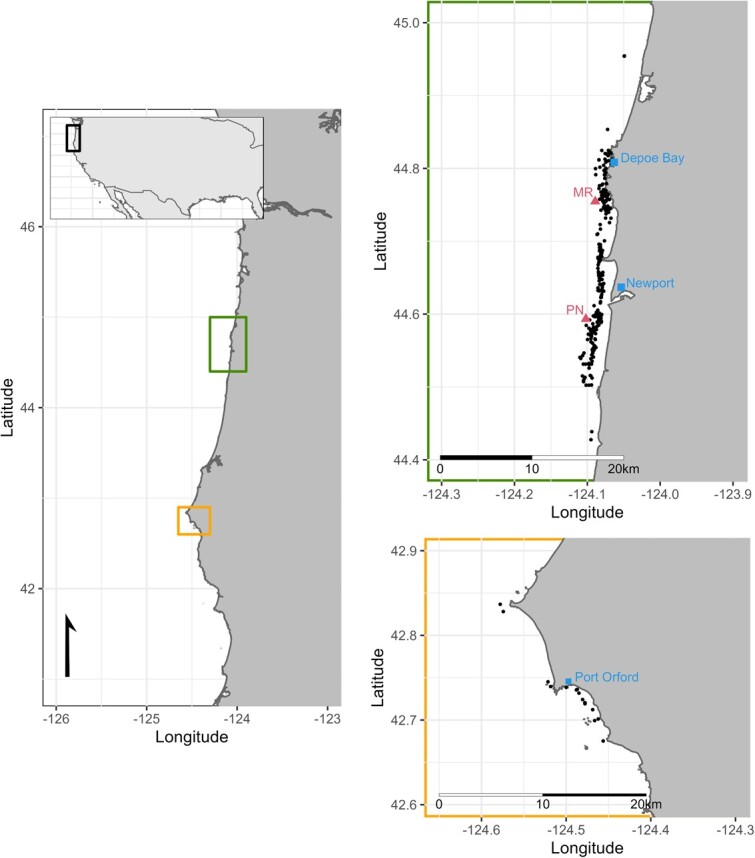
Map of the study area and locations of faecal sampling (represented as black dots). Major ports are marked as blue squares, while the red triangles indicate the hydrophone locations (Port of Newport, PN; and Marine Reserve, MR).

Gray whale faecal samples were collected from a small research vessel over seven foraging seasons (late May to mid-October 2016–2022) along the coast of Oregon, USA (Fig. 1), as described in Lemos *et al.* (2022*a*). Samples were frozen at −20°C until analysis, which occurred within 11 months of collection for all samples. Details of faecal sample preparation, hormone extraction and assays have been described previously (Lemos *et al.,* 2020*b*, 2022a); in brief, samples were filtered to remove seawater and rinsed with distilled water to remove salt, freeze-dried, weighed to the nearest 0.0001 g and hormones were extracted with 30 min vortexing in 90% methanol (Methanol HPLC grade, Fisher Chemical™), after which samples were dried and resuspended in assay buffer. Samples weighing less than 20 mg were excluded from analysis because such samples do not yield reliable hormone data (Lemos *et al.,* 2020*b*). After extraction, commercial enzyme immunoassay kits for cortisol (#ADI-900-071), progesterone (#ADI-900-011) and testosterone (#ADI-900-065) from Enzo Life Sciences (https://www.enzolifesciences.com) and for T3 from Arbor Assays (#K056-H1, https://www.arborassays.com) were used to quantify the concentrations of GC, progestin, androgen and thyroid faecal metabolites, respectively (i.e. the metabolized faecal breakdown products of the parent hormones). All assays have previous passed parallelism and accuracy validations for gray whale faecal extract (Lemos *et al.,* 2020*b*) and employed standard QA/QC, including assay of full standard curves, controls, samples, non-specific binding wells and zero-dose wells in duplicate in every plate and re-assay of any sample with coefficient of variation between wells >15%. Intra- and inter-assay variations were all below 15%; for additional details including antibody cross-reactivities, see Lemos *et al.* (2020*b*, 2022a). Final data are expressed in ng of immunoreactive hormone per g of dried faecal powder.

Aerial photogrammetry data were collected from unoccupied aircraft systems (hereafter ‘drones’; see Supplementary Material, Table S2 for details of drone types), deployed from the survey vessel as described in Lemos *et al.* (2022*b*). Aerial imagery was processed to derive total length and Body Area Index (BAI), a unitless and length-standardized metric of body condition (Burnett *et al.,* 2019), following methods of Bierlich *et al.* (2021) that accommodate the uncertainty associated with different drone types. Individual whales were photo-identified by comparing photographs taken in the field with catalogues for the PCFG held by the Marine Mammal Institute at Oregon State University and Cascadia Research Collective (Olympia, WA, USA). Sex was derived from existing information in the catalogues, previous genetic analyses (Lang *et al.,* 2014), or genetic analyses of the faecal samples (detailed methods in Lemos *et al.,* 2020*a*). Age in years was estimated from the date of first sighting, which provided either a minimum estimate or a known age (for whales that were first sighted as calves). Based on the mean age of sexual maturity for the species (Rice and Wolman, 1971), individuals 8 years old or older were classified as mature, while younger individuals were considered juveniles (see Supplementary material for an example of an alternative analysis using the maximum reported age of sexual maturity, i.e. 12 years; Supplementary Material, Fig. S9). As sexual maturity in mysticetes may be determined by body size, large individuals, even if young, were considered mature if at least 50% of the posterior predictive distribution of their total length estimated from photogrammetry was greater than the mean length at maturity for gray whales (11.1 m for males and 11.7 m for females and individuals with an unknown sex; Rice and Wolman, 1971). As a result, 15 individuals <8 years old were classified as ‘mature’ based on their length. Mature females were classified as lactating in a given year if they were repeatedly sighted in close association with calves (i.e. individuals < 8 m in length); consequently, these females were classified as pregnant in the previous year.

Daily counts of recreational vessels transiting to and from the ports of Newport and Depoe Bay were available from the Oregon Department of Fish and Wildlife through video analysis at the ports. Vessel counts only included recreational craft, categorized as either commercial charters or private vessels, whereas commercial fishing, research and government vessel counts were not available.

Ambient soundscape conditions in the study area were obtained from passive acoustic monitoring (PAM). Specifically, in each year (except in 2016) hydrophones were deployed at two locations (outside the Port of Newport: 44.59°N, 124.10°W; and near the Otter Rock Marine Reserve: 44.75°N, 124.09°W) concurrently with the gray whale data collection (Supplementary Material, Table S1). The hydrophone at the Port of Newport location was lost in 2021 following a storm, so no acoustic data were available from this location in 2021. The PAM system is described in detail in Lemos *et al.,* 2022a and Haver *et al.* (2023). Briefly, it included an omni-directional hydrophone (International Transducer Corporation transducer model ITC1032) with sensitivity −192 dB re μPa V^−1^ @ 1 m, recording at 32 kHz sample rate on a 20% duty cycle (12 min of every hour). Following the methods described in Haxel *et al.* (2013), root mean square sound pressure levels (SPL_rms_) were calculated from two frequency bands: 50 Hz–1 kHz and 1–4 kHz. These bands were chosen to distinguish contributions to the soundscape at low and high frequencies, and to cover the hypothesized communication range of gray whales (Dahlheim *et al.,* 1984), which have a consistent repertoire of calls concentrating in frequencies below 1500 Hz but with a frequency range approaching 5 kHz (Crane and Lashkari, 1996). Frequencies above 4 kHz were excluded to help minimize the effect of wind on the measured underwater sound levels (Lemos *et al.,* 2022*a*; Haver *et al.,* 2023), which concentrates in the frequency band 400 Hz–20 kHz with a dominant frequencies around 8 kHz (Hildebrand *et al.,* 2021). An exploration of the correlations between wind speed, vessel counts and sound levels is reported in the Supplementary Material, Figs S17–S23). In June–July 2021, a seismic survey was conducted along the outer coast of British Columbia, Washington and Oregon. In the Supplementary material, we report the distribution of sound levels and GC concentrations in 2021 compared to other years (Supplementary Material, Figs S3–S5).

### Data processing and exploration

Sound levels were summarized over 24 h and 48 h prior to the faecal sampling. Moreover, we also considered the interval between 5 AM and 6 PM in the day prior to sampling, because previous work and data exploration showed that most anthropogenic sound was concentrated during the daytime (Lemos *et al.* (2022*a*) and Supplementary Material, Fig. S1). Gut passage time in large cetaceans is unknown, but these temporal windows were selected based on a theorized 1–2 day time lag between exposure to stressors and excretion of GC metabolites in the faeces in baleen whales (Wasser *et al.,* 2000; Rolland *et al.,* 2012; Lemos *et al.,* 2022*a*). Over these three temporal windows, we computed three summary metrics to describe sound levels (median, variance and 95th percentile) from the SPL_rms_ time series in the two frequency bands (50 Hz–1 kHz and 1–4 kHz), at the two hydrophone locations. Each faecal sample was then associated with the resulting sound variables from the hydrophone location closest to the position where the sample was collected (see Supplementary material for the exploration of an alternative method of spatially linking faecal samples to relevant sound variables; Supplementary Material, Fig. S12 and S13). While whales move within the study region, sightings of the same individual 24-h and 48-h apart were, on average, within less than 2.5 km and 3.2 km of each other, respectively (Supplementary Material, Fig. S2), suggesting that individuals frequently remain in the same portion of the study area over the span of multiple days. As in Lemos *et al.* (2022*a*), the location of faecal sampling was therefore assumed to reflect the whale’s main location and stressor exposure over the temporal windows under analysis. Vessel counts (charters, private vessels and total recreational craft) in the previous day or two days prior to the sampling dates were also associated with each faecal sample. Faecal samples were paired with the nearest BAI estimate of that whale within 14 days of the sampling date, if available (as gray whale body condition does not significantly change over a two-week period; Lemos *et al.,* 2022*b*). BAI estimates were included without uncertainty in the models, but in the Supplementary material we show that incorporating this uncertainty did not affect the results (Fig. S11).

### Data availability and gaps

The final dataset included 291 faecal samples, collected over seven years from 86 individuals (42 females, 32 males and 12 unknowns; age was known for 27% of individuals, while for the rest of individuals we only recorded a minimum age). The majority of samples (68%) were collected from mature males or mature non-pregnant, non-lactating females (hereafter, ‘mature’), followed by juveniles (24%), pregnant (3%) and lactating females (1%); reproductive status was unknown for 3% of the samples. There were seven individuals that were sampled twice on the same day; our statistical approach avoids pseudoreplication by treating these paired samples as repeats for those individuals (see *Statistical analysis*). GC concentration was undetectable in 16 faecal samples that were diluted more than other samples (due to limited sample volume); these samples were excluded from further analysis. One sample assayed at normal concentration had GC concentration below the limit of detection of the assay; this sample was included with censoring. Therefore, the final sample size used for the analyses was 275.

Several explanatory variables had missing values, but these data points were not discarded because the analytical approach could accommodate their inclusion (see *Statistical analysis*). Specifically, thyroid hormone metabolites were not assayed in the 33 faecal samples collected in 2016 (11%). Paired BAI was not available for 90 samples; for the remaining 201 samples, BAI was measured on the same date as 45% of faecal collections, and within 14 days for the rest. Missing sound and vessel data varied depending on the metric, but, notably, no associated sound information was available for any of the 33 faecal samples from 2016 (no hydrophones deployed) and for the 18 faecal samples collected near the Port of Newport hydrophone in 2021 (hydrophone not recovered after storm). Moreover, there were 16 faecal samples collected from a region further south (around Port Orford, OR); these samples were retained, but the associated stressor variables (i.e. all vessel and sound covariates) were considered missing (see Supplementary Material, Fig. S10, Supplementary material where we show that excluding these samples from the analysis did not change the results).

### Statistical analysis

We investigated the relationship between stressor variables and GC concentrations in each faecal sample using a generalized additive modelling (GAM) framework (Wood, 2006). GAMs allow for the estimation of smooth relationships between the response and explanatory variables, which we deemed suitable for capturing potentially non-linear associations in our dataset. Because GC concentrations are restricted to positive values and their distribution is right skewed (as it is typical for wildlife endocrine samples to include a few with high GC content), we assumed a Gamma distribution of the residuals and used a logarithmic link function. See Supplementary Material, Figs S6–S8 for an alternative formulation of the model that focused on potential physiological thresholds of GCs above baseline; in this model, GC concentrations were transformed into binary values above/below either the 75th or the 95th percentile (GC concentration = 18.5 or 33.1 ng · g^−1^).

In all models, we included a set of covariates representing potential contextual variables that could affect GC concentrations, based on the exploratory data analysis and on information from the literature (Hunt *et al.,* 2006; Corkeron *et al.,* 2017). Specifically, we included the sex of an individual (male or female), its reproductive status (mature, juvenile, pregnant or lactating), individual age (in years), the log-transformed concentrations of the other hormone metabolites in the sample (progestin, androgen and thyroid metabolites), BAI, day of the year (DOY) and year. Sex and status were treated as factors, DOY and age were included as linear terms, while the other covariates were included as smooth terms. Year was modelled as a random effect, using a *t*-distribution with 1 degree of freedom to accommodate larger residuals at the extremes, as evidenced by preliminary analyses. The collinearity among contextual variables was assessed using the Variance Inflation Factor from a preliminary version of the model that only included complete records (i.e. excluding all missing values) and was found to be negligible. Similarly, we tested the inclusion of a random effect by individual in a simplified model, but this inclusion was not warranted by AIC.

In addition to contextual covariates, the models included the stressor variables of interest, i.e. the metrics of sound levels and vessel traffic. Specifically, for sound levels, we included one sound variable at a time, cycling across frequency bands (50 Hz–1 kHz, or 1–4 kHz), temporal windows (24 or 48 h prior to sampling, or between 5 AM and 6 PM in the day prior to sampling), and summary metrics (median, variance and 95th percentile). For vessel counts, we cycled across different time windows (1 or 2 days prior to sampling) and vessel types (charter, private and total). Based on the exploratory data analysis, we investigated the potential interactions between these stressor variables and three factors: the sex of an individual, their BAI and the area where the animal was sampled (i.e. in the sound models, the location of the closest hydrophone; in the vessel models, the location of the closest port). Therefore, we also ran alternative versions of the model where each stressor variable was allowed to interact with each of these factors (i.e. a separate spline was estimated for each level). BAI is a continuous metric, but sample size was too small to fit a bidimensional spline to investigate its interaction with the stressor variables. Instead, we used the median BAI across all individuals (BAI = 26.9) to convert it into a categorical variable: low (i.e. less than the median) vs. high BAI (i.e. equal or greater than the median); in these models, the smooth term for BAI was substituted with the categorical BAI variable. All smooth terms were included as thin plate regression splines with shrinkage (Marra and Wood, 2011).

The basic GAM framework required some modifications due to specific features in our dataset, and, to accommodate these complexities, it was fitted in a Bayesian framework. First, we allowed response values to be left-censored, which applied to samples where GC concentration was below the limit of detection of the assay. Moreover, some individuals were sampled repeatedly within the same day (time difference: 0.4–3.2 h). We treated these samples as repeats of the physiological status of those individuals on those days, and we included an observation model to quantify and account for sampling variability. Specifically, the GC concentration measured in each sample was assumed to emerge from a normal distribution around the true underlying concentration on that day, with standard deviation σ. Finally, there were missing values across several of the explanatory variables, with varying degree of prior information available depending on the variable. Age in years was only known for some individuals, but, for the others, a minimum estimate of age was available. We used minimum age as the lower boundary of a uniform prior distribution, while the upper boundary was the maximum known age across individuals (i.e. 34 years) plus 20 years. Some concentrations of the progestin, androgen and thyroid hormone metabolites were below the corresponding limit of detection of the assay provided by the manufacturer; the limit of detection was thus used as the upper boundary of a uniform prior distribution for these observations. The sex and reproductive status of some individuals were unknown, and a population sex ratio of 1:1 was assumed to define the prior distribution for unknown sex. For reproductive status, we used the observed proportions of different classes in our data as the prior distribution. Missing values in sound levels, vessel counts, BAI and thyroid hormone metabolites (in 2016) were assumed to have a uniform prior distribution ranging between the minimum and the maximum values observed in the data.

Markov chain Monte Carlo (MCMC) algorithms were implemented in software JAGS ver. 4.3.0, run through package runjags (Denwood, 2016) for R (R Core Team, 2023). We used the function jagam in package mgcv (Wood, 2006) to build the spline bases and the design matrix, as well as other package functionalities to summarize the posterior results. The resulting code and R objects were manually modified to accommodate the features described above into the analysis (see code provided on the Open Science Framework https://osf.io/9rgqc/). Three chains were iterated in parallel for 100 000 iterations after an adaptation phase of 5000 iterations (only 1 in 10 iterations was retained to limit object size). Standard diagnostic tools were used to ensure that the chains mixed appropriately and converged to the posterior distribution (Lunn *et al.,* 2013). Residual plots were also used to investigate the distribution of model residuals.

The relevance and strength of the estimated relationships for the smooth terms were assessed using the effective degrees of freedom (edf; Marra and Wood, 2011) and, as is standard in Bayesian analyses (Gelman *et al.,* 1995), the uncertainty around the estimated relationships (as represented by the 95% credible intervals (CI) returned by the plotting functionalities in the mgcv package). For parametric terms, we assessed whether the 95% CI of the corresponding coefficients overlapped with 0, indicating a probability greater than 0.05 that there was no relationship between those terms and GC concentrations (Gelman *et al.,* 1995).

**Figure 2:**
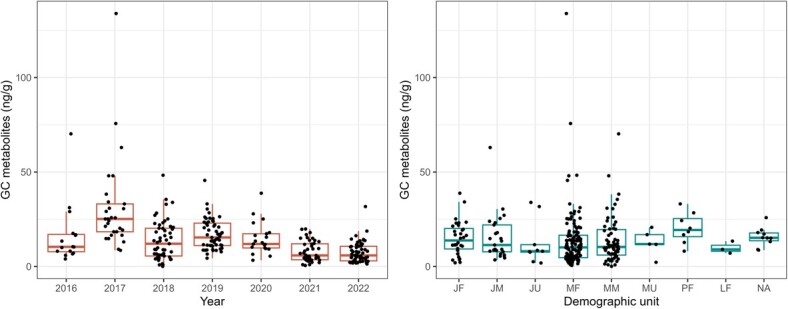
Distribution of GC metabolite concentrations across different years and demographic units. Demographic units are indicated by two letters; the first represents reproductive status (Juvenile, Mature, Pregnant or Lactating), while the second is sex (Male, Female or Unknown). When both are missing, samples are marked as NA on the x-axis. GC concentrations appeared to be highest in 2017, among pregnant females and in some mature males and females, and lowest in 2021–2022; however, the associated variability was large.

## Results

The exploratory data analysis indicated that GC concentrations varied both among years and also as a function of individual reproductive status and sex (Fig. 2). The variation across other variables appeared more inconsistent when explored in isolation. The GAMs converged rapidly, and all samples after the adaptation phase could be used for posterior inference. Model residuals did not highlight any major problem with model fitting (see Supplementary Material, Fig. S14, Supplementary material for one model example). Importantly, there was no spatial or temporal pattern in the model residuals, suggesting that we were not missing any source of substantial GC variation in space or time (Supplementary Material, Fig. S15 and S16). Across GAMs, the standard deviation of the observation model for GC concentration (σ) had a posterior mean varying in the range 3.8–4.1 ng · g^−1^ (SD: 0.4 ng · g^−1^), and measured GC concentrations were estimated to deviate from their true underlying value by, at most, 12 ng · g^−1^. This estimate captures the differences in GC concentration due to sampling variability between repeated samples collected from the same individual in one day.

**Table 1:** Summary of all models, including different combinations of stressor variables (sound levels or vessel counts), summarized at multiple temporal scales, and interacting factors (sex, BAI and location, as represented by the closest hydrophone or the closest port)

Stressor	Interaction	Frequency band	Time window	Metric	edf – reference level	edf – contrast	CI – reference level	CI –contrast	CI – BAI effect
Sound levels	Sex	50 Hz–1 kHz	5 AM–6 PM	Median	1.04	1.02			
Sound levels	Sex	50 Hz–1 kHz	5 AM–6 PM	95th percentile	1.02	1.02			
Sound levels	Sex	50 Hz–1 kHz	5 AM–6 PM	Variance	1.02	1.41			
Sound levels	Sex	50 Hz–1 kHz	24 h	Median	1.14	0.95			
Sound levels	Sex	50 Hz–1 kHz	24 h	95th percentile	1.05	1.07			
Sound levels	Sex	50 Hz–1 kHz	24 h	Variance	1.02	0.93			
Sound levels	Sex	50 Hz–1 kHz	48 h	Median	1.27	0.99			
Sound levels	Sex	50 Hz–1 kHz	48 h	95th percentile	0.98	0.94			
Sound levels	Sex	50 Hz–1 kHz	48 h	Variance	1	0.96			
Sound levels	Sex	1 kHz–4 kHz	5 AM–6 PM	Median	1	1.21			
Sound levels	Sex	1 kHz–4 kHz	5 AM–6 PM	95th percentile	1	1.03			
Sound levels	Sex	1 kHz–4 kHz	5 AM–6 PM	Variance	1.05	1.17			
Sound levels	Sex	1 kHz–4 kHz	24 h	Median	1.08	1.01			
Sound levels	Sex	1 kHz–4 kHz	24 h	95th percentile	0.99	1			
Sound levels	Sex	1 kHz–4 kHz	24 h	Variance	1.05	0.99			
Sound levels	Sex	1 kHz–4 kHz	48 h	Median	1.08	1.03			
Sound levels	Sex	1 kHz–4 kHz	48 h	95th percentile	0.98	0.92			
Sound levels	Sex	1 kHz–4 kHz	48 h	Variance	1.01	0.97			
Sound levels	Binary BAI	50 Hz–1 kHz	5 AM–6 PM	Median	1.15	0.94			
Sound levels	Binary BAI	50 Hz–1 kHz	5 AM–6 PM	95th percentile	1	1.04		*	
Sound levels	Binary BAI	50 Hz–1 kHz	5 AM–6 PM	Variance	0.99	1.23	*		
Sound levels	Binary BAI	50 Hz–1 kHz	24 h	Median	1.37	1	*		*
Sound levels	Binary BAI	50 Hz–1 kHz	24 h	95th percentile	1.08	1.06	*	*	
Sound levels	Binary BAI	50 Hz–1 kHz	24 h	Variance	0.96	1.03		*	*
Sound levels	Binary BAI	50 Hz–1 kHz	48 h	Median	1.36	1.05	*		*
Sound levels	Binary BAI	50 Hz–1 kHz	48 h	95th percentile	1.05	0.97	*		
Sound levels	Binary BAI	50 Hz–1 kHz	48 h	Variance	0.96	1.06	*	*	*
Sound levels	Binary BAI	1 kHz–4 kHz	5 AM–6 PM	Median	1.28	0.99			
Sound levels	Binary BAI	1 kHz–4 kHz	5 AM–6 PM	95th percentile	0.96	1.02			
Sound levels	Binary BAI	1 kHz–4 kHz	5 AM–6 PM	Variance	0.97	1.11			
Sound levels	Binary BAI	1 kHz–4 kHz	24 h	Median	1.56	0.97			*
Sound levels	Binary BAI	1 kHz–4 kHz	24 h	95th percentile	0.91	1.03		*	
Sound levels	Binary BAI	1 kHz–4 kHz	24 h	Variance	0.98	1.06		*	*
Sound levels	Binary BAI	1 kHz–4 kHz	48 h	Median	1.37	1			*
Sound levels	Binary BAI	1 kHz–4 kHz	48 h	95th percentile	0.97	0.97			
Sound levels	Binary BAI	1 kHz–4 kHz	48 h	Variance	0.95	1.03			*
Sound levels	Closest HP	50 Hz–1 kHz	5 AM–6 PM	Median	1.06	0.94			
Sound levels	Closest HP	50 Hz–1 kHz	5 AM–6 PM	95th percentile	0.97	1.03			
Sound levels	Closest HP	50 Hz–1 kHz	5 AM–6 PM	Variance	0.93	1.1			
Sound levels	Closest HP	50 Hz–1 kHz	24 h	Median	1.03	1.33			
Sound levels	Closest HP	50 Hz–1 kHz	24 h	95th percentile	1.05	1.01			
Sound levels	Closest HP	50 Hz–1 kHz	24 h	Variance	0.91	0.88		*	
Sound levels	Closest HP	50 Hz–1 kHz	48 h	Median	0.94	1.08			
Sound levels	Closest HP	50 Hz–1 kHz	48 h	95th percentile	0.91	0.98			
Sound levels	Closest HP	50 Hz–1 kHz	48 h	Variance	0.85	0.84			
Sound levels	Closest HP	1 kHz–4 kHz	5 AM–6 PM	Median	1.61	1.02			
Sound levels	Closest HP	1 kHz–4 kHz	5 AM–6 PM	95th percentile	0.93	0.91			
Sound levels	Closest HP	1 kHz–4 kHz	5 AM–6 PM	Variance	0.95	0.99			
Sound levels	Closest HP	1 kHz–4 kHz	24 h	Median	0.97	0.94			
Sound levels	Closest HP	1 kHz–4 kHz	24 h	95th percentile	1.05	0.91			
Sound levels	Closest HP	1 kHz–4 kHz	24 h	Variance	0.85	0.97		*	
Sound levels	Closest HP	1 kHz–4 kHz	48 h	Median	1.05	1.07			
Sound levels	Closest HP	1 kHz–4 kHz	48 h	95th percentile	0.66	0.89			
Sound levels	Closest HP	1 kHz–4 kHz	48 h	Variance	0.78	0.96			
Vessel counts	Sex	-	1 d prior	Total	1.13	1.02			
Vessel counts	Sex	-	1 d prior	Private	1.1	0.99			
Vessel counts	Sex	-	1 d prior	Charters	1.06	1.25		*	
Vessel counts	Sex	-	2 d prior	Total	1	1.08			
Vessel counts	Sex	-	2 d prior	Private	1.06	1.15			
Vessel counts	Sex	-	2 d prior	Charters	1.04	1.17		*	
Vessel counts	Binary BAI	-	1 d prior	Total	1.02	1.06			
Vessel counts	Binary BAI	-	1 d prior	Private	1	1.05			
Vessel counts	Binary BAI	-	1 d prior	Charters	1.16	1.09			
Vessel counts	Binary BAI	-	2 d prior	Total	1	1.09			
Vessel counts	Binary BAI	-	2 d prior	Private	1.07	1.09			
Vessel counts	Binary BAI	-	2 d prior	Charters	1.28	1.06			
Vessel counts	Closest port	-	1 d prior	Total	1.01	1.08			
Vessel counts	Closest port	-	1 d prior	Private	0.99	1.09			
Vessel counts	Closest port	-	1 d prior	Charters	1.03	1.1			
Vessel counts	Closest port	-	2 d prior	Total	1	1.04			
Vessel counts	Closest port	-	2 d prior	Private	1.05	1.12			
Vessel counts	Closest port	-	2 d prior	Charters	0.96	1.18			

The edf for the spline relationships estimated for the reference and contrast levels are reported. The last three columns are marked with an ‘*’ symbol if the corresponding credible interval (CI) did not fully include the 0 line.

**Figure 3:**
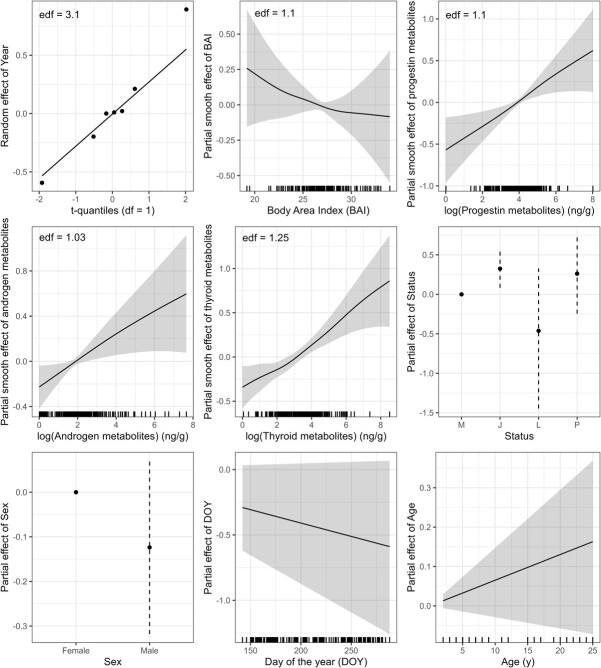
Estimated relationships between faecal GC metabolite concentrations and all contextual variables, reported for the model including the interaction between sex and the 95th percentile of sound levels in the high frequency band (1–4 kHz) between 5 AM and 6 PM in the day prior to sampling. Each panel reports the posterior effect, with the shaded areas representing the 95% credible intervals. For smooth relationships, the effective degrees of freedom (edf) of the spline are also reported on each panel. The random effect for year was well captured using a *t*-distribution, except for one year (2017) that deviated from the expected distribution. GC concentrations decreased with increasing body area index (BAI) and increased with the log-transformed concentration of the other hormone metabolites. They were also higher in juveniles, while the effect of lactation and pregnancy status overlapped with 0. The effects of sex, day of the year (DOY) and age overlapped with 0.

All results of the statistical analysis are discussed in terms of the shape of the estimated relationships between the explanatory variables and the response variable (i.e. GC concentrations of an individual whale on a given day), with an evaluation of the edf after shrinkage (whether or not these were greater than 1; Marra and Wood, 2011) and/or the associated 95% credible intervals (whether or not these included 0; Gelman *et al.,* 1995). The results across all models are reported in Table 1 and in Supplementary material. The relationships between GC concentrations and the set of contextual variables (year, BAI, other hormone metabolites, reproductive status, sex, DOY and age) remained largely unvaried across all models, highlighting several important sources of baseline variation in GC concentrations in gray whale faecal samples (reported for one example in Fig. 3). Specifically, GC concentrations were estimated to increase almost linearly as a function of the logarithmic concentration of the other hormones–progestin, androgen and thyroid metabolites. GC concentrations were also higher in juveniles and pregnant females, but the CI of the latter effect overlapped with 0. The effect of lactation status on GC concentrations was estimated to have large CI overlapping with 0. Males were estimated to have lower GC concentrations than females on average, but this effect also overlapped with 0. GC concentrations declined with DOY and increased with age, but the CI of these relationships were large and, depending on the model, generally overlapped with 0. The smooth relationship between GC concentrations and BAI showed a negative trend (i.e. lower BAI associated with higher GC concentration) and the edf were greater than 1 in most models, but the CI was wide and always included the 0 line. In the models involving an interaction between the stressor variables and BAI, where BAI was included as a binary factor (i.e. smaller, or greater than the median BAI), being in a high-BAI state was associated with lower GC concentrations, and this effect did not overlap with 0 in 40% of these models. The random effect of year followed the *t*-distribution, except for one year (2017) where the effect was larger than expected despite the long tail of the distribution (Fig. 3).

**Figure 4:**
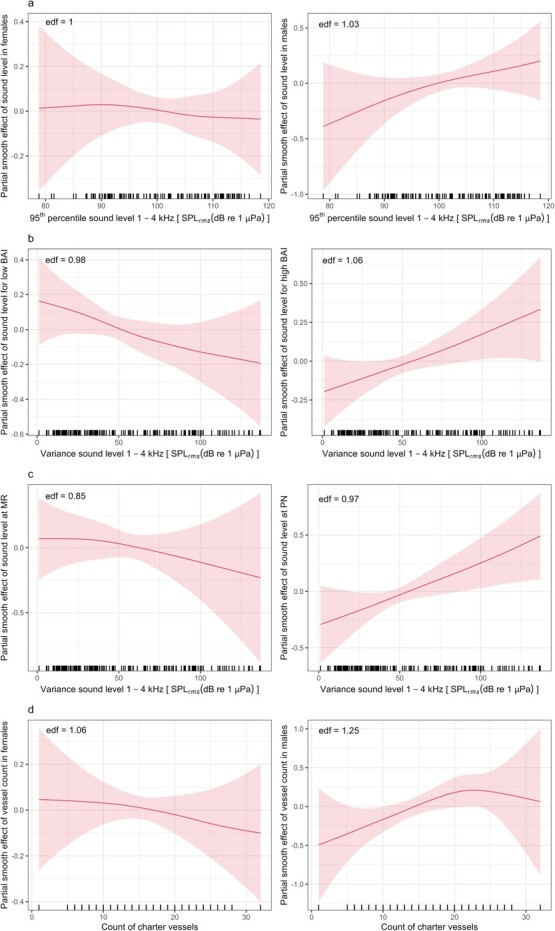
Estimated relationships between faecal GC metabolite concentrations and a subset of stressor variable combinations. In (a), interaction between sex and the 95th percentile of sound levels in the high-frequency band (1–4 kHz) between 5 AM and 6 PM in the day prior to sampling; GC concentrations increased with sound level in males (right panel), but not in females (left panel). In (b), interaction between Body Area Index (BAI) and the variance of sound levels in the high-frequency band (1–4 kHz) in the 24 h prior to sampling; GC concentrations decreased with sound level for individuals with low BAI (left panel) and increased with sound level for individuals with high BAI (right panel). In (c), interaction between closest hydrophone location (Marine Reserve, MR, and Port of Newport, PN) and the variance of sound levels in the high-frequency band (1–4 kHz) in the 24 h prior to sampling; GC concentrations increased with sound level at PN (right panel), but not at MR (left panel). In (d), interaction between sex and the count of charter vessels in the day prior to sampling; GC concentrations showed an increasing trend with the count of charter vessels in males (right panel), but not in females (left panel). Each panel reports the posterior effect, with the shaded areas representing the 95% credible intervals. The effective degrees of freedom (edf) of the spline are also reported on each panel.

After accounting for the effects of the contextual variables, there was large variability in the direction and relevance of the effects of the sound stressor variables, which depended on the interacting factors (sex, BAI and location) and, for the sound variables, on the frequency band (50 Hz–1 kHz or 1–4 kHz), temporal window prior to sampling (24 or 48 h prior to sampling, or between 5 AM and 6 PM in the day prior to sampling) and summary metric (median, 95th percentile or variance). However, some overall trends emerged when considering the interactions of the stressor variables with sex, BAI and location. Specifically, when the sound variables interacted with sex, the estimated relationships suggested that GC concentrations increased in males with increasing sound levels in the high frequency band (1–4 kHz), across all time windows and metrics. In contrast, the corresponding relationships between GC concentrations and sound variables in females showed a more inconsistent and, in some cases, declining trend. However, for both sexes, the CI always included the 0 line, suggesting that there was some probability (variable across models) of no relationship with these stressor variables. We note that the CI were narrower for the relationship with the 95th percentile of sound levels in 1–4 kHz in the window 5 AM–6 PM of the previous day for males (Fig. 4). When considering the effect of sound levels in the lower frequency band (50 Hz–1 kHz), estimated relationships were inconsistent: the positive relationship between GC concentrations and sound variables in males was visible when considering the previous day between 5 AM and 6 PM, and the median or 95th percentile metrics, but again the CI included the 0 line. The interaction between the sound variables and the binary BAI variable showed that GC concentrations tended to increase with increasing sound levels in individuals with BAI ≥ median, and decrease in individuals with BAI < median (Fig. 4), particularly when considering the variance and 95th percentile of sound levels, in both frequency bands. However, only in some combinations of conditions were the edf ≥ 1 and the 0 line excluded from the CI (Table 1). Finally, the results of the models including the interaction between sound levels and the location of the closest hydrophone were variable, but in some models we found that GC concentrations increased with sound levels at higher frequencies for animals sampled near the Port of Newport location (e.g. Fig. 4; Table 1). The CI mostly included the 0 line, with the exception of the relationship between GC concentrations and sound level variance in the previous 24 h in both frequency bands, but even these splines had edf < 1 (Table 1).

The estimated relationships between GC concentrations and vessel counts, evaluated separately for the two sexes, low and high BAI and the two ports, showed a large uncertainty. The positive relationships between GC concentrations in males and the count of charter vessels 1 or 2 days prior to the sampling were the only cases where the posterior CI did not fully include the 0 line (Fig. 4, Table 1).

## Discussion

We estimated the relationships between short-term variation in levels of stressors (sound and vessels) and faecal GC concentrations in a wild marine mammal species. Our results quantify the effect of contextual variables on baseline GC concentrations and demonstrate large variability in the estimated relationships between faecal hormone levels and exposure to stressors, highlighting the importance of individual context in modulating short-term physiological variation associated with disturbance. We outline an analytical approach that captures the potentially non-linear nature of changes in hormone concentrations and accommodates the complexities of physiological data (e.g. missing values, repeated samples, sampling variability, concentrations below the limit of detection). Crucially, the analysis allows incorporation of the many other variables that affect GC concentrations (e.g. reproductive status, sex, age, body condition, year, time of year) (Dickens and Romero, 2013; Dantzer *et al.,* 2014; MacDougall-Shackleton *et al.,* 2019).

Among these contextual variables, the other hormones (progestins, androgens, thyroid hormones) showed the strongest association with GC concentrations, as seen in previous studies (e.g. Jelincic *et al.,* 2017; Lemos *et al.,* 2022*b*). This pattern could emerge from a combination of two processes: first, reproductive status and metabolic status are known to affect stress physiology (Dantzer *et al.,* 2014). This effect can be so pronounced that prior studies of effects of stressors on GC concentrations (e.g. Rolland *et al.,* 2012) have had to exclude all high-progestin and high-androgen samples. Our analytical approach, in contrast, can discriminate the contributions of other hormones to GC concentrations. Alternatively, these positive correlations could indicate a methodological artefact by which some gray whale faecal samples have high concentrations of all steroid and thyroid hormones, without necessarily reflecting concentrations in plasma. A similar phenomenon has been noted in terrestrial faecal-hormone studies (KEH, pers. obs.) and may be due to variation in dietary lipid or fibre content (Goymann, 2012; Dantzer *et al.,* 2014). Indeed, gray whales in our study area have a variable diet (Hildebrand *et al.,* 2022). However, the lack of strong collinearity among other steroid and thyroid hormones is in contrast with this second hypothesis, suggesting that faecal GC correlations with other hormones may reflect similar relationships in plasma.

Life-history status also affected GC concentrations (higher GCs in juveniles and pregnant females), albeit with large uncertainty (see also Supplementary Material, Fig. S9, Supplementary material). Changes in GCs with maturity have been documented in many mammals (Wada, 2008). Moreover, GC concentrations are expected to vary with female reproductive states such as pregnancy and lactation (Brunton *et al.,* 2008), and baseline GCs can also increase when energy demands are high (MacDougall-Shackleton *et al.,* 2019). Notably, the effect of pregnancy aligns with results from other baleen whales (e.g. Pallin *et al.,* 2022) but differs from previous results from gray whales using blubber samples (Melica *et al.,* 2022), warranting future comparisons across matrices.

The estimated relationships of GCs with the other contextual variables (BAI, day of the year, age) may provide insights into the effects of individual energetic status, season and age on physiology, but the wide CI prevent a conclusive interpretation of these patterns. Nonetheless, animals in good body condition tended to have consistently lower GC concentrations, in line with previous results on a subset of these data (Lemos *et al.,* 2022*b*) and other marine mammal species (e.g. Jeanniard du Dot *et al.,* 2009). Finally, there were differences in GC concentrations across years. However, we did not detect an effect of the gray whale Unusual Mortality Event that began in 2019, which reinforces the finding that PCFG whales were not affected by this event experienced by the broader eastern North Pacific population (Torres *et al.,* 2022).

After accounting for the contextual variables, the relationships with sound levels and vessel counts were generally weak and with high uncertainty. This is unsurprising, as the effects of short-term disturbance are expected to be minor compared to variations in baseline levels of GCs (Dickens and Romero, 2013). Importantly, we found some indication that faecal GC concentrations in males increased with increasing sound levels from the day before, a temporal lag that is consistent with the theorized gut passage times in baleen whales (Wasser *et al.,* 2000; Rolland *et al.,* 2012; Lemos *et al.,* 2022*a*). Particularly, relationships were stronger for the 5 AM to 6 PM window, when smaller recreational vessels are more likely to be transiting to and from ports through gray whale foraging habitat and contributing to sound levels in the higher 1–4 kHz frequency band quantified here (Lemos *et al.,* 2022*a*; Haver *et al.,* 2023). Small vessels that approach and interact with the animals in coastal waters are a source of disturbance in other marine mammal species (e.g. Pirotta *et al.,* 2015) and are expected to represent a significant stressor for gray whales in this area (Sullivan and Torres, 2018). This observation is supported by the detected effect of the count of charter vessels on GC concentrations, which could offer a better proxy of the traffic and sound levels experienced by the whales than the recreational vessel counts that mostly utilize offshore fishing grounds. The more marked effect of the 95th percentile sound levels at higher frequencies on GC concentrations also suggests that individuals may be more responsive to discrete acoustic events of greater intensity, supporting the idea that context and nature of the sound source affects perceived risk (Ellison *et al.,* 2012).

In our study, males showed a stronger association between GC concentrations and sound levels than females, agreeing with prior reports that, in many vertebrates, males are generally more responsive to disturbance (Dantzer *et al.,* 2014). In baleen whales, males also tend to be more vocal (Croll *et al.,* 2002), which could make them more sensitive to changes in the soundscape. Stronger responses in males would not necessarily indicate that males are more vulnerable to possible negative health consequences of stress; rather, males may simply have greater ability to activate the HPA axis to cope with a stressor, while females might have limited physiological scope to do so, for example due to reproduction.

Across frequencies and time lags, GC concentrations tended to increase with sound levels for animals in high BAI status, and decrease otherwise, particularly for the 95th percentile and variance metrics. Albeit variable, this finding is relevant for understanding the effects of nutritional status on the ability to respond to stressors (Romero *et al.,* 2009). The trade-offs between nutritional status and responsiveness to disturbance have been documented from a behavioural perspective, with some evidence that animals in poorer body condition tend to take more risks (Moran *et al.,* 2021), and have been posited to affect the population-level consequences of disturbance (Burslem *et al.,* 2022). Recent findings from terrestrial vertebrates suggest that energy availability may also be an important modulator of an individual’s physiological responses to stressors, as well as affecting subsequent health and fitness impacts (Breuner and Berk, 2019). We speculate that HPA axis may be inhibited in animals that take more risks because of nutritional constraint, or stimulated in individuals in better body condition with greater reproductive potential (Moran *et al.,* 2021), although the underlying mechanisms may be complex (Madliger and Love, 2014; MacDougall-Shackleton *et al.,* 2019). Our results also suggest that the strength and direction of the relationship between sound and GCs may vary with other contextual factors, such as location. Overall, the relevance of these interacting factors indicates a strong context-dependency of any effects of disturbance on GCs, as previously recognized for behavioural (Beale, 2007; Tablado and Jenni, 2017) and physiological changes (Madliger and Love, 2014).

Many other factors also contribute to the estimated uncertainty in the relationships between stressors and GCs. First, sound levels at the closest hydrophone are not necessarily an accurate metric of the cumulative sound an individual was exposed to throughout a given 1-day or 2-day temporal window. Moreover, some of the sound (particularly at higher frequencies) may be attributable to natural sources (e.g. wind, waves) that may not be perceived as stressors by the whales. Similarly, the count of vessels going in and out of the harbour does not necessarily reflect the true level of traffic that an individual encountered or their direct interactions with vessels (Lemos *et al.,* 2022*a*). An exposimeter attached to the body of an animal (e.g. Johnson and Tyack, 2003) would provide more accurate dose metrics at the individual level but would likely yield much smaller sample size. Alternatively, a larger array of hydrophones could be used to better characterize the soundscape at a finer spatial scale, but would require added logistics and expense. Additional sources of variation include the unknown and variable temporal lag between stressor exposure and resultant GC excretion in faeces, the nature of the species’ diet and the consistency of the species’ faecal samples (Fernandez Ajó *et al.,* 2023). While we explicitly included an observation model to capture sampling variability, we only had seven daily repeats. Finally, the magnitude of the physiological variation associated with exposure to the stressors under analysis may simply be small compared to all other factors affecting GC secretion; animals may be habituated to these stressors, or, conversely, GC concentrations may be chronically elevated due to the overall stressors that gray whales experience.

From a methodological perspective, building more mechanistic knowledge into the analysis could support a less uncertain estimation of the relationships between stressors and GC concentrations (Pirotta *et al.,* 2022). Observational hormone studies are also correlative in nature and therefore cannot establish causal links between stressor exposure and observed physiology. Here, we could not estimate formal dose–response relationships, because GC concentrations could not be measured in response to experimental exposures (but see an example of an observational dose–response relationship derived from our results in Supplementary Material, Fig. S24). Prolonged focal follows or experiments involving controlled exposures of animals of different sex or body condition to repeatable stressors (e.g. Southall *et al.,* 2016) and subsequent faecal sampling at different temporal lags would be challenging, but not impossible given the high residency of individual gray whales in the study area during the feeding season. These data could inform physiological dose–response functions that are comparable to those obtained in behavioural studies, which could then be used to simulate responses under variable exposure scenarios and their cascading effects on individuals and populations. These predictive tools will be required to guide appropriate management measures in the face of increased resource extraction, including planned offshore renewable energy developments along the U.S. West coast (e.g. U.S. Wind Energy Technologies Office, 2023).

Broadly, the large variability emerging from our analyses confirms that it is rarely possible to fully interpret the physiological status of a wild animal from an isolated GC measurement (MacDougall-Shackleton *et al.,* 2019). The HPA axis responds to a large variety of inputs, and an extensive suite of additional information about each individual is therefore necessary to correctly interpret GC data and identify the variation associated with a potential stressor (Dickens and Romero, 2013; Dantzer *et al.,* 2014; Romero and Wingfield, 2015). Our results also confirm that physiological responses tend to be highly context dependent, requiring extensive data across combinations of conditions (Tablado and Jenni, 2017). This poses a challenge for management efforts to identify specific sources of disturbance that may have impacts on physiology and, ultimately, vital rates. Untangling this extensive web of factors in a field setting requires a complex data collection effort. Our analytical approach provides one way to tackle the resulting datasets and reveal subtle but potentially important short-term physiological variation associated with stressors. Overall, the demand for large datasets presents a practical obstacle to applying this approach in unstudied populations, or when impact assessments are needed under pressing management needs. Here, we collected a comparatively large sample size for a baleen whale species, but sample numbers remained small when partitioned among contextual factors (e.g. for some reproductive classes). Further expanding this dataset will allow refinement of results in the future and apportioning the observed variation among the various sources of uncertainty.

As our dataset grows, information on life-history events of sampled individuals also accumulates (e.g. growth, injuries, deaths, female reproductive attempts). Linking hormone levels to vital rates will be an exciting next step in the investigation of gray whale physiological response pathways and population-level consequences (Pirotta *et al.,* 2018). Physiological responses to stressors are adaptive and, even if short-term responses can be conclusively detected, these are not necessarily indicative of long-term effects on individual performance (Dickens and Romero, 2013; Dantzer *et al.,* 2014; Breuner and Berk, 2019). A better mechanistic understanding of the processes involved in baleen whale stress physiology is therefore imperative (Madliger and Love, 2014), which will require further theoretical studies (Dickens and Romero, 2013), meta-analyses of the increasing number of empirical studies across species (e.g. Hunt *et al.,* 2017*b*), and, potentially, empirical work on related species that can be handled (e.g. odontocetes; Champagne *et al.,* 2017, 2018; Houser *et al.,* 2021; Schwacke *et al.,* 2023). Physiological studies will also likely benefit from adding a suite of other biomarkers to the GCs, such as aldosterone and dehydroepiandrosterone to confirm HPA activation (e.g. Burgess *et al.,* 2017; Dalle Luche *et al.,* 2021), as well as other physiological markers of energetic status (e.g. body condition), immune status (e.g. inflammation, faecal immunoglobulins) or organ status (e.g. injuries or organ pathologies) (Schwacke *et al.,* 2023), resulting in a multivariate measure of physiological status and health (MacDougall-Shackleton *et al.,* 2019). Further testing of these ‘stress panels’ could help inform multivariate, mechanistic dose–response functions that better describe the effects of exposure to varying levels of stressors on the health of individuals holistically (Romero *et al.,* 2015). Ultimately, understanding the complex relationships among stress biomarkers and across the many relevant ecological and context-dependent variables will be critical to quantify the cascading effects of stressors on health and vital rates, and thus predict long-term population consequences to inform management and conservation efforts in cetaceans and other free-ranging vertebrates (Tyack *et al.,* 2022).

## Supplementary Material

Web_Material_coad082

## Data Availability

Data and code to run the analyses are stored on the Open Science Framework repository (https://osf.io/9rgqc/).
