# Supplementary material for: Assessing variation in faecal glucocorticoid concentrations in gray whales exposed to anthropogenic stressors
Source: Conserv Physiol. 2023 Nov 11;11(1):coad082. doi: 10.1093/conphys/coad082 (PMC10660368; doi:10.1093/conphys/coad082)
Supplement: Web_Material_coad082 [file web_material_coad082.pdf]

Supplementary material

Table of contents

Acoustic data collection 2
Photogrammetry data collection 3
Daily sound level patterns 4
Distance between individuals' sightings 5
Sound levels and GC concentrations in conjunction with the 2021 seismic survey 6
Binary analyses 8
Analysis using a different age of sexual maturity 12
Analysis excluding the data from Port Orford 14
Analysis incorporating the uncertainty in BAI estimates 16
Using a different criterion to compute gray whale sound level exposure 17
Residual diagnostics for one model example 20
Correlations among vessel counts, wind speed and sound levels 22
Complete model results 29
Example of observational dose-response function 91
References 92

Acoustic data collection

Table S1. Hydrophone deployment and retrieval dates in each location and each year.

Year	Location	Deployment dates
2016	Marine Reserve	Not deployed
	Port of Newport	Not deployed
2017	Marine Reserve	15 Jun – 8 Oct
	Port of Newport	15 Jun – 8 Oct
2018	Marine Reserve	6 Jun – 25 Jul & 30 Jul – 3 Oct
	Port of Newport	6 Jun – 25 Jul & 30 Jul – 3 Oct
2019	Marine Reserve	28 Jun – 4 Oct
	Port of Newport	28 Jun – 4 Oct
2020	Marine Reserve	2 Jun – 26 Sep
	Port of Newport	2 Jun – 26 Sep
2021	Marine Reserve	20 May – 22 Nov
	Port of Newport	Lost
2022	Marine Reserve	27 May - 23 Sep
	Port of Newport	27 May - 23 Sep

Photogrammetry data collection

Table S2. Camera specifications associated with each unoccupied aircraft system (UAS) used in this study. Each UAS had a barometer to record the altitude of the drone during video collection, while the Inspire 2 also had a laser altimeter (LiDAR) (Dawson *et al.*, 2017).

UAS	Year	Sensor (mm)	Pixel resolution (px)	Focal length lens (mm)	Altimeter
Phantom 3 Pro	2016-2017	6.16 x 4.6	3840 x 2160	3.61	Barometer
Phantom 4	2016-2017	6.16 x 4.6	3840 x 2160	3.61	Barometer
Phantom 4 Pro	2017-2019	13.2 x 8.8	3840 x 2160	8.8	Barometer
Inspire 2	2020-2022	17.3 x 13	3840 x 2160	25	Barometer & LiDAR

Daily sound level patterns

We investigated the variation in hourly sound levels at the hydrophone location outside the port of Newport, OR. Specifically, we modelled the hourly median root-mean-square sound pressure levels (SPL_{rms}) as a function of hour of the day using a cyclic spline in a generalized additive model (GAM). Here, we present the results for sound levels in the low frequency band (50 Hz – 1 kHz), but results are largely unchanged if the high-frequency (1– 4 kHz) band is used instead.

Model results indicate that sound levels are consistently elevated between 5 AM and 6 PM, in conjunction with most of the boating activity in the area (Fig S1; Lemos *et al.*, 2022).

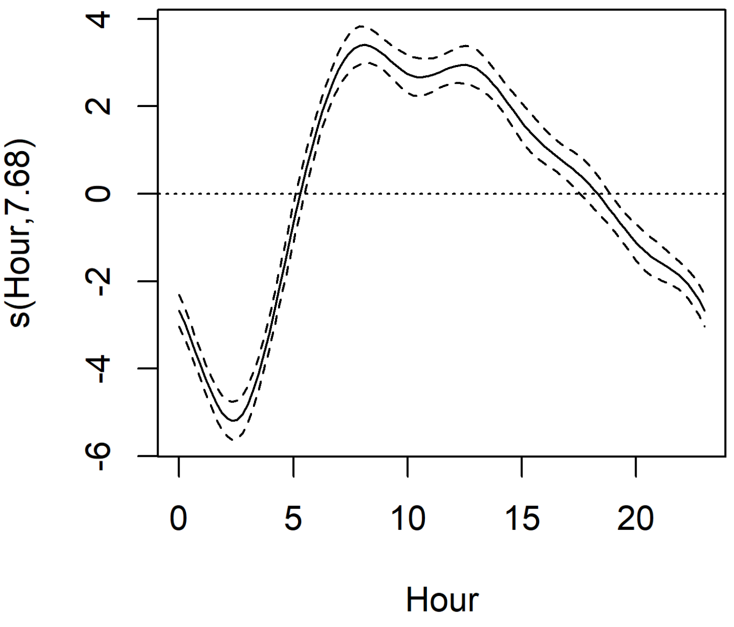

Figure S1. Estimated relationship between hour of the day and hourly median root mean square sound pressure levels in the frequency band 50 Hz – 1 kHz. The plot reports the effective degrees of freedom of the cyclic spline (in brackets), and the 0 line for reference.

Distance between individuals' sightings

In our analyses, we associated each faecal sample to the metrics of sound level recorded at the hydrophone that was closest to the location where an individual was sighted. This assumes that, on average, individuals remained in the same portion of the study area over the temporal windows we used to summarise the soundscape.

Here, we explored this assumption by analysing the distance between sightings of the same individual. Of the 1,874 overall sightings in the period 2016-2022, we considered only the ones where an individual was resighted within a 7-day temporal window. First, we computed the time elapsed between consecutive sightings and their geodesic distance in km. Next, we fitted a generalized additive model (GAM) to estimate a smooth relationship between the time difference and the log-transformed distance. We investigated the inclusion of an individual-level random effect, but this was not supported by Akaike Information Criterion (AIC).

Model results suggested that consecutive sightings 24-h apart were, on average, within less than 2.5 km of each other, while, for sightings that were two days apart, this distance increased to 3.2 km (Fig. S2a). There was variability around this relationship (Fig. S2b), but the mean trend supported our assumption that individuals frequently remain in the same portion of the study area over the span of one to three days, justifying the use of sound level metrics at the closest hydrophone.

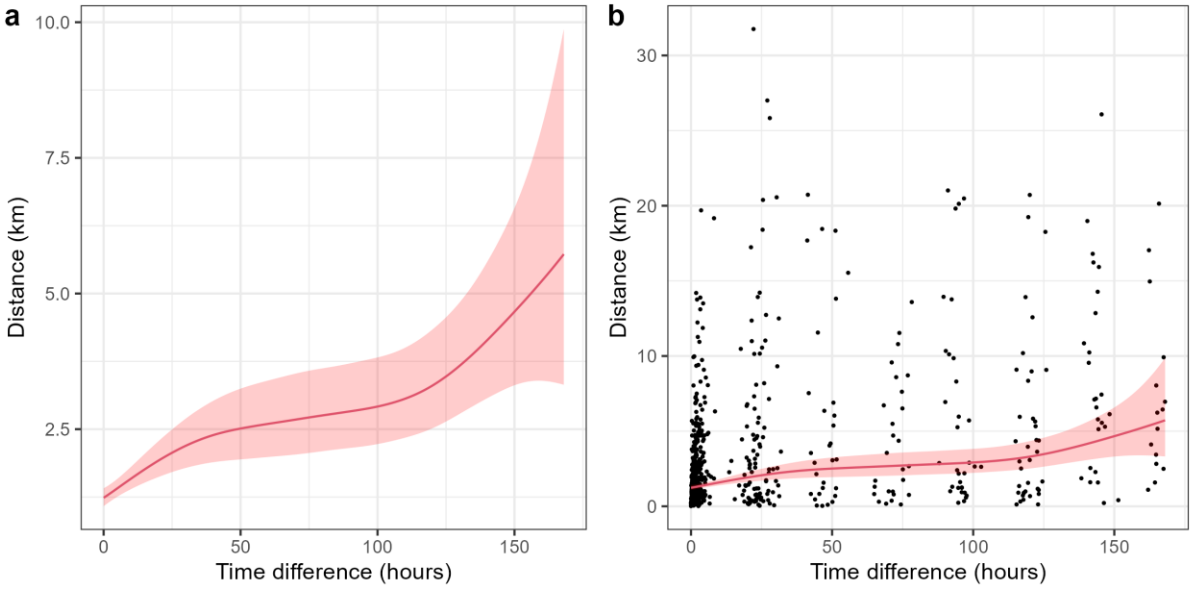

Figure S2. Estimated spline relationship between the time elapsed between consecutive sightings (in hours) and their distance (in km), without (a) and with (b) the data plotted on top of it. This analysis was restricted to sightings within 7 days of each other.

Sound levels and GC concentrations in conjunction with the 2021 seismic survey

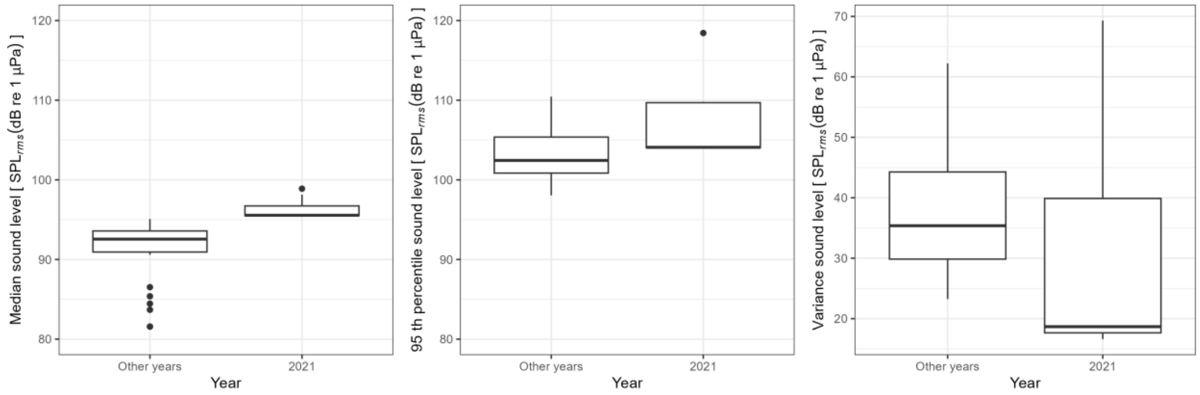

Figure S3. Distribution of the median, 95th percentile and variance of sound levels in the low-frequency band (50 Hz – 1 kHz), measured at the hydrophone deployed at the Marine Reserve location and summarised over the 24-hr window prior to sampling. Sound levels associated with faecal samples collected in June and July 2021 are compared to sound levels associated with samples collected in the same months in other years. The comparison shows that the median and 95th percentile low-frequency sound levels were higher in 2021 while the seismic survey was carried out along the Oregon coast.

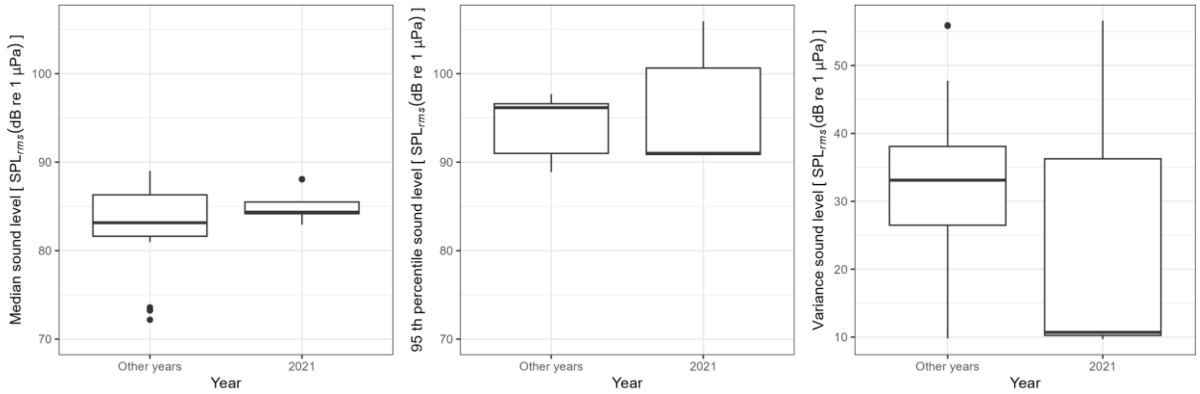

Figure S4. Distribution of the median, 95th percentile and variance of sound levels in the high-frequency band (1 – 4 kHz), measured at the hydrophone deployed at the Marine Reserve location and summarised over the 24-hr window prior to sampling. Sound levels associated with faecal samples collected in June and July 2021 are compared to sound levels associated with samples collected in the same months in other years. The boxplots highlight that the median and 95th percentile sound levels in the high-frequency band during the 2021 seismic survey were comparable to other years.

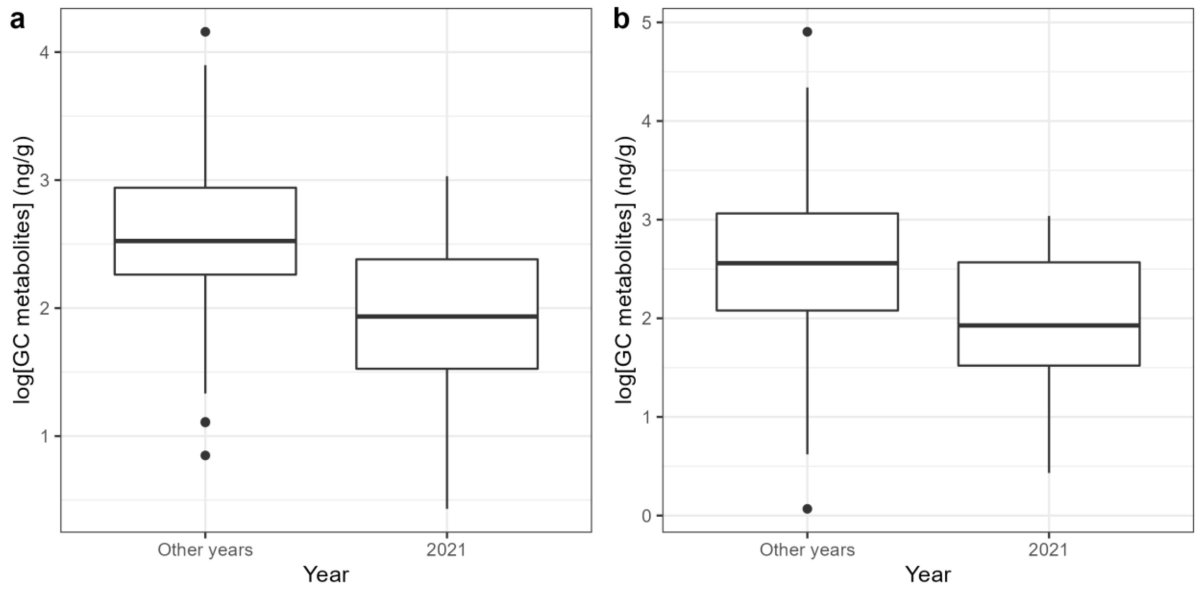

Figure S5. Distribution of log-transformed glucocorticoid (GC) concentrations in gray whale faecal samples collected in a) June-July 2021 (while a seismic survey was conducted along the Oregon coast) and b) the entire field season, compared to the same periods in other years. The boxplot shows that GC concentrations were lower in 2021 during the months of the seismic survey (a), but there was overlap between the distributions and this lower level was also visible outside the period when the survey took place (b).

Binary analyses

In all analyses presented in the main text, we treated the response variable (i.e., the concentration of GC metabolites in faecal samples) as continuous and used a Gamma distribution and a logarithmic link function to model the relationships with the covariates of interest. Here, we explore the alternative assumption that grey whale physiology switches between two possible states, i.e., that there exist a baseline state and a hypothetical response state, characterised by different GC hormone profiles. We used two possible thresholds of GC concentration to define the response state: the 75th and the 95th percentiles of GC concentrations across all samples (Fig. S6). The generalised additive modelling framework was modified to accommodate this binary response, assuming a binomial distribution of the residuals and a logit link function. The observation model was also adjusted; specifically, we introduced a binary misclassification state:

$$m \sim Bernoulli(p)$$

which took value of 1 if the measurement error for GC concentration led to the wrong assignment of the sample with respect to the threshold of response. Therefore, p was the misclassification probability and had a normal prior (truncated at 0 and 1), centred on the mean proportion of repeat samples that were assigned to contrasting states across repeats, and with precision 50 (i.e., standard deviation = 0.14).

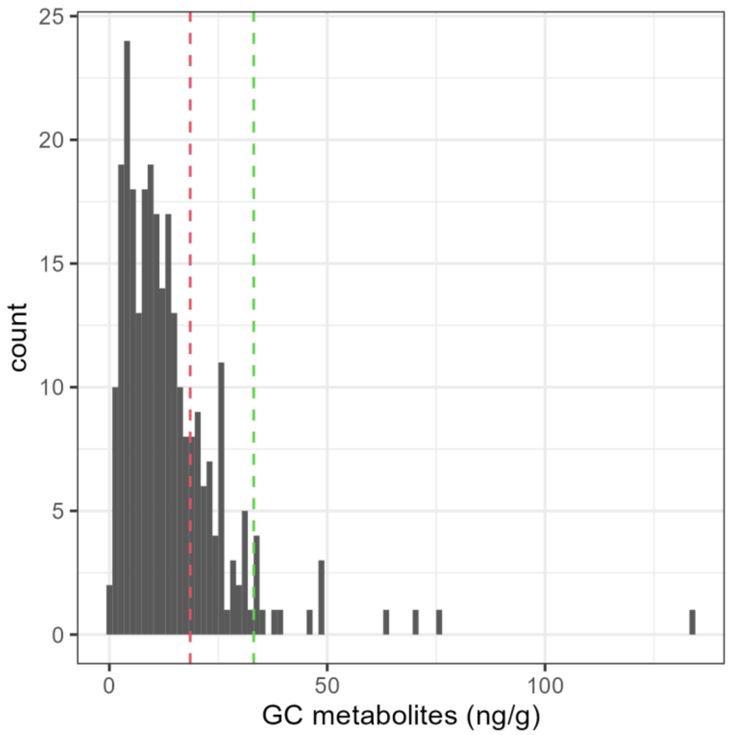

Figure S6. Distribution of GC metabolite concentrations across gray whale faecal samples, and thresholds of response tested within binary analyses (75th percentile in red, and 95th percentile in green).

We tested this alternative formulation for the two possible thresholds, focusing on the model that included the interaction between sex and the 95th percentile of sound levels in the high-frequency band (1– 4 kHz) between 5 AM and 6 PM in the day prior to sampling. In both models, the estimated relationships with the contextual variables were comparable to those described for the continuous response in the main text, albeit with larger credible intervals (CI; Fig. S7 and S8). In the model using the 75th percentile of GC concentrations as the threshold, there was a positive trend between sound levels and the probability of being in a response state for males, and a negative trend for females (Fig. S7), similar to the results presented in the main text. However, both curves included the 0 line and had EDF < 1. These trends disappeared and became even more variable when using the 95th percentile of GC concentrations as the threshold (Fig. S8), but it should be noted that only 14 samples were classified as responses in this case. Overall, the alternative binary formulation did not improve the precision of the results. Given these results, and the requirement to choose an arbitrary threshold of response under this formulation, we decided not to progress this approach further.

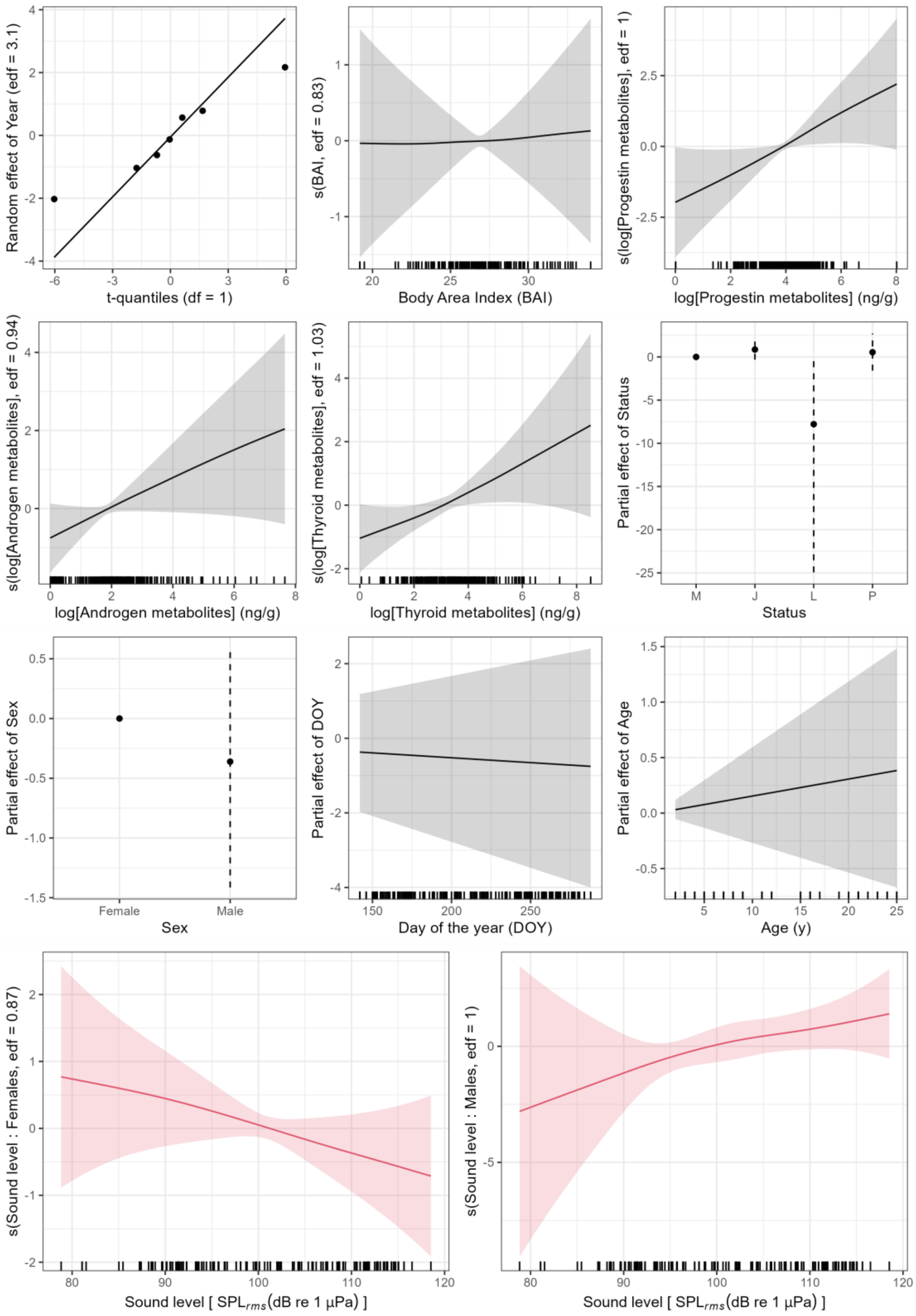

Figure S7. Results of the binary model using the 75th percentile of GC concentrations in gray whale faecal samples as the threshold of response and including the interaction between sex and the 95th percentile of sound levels in the high frequency band (1 – 4 kHz) between 5 AM and 6 PM in the day prior to sampling. Each panel reports the posterior effect of each contextual (black) and stressor covariate (red) in the model, with the shaded areas representing the 95% credible intervals. For smooth relationships, the label of the y-axis also reports the effective degrees of freedom (edf) of the spline.

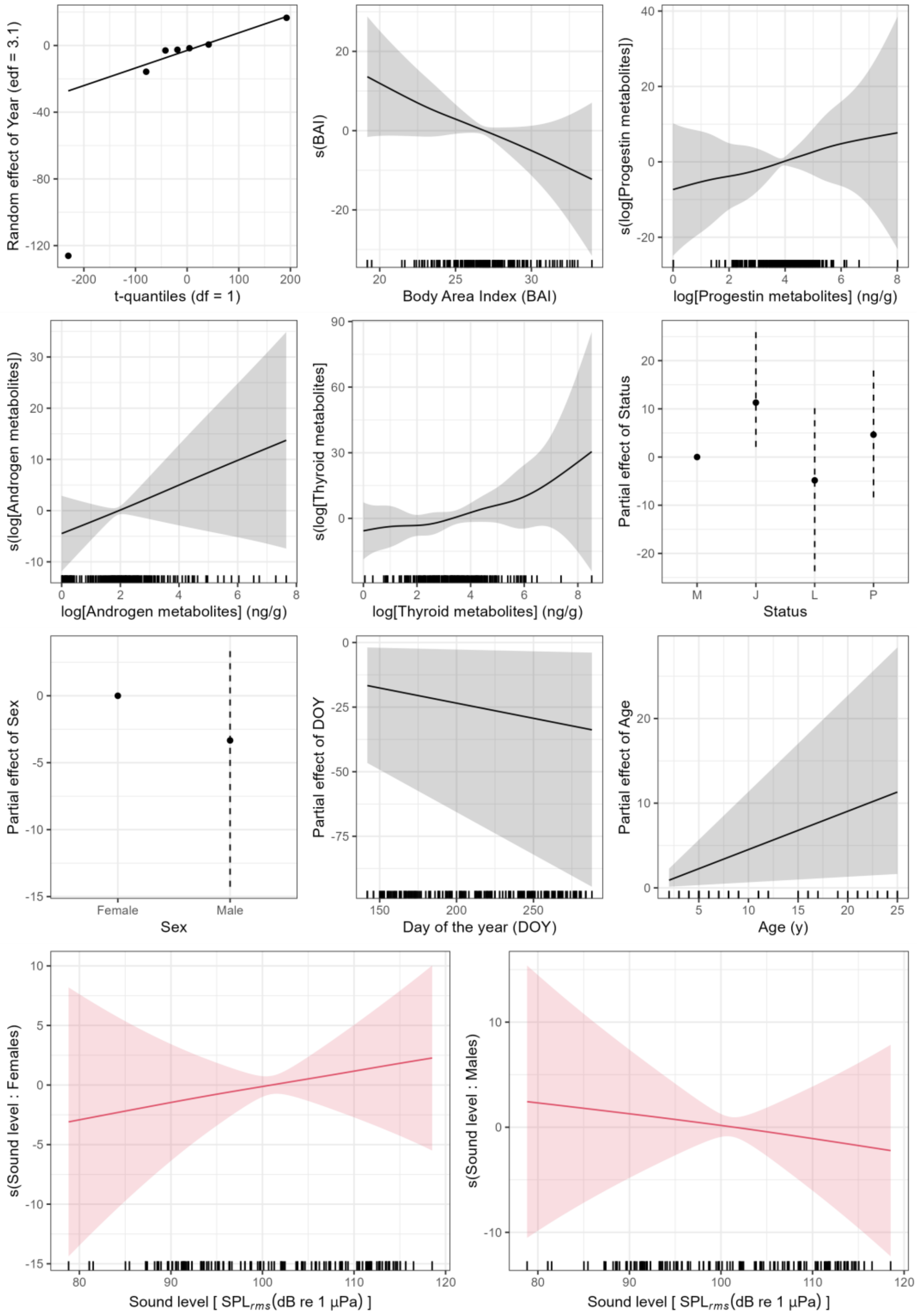

Figure S8. Results of the binary model using the 95th percentile of GC concentrations in gray whale faecal samples as the threshold of response and including the interaction between sex and the 95th percentile of sound levels in the high frequency band (1 – 4 kHz) between 5 AM and 6 PM in the day prior to sampling. Each panel reports the posterior effect of each contextual (black) and stressor covariate (red) in the model, with the shaded areas representing the 95% credible intervals. The effective degrees of freedom of the spline relationships could not be calculated, likely due to the small number of samples classified as responses.

Analysis using a different age of sexual maturity

For all analyses reported in the main text, we used an age of 8 years to classify an individual as mature (corresponding to the mean age at maturity reported by Rice and Wolman, 1971). Here, we explored the influence of this assumption on the results, using 12 years as the age of maturity, in alternative (corresponding to the maximum age at maturity in Rice and Wolman, (1971). Individuals with known or minimum age <12 years were considered mature if at least 50% of the posterior predictive distribution of their total length estimated from photogrammetry was greater than the mean length at maturity for gray whales (11.1 m for males and 11.7 m for females and individuals with an unknown sex; Rice and Wolman, 1971).

We tested this influence on the results of the model that included the interaction between sex and the 95th percentile of sound levels in the high frequency band (1– 4 kHz) between 5 AM and 6 PM in the day prior to sampling. Fig. S9 shows that all estimated relationships between covariates and GC concentrations remained largely unchanged, with the exception of the effect of the categorical variable for status, where the credible interval for the effect of being a juvenile included 0. This result suggests that the effect of juvenile status on GC concentrations should be interpreted with caution, as it is somewhat dependent on what criterion is used to classify an animal as a juvenile.

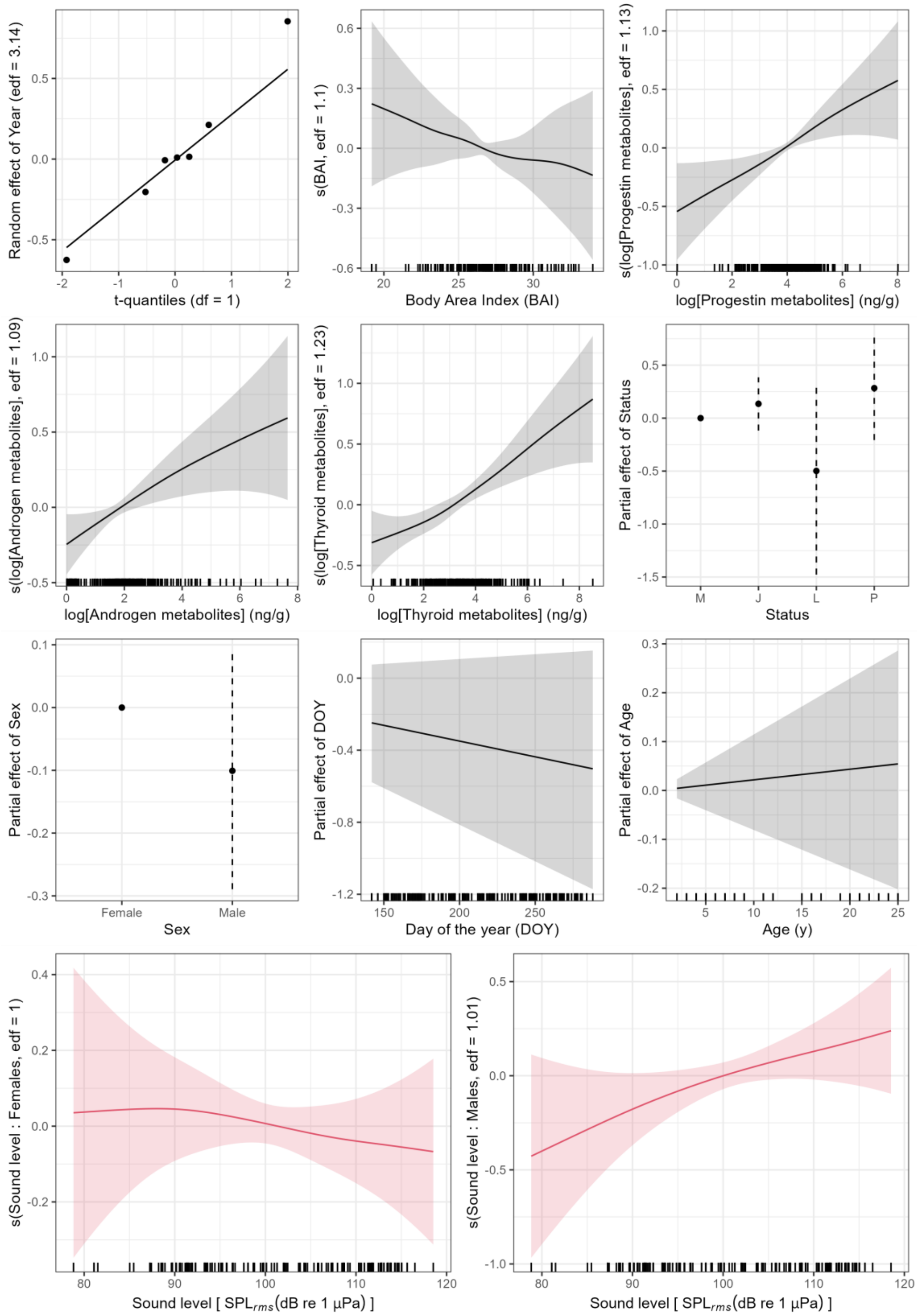

Figure S9. Results of the model using 12 years as the age of maturity and including the interaction between sex and the 95th percentile of sound levels in the high frequency band (1 – 4 kHz) between 5 AM and 6 PM in the day prior to sampling. Each panel reports the posterior effect of each contextual (black) and stressor covariate (red) in the model, with the shaded areas representing the 95% credible intervals. For smooth relationships, the label of the y-axis also reports the effective degrees of freedom (edf) of the spline.

Analysis excluding the data from Port Orford

There were 16 faecal samples collected outside our main study area (around Port Orford, OR). These samples came from whales that were sighted and sampled on other occasions in our study area, and therefore they were retained in the analysis (although with all the stressor variables set to missing). Here, we assessed the effect of excluding these samples on the results of the model that included the interaction between sex and the 95th percentile of sound levels in the high frequency band (1 – 4 kHz) between 5 AM and 6 PM in the day prior to sampling. Fig. S10 shows that all estimated relationships between covariates and GC concentrations remained largely unchanged when these data points were excluded.

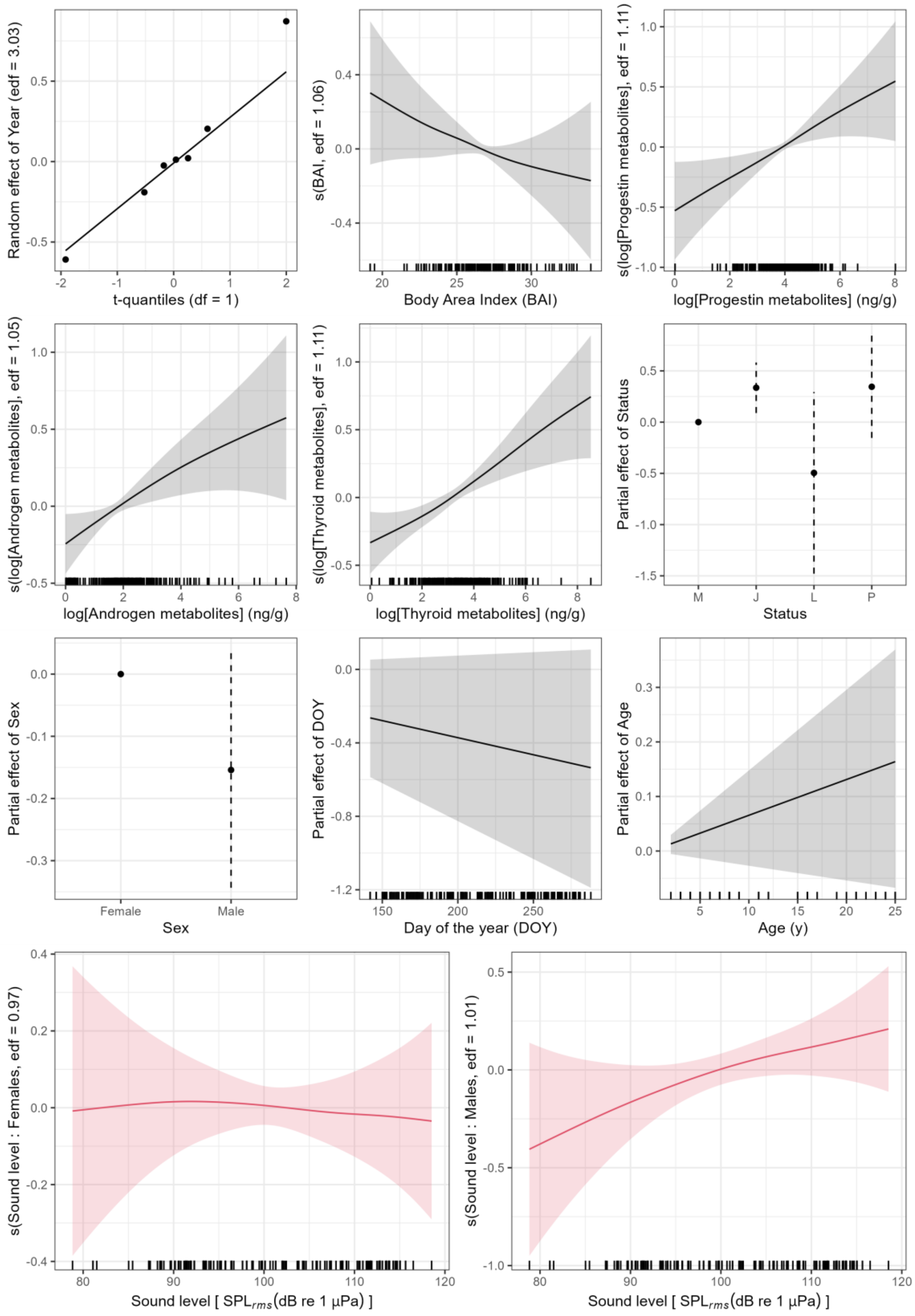

Figure S10. Results of the model using 12 years as the age of maturity and including the interaction between sex and the 95th percentile of sound levels in the high frequency band (1 – 4 kHz) between 5 AM and 6 PM in the day prior to sampling. Each panel reports the posterior effect of each contextual (black) and stressor covariate (red) in the model, with the shaded areas representing the 95% credible intervals. For smooth relationships, the label of the y-axis also reports the effective degrees of freedom (edf) of the spline.

Analysis incorporating the uncertainty in BAI estimates

The Body Area Index (BAI) is a unitless metric that is generated from aerial imagery using photogrammetric techniques. In our study, we used the Bayesian approach described in Bierlich *et al.* (2021) to analyse these data and, therefore, all BAI mean estimates had an associated standard deviation. In all models in the main text, we did not incorporate such uncertainty. Here, we tested its influence on the results. Specifically, we included an additional observation model, whereby the true BAI value for an individual was drawn from a normal distribution, centred on the posterior mean and with the posterior standard deviation. We investigated the influence of this modification on the results of the model that included the interaction between sex and the 95th percentile of sound levels in the high frequency band (1 – 4 kHz) between 5 AM and 6 PM in the day prior to sampling. All the estimated relationships between covariates and GC concentrations were not affected by uncertainty in BAI. Specifically, Fig. S11 shows that the estimated relationship between BAI and GC concentrations was unchanged under this model.

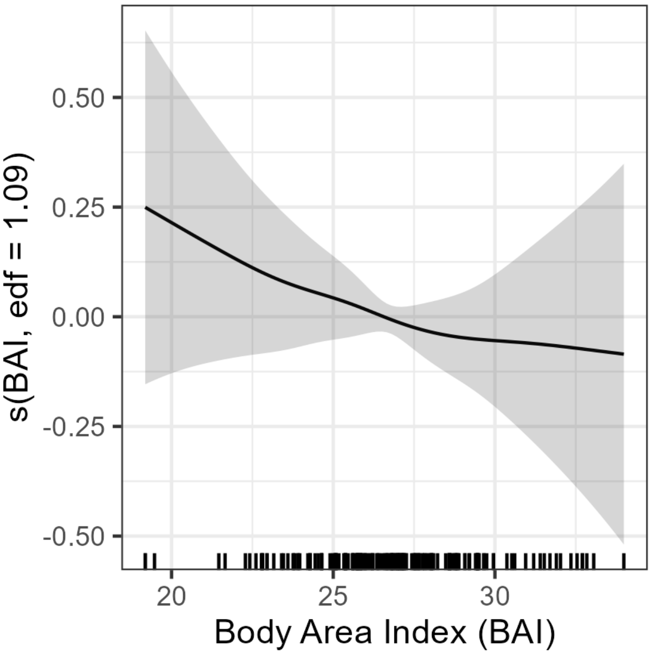

Figure S11. Estimated effect of Body Area Index (BAI) on GC concentrations in gray whale faecal samples when the model included uncertainty in BAI estimates. The shaded area represents the 95% credible interval. The label of the y-axis also reports the effective degrees of freedom (edf) of the spline.

Using a different criterion to compute gray whale sound level exposure

In the analyses presented in the main text, we associated each faecal sample with the sound levels measured at the closest hydrophone (either at the Marine Reserve or the Port of Newport location). However, there is uncertainty as to whether those sound levels accurately capture the soundscape to which individuals were exposed during the temporal windows of interest (24 hr, 48 hr, or between 5 AM and 6 PM in the day prior to sampling). This uncertainty emerges in part from the potential movement of the animals over those periods. In part, it also results from how representative the two hydrophone locations were of the soundscape in different portions of the study area.

In summer 2016, a free drifting hydrophone system was deployed to collect 40 spatially varied, short-term recordings (15-90 minutes duration). The drifting hydrophone system, from the surface to 6 m depth, consists of a spar buoy, compliant line, a heave plate, and the hydrophone sensor that effectively mitigates surface wave motion, thereby avoiding excessive low frequency data contamination from flow noise. These recordings were used to characterize and quantify the spatial variation in ambient sound levels throughout the study area. Spatial interpolation of these data showed that the Marine Reserve hydrophone was located in a comparatively quieter portion of the study area (Fig. S12). Specifically, sound levels tended to be higher and comparable in proximity of the two main harbours in the area (Newport and Depoe Bay), while they were lower in the stretch of coast between Yaquina Head (44.6768° N, 124.0795° W) and Cape Foulweather (44.7723° N, 124.0759° W), which contained the Otter Rock Marine Reserve. Therefore, the sound levels measured at the Port of Newport hydrophone may provide a better representation of the soundscape around Depoe Bay in the north than the recordings at the Marine Reserve hydrophone (Haver *et al.*, 2023).

Thus, as an alternative assumption to using the closest hydrophone to each sampling location, we tested the effects of associating sound levels to each faecal sample based on the three soundscape regions described above: north of Cape Foulweather (Depoe Bay area), between Cape Foulweather and Yaquina Head (Marine Reserve area), and south Yaquina Head (Newport area); whereby the sound levels in the Newport area were assumed to be representative of the sound levels in the Depoe Bay area. In other words, if a faecal sample was collected within the Depoe Bay area, it was associated with the sound metrics collected at the Port of Newport hydrophone.

The analysis was then repeated for a set of key models discussed in the Results section of the main text, including the 95th and variance of sound levels in the two frequency bands, summarised over the 24-hr and 5 AM – 6 PM windows, and interacting with the three factors of interest (sex, BAI and closest hydrophone). Across all models, the posterior relationships between sound levels and glucocorticoid concentrations were largely unchanged as a result of the different criterion of association (e.g., Fig. S13), suggesting that our results were robust to some uncertainty in these stressor variables.

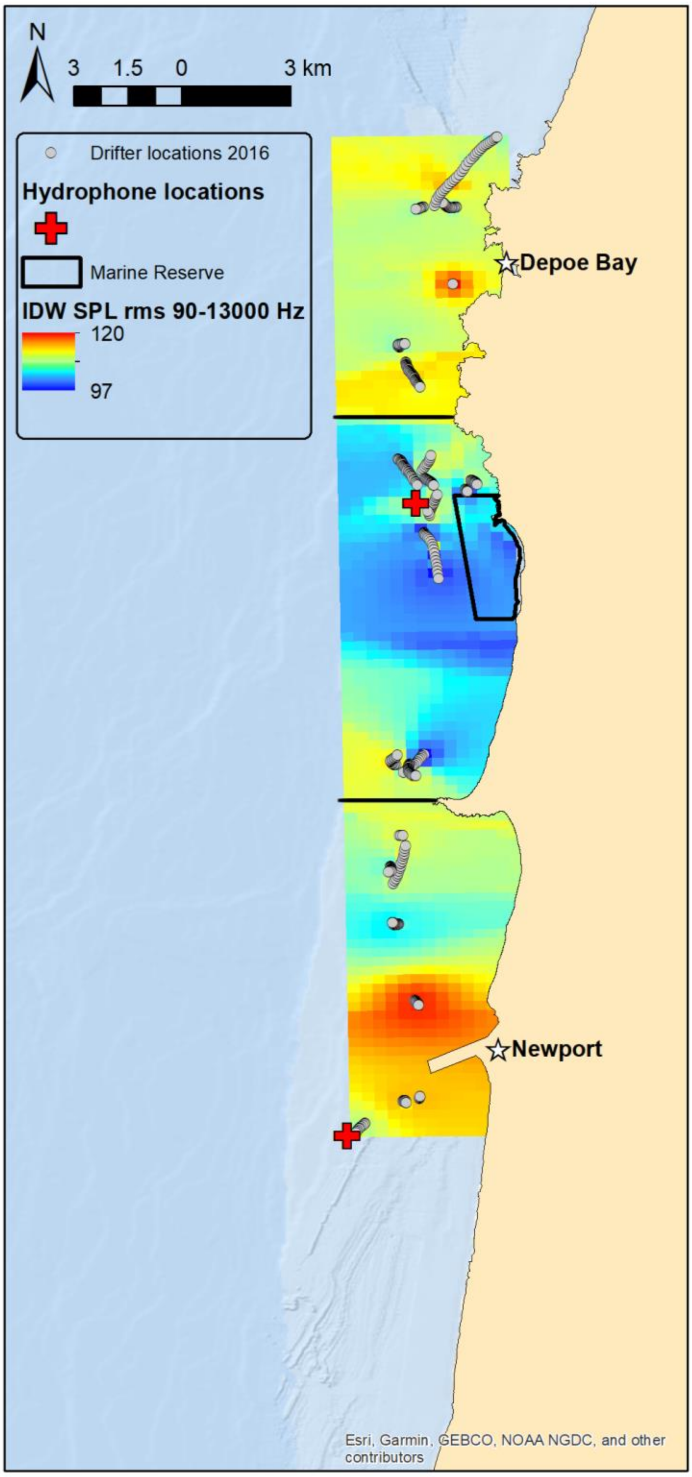

Figure S12. Map of the study area, with locations of the recordings using the drifting hydrophone system (in grey) and interpolated sound levels represented as an underlying, coloured surface (Inverse Distance Weighting [IDW] interpolation, with 5000 m search radius and 250 m cell size, of the median root-mean-square sound pressure levels [SPL_{rms}] at each drifter location in the frequency band 90 Hz – 13000 Hz). The map also reports the two main harbours in the area (Newport and Depoe Bay), the locations of the two fixed hydrophones (Port of Newport and Marine Reserve), and the boundaries of the three sound regions.

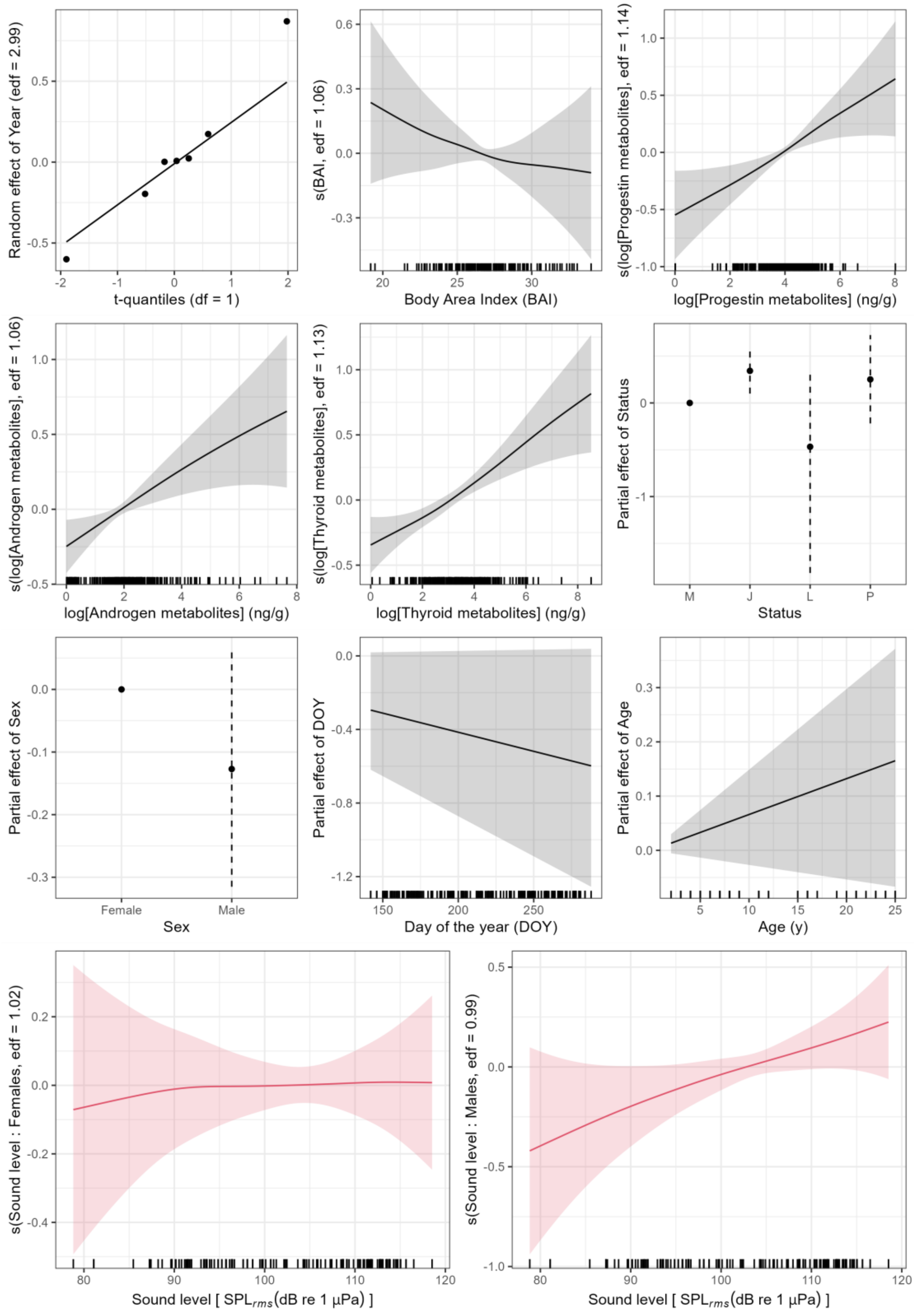

Figure S13. Results of the model using an alternative criterion for the association of sound levels to the gray whale faecal samples, and including the interaction between sex and the 95th percentile of sound levels in the high frequency band (1 – 4 kHz) between 5 AM and 6 PM in the day prior to sampling. Each panel reports the posterior effect of each contextual (black) and stressor covariate (red) in the model, with the shaded areas representing the 95% credible intervals. For smooth relationships, the label of the y-axis also reports the effective degrees of freedom (edf) of the spline.

Residual diagnostics for one model example

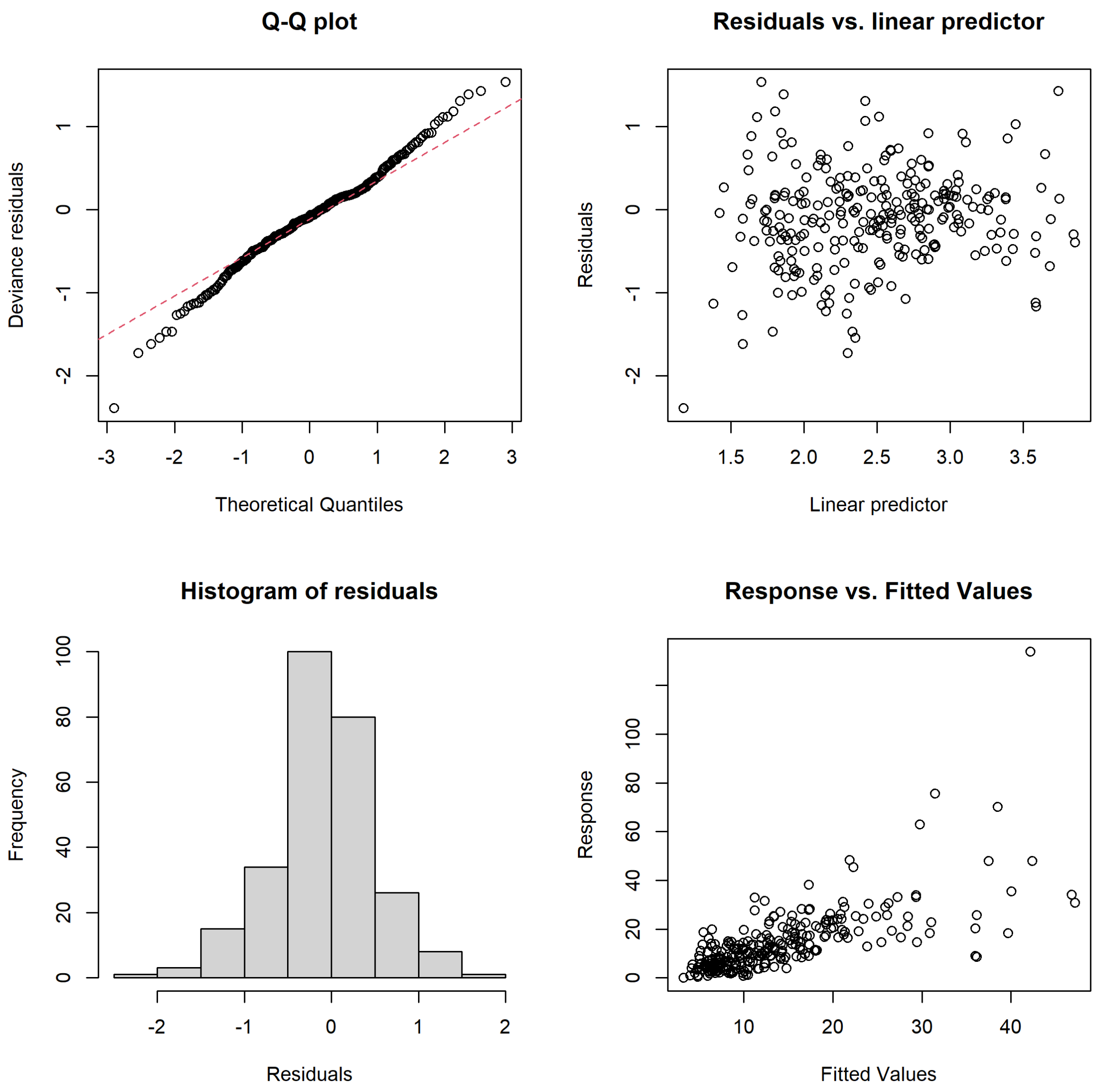

Figure S14. Residual diagnostic plots for the model including the interaction between sex and the 95th percentile of sound levels in the high frequency band (1 – 4 kHz) between 5 AM and 6 PM in the day prior to sampling (using deviance residuals). The quantile-quantile plot shows some deviation from theoretical quantiles for extreme GC concentrations in gray whale faecal samples, but otherwise the plots do not highlight any problematic residual pattern.

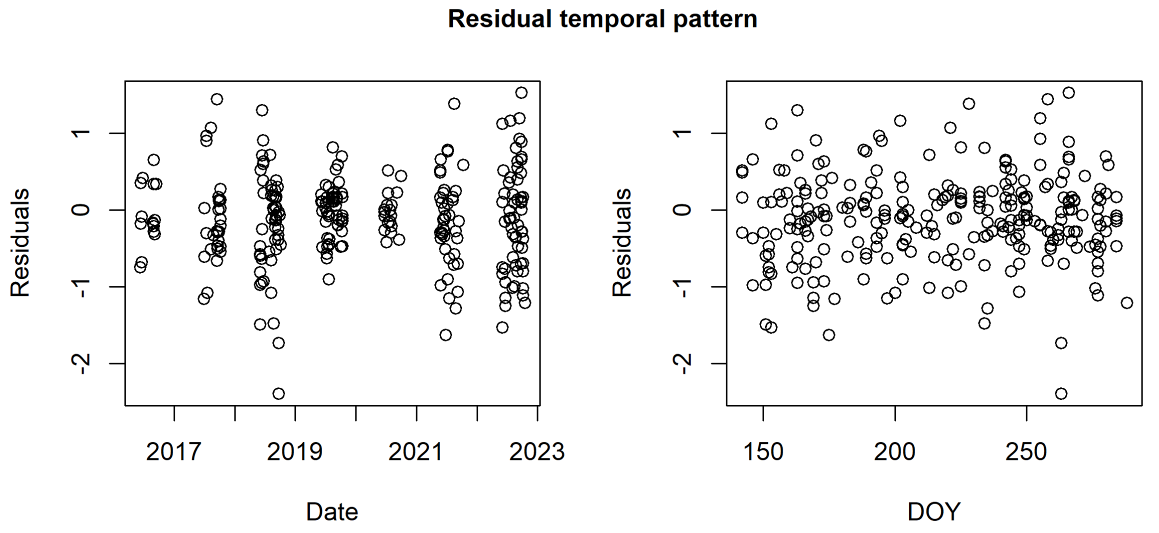

Figure S15. Residuals of the model including the interaction between sex and the 95th percentile of sound levels in the high frequency band (1 – 4 kHz) between 5 AM and 6 PM in the day prior to sampling (using deviance residuals), plotted against sampling date (left) and day of the year (DOY; right). The plots do not show any problematic residual temporal pattern.

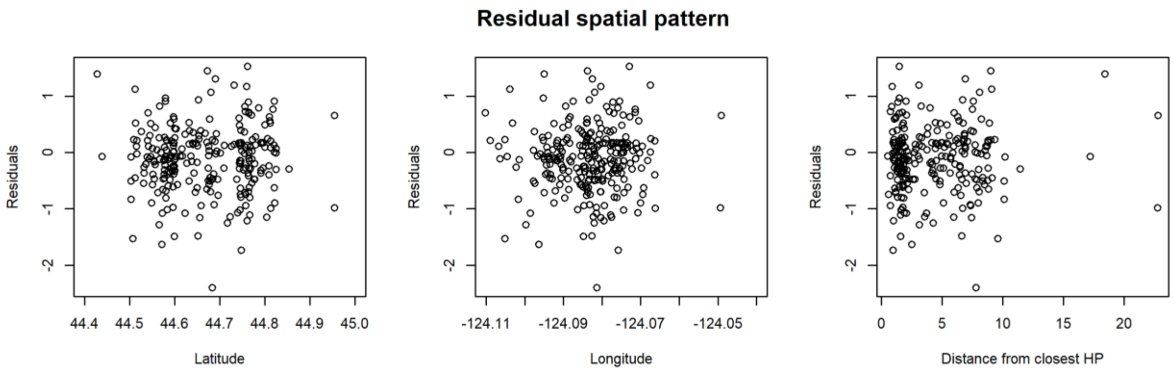

Figure S16. Residuals of the model including the interaction between sex and the 95th percentile of sound levels in the high frequency band (1 – 4 kHz) between 5 AM and 6 PM in the day prior to sampling (using deviance residuals), plotted against latitude of the sampling location (left), longitude (middle) and distance of the sampling location from the closest hydrophone (HP; right). The samples collected further south (around Port Orford, OR) were excluded from these plots to facilitate visualisation. The plots do not show any problematic residual spatial pattern.

Correlations among vessel counts, wind speed and sound levels

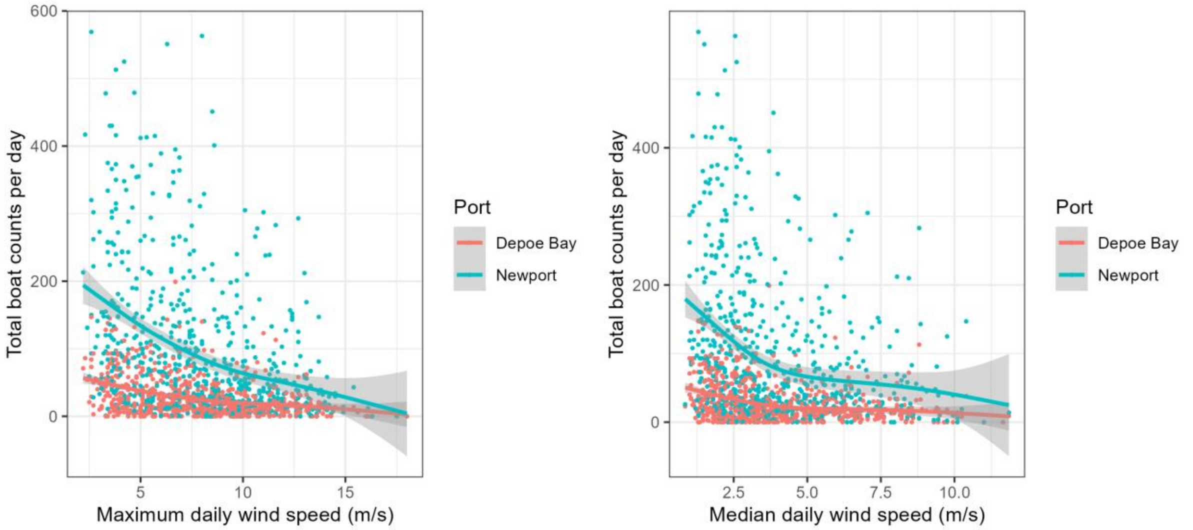

Figure S17. Total number of vessels counted per day in each of the two ports (Depoe Bay and Newport), plotted against the daily maximum and median wind speed measured at Newport harbour (data from an anemometer station located on South Beach, Newport, OR, USA; station NWPO3). The relationships are smoothed using cubic splines.

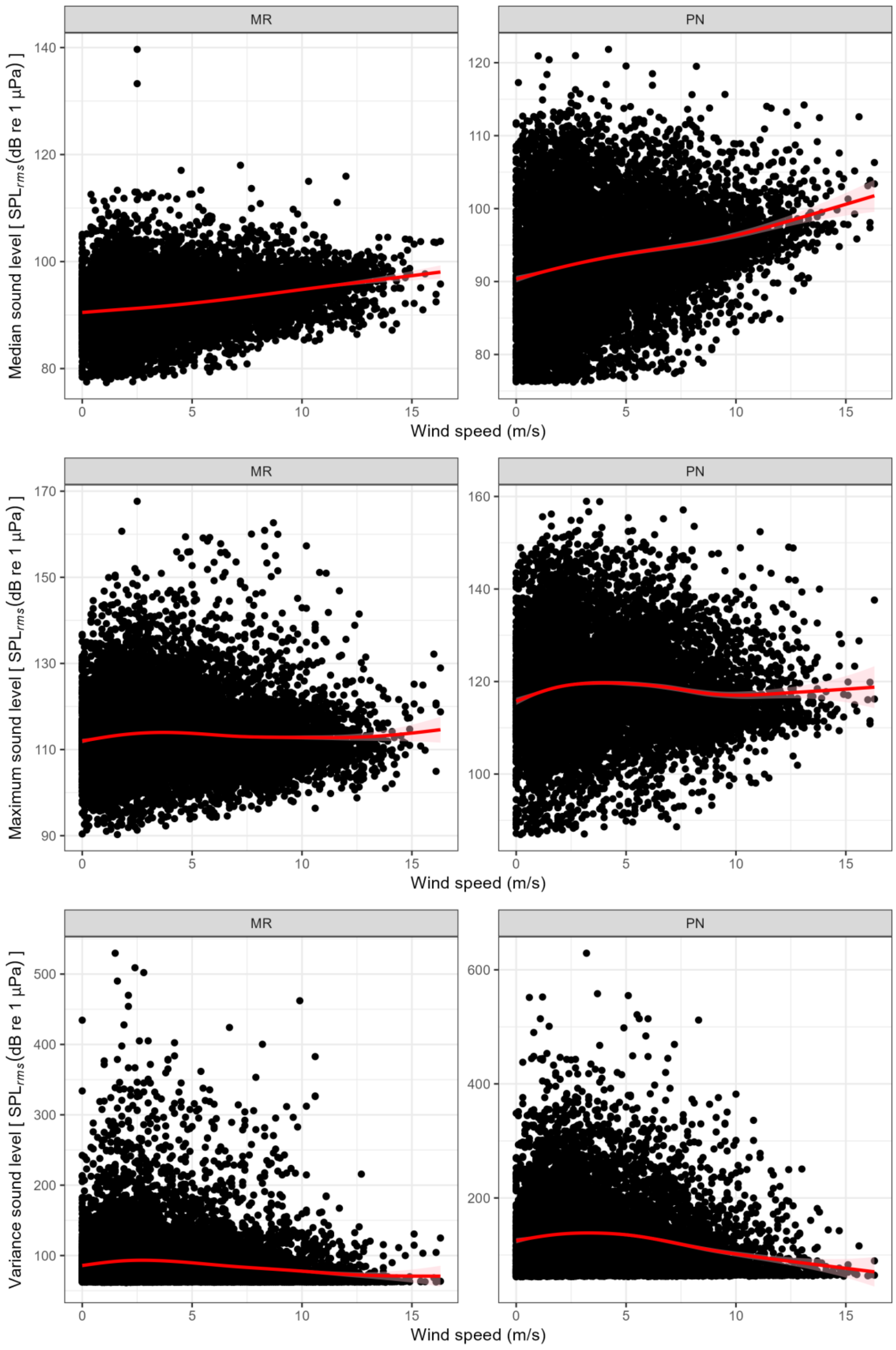

Figure S18. Hourly median, maximum and variance of sound levels in the low frequency band (50 Hz – 1 kHz) measured at each hydrophone location (MR = Marine Reserve; PN = Port of Newport), plotted against the hourly wind speed measured at Newport harbour. The relationships are smoothed using cubic splines.

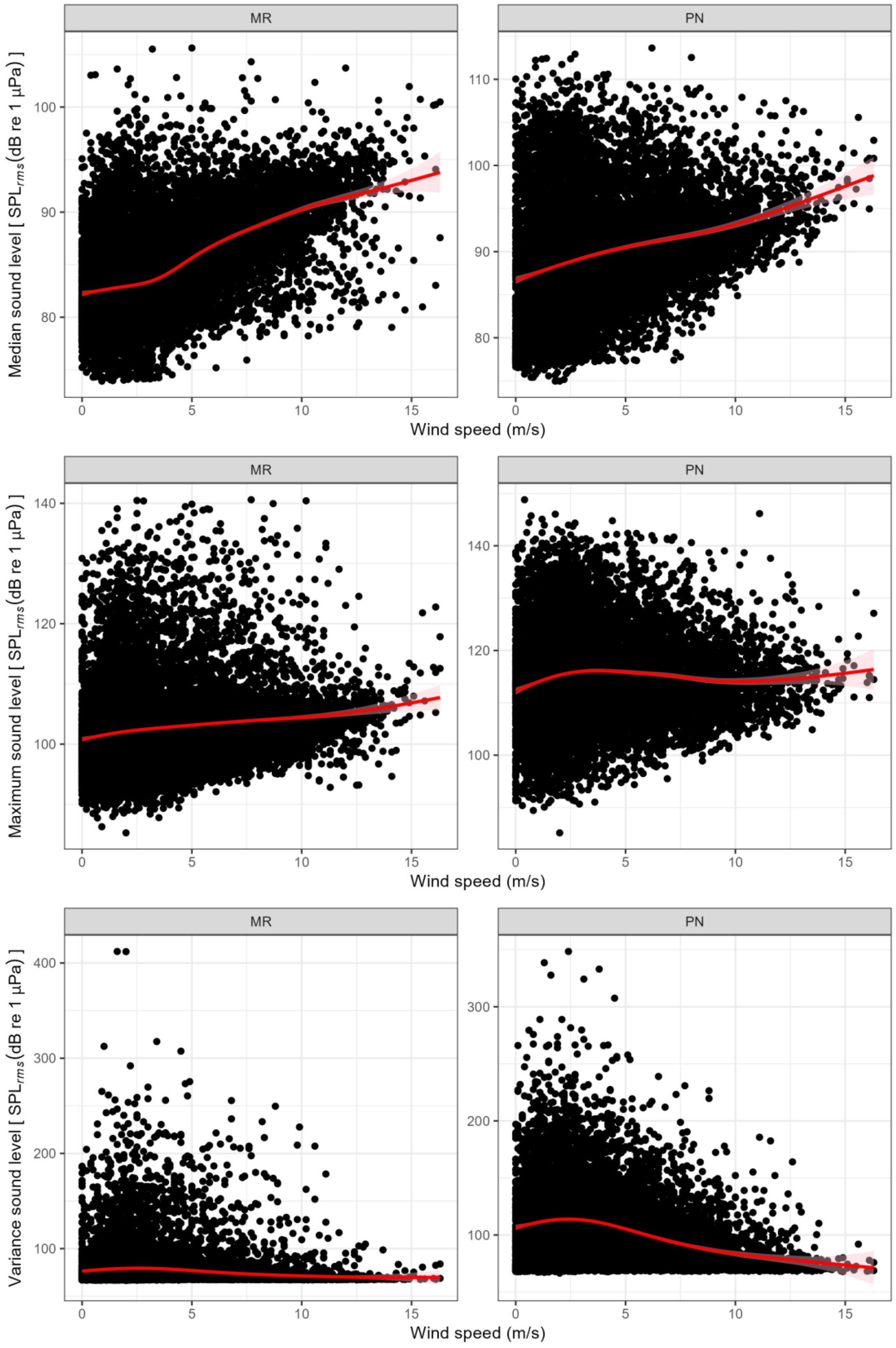

Figure S19. Hourly median, maximum and variance of sound levels in the high frequency band (1 – 4 kHz) measured at each hydrophone location (MR = Marine Reserve; PN = Port of Newport), plotted against the hourly wind speed measured at Newport harbour. The relationships are smoothed using cubic splines.

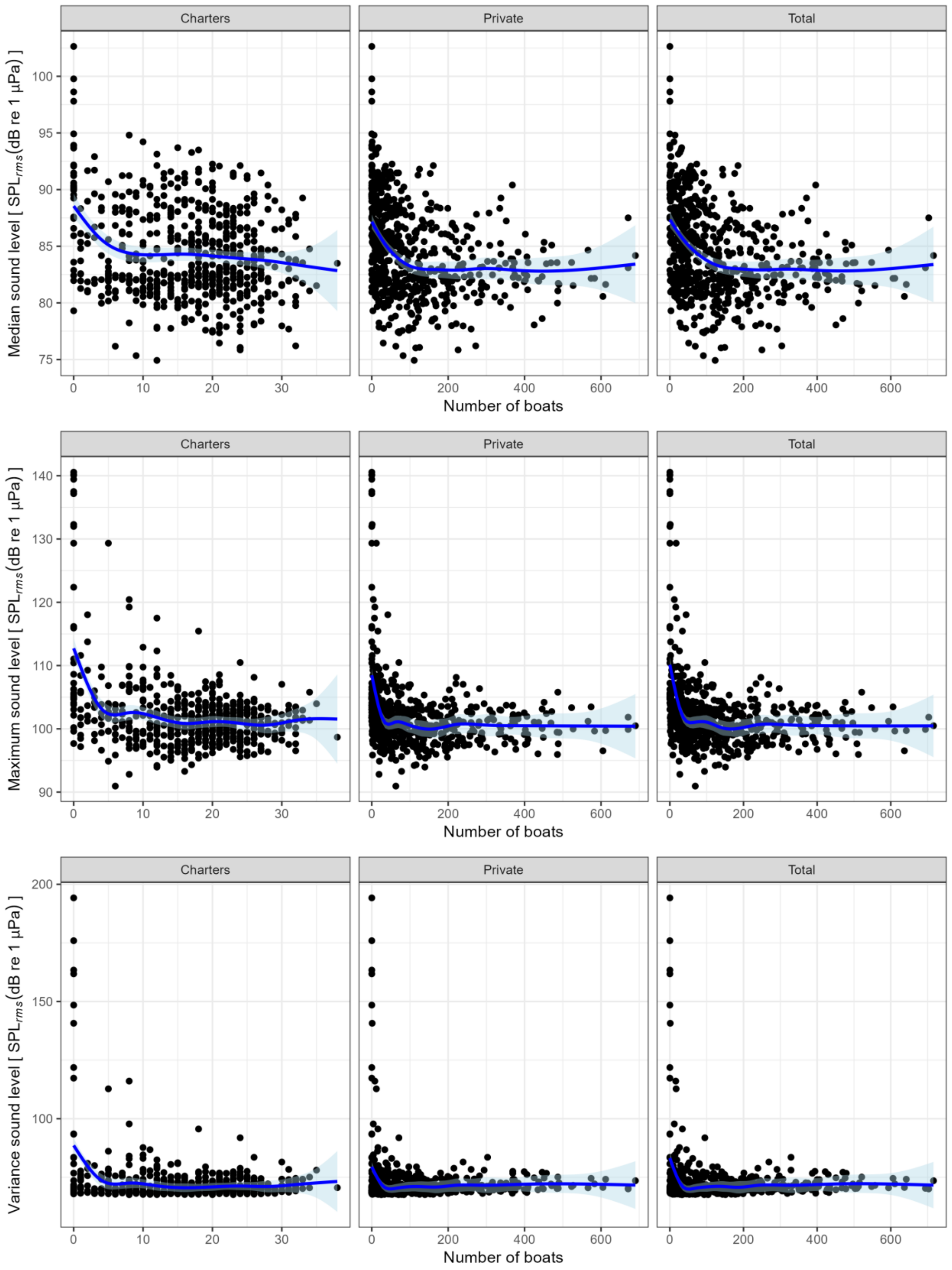

Figure S20. Daily median, maximum and variance of sound levels in the high frequency band (1 – 4 kHz) measured at the Marine Reserve hydrophone location, plotted against the count of charter, private and total vessels (summed across the two harbours). The relationships are smoothed using cubic splines.

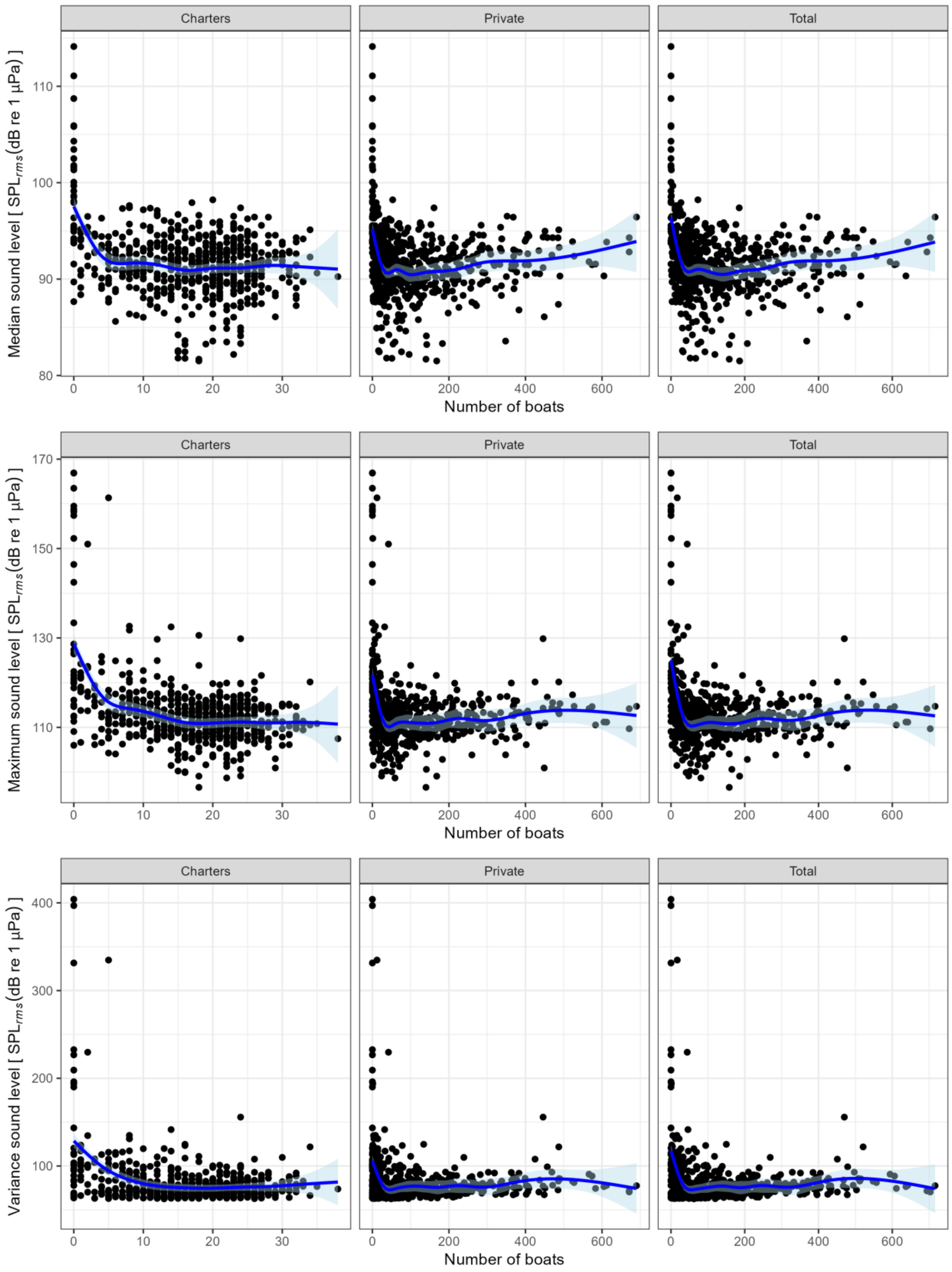

Figure S21. Daily median, maximum and variance of sound levels in the low frequency band (50 Hz – 1 kHz) measured at the Marine Reserve hydrophone location, plotted against the count of charter, private and total vessels (summed across the two harbours). The relationships are smoothed using cubic splines.

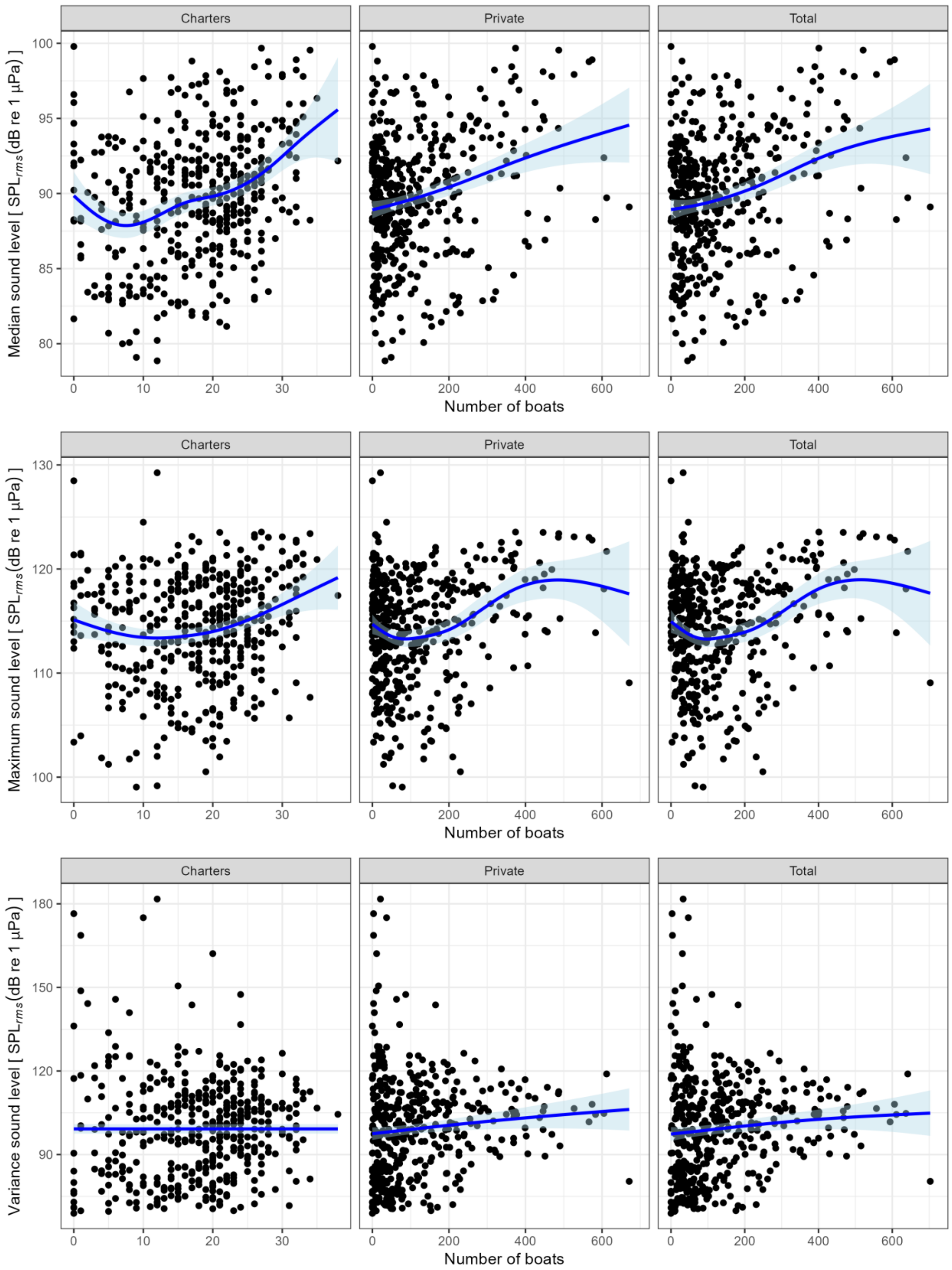

Figure S22. Daily median, maximum and variance of sound levels in the high frequency band (1 – 4 kHz) measured at the Port of Newport hydrophone location, plotted against the count of charter, private and total vessels (summed across the two harbours). The relationships are smoothed using cubic splines.

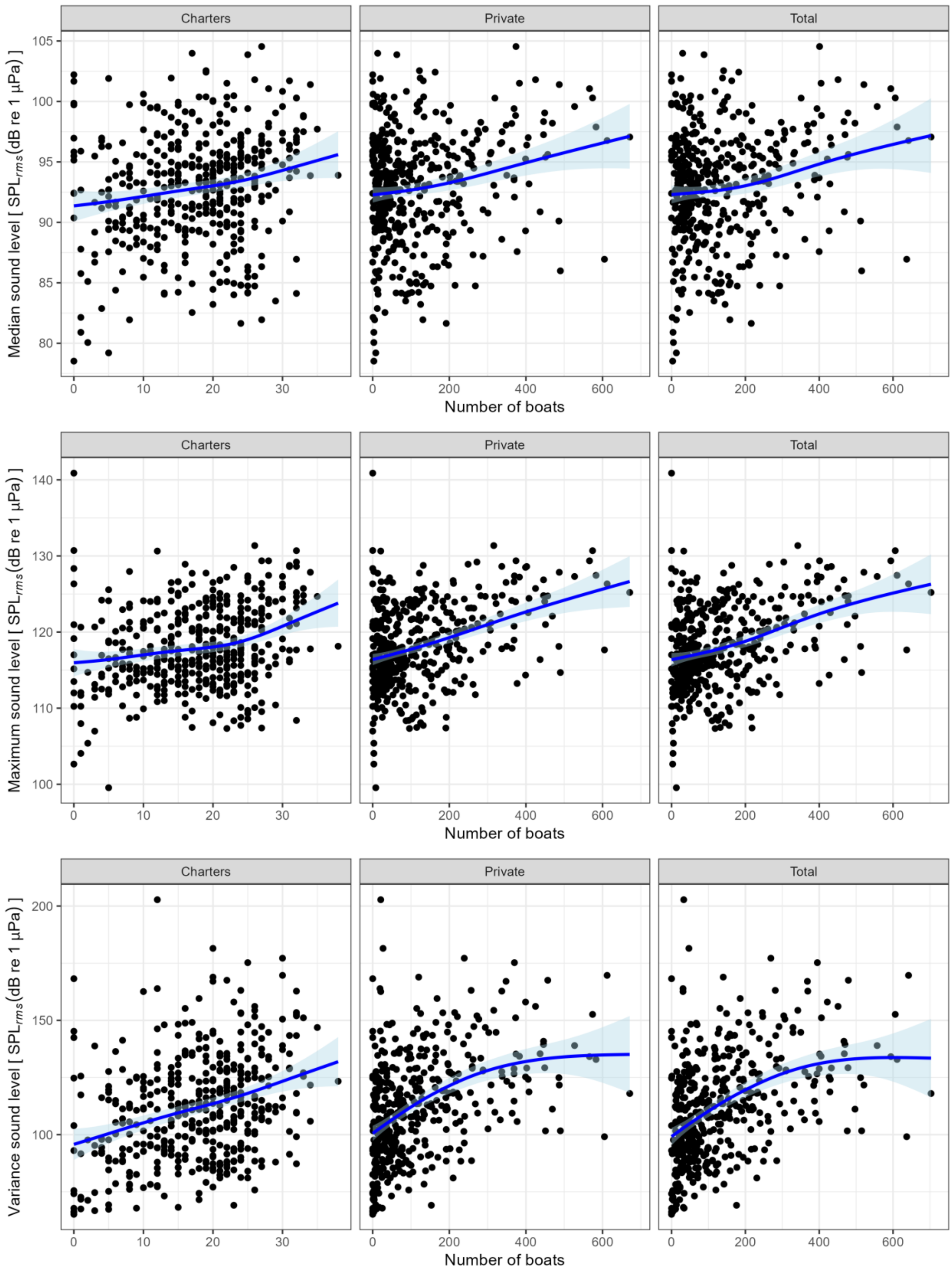

Figure S23. Daily median, maximum and variance of sound levels in the low frequency band (50 Hz – 1 kHz) measured at the Port of Newport hydrophone location, plotted against the count of charter, private and total vessels (summed across the two harbours). The relationships are smoothed using cubic splines.

Complete model results

The following plots report the estimated relationships of glucocorticoid concentrations with contextual and stressor variables across all models. In all plots, each panel reports the posterior effect of a contextual (black) or a stressor covariate (red) in the model, with the shaded areas representing the 95% credible intervals. For smooth relationships, the label of the y-axis also reports the effective degrees of freedom (edf) of the spline. Plot titles specify the stressor metric included in the model, for sound levels and vessel counts.

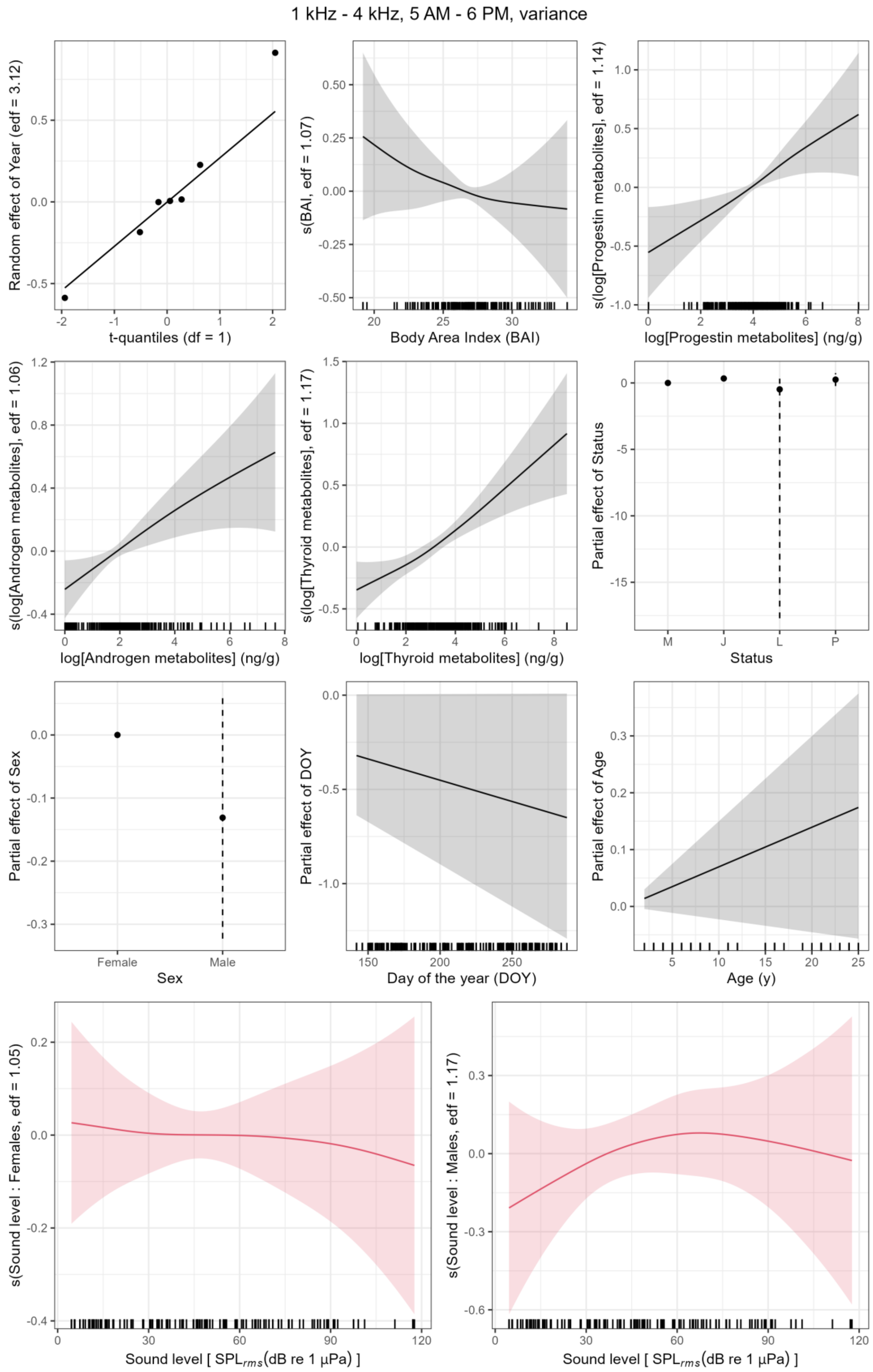

1 kHz - 4 kHz, 5 AM - 6 PM, variance
Random effect of Year (edf = 3.12)
t-quantiles (df = 1)
s(BAI, edf = 1.07)
Body Area Index (BAI)
s(log[Progestin metabolites], edf = 1.14)
log[Progestin metabolites] (ng/g)
s(log[Androgen metabolites], edf = 1.06)
log[Androgen metabolites] (ng/g)
s(log[Thyroid metabolites], edf = 1.17)
log[Thyroid metabolites] (ng/g)
Partial effect of Status
Status
M
J
L
P
Partial effect of Sex
Sex
Female
Male
Partial effect of DOY
Day of the year (DOY)
Partial effect of Age
Age (y)
s(Sound level : Females, edf = 1.05)
Sound level [SPL$_{rms}$(dB re 1 µPa)]
s(Sound level : Males, edf = 1.17)
Sound level [SPL$_{rms}$(dB re 1 µPa)]

1 kHz - 4 kHz, 5 AM - 6 PM, median

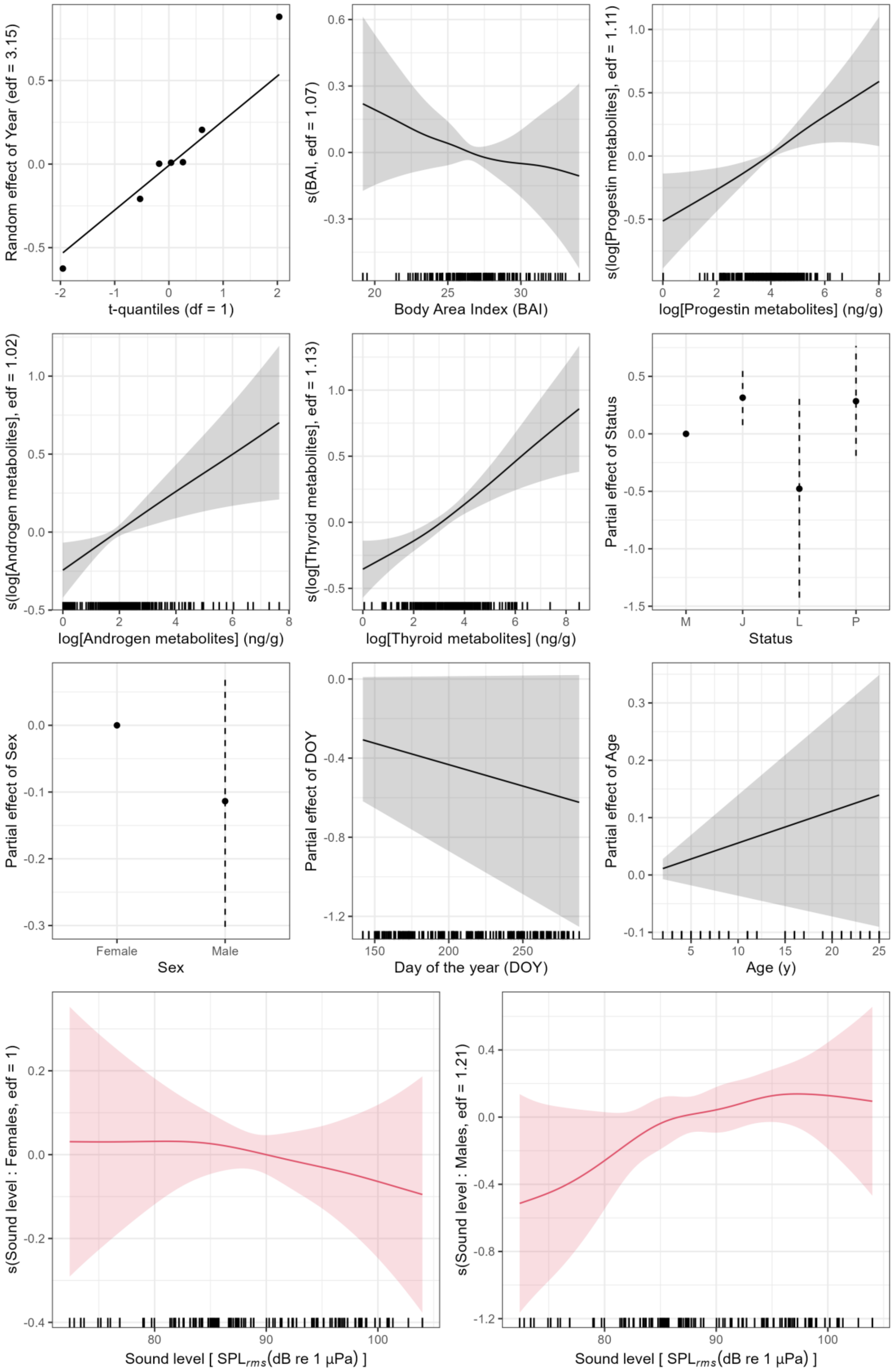

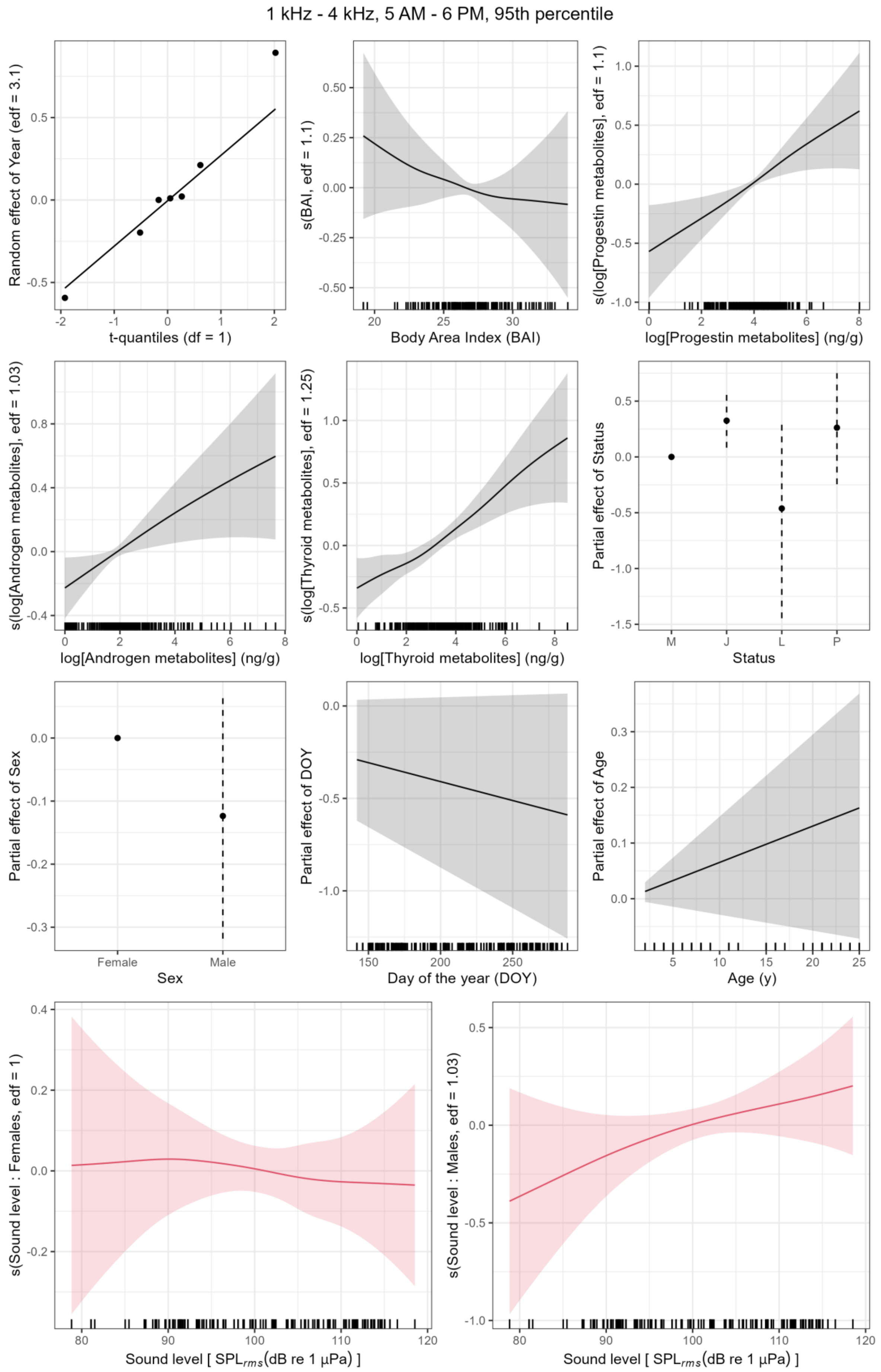
1 kHz - 4 kHz, 5 AM - 6 PM, 95th percentile
Random effect of Year (edf = 3.1)
t-quantiles (df = 1)
s(BAI, edf = 1.1)
Body Area Index (BAI)
s(log[Progestin metabolites], edf = 1.1)
log[Progestin metabolites] (ng/g)
s(log[Androgen metabolites], edf = 1.03)
log[Androgen metabolites] (ng/g)
s(log[Thyroid metabolites], edf = 1.25)
log[Thyroid metabolites] (ng/g)
Partial effect of Status
Status
M
J
L
P
Partial effect of Sex
Sex
Female
Male
Partial effect of DOY
Day of the year (DOY)
Partial effect of Age
Age (y)
s(Sound level : Females, edf = 1)
Sound level [SPL_{rms}(dB re 1 µPa)]
s(Sound level : Males, edf = 1.03)
Sound level [SPL_{rms}(dB re 1 µPa)]

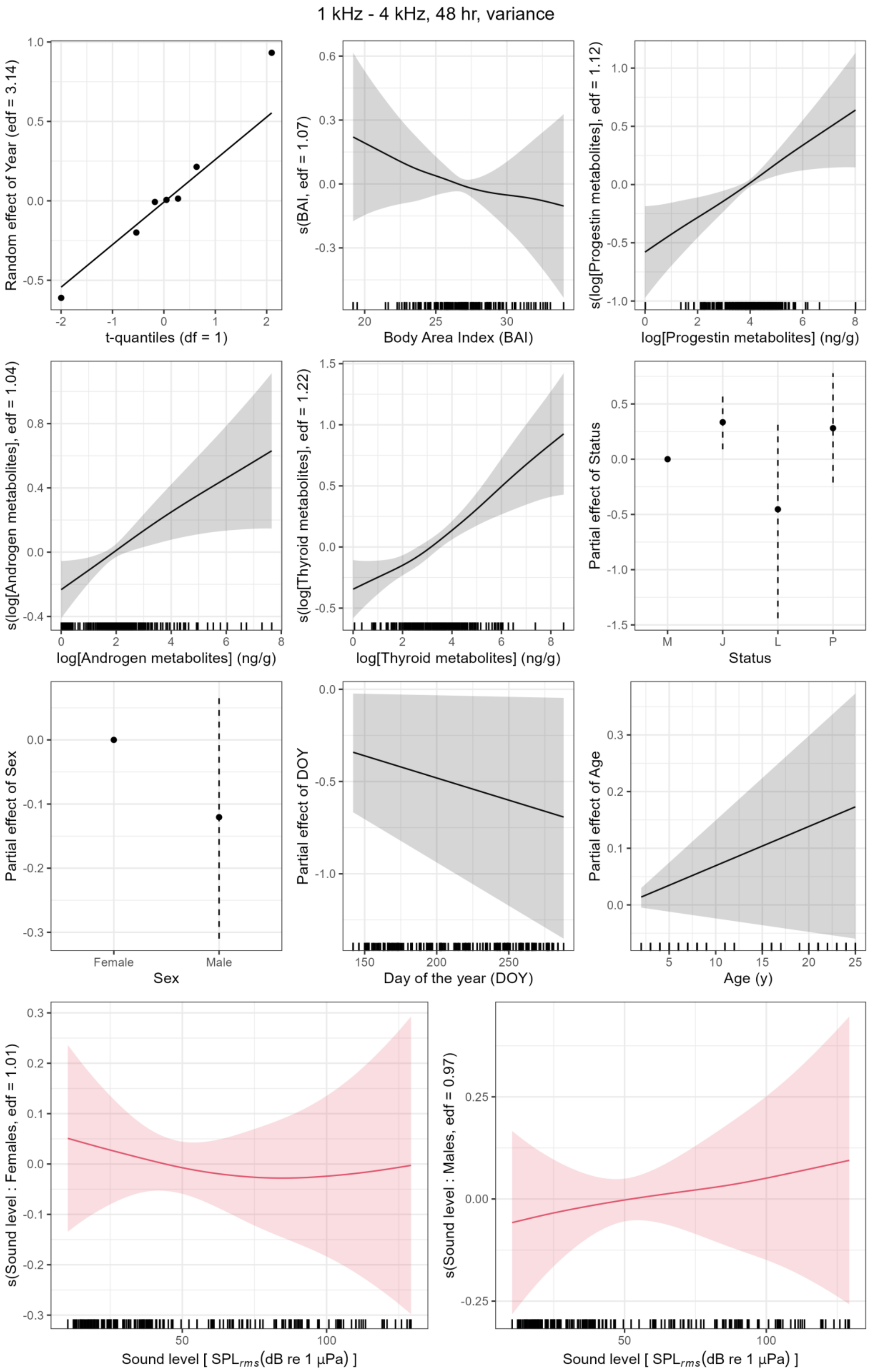

1 kHz - 4 kHz, 48 hr, variance
Random effect of Year (edf = 3.14)
t-quantiles (df = 1)
s(BAI, edf = 1.07)
Body Area Index (BAI)
s(log[Progestin metabolites], edf = 1.12)
log[Progestin metabolites] (ng/g)
s(log[Androgen metabolites], edf = 1.04)
log[Androgen metabolites] (ng/g)
s(log[Thyroid metabolites], edf = 1.22)
log[Thyroid metabolites] (ng/g)
Partial effect of Status
Status
M
J
L
P
Partial effect of Sex
Sex
Female
Male
Partial effect of DOY
Day of the year (DOY)
Partial effect of Age
Age (y)
s(Sound level : Females, edf = 1.01)
Sound level [SPLrms(dB re 1 µPa)]
s(Sound level : Males, edf = 0.97)
Sound level [SPLrms(dB re 1 µPa)]

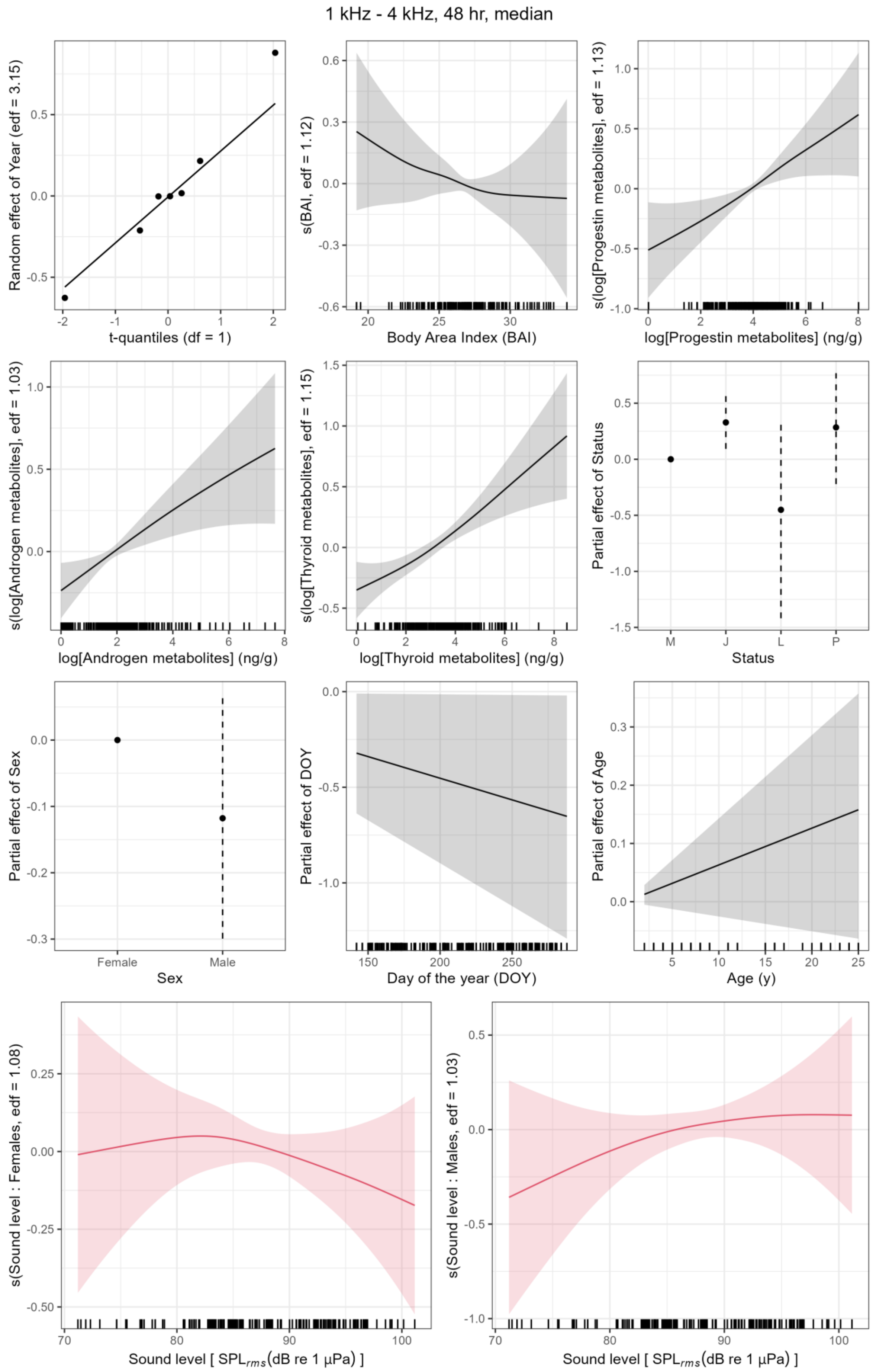

1 kHz - 4 kHz, 48 hr, median
Random effect of Year (edf = 3.15)
t-quantiles (df = 1)
s(BAI, edf = 1.12)
Body Area Index (BAI)
s(log[Progestin metabolites], edf = 1.13)
log[Progestin metabolites] (ng/g)
s(log[Androgen metabolites], edf = 1.03)
log[Androgen metabolites] (ng/g)
s(log[Thyroid metabolites], edf = 1.15)
log[Thyroid metabolites] (ng/g)
Partial effect of Status
Status
M
J
L
P
Partial effect of Sex
Sex
Female
Male
Partial effect of DOY
Day of the year (DOY)
Partial effect of Age
Age (y)
s(Sound level : Females, edf = 1.08)
Sound level [SPL_{rms}(dB re 1 μPa)]
s(Sound level : Males, edf = 1.03)
Sound level [SPL_{rms}(dB re 1 μPa)]

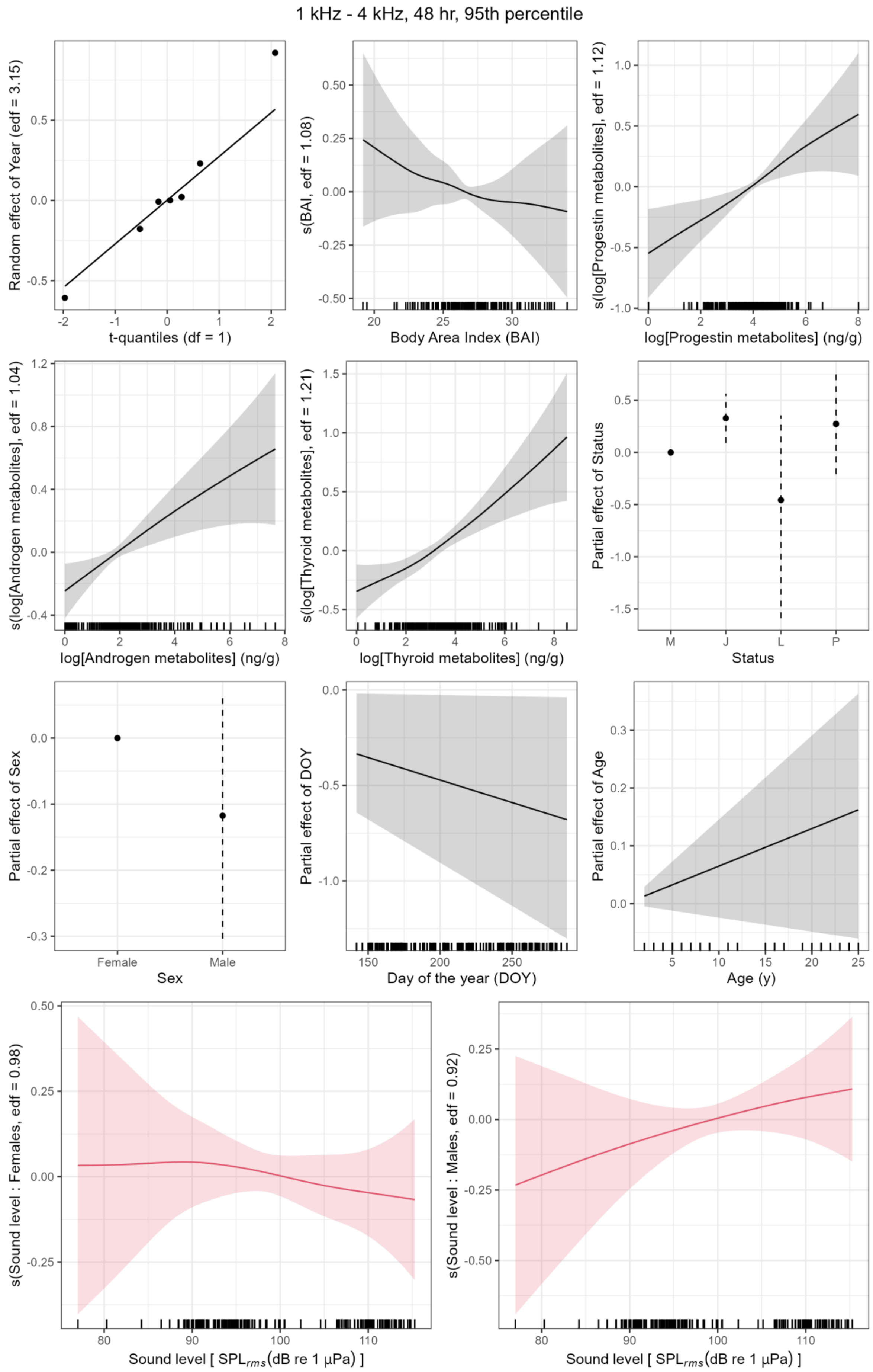
1 kHz - 4 kHz, 48 hr, 95th percentile
Random effect of Year (edf = 3.15)
t-quantiles (df = 1)
s(BAI, edf = 1.08)
Body Area Index (BAI)
s(log[Progestin metabolites], edf = 1.12)
log[Progestin metabolites] (ng/g)
s(log[Androgen metabolites], edf = 1.04)
log[Androgen metabolites] (ng/g)
s(log[Thyroid metabolites], edf = 1.21)
log[Thyroid metabolites] (ng/g)
Partial effect of Status
Status
M
J
L
P
Partial effect of Sex
Sex
Female
Male
Partial effect of DOY
Day of the year (DOY)
Partial effect of Age
Age (y)
s(Sound level : Females, edf = 0.98)
Sound level [SPLrms(dB re 1 µPa)]
s(Sound level : Males, edf = 0.92)
Sound level [SPLrms(dB re 1 µPa)]

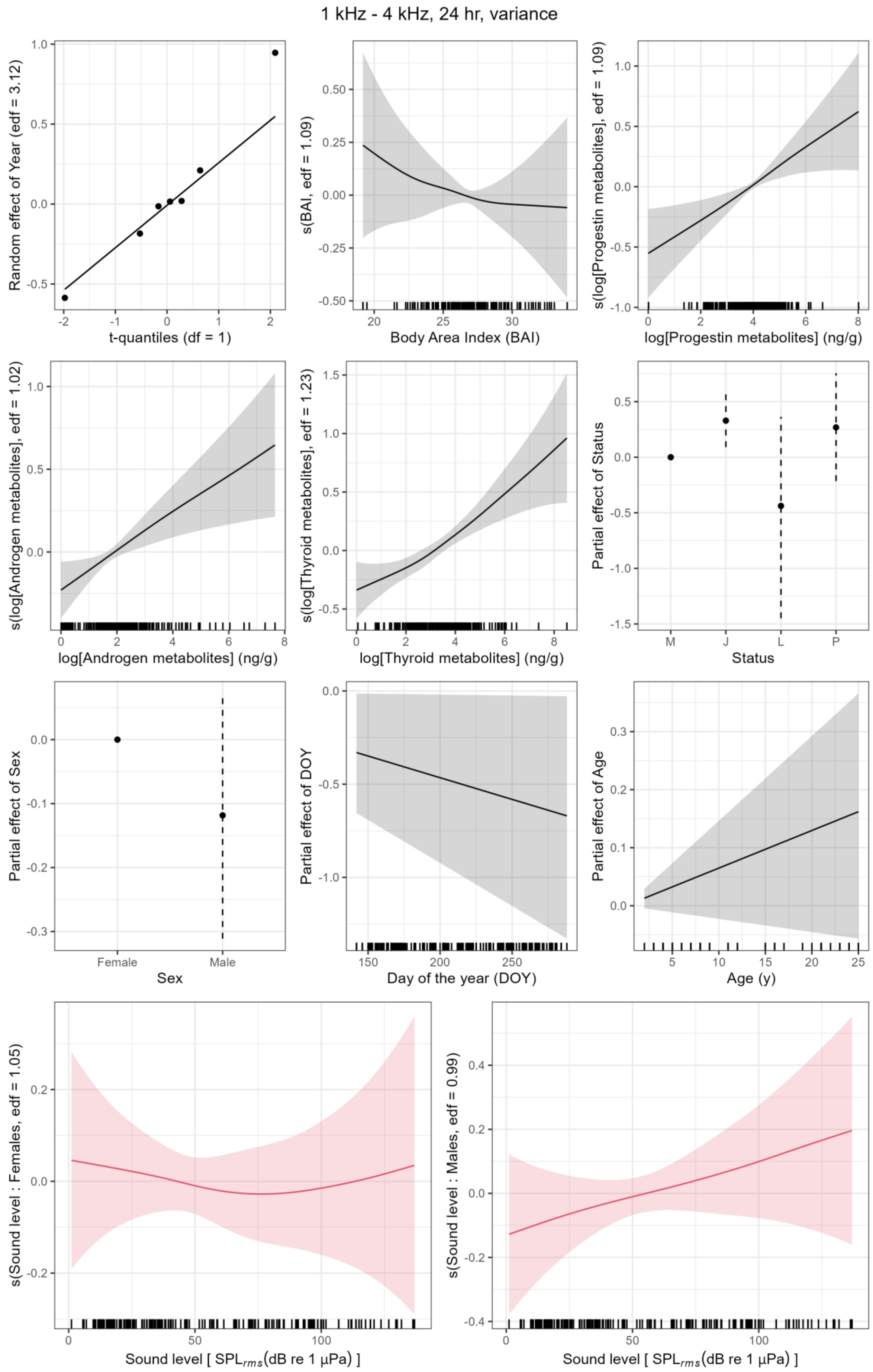
1 kHz - 4 kHz, 24 hr, variance
Random effect of Year (edf = 3.12)
t-quantiles (df = 1)
s(BAI, edf = 1.09)
Body Area Index (BAI)
s(log[Progestin metabolites], edf = 1.09)
log[Progestin metabolites] (ng/g)
s(log[Androgen metabolites], edf = 1.02)
log[Androgen metabolites] (ng/g)
s(log[Thyroid metabolites], edf = 1.23)
log[Thyroid metabolites] (ng/g)
Partial effect of Status
Status
M
J
L
P
Partial effect of Sex
Sex
Female
Male
Partial effect of DOY
Day of the year (DOY)
Partial effect of Age
Age (y)
s(Sound level : Females, edf = 1.05)
Sound level [SPL$_{rms}$(dB re 1 µPa)]
s(Sound level : Males, edf = 0.99)
Sound level [SPL$_{rms}$(dB re 1 µPa)]

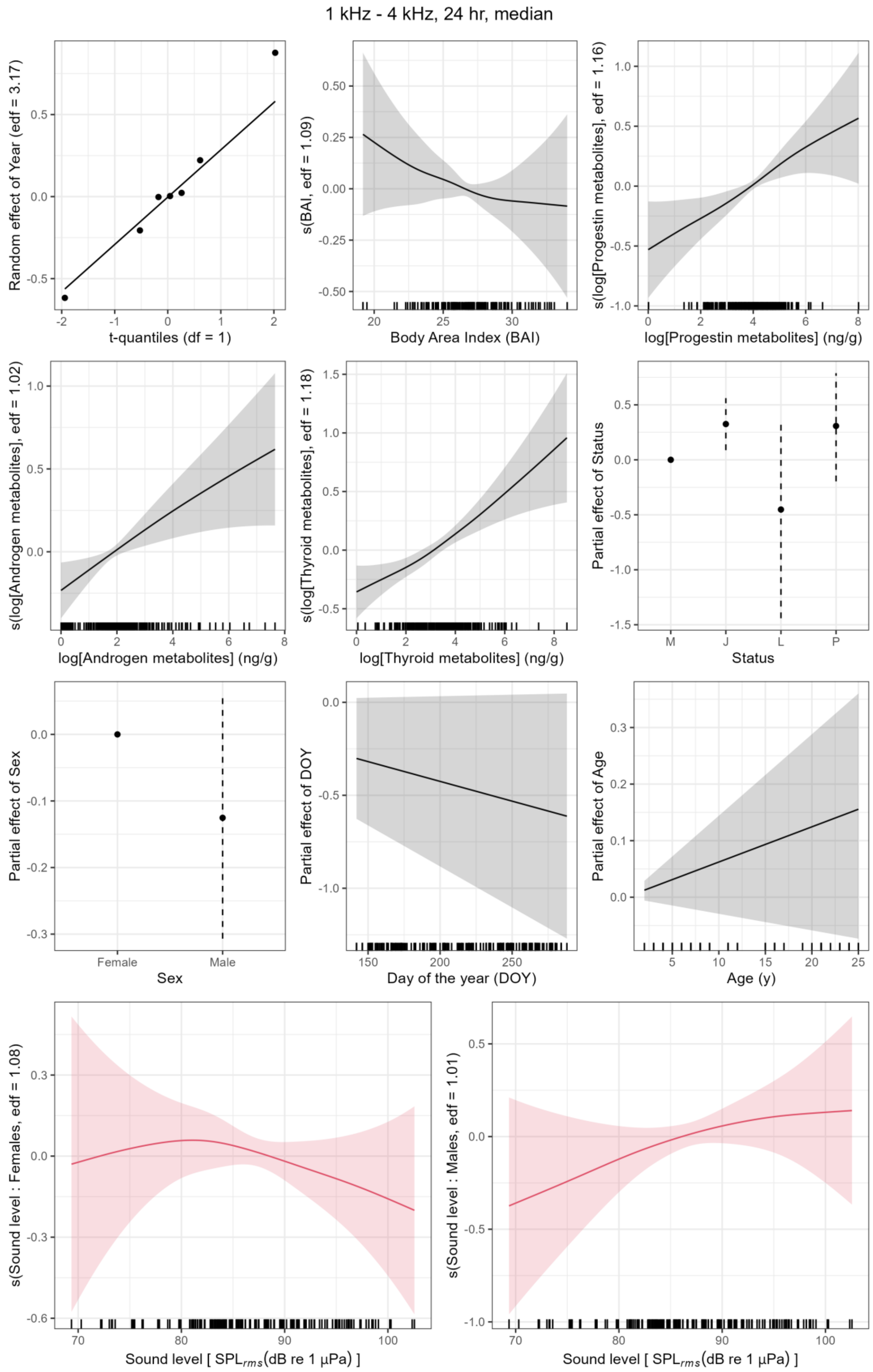

1 kHz - 4 kHz, 24 hr, median
Random effect of Year (edf = 3.17)
t-quantiles (df = 1)
s(BAI, edf = 1.09)
Body Area Index (BAI)
s(log[Progestin metabolites], edf = 1.16)
log[Progestin metabolites] (ng/g)
s(log[Androgen metabolites], edf = 1.02)
log[Androgen metabolites] (ng/g)
s(log[Thyroid metabolites], edf = 1.18)
log[Thyroid metabolites] (ng/g)
Partial effect of Status
Status
M
J
L
P
Partial effect of Sex
Sex
Female
Male
Partial effect of DOY
Day of the year (DOY)
Partial effect of Age
Age (y)
s(Sound level : Females, edf = 1.08)
Sound level [SPL_{rms}(dB re 1 μPa)]
s(Sound level : Males, edf = 1.01)
Sound level [SPL_{rms}(dB re 1 μPa)]

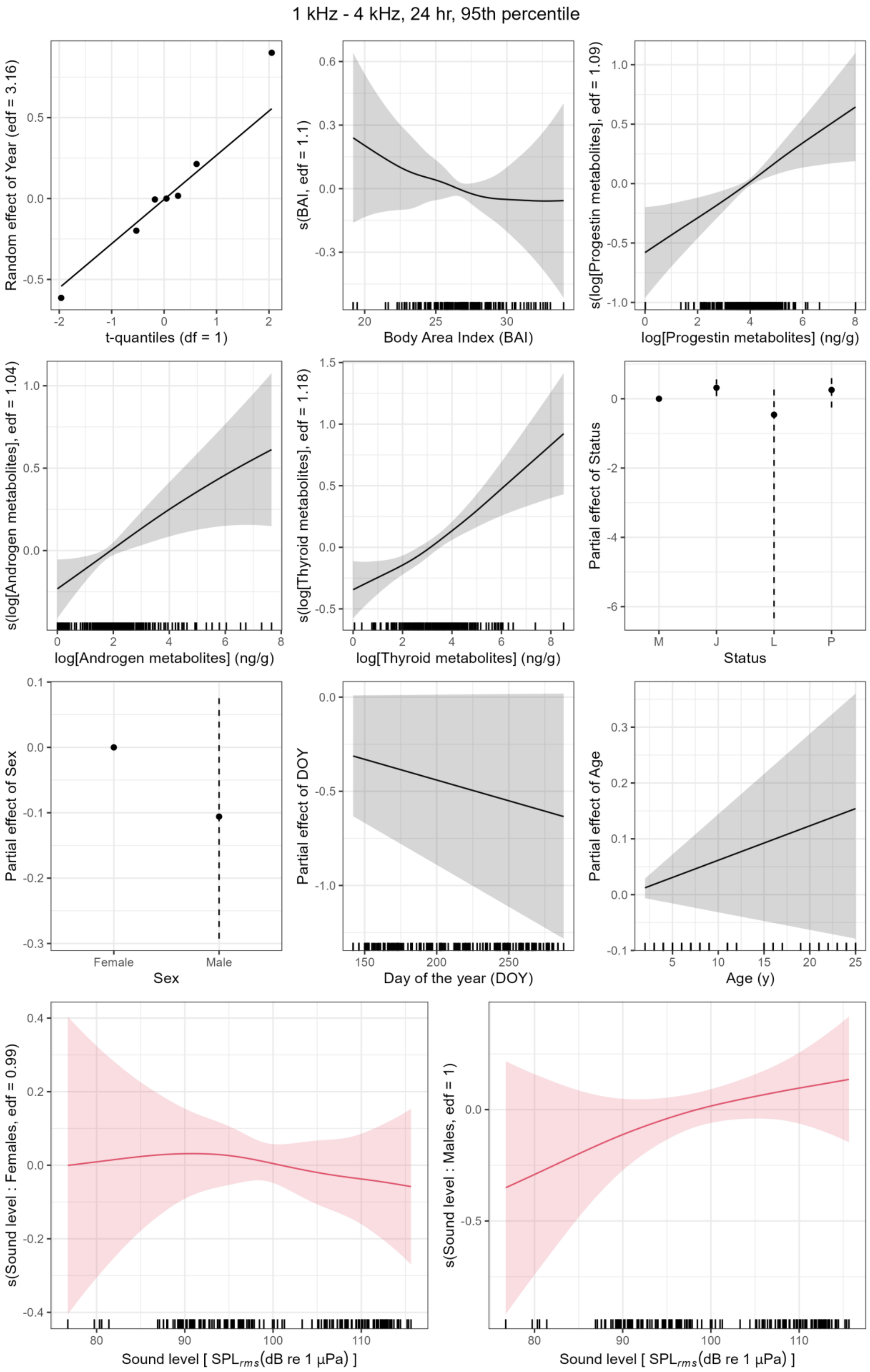
1 kHz - 4 kHz, 24 hr, 95th percentile
Random effect of Year (edf = 3.16)
t-quantiles (df = 1)
s(BAI, edf = 1.1)
Body Area Index (BAI)
s(log[Progestin metabolites], edf = 1.09)
log[Progestin metabolites] (ng/g)
s(log[Androgen metabolites], edf = 1.04)
log[Androgen metabolites] (ng/g)
s(log[Thyroid metabolites], edf = 1.18)
log[Thyroid metabolites] (ng/g)
Partial effect of Status
Status
M
J
L
P
Partial effect of Sex
Sex
Female
Male
Partial effect of DOY
Day of the year (DOY)
Partial effect of Age
Age (y)
s(Sound level : Females, edf = 0.99)
Sound level [SPLrms(dB re 1 μPa)]
s(Sound level : Males, edf = 1)
Sound level [SPLrms(dB re 1 μPa)]

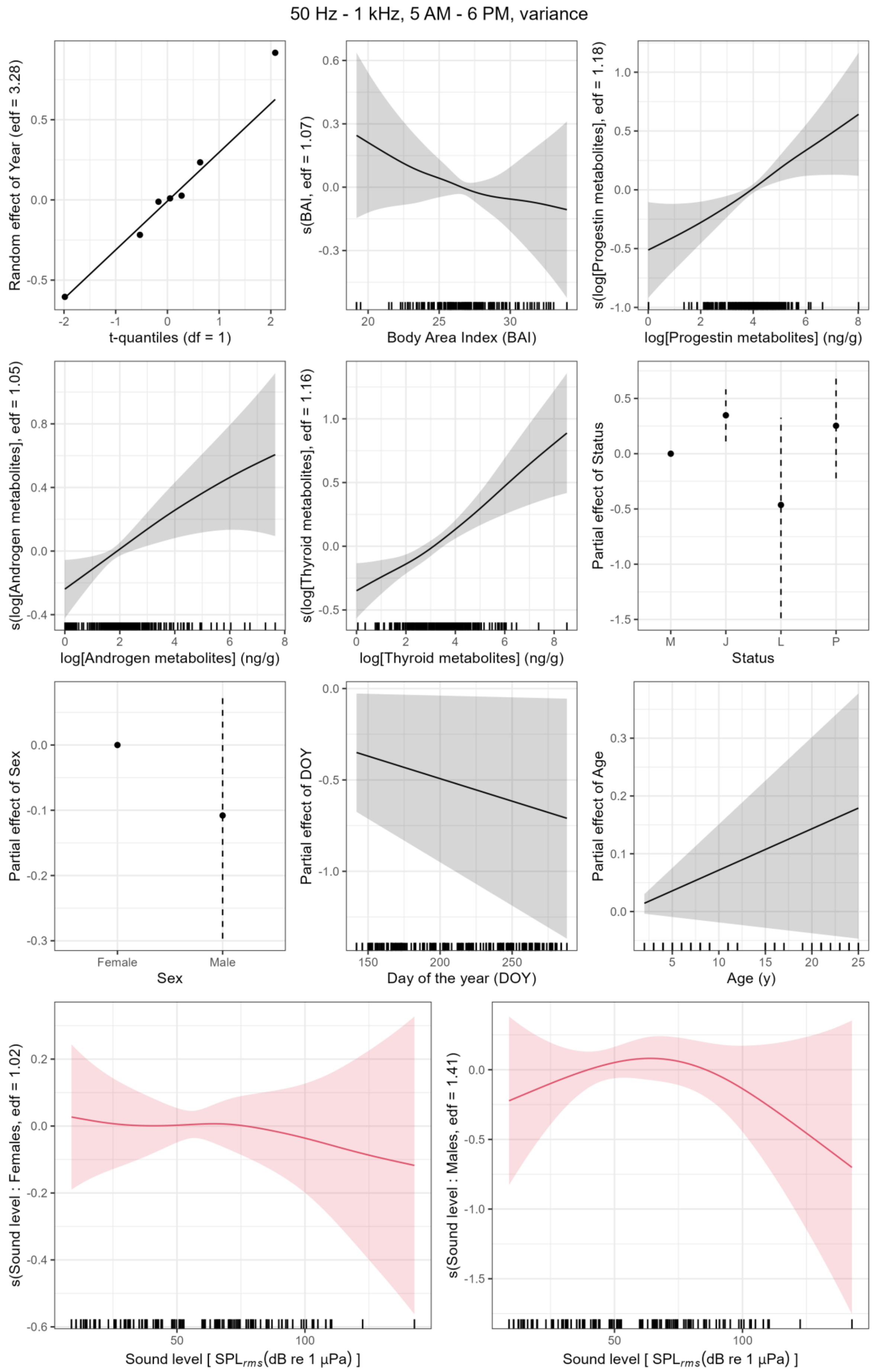
50 Hz - 1 kHz, 5 AM - 6 PM, variance
Random effect of Year (edf = 3.28)
t-quantiles (df = 1)
s(BAI, edf = 1.07)
Body Area Index (BAI)
s(log[Progestin metabolites], edf = 1.18)
log[Progestin metabolites] (ng/g)
s(log[Androgen metabolites], edf = 1.05)
log[Androgen metabolites] (ng/g)
s(log[Thyroid metabolites], edf = 1.16)
log[Thyroid metabolites] (ng/g)
Partial effect of Status
Status
M
J
L
P
Partial effect of Sex
Sex
Female
Male
Partial effect of DOY
Day of the year (DOY)
Partial effect of Age
Age (y)
s(Sound level : Females, edf = 1.02)
Sound level [SPL_{rms}(dB re 1 µPa)]
s(Sound level : Males, edf = 1.41)
Sound level [SPL_{rms}(dB re 1 µPa)]

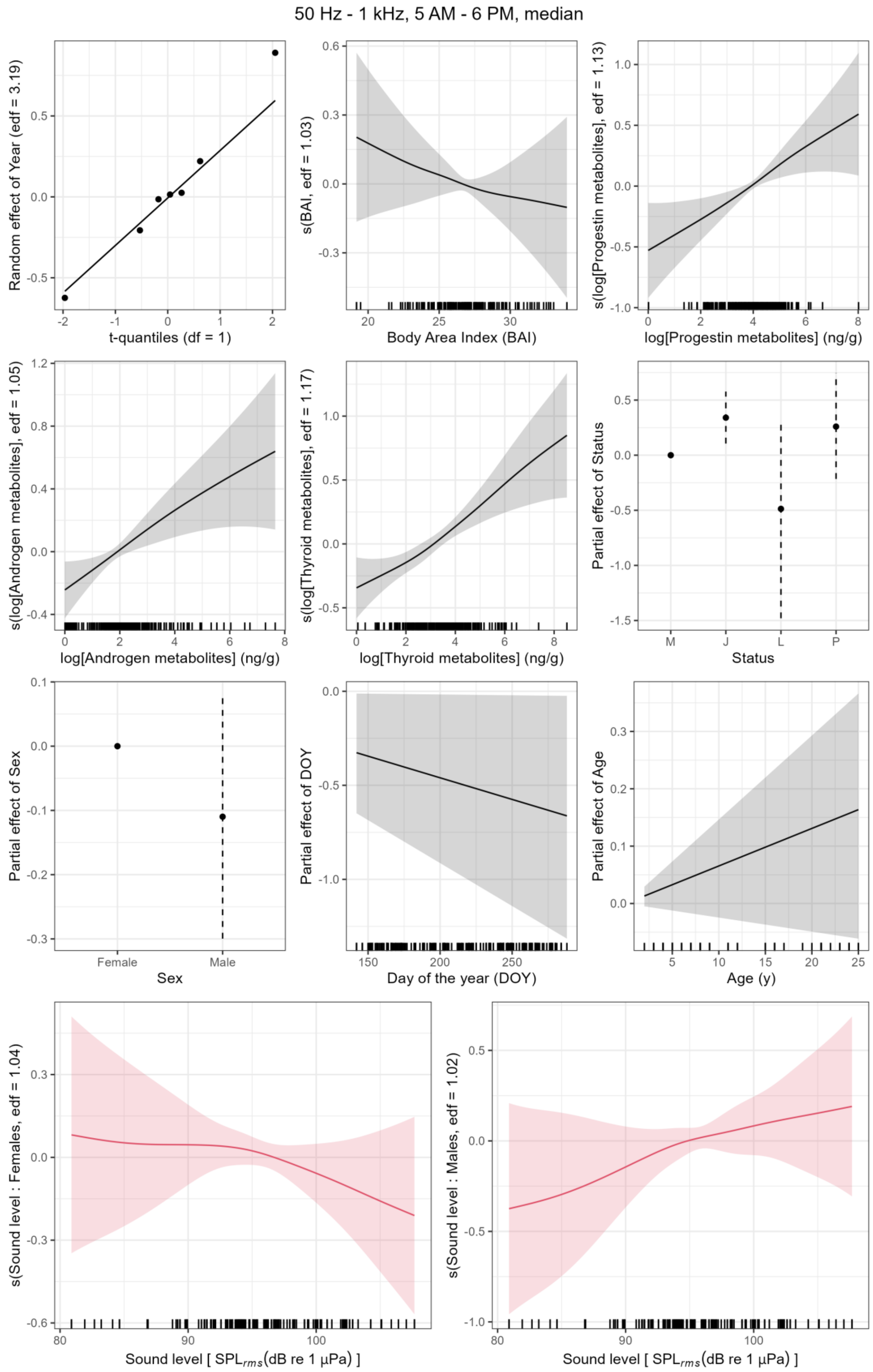
50 Hz - 1 kHz, 5 AM - 6 PM, median
Random effect of Year (edf = 3.19)
t-quantiles (df = 1)
s(BAI, edf = 1.03)
Body Area Index (BAI)
s(log[Progestin metabolites], edf = 1.13)
log[Progestin metabolites] (ng/g)
s(log[Androgen metabolites], edf = 1.05)
log[Androgen metabolites] (ng/g)
s(log[Thyroid metabolites], edf = 1.17)
log[Thyroid metabolites] (ng/g)
Partial effect of Status
Status
M
J
L
P
Partial effect of Sex
Sex
Female
Male
Partial effect of DOY
Day of the year (DOY)
Partial effect of Age
Age (y)
s(Sound level : Females, edf = 1.04)
Sound level [SPLrms(dB re 1 µPa)]
s(Sound level : Males, edf = 1.02)
Sound level [SPLrms(dB re 1 µPa)]

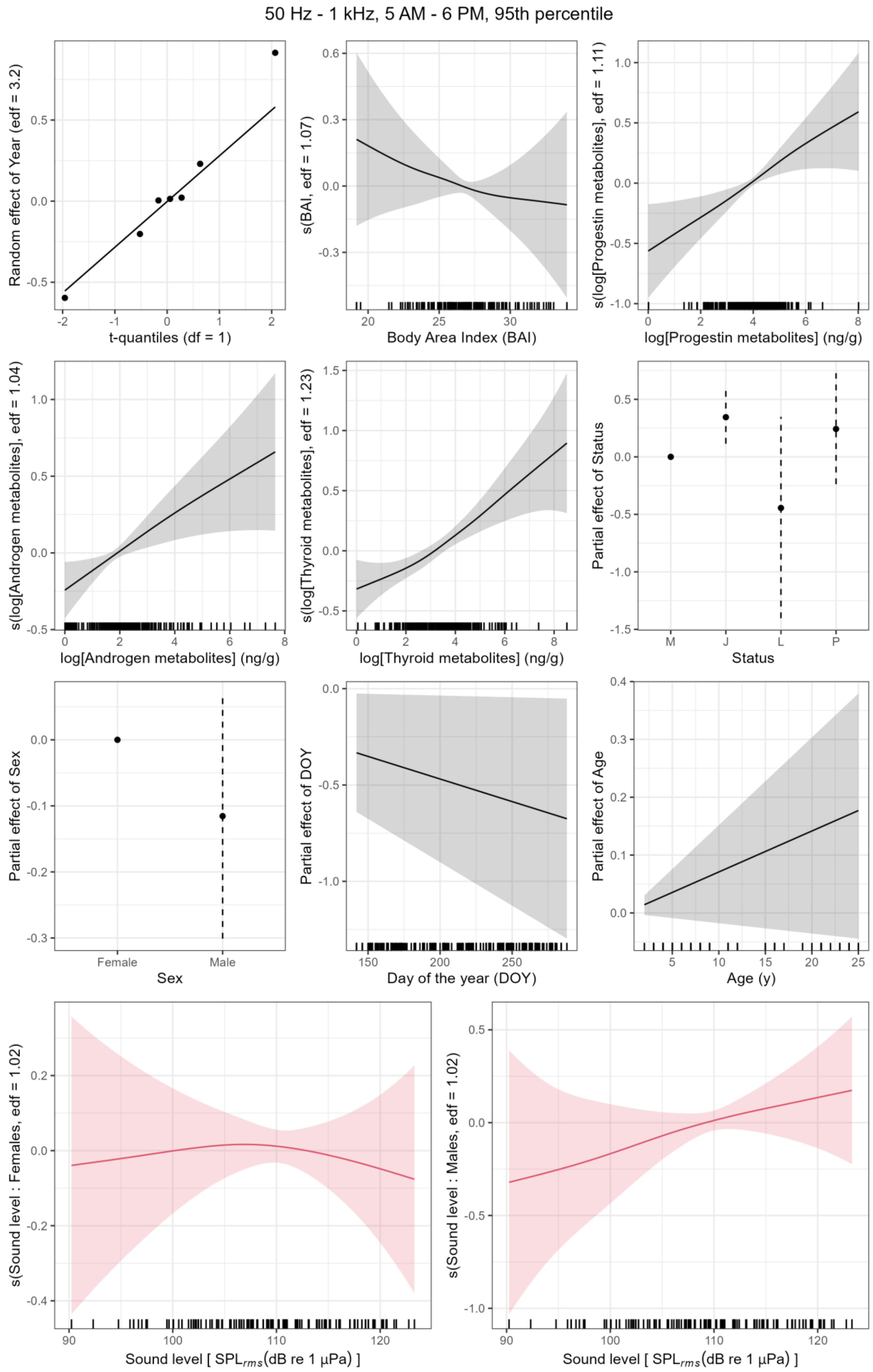

50 Hz - 1 kHz, 5 AM - 6 PM, 95th percentile
Random effect of Year (edf = 3.2)
t-quantiles (df = 1)
s(BAI, edf = 1.07)
Body Area Index (BAI)
s(log[Progestin metabolites], edf = 1.11)
log[Progestin metabolites] (ng/g)
s(log[Androgen metabolites], edf = 1.04)
log[Androgen metabolites] (ng/g)
s(log[Thyroid metabolites], edf = 1.23)
log[Thyroid metabolites] (ng/g)
Partial effect of Status
Status
Partial effect of Sex
Female
Male
Sex
Partial effect of DOY
Day of the year (DOY)
Partial effect of Age
Age (y)
s(Sound level : Females, edf = 1.02)
Sound level [SPL$_{rms}$(dB re 1 μPa)]
s(Sound level : Males, edf = 1.02)
Sound level [SPL$_{rms}$(dB re 1 μPa)]

50 Hz - 1 kHz, 48 hr, variance

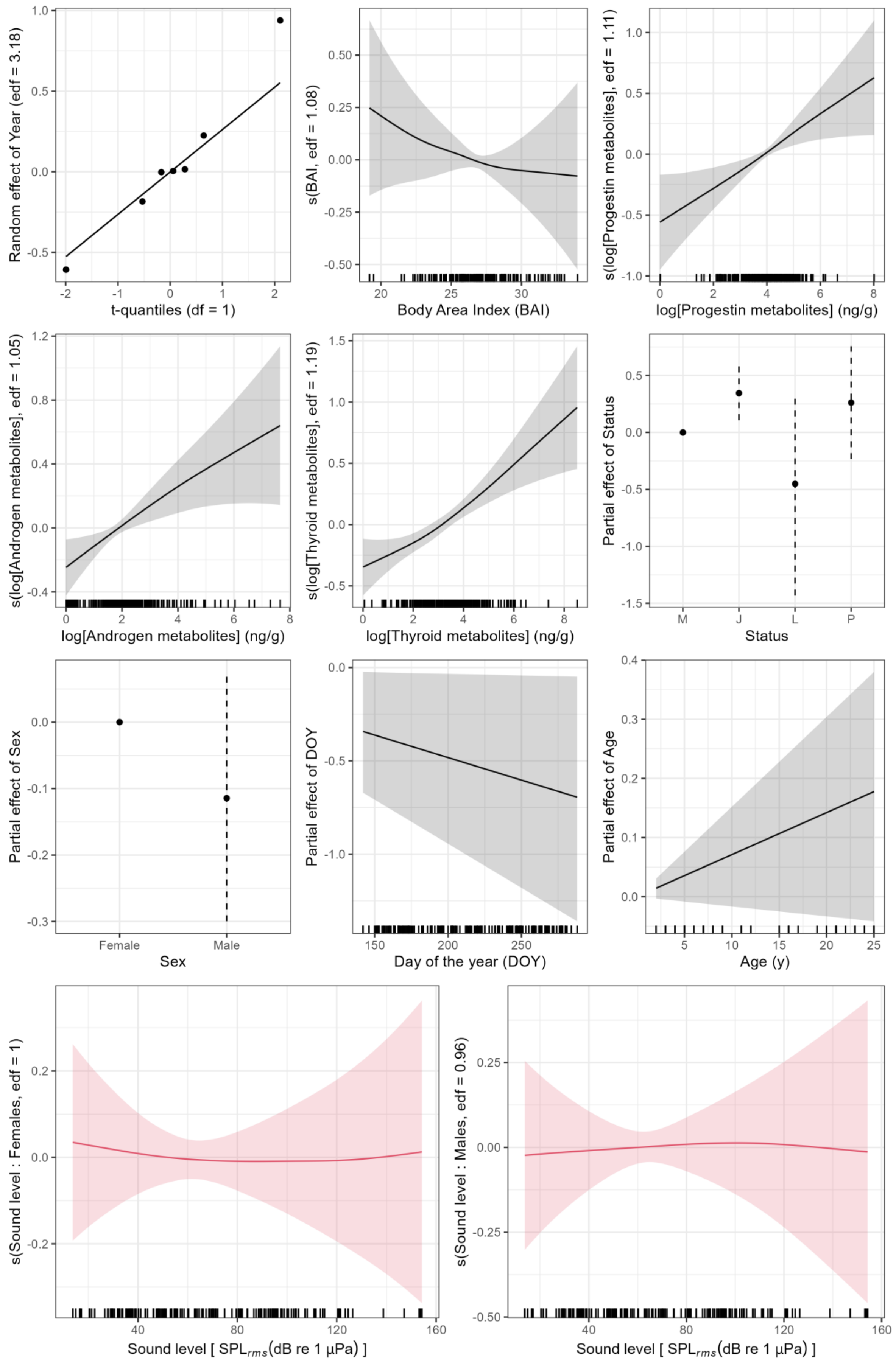

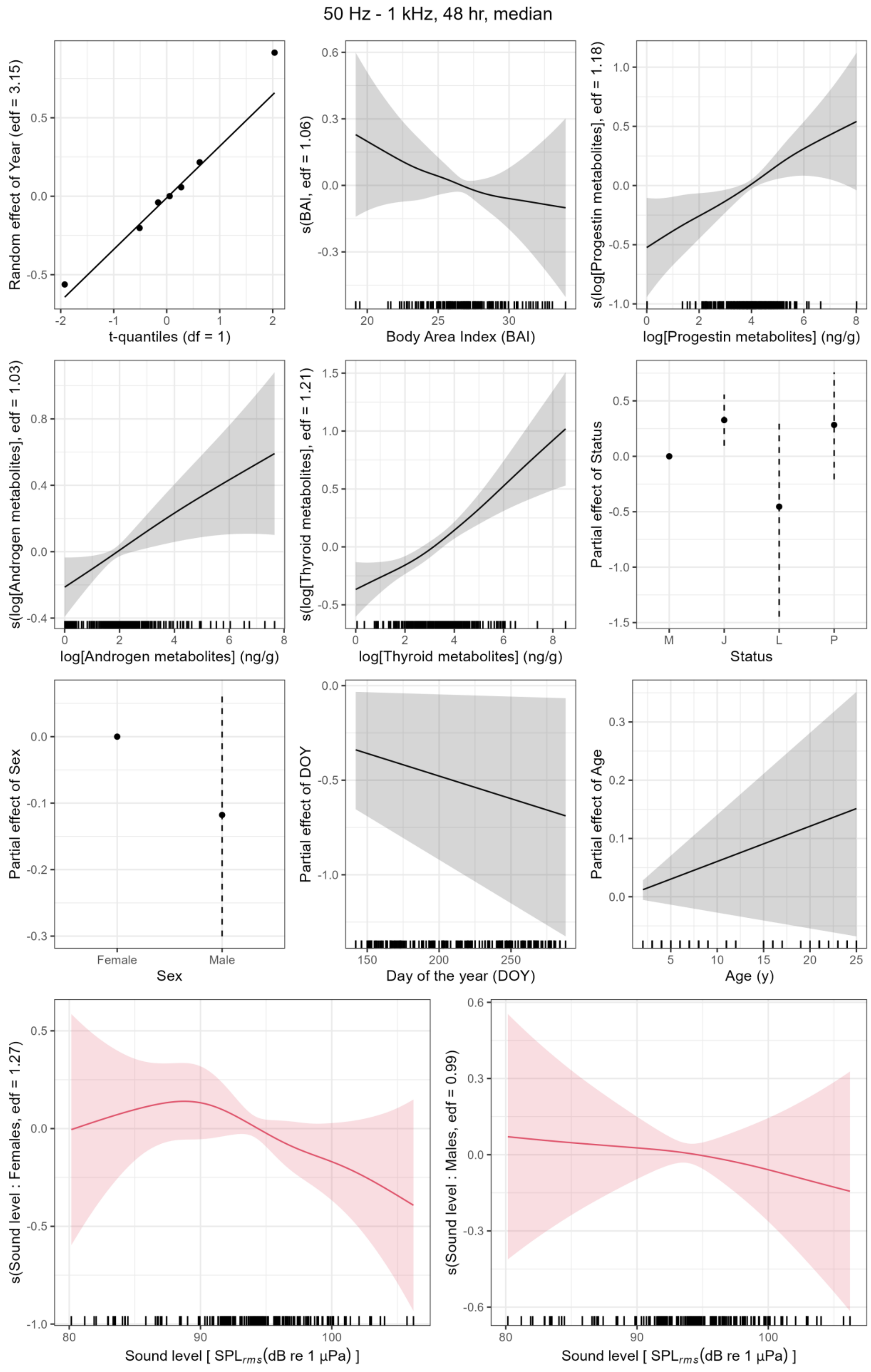
50 Hz - 1 kHz, 48 hr, median
Random effect of Year (edf = 3.15)
t-quantiles (df = 1)
s(BAI, edf = 1.06)
Body Area Index (BAI)
s(log[Progestin metabolites], edf = 1.18)
log[Progestin metabolites] (ng/g)
s(log[Androgen metabolites], edf = 1.03)
log[Androgen metabolites] (ng/g)
s(log[Thyroid metabolites], edf = 1.21)
log[Thyroid metabolites] (ng/g)
Partial effect of Status
Status
M
J
L
P
Partial effect of Sex
Sex
Female
Male
Partial effect of DOY
Day of the year (DOY)
Partial effect of Age
Age (y)
s(Sound level : Females, edf = 1.27)
Sound level [SPLrms(dB re 1 µPa)]
s(Sound level : Males, edf = 0.99)
Sound level [SPLrms(dB re 1 µPa)]

50 Hz - 1 kHz, 48 hr, 95th percentile

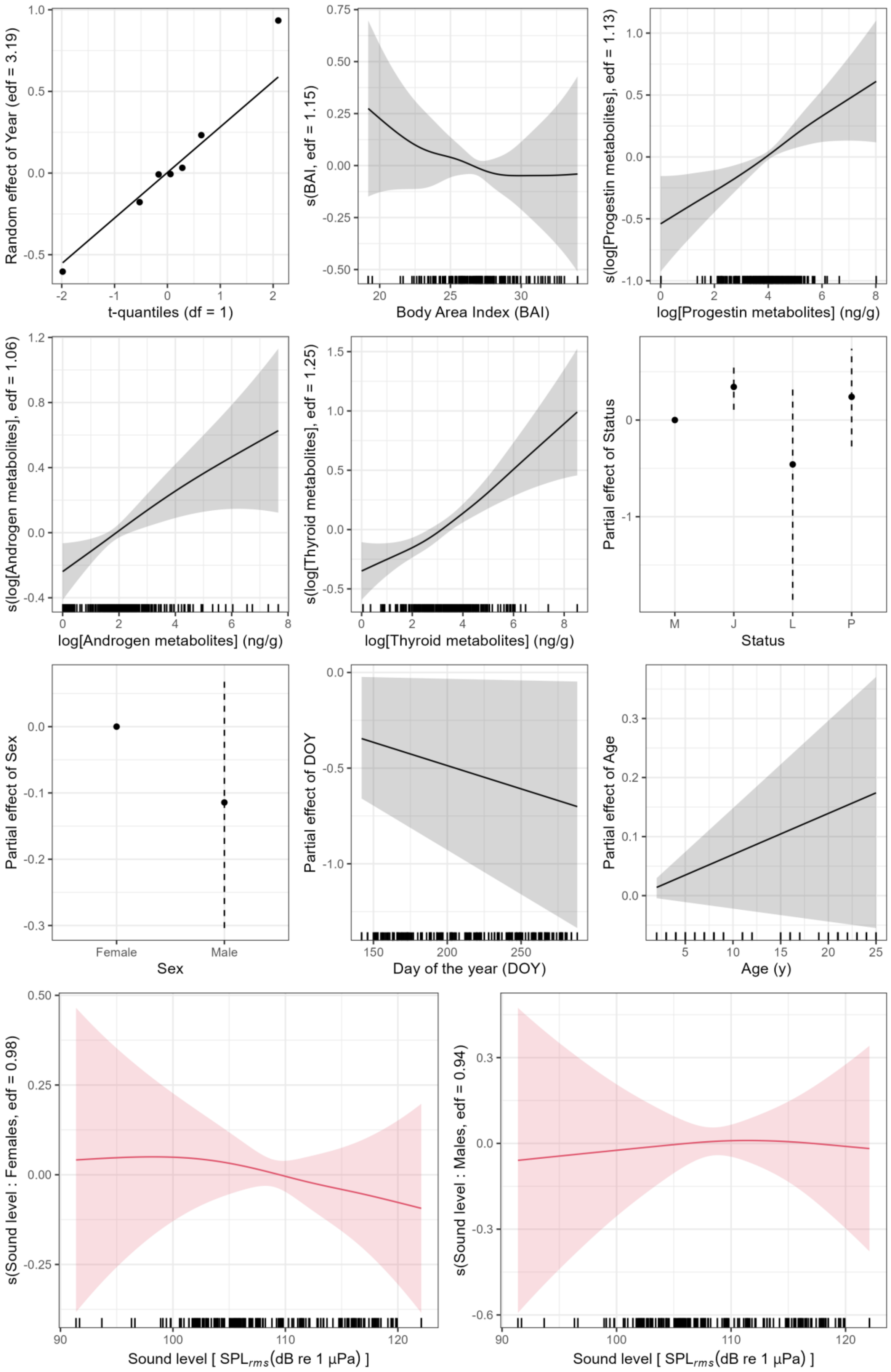

50 Hz - 1 kHz, 24 hr, variance

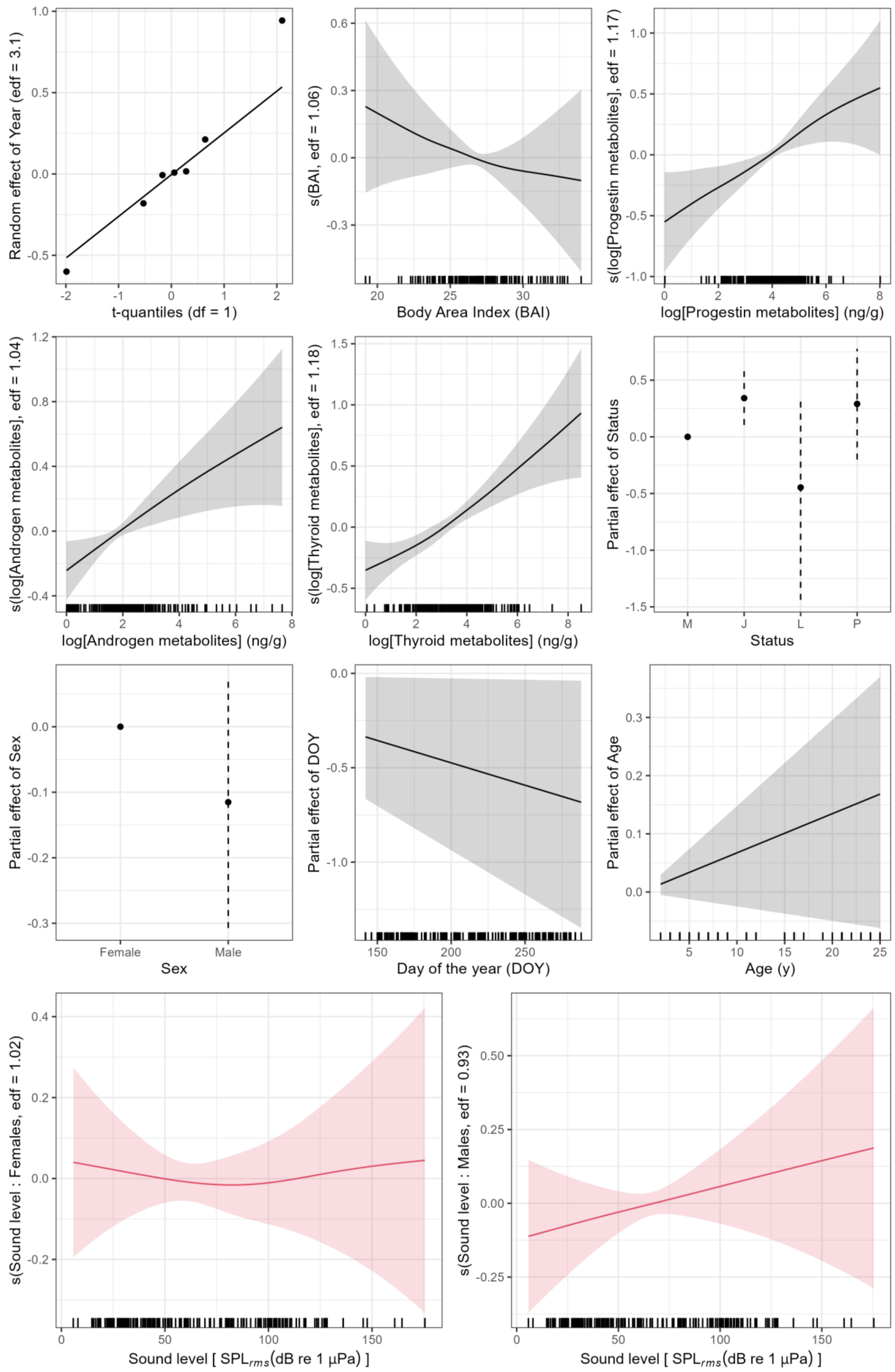

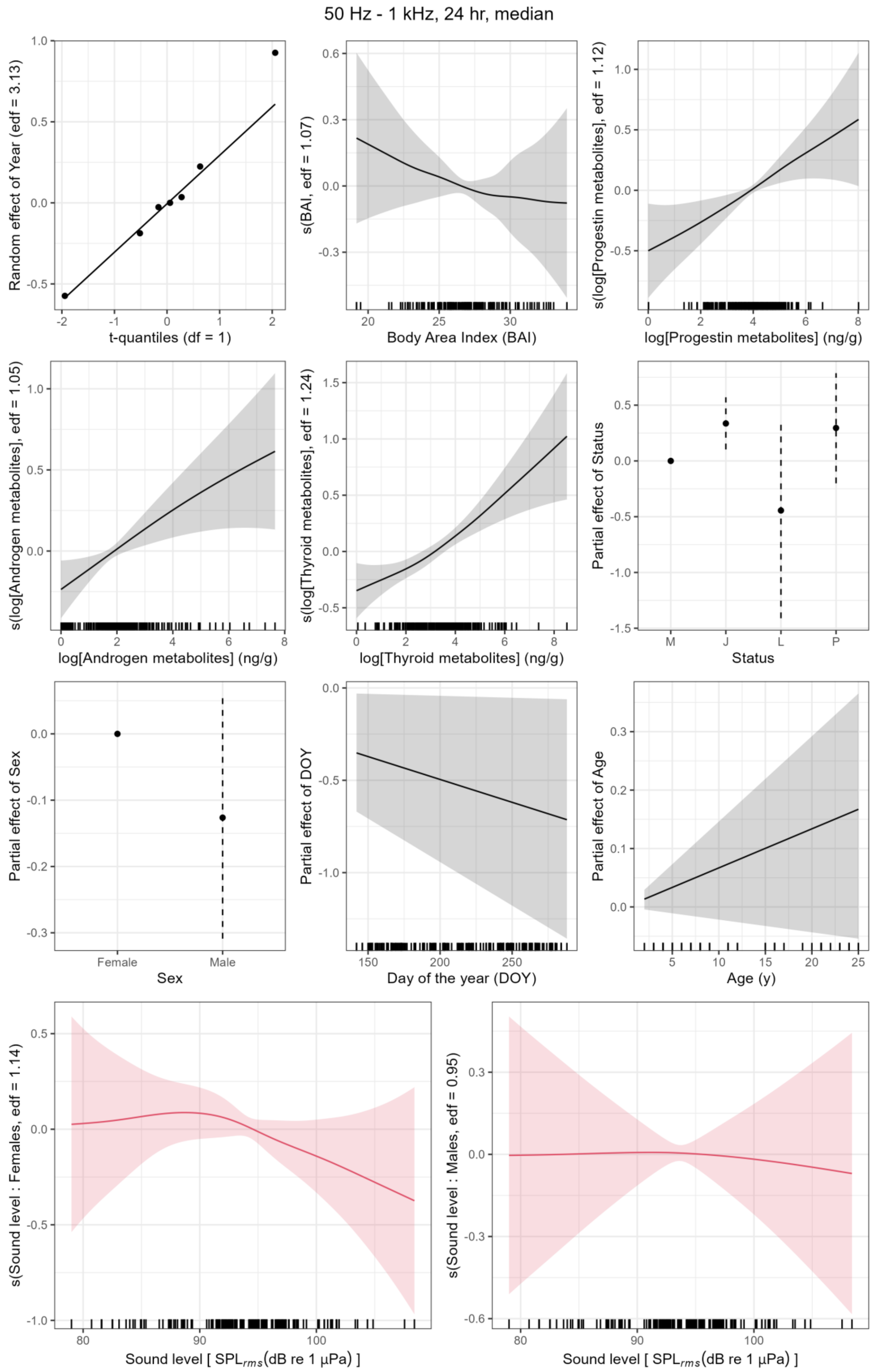
50 Hz - 1 kHz, 24 hr, median
Random effect of Year (edf = 3.13)
t-quantiles (df = 1)
s(BAI, edf = 1.07)
Body Area Index (BAI)
s(log[Progestin metabolites], edf = 1.12)
log[Progestin metabolites] (ng/g)
s(log[Androgen metabolites], edf = 1.05)
log[Androgen metabolites] (ng/g)
s(log[Thyroid metabolites], edf = 1.24)
log[Thyroid metabolites] (ng/g)
Partial effect of Status
Status
M
J
L
P
Partial effect of Sex
Sex
Female
Male
Partial effect of DOY
Day of the year (DOY)
Partial effect of Age
Age (y)
s(Sound level : Females, edf = 1.14)
Sound level [SPL_{rms}(dB re 1 µPa)]
s(Sound level : Males, edf = 0.95)
Sound level [SPL_{rms}(dB re 1 µPa)]

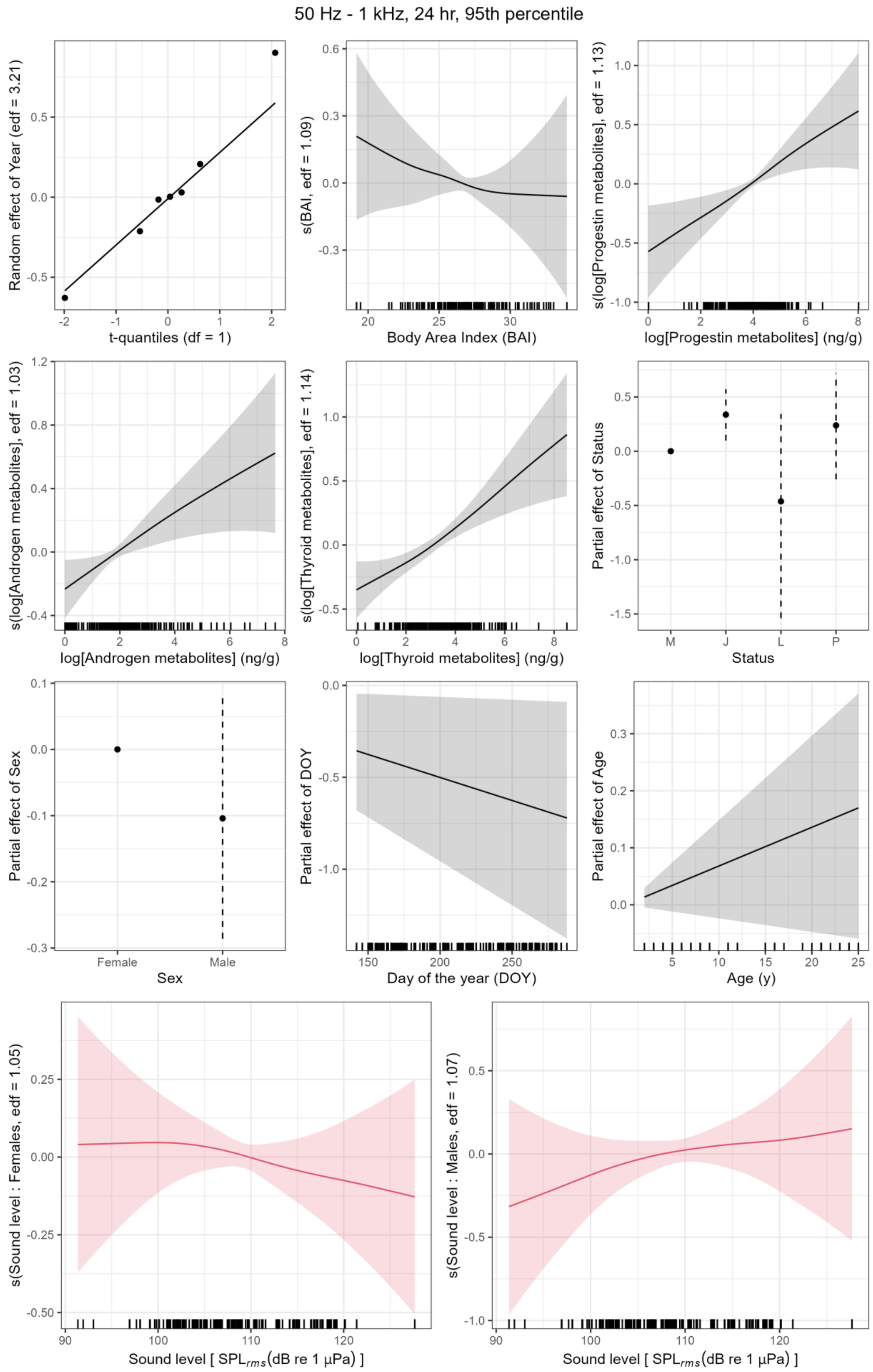
50 Hz - 1 kHz, 24 hr, 95th percentile
Random effect of Year (edf = 3.21)
t-quantiles (df = 1)
s(BAI, edf = 1.09)
Body Area Index (BAI)
s(log[Progestin metabolites], edf = 1.13)
log[Progestin metabolites] (ng/g)
s(log[Androgen metabolites], edf = 1.03)
log[Androgen metabolites] (ng/g)
s(log[Thyroid metabolites], edf = 1.14)
log[Thyroid metabolites] (ng/g)
Partial effect of Status
M
J
L
P
Status
Partial effect of Sex
Female
Male
Sex
Partial effect of DOY
Day of the year (DOY)
Partial effect of Age
Age (y)
s(Sound level : Females, edf = 1.05)
Sound level [SPL$_{rms}$(dB re 1 µPa)]
s(Sound level : Males, edf = 1.07)
Sound level [SPL$_{rms}$(dB re 1 µPa)]

1 kHz - 4 kHz, 5 AM - 6 PM, variance

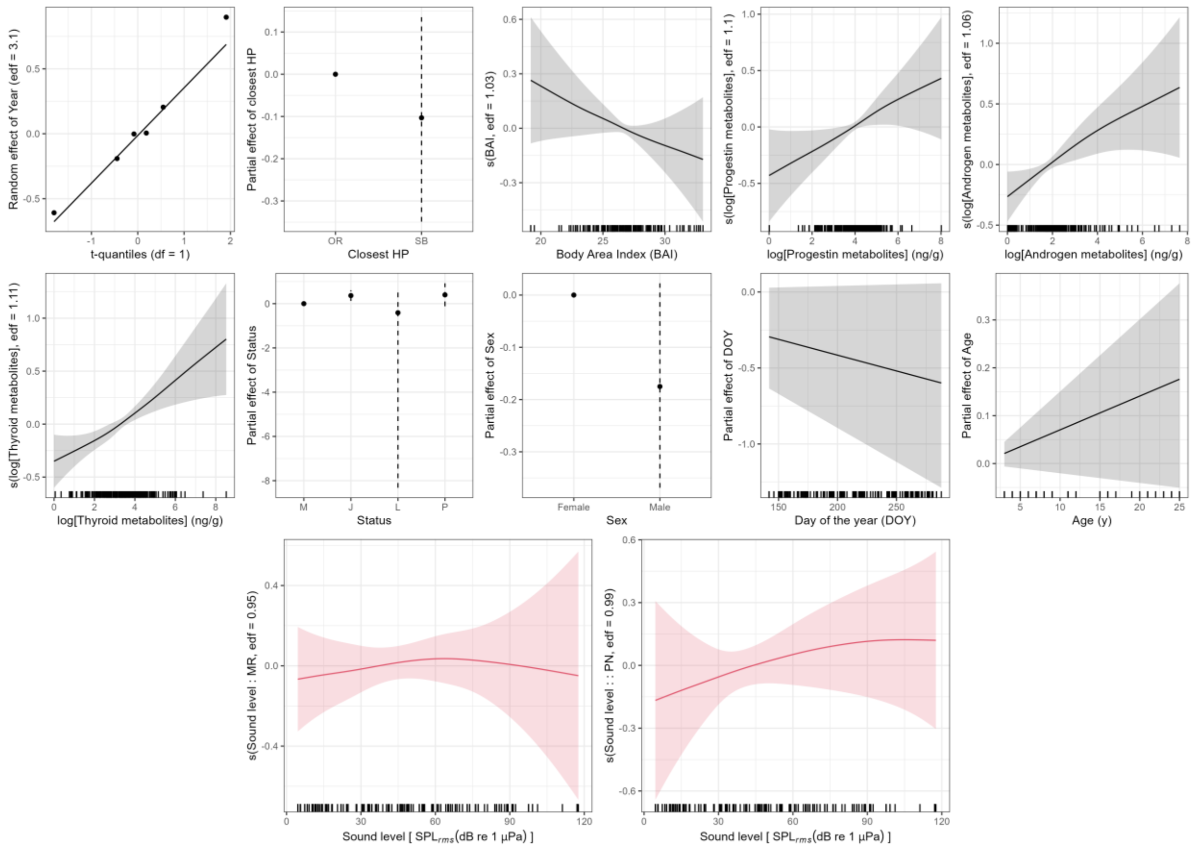

1 kHz - 4 kHz, 5 AM - 6 PM, median

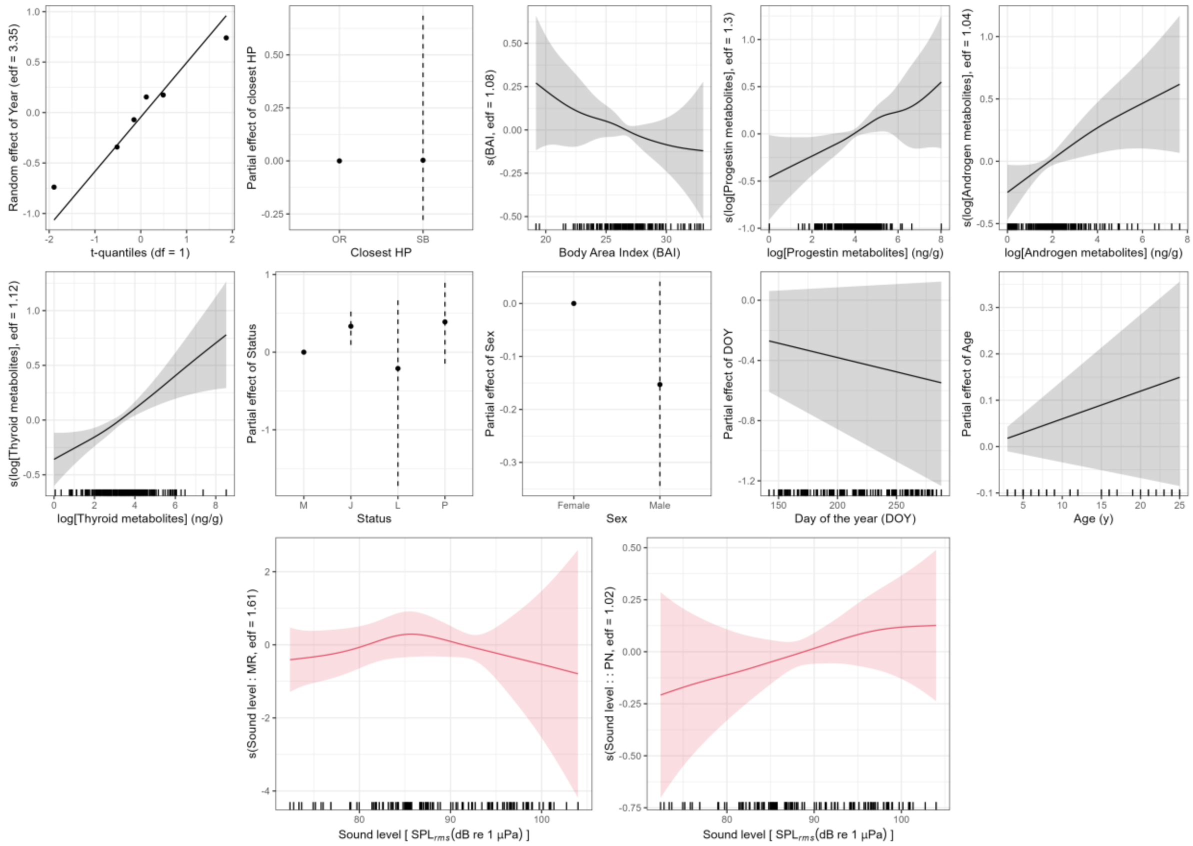

1 kHz - 4 kHz, 5 AM - 6 PM, 95th percentile

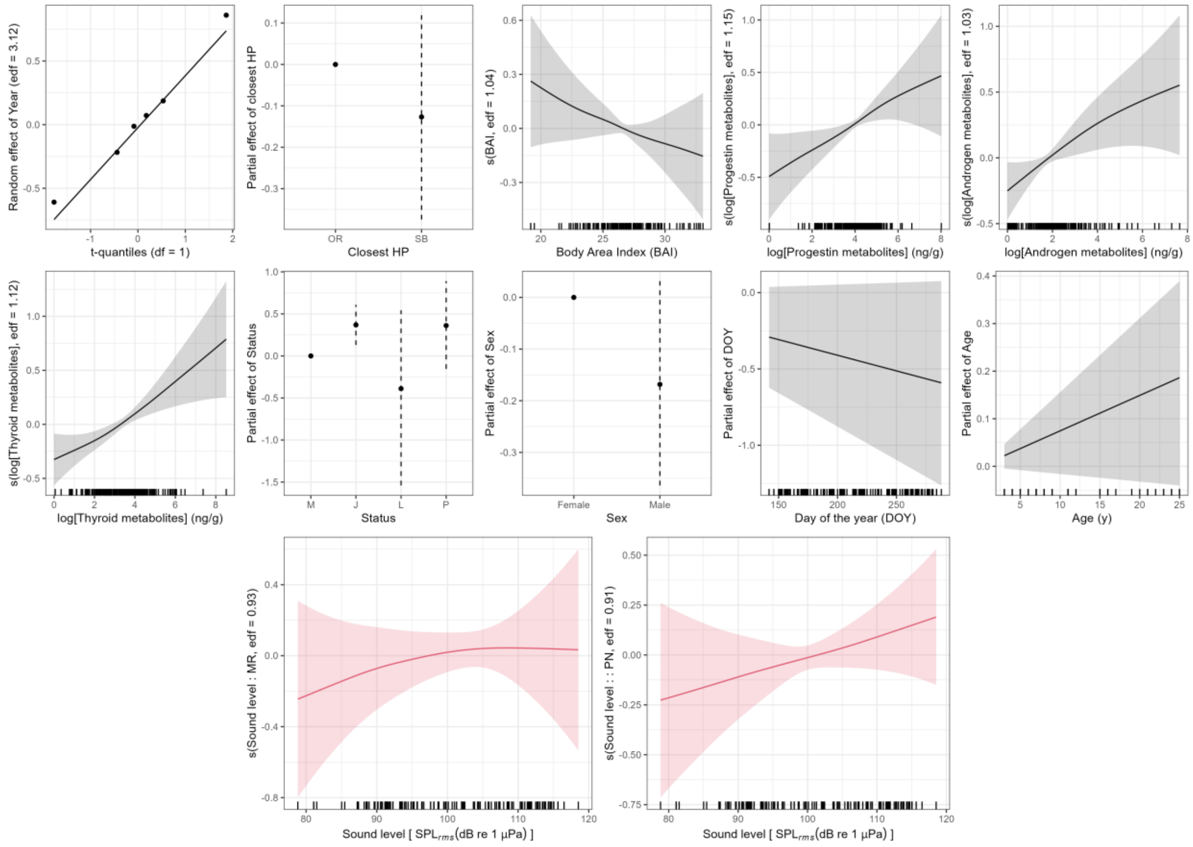

1 kHz - 4 kHz, 48 hr, variance

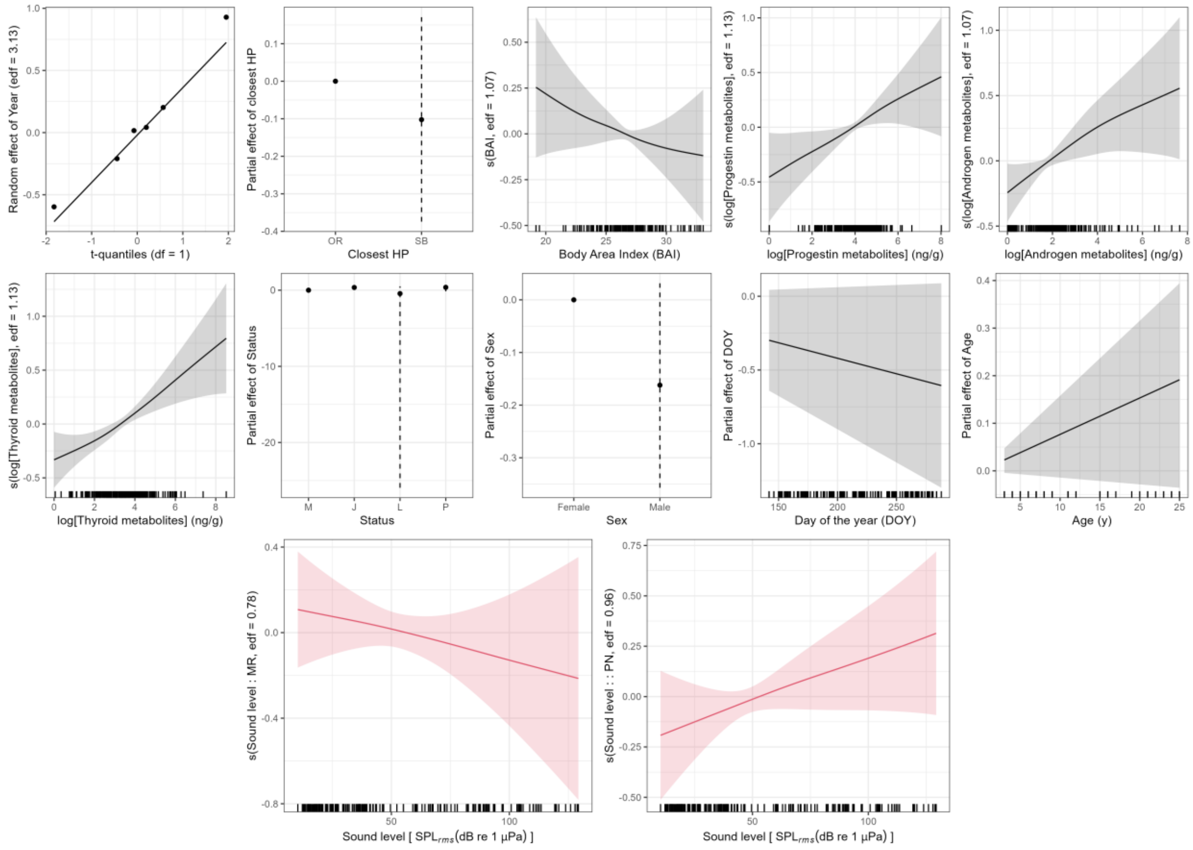

1 kHz - 4 kHz, 48 hr, median

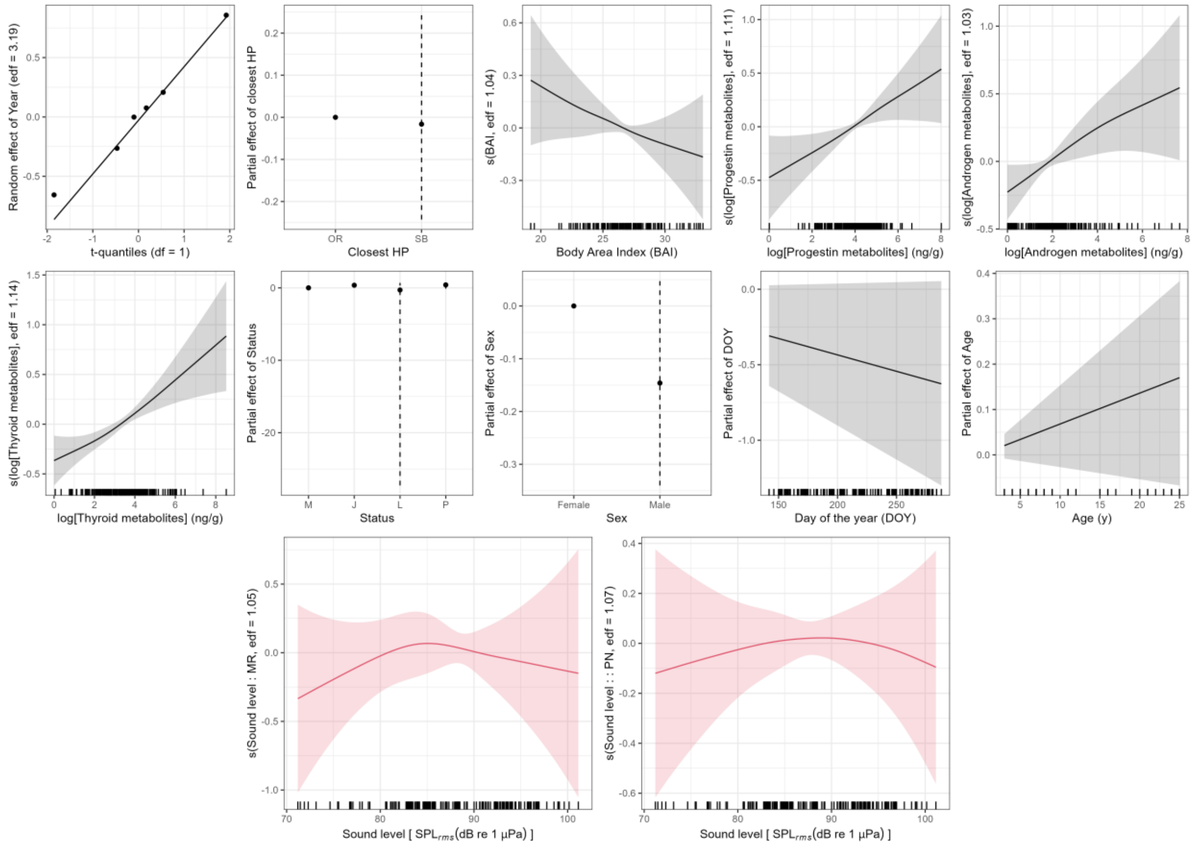

1 kHz - 4 kHz, 48 hr, 95th percentile

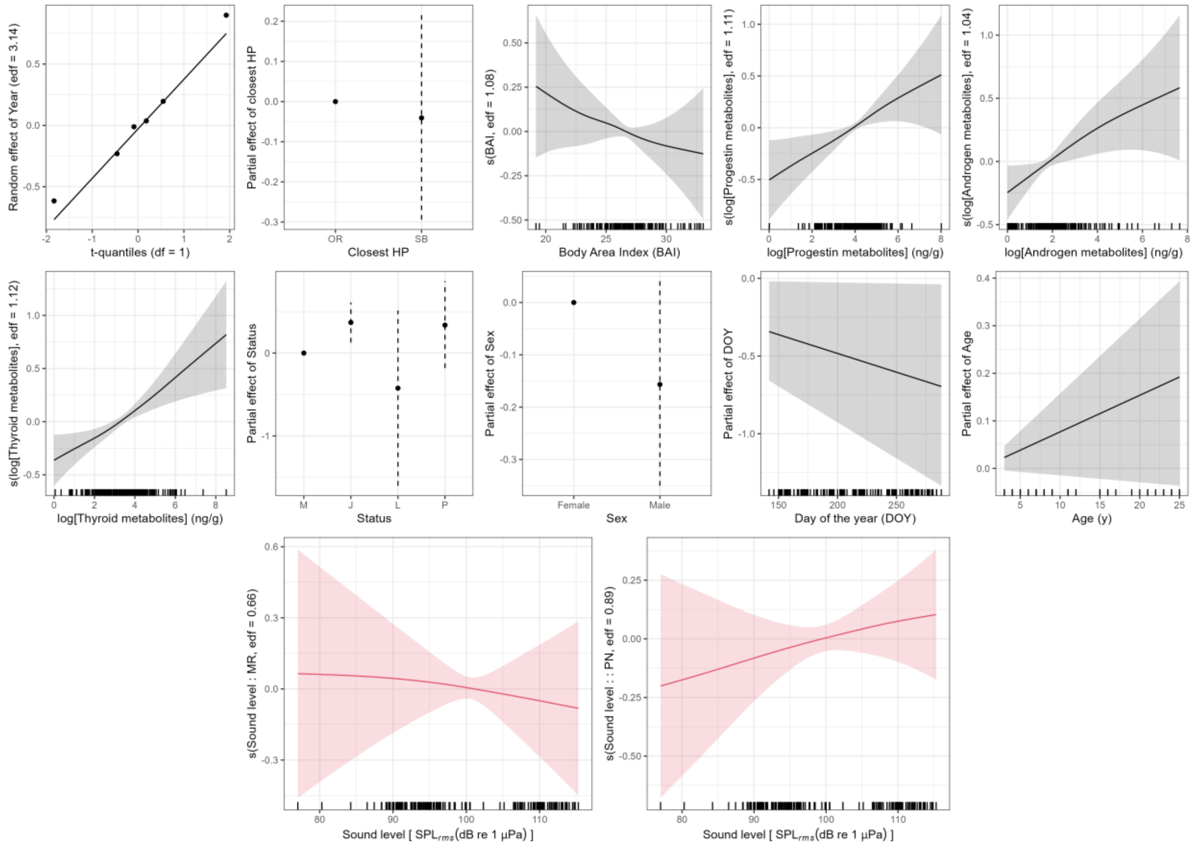

1 kHz - 4 kHz, 24 hr, variance

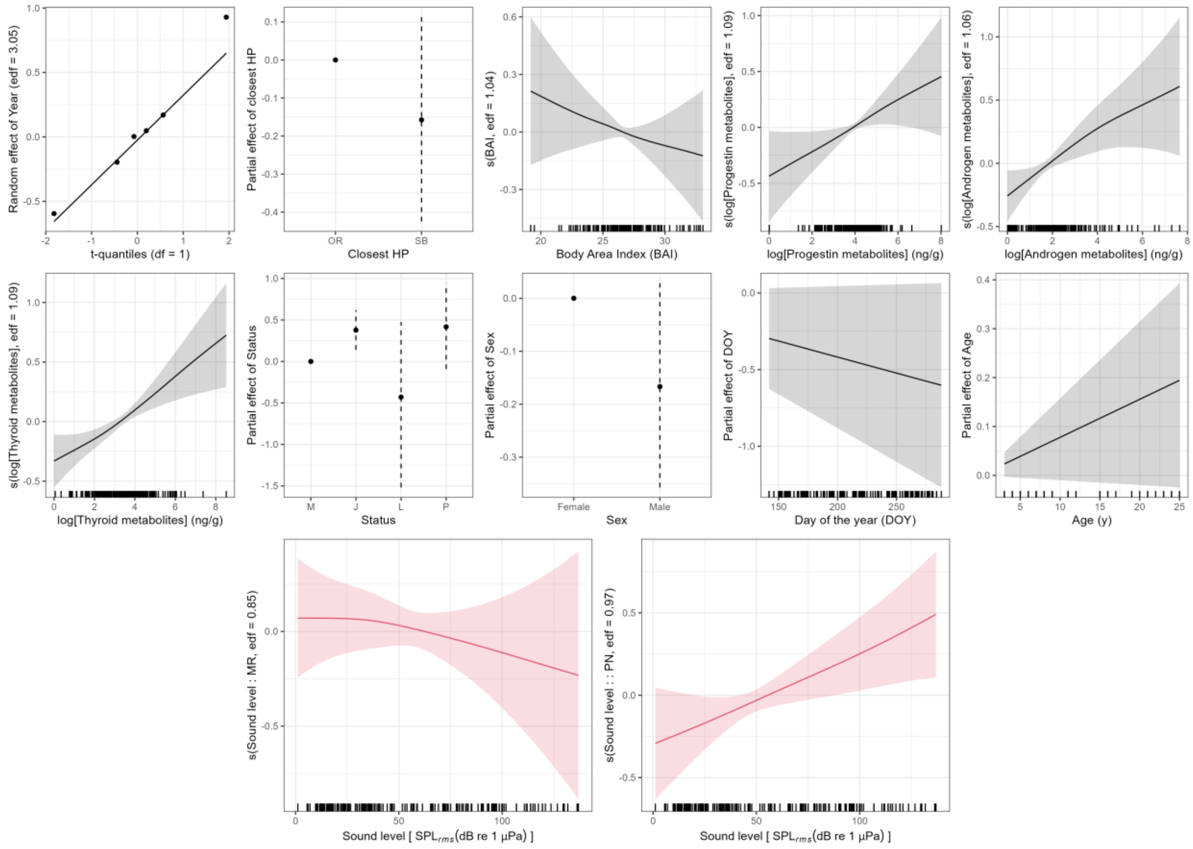

1 kHz - 4 kHz, 24 hr, median

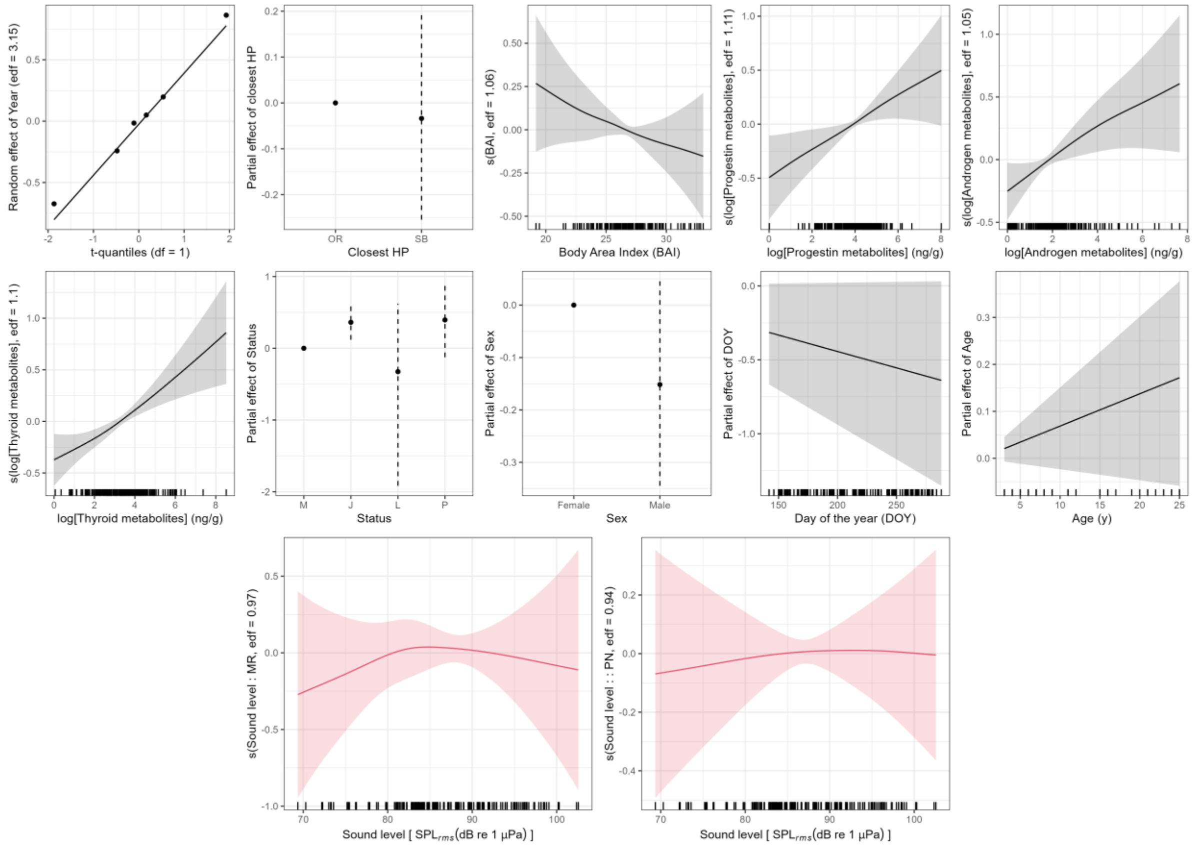

1 kHz - 4 kHz, 24 hr, 95th percentile

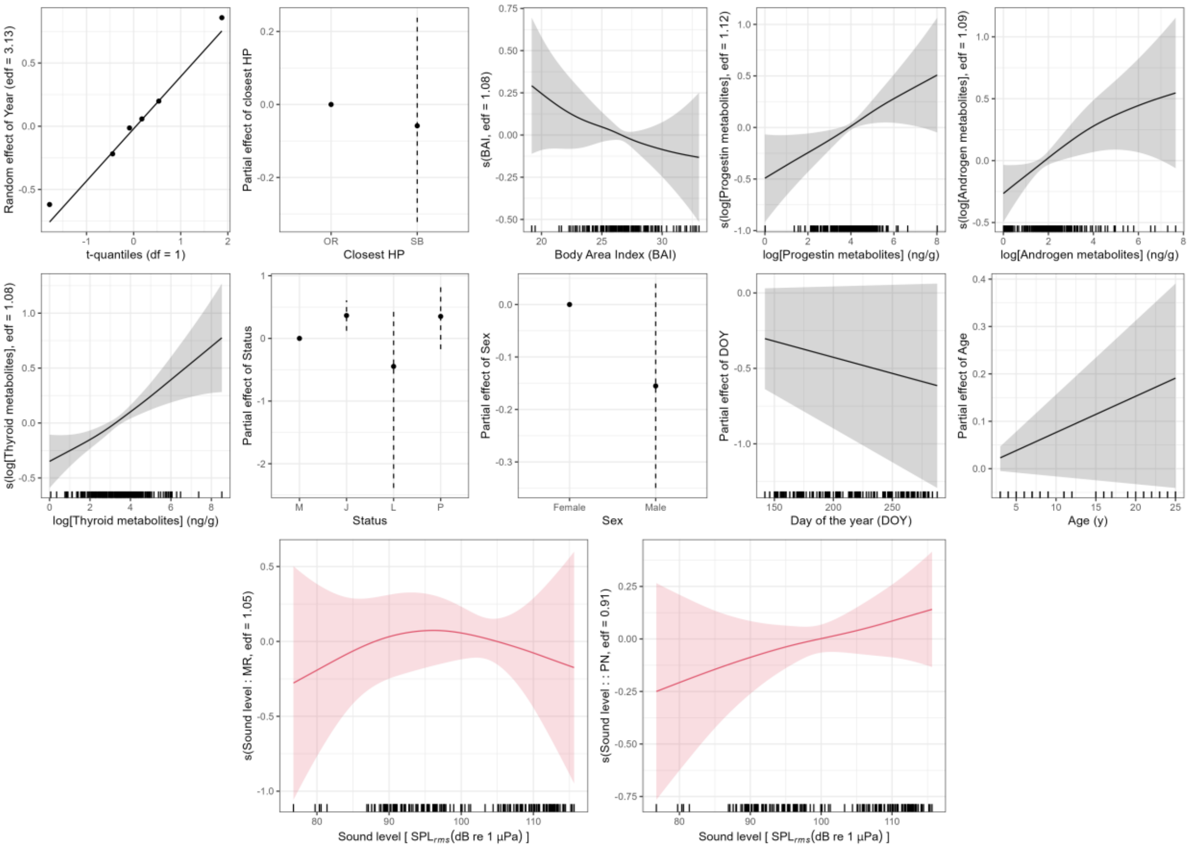

50 Hz - 1 kHz, 5 AM - 6 PM, variance

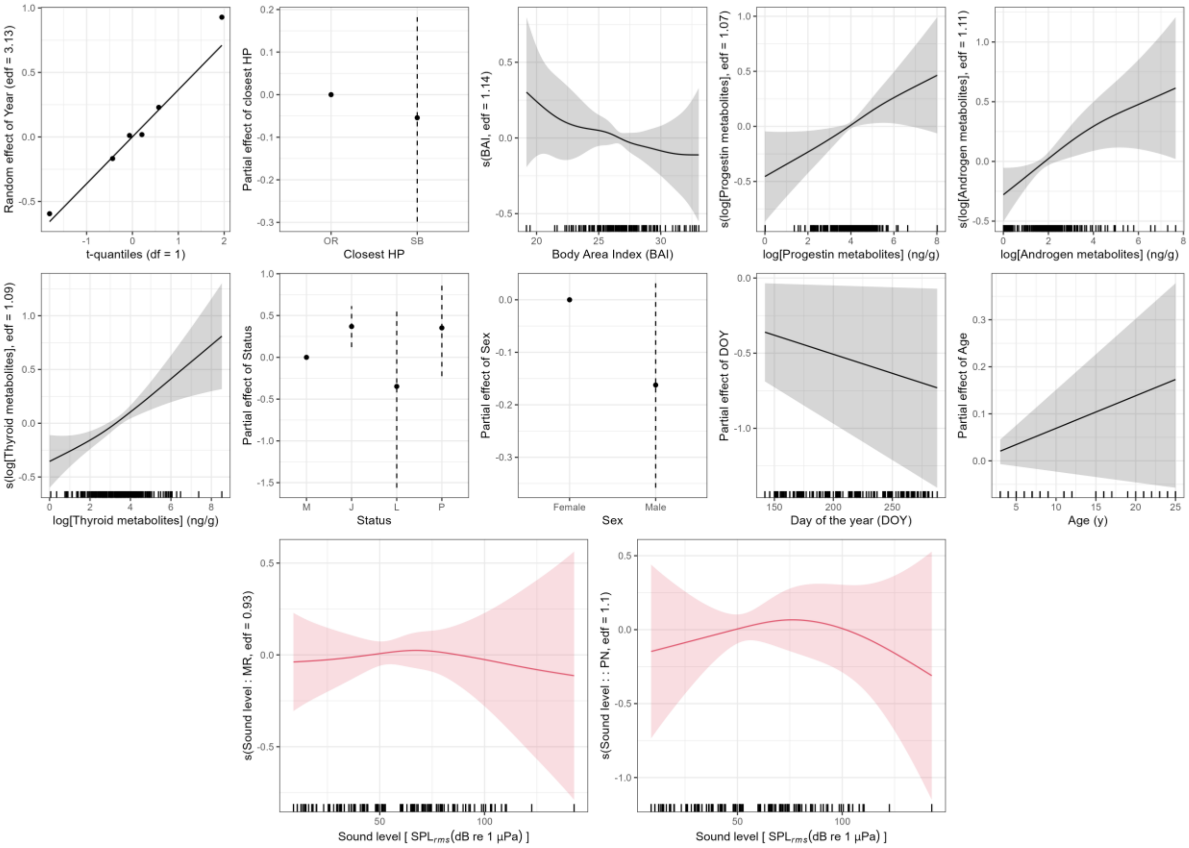

50 Hz - 1 kHz, 5 AM - 6 PM, median

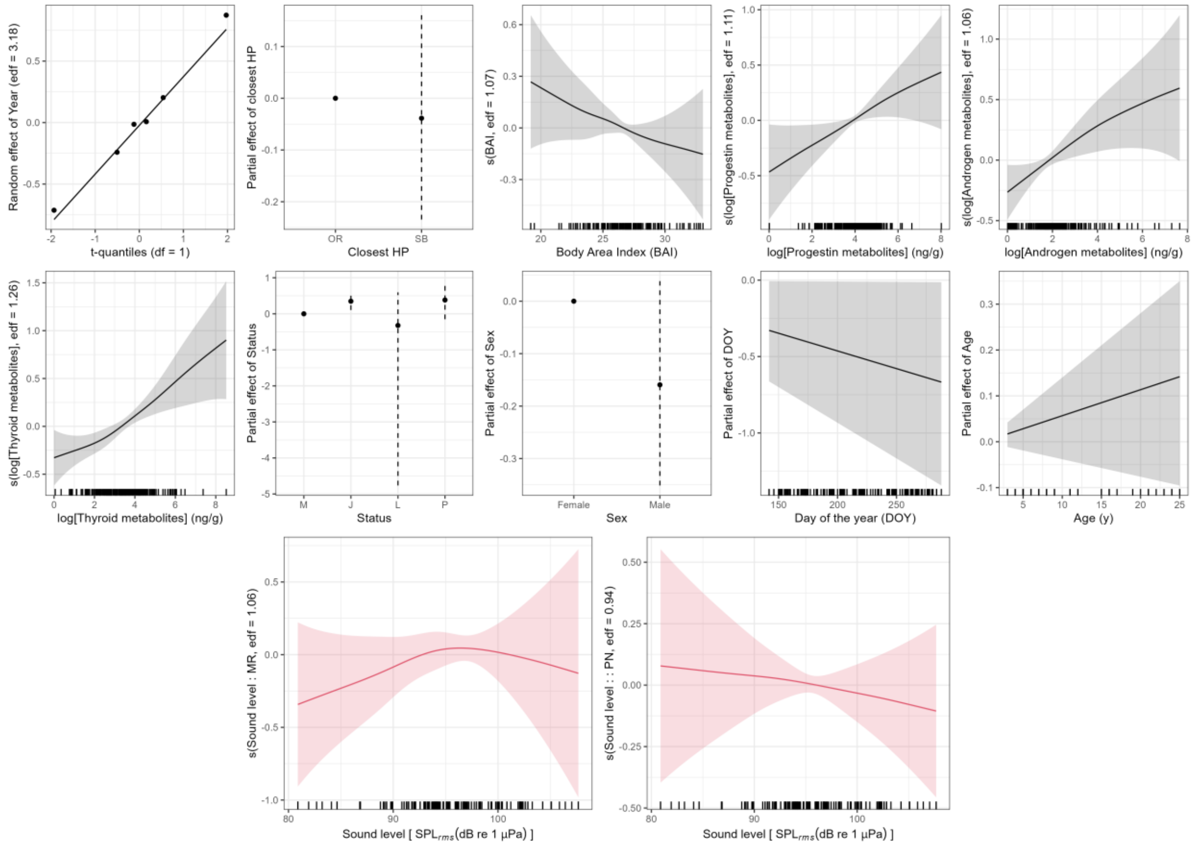

50 Hz - 1 kHz, 5 AM - 6 PM, 95th percentile

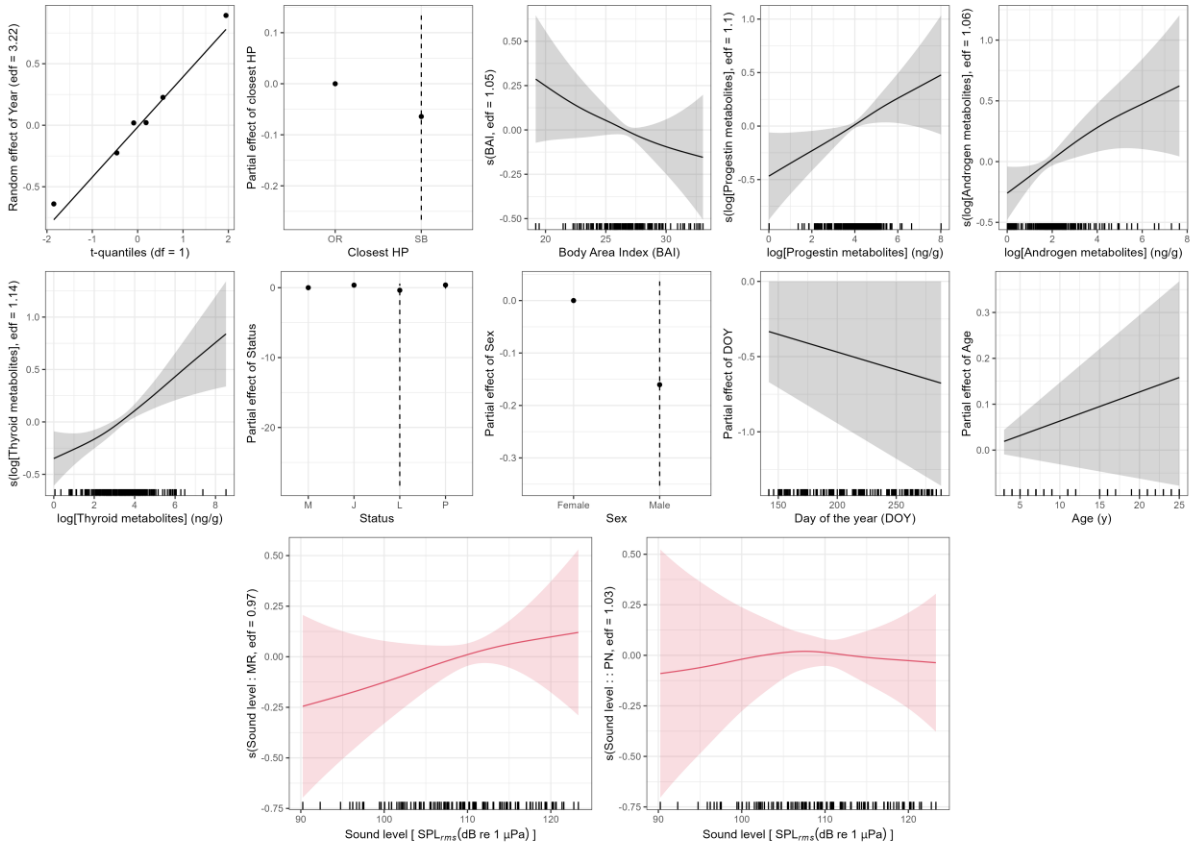

50 Hz - 1 kHz, 48 hr, variance

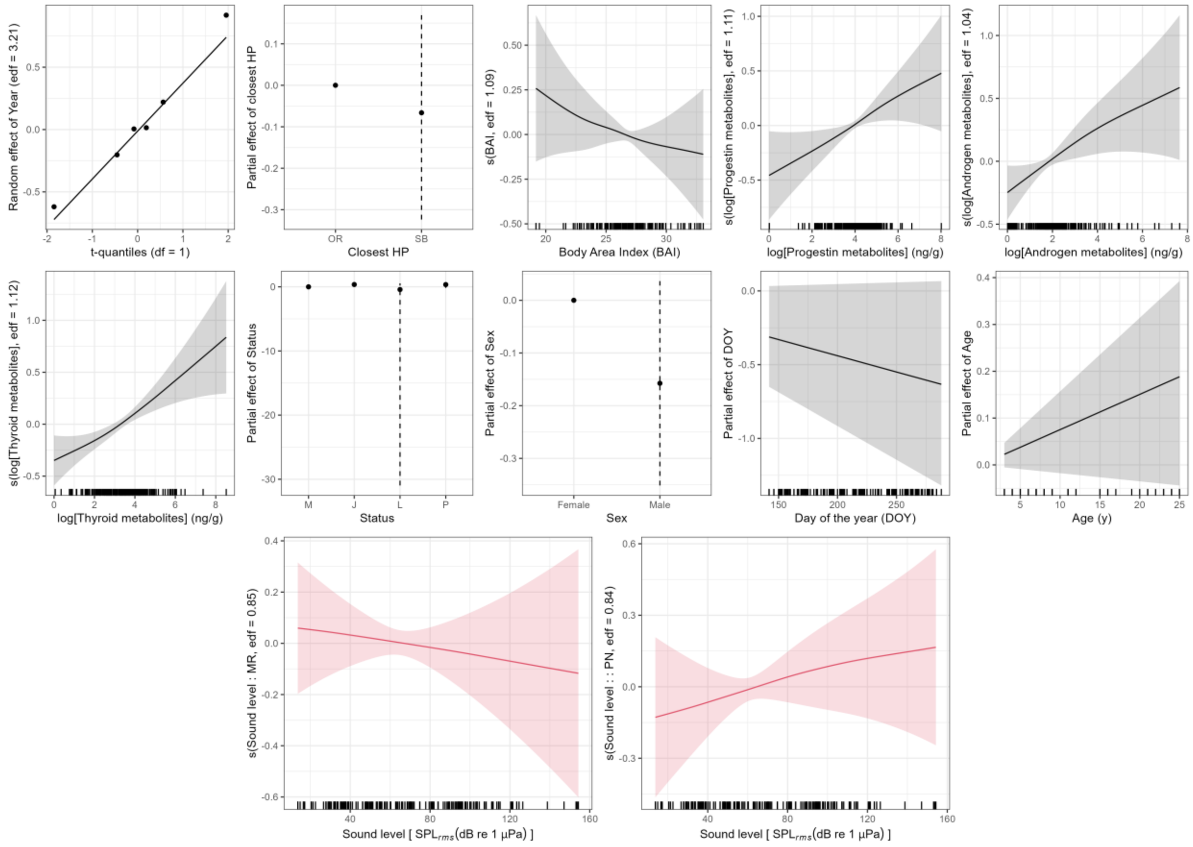

50 Hz - 1 kHz, 48 hr, median

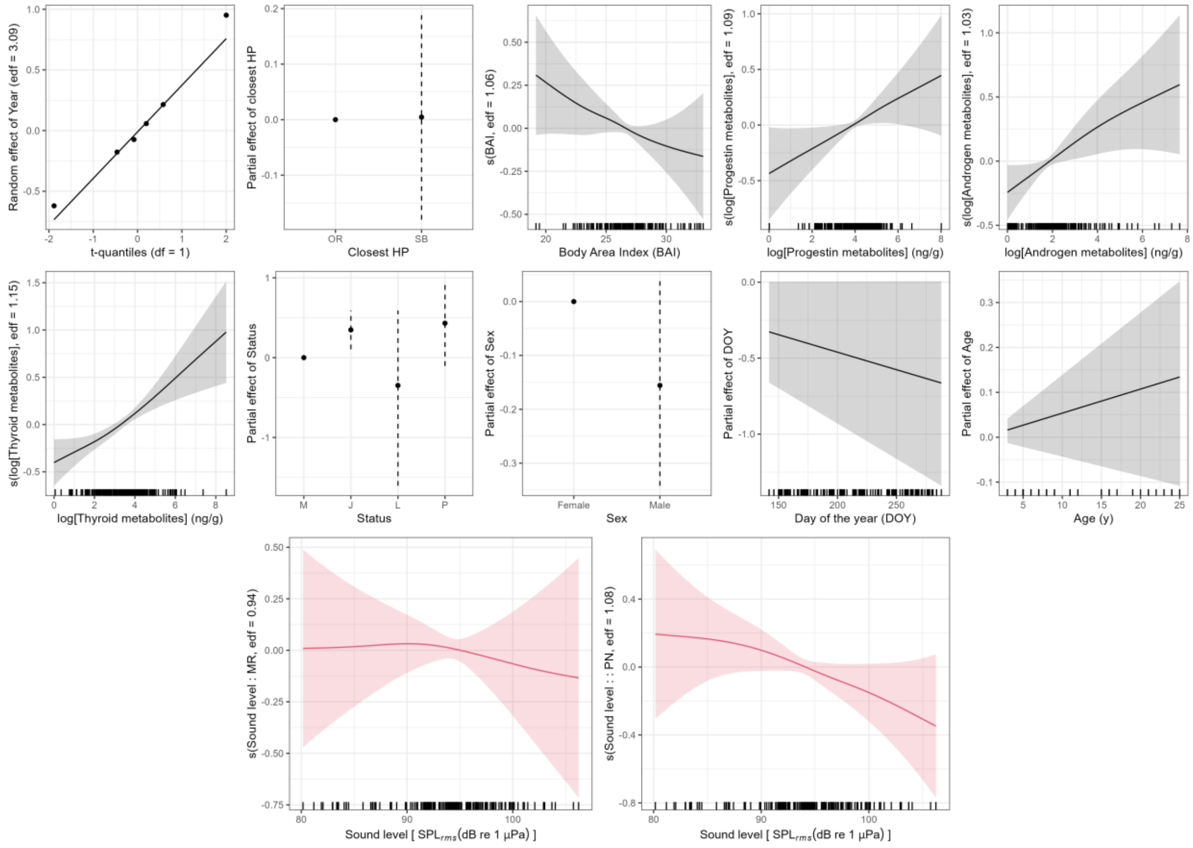

50 Hz - 1 kHz, 48 hr, 95th percentile

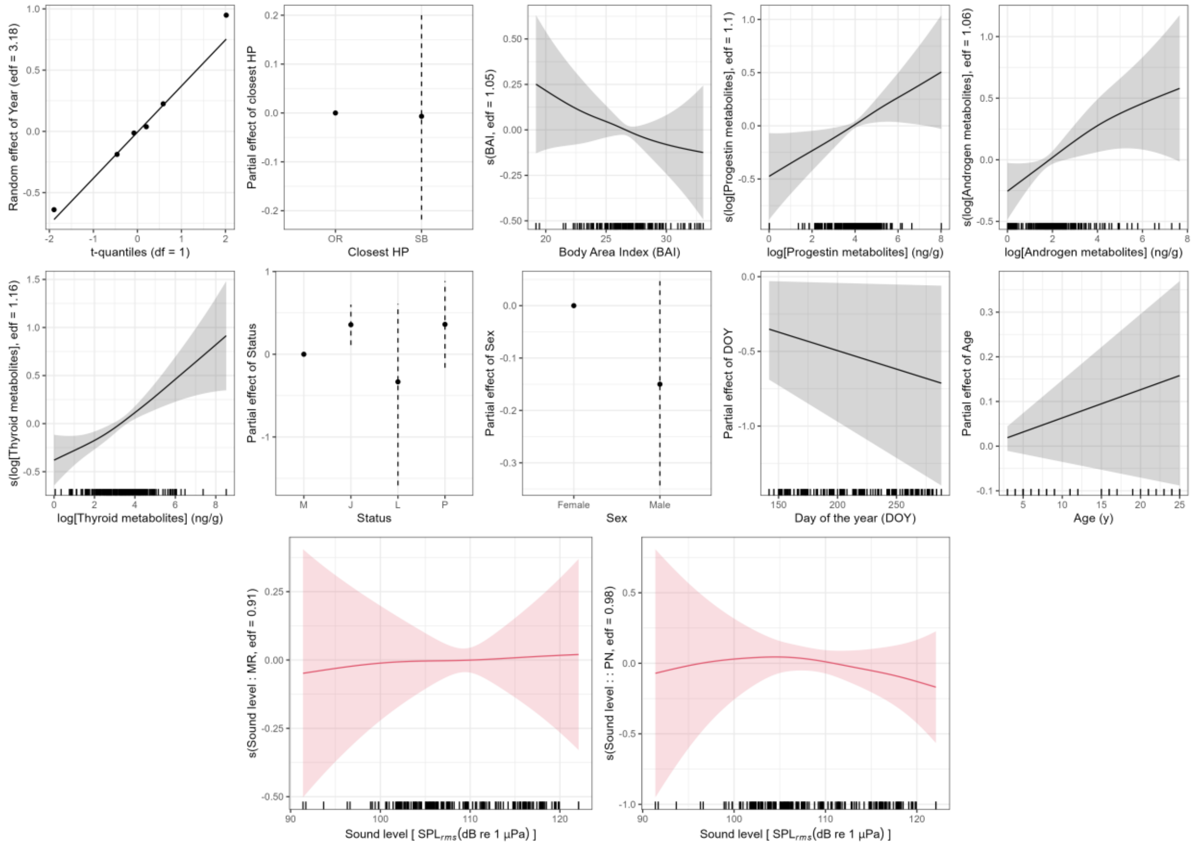

50 Hz - 1 kHz, 24 hr, variance

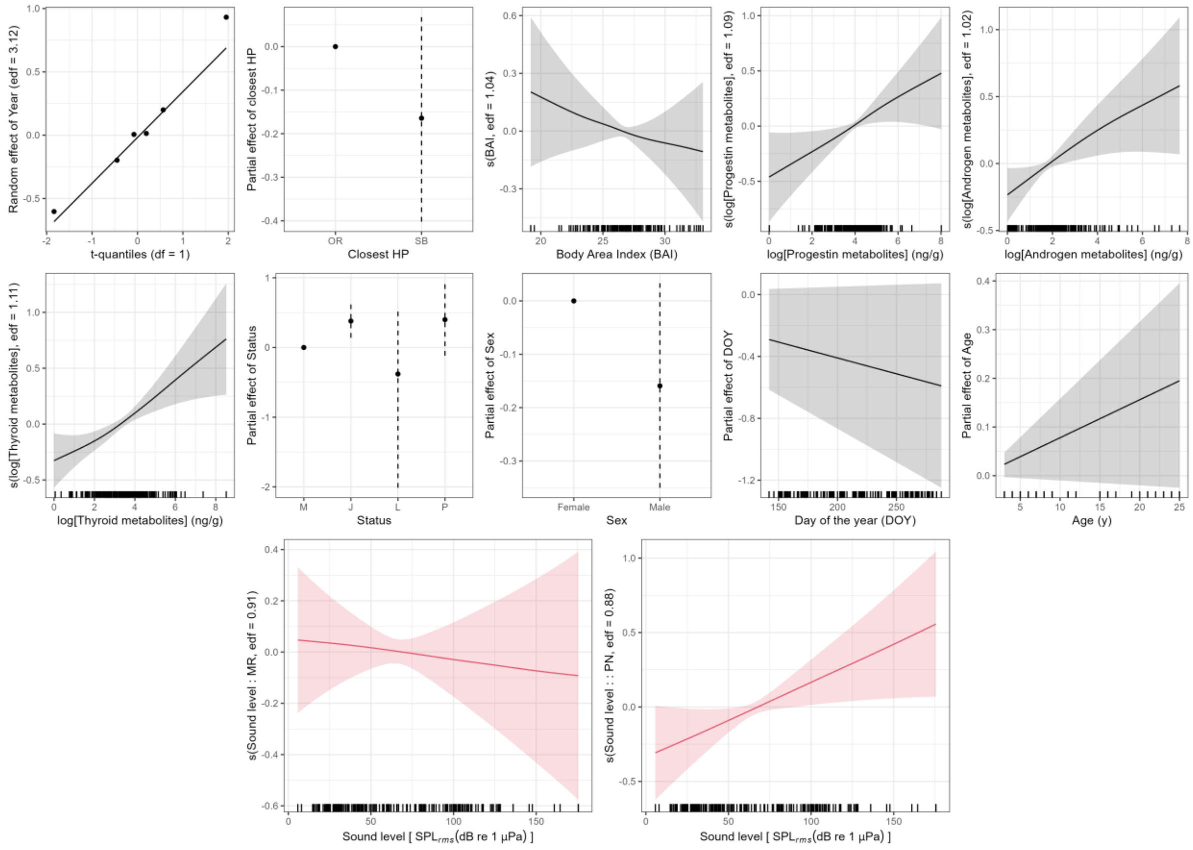

50 Hz - 1 kHz, 24 hr, median

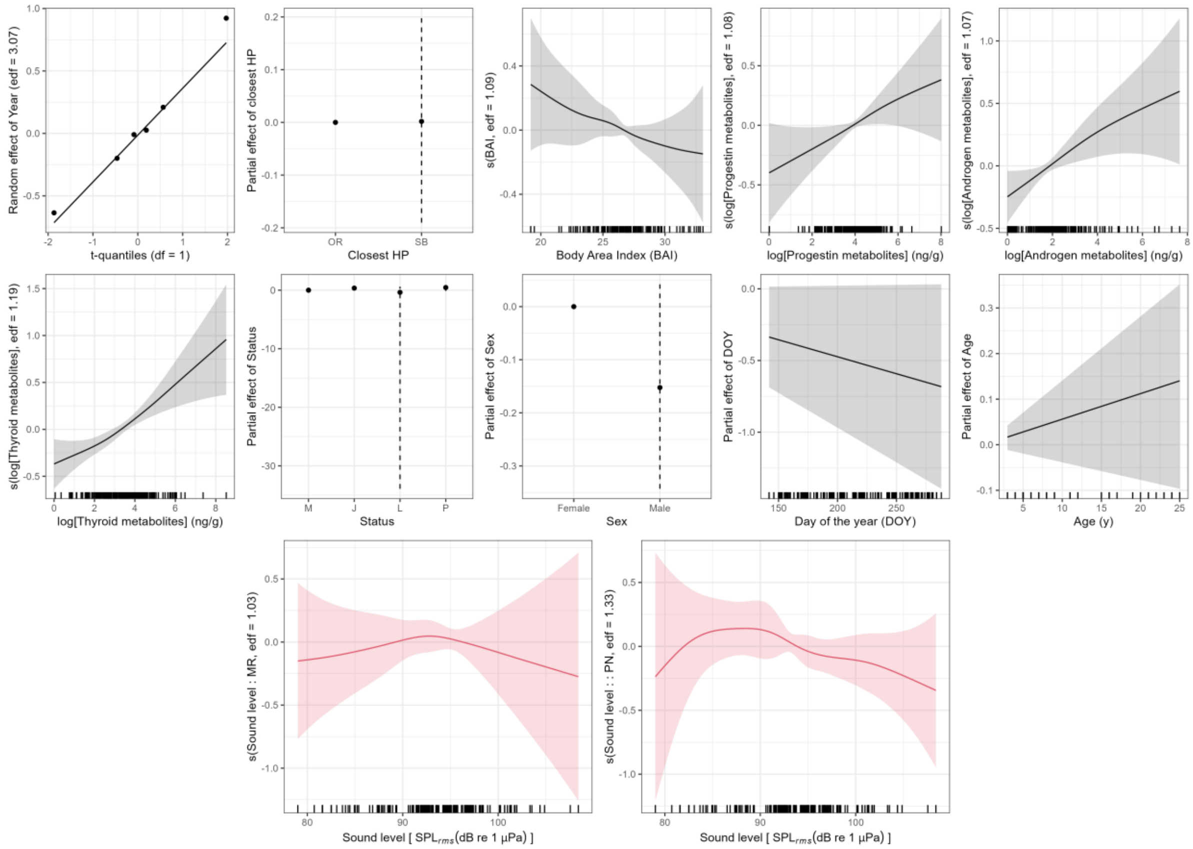

50 Hz - 1 kHz, 24 hr, 95th percentile

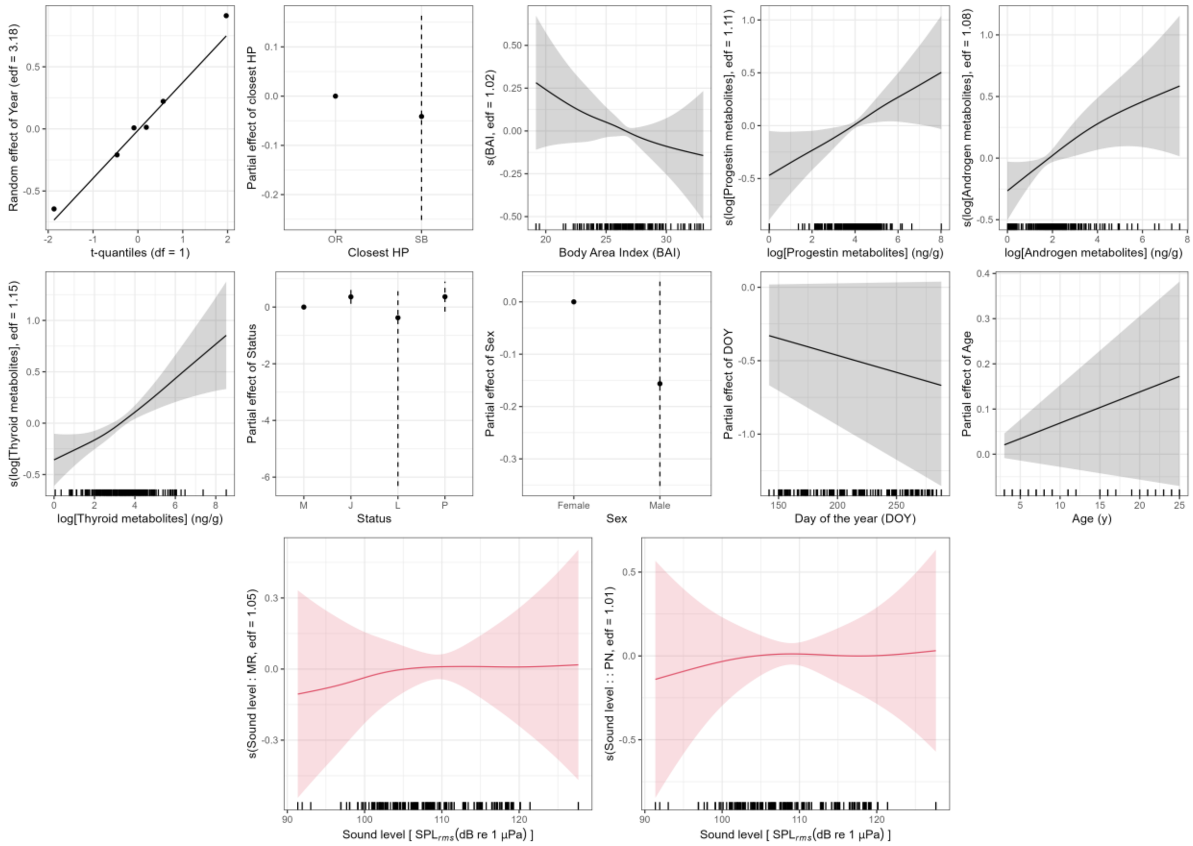

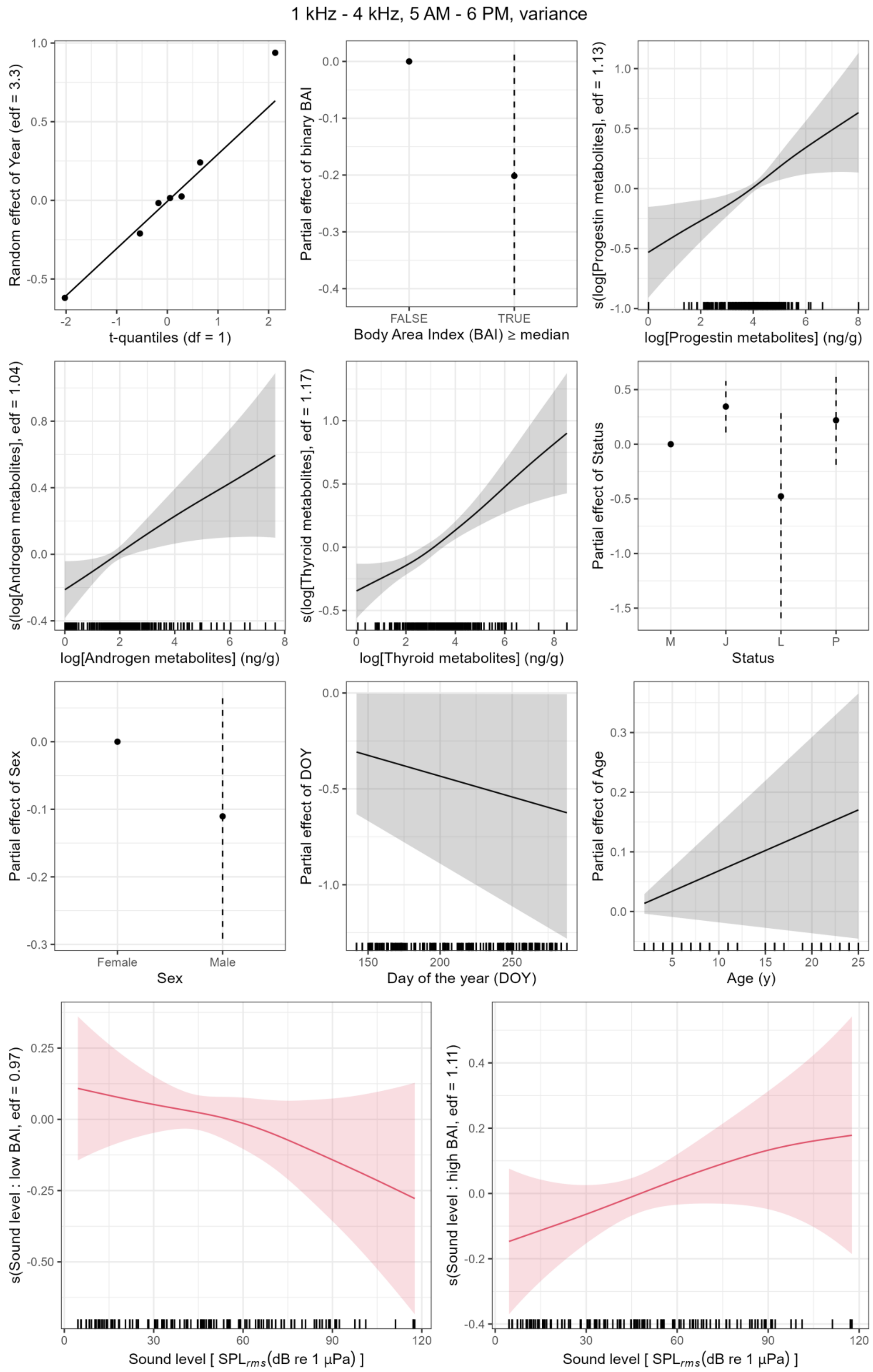
1 kHz - 4 kHz, 5 AM - 6 PM, variance
Random effect of Year (edf = 3.3)
t-quantiles (df = 1)
Partial effect of binary BAI
FALSE
TRUE
Body Area Index (BAI) ≥ median
s(log[Progestin metabolites], edf = 1.13)
log[Progestin metabolites] (ng/g)
s(log[Androgen metabolites], edf = 1.04)
log[Androgen metabolites] (ng/g)
s(log[Thyroid metabolites], edf = 1.17)
log[Thyroid metabolites] (ng/g)
Partial effect of Status
M
J
L
P
Status
Partial effect of Sex
Female
Male
Sex
Partial effect of DOY
Day of the year (DOY)
Partial effect of Age
Age (y)
s(Sound level : low BAI, edf = 0.97)
Sound level [SPL$_{rms}$(dB re 1 µPa)]
s(Sound level : high BAI, edf = 1.11)
Sound level [SPL$_{rms}$(dB re 1 µPa)]

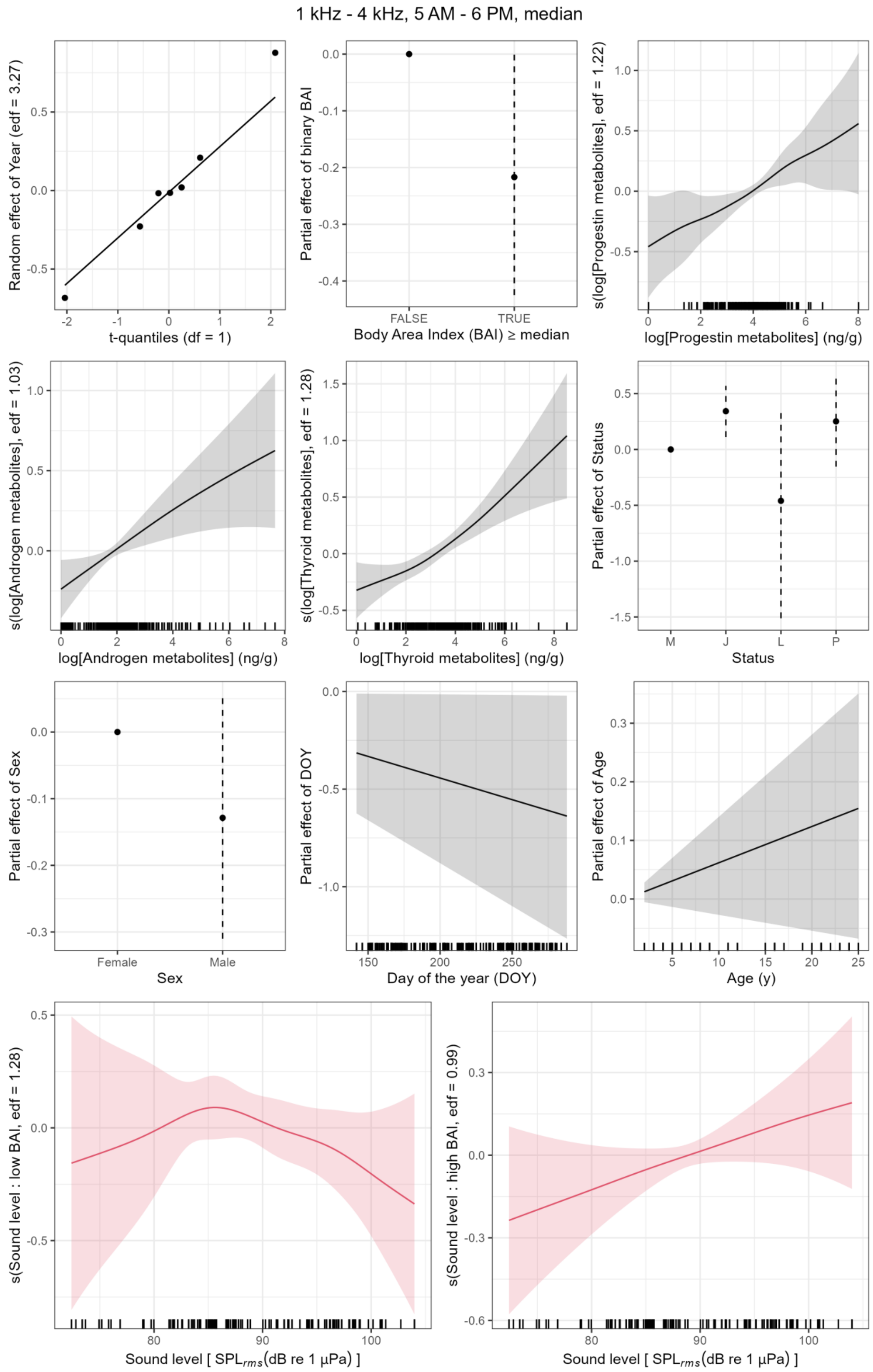
1 kHz - 4 kHz, 5 AM - 6 PM, median
Random effect of Year (edf = 3.27)
t-quantiles (df = 1)
Partial effect of binary BAI
FALSE
TRUE
Body Area Index (BAI) ≥ median
s(log[Progestin metabolites], edf = 1.22)
log[Progestin metabolites] (ng/g)
s(log[Androgen metabolites], edf = 1.03)
log[Androgen metabolites] (ng/g)
s(log[Thyroid metabolites], edf = 1.28)
log[Thyroid metabolites] (ng/g)
Partial effect of Status
M
J
L
P
Status
Partial effect of Sex
Female
Male
Sex
Partial effect of DOY
Day of the year (DOY)
Partial effect of Age
Age (y)
s(Sound level : low BAI, edf = 1.28)
Sound level [SPLrms(dB re 1 µPa)]
s(Sound level : high BAI, edf = 0.99)
Sound level [SPLrms(dB re 1 µPa)]

1 kHz - 4 kHz, 5 AM - 6 PM, 95th percentile

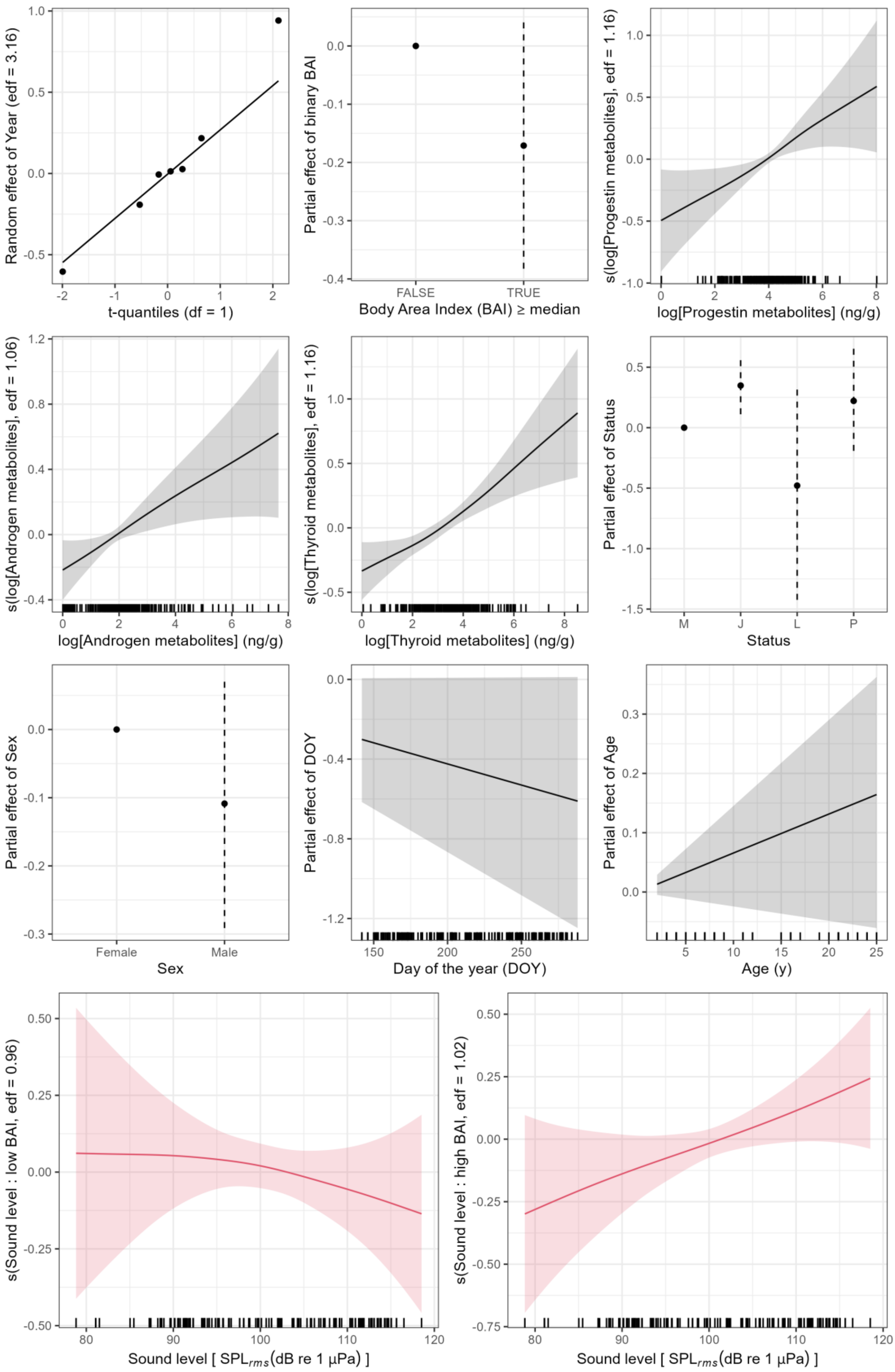

1 kHz - 4 kHz, 48 hr, variance

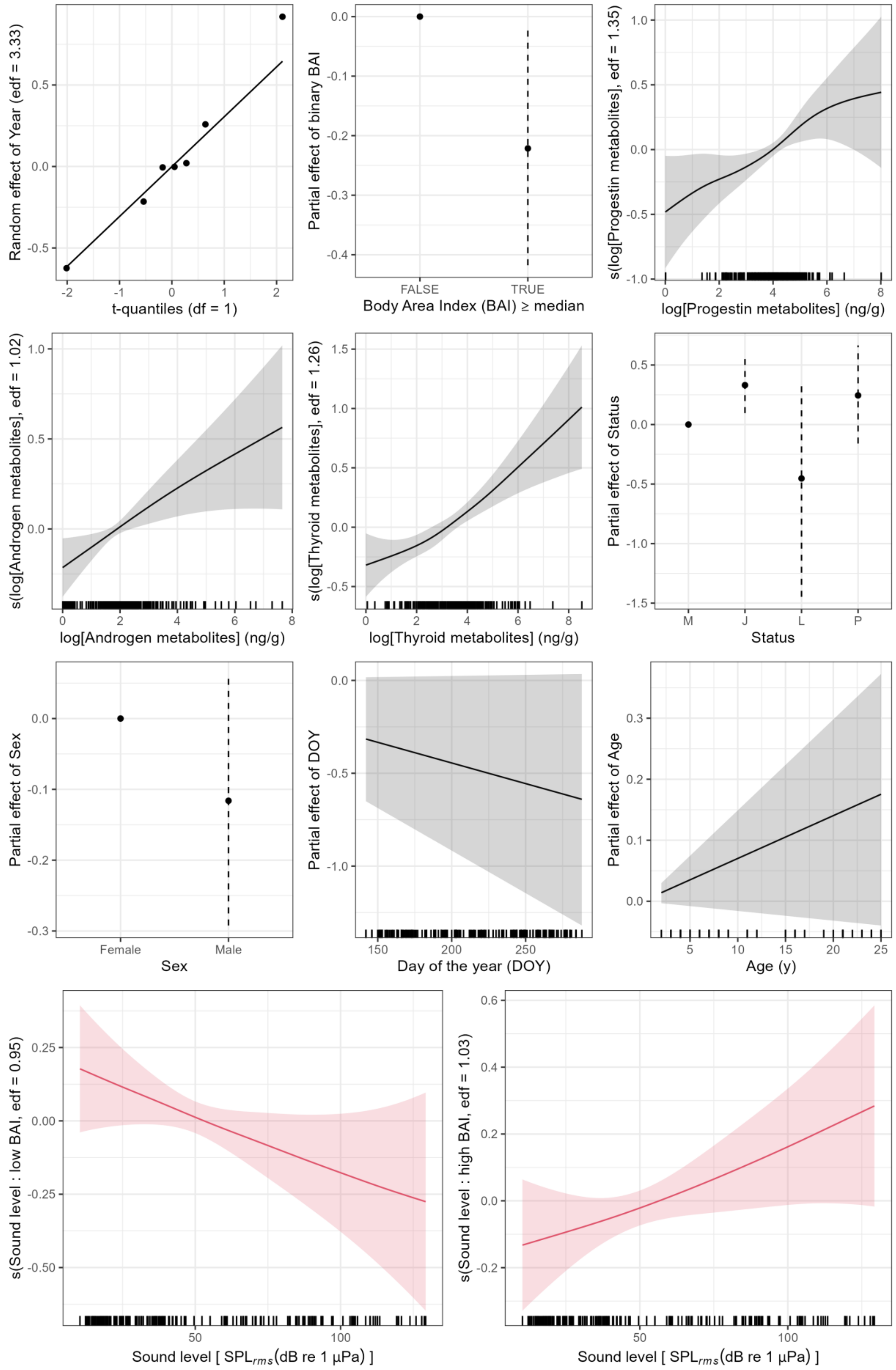

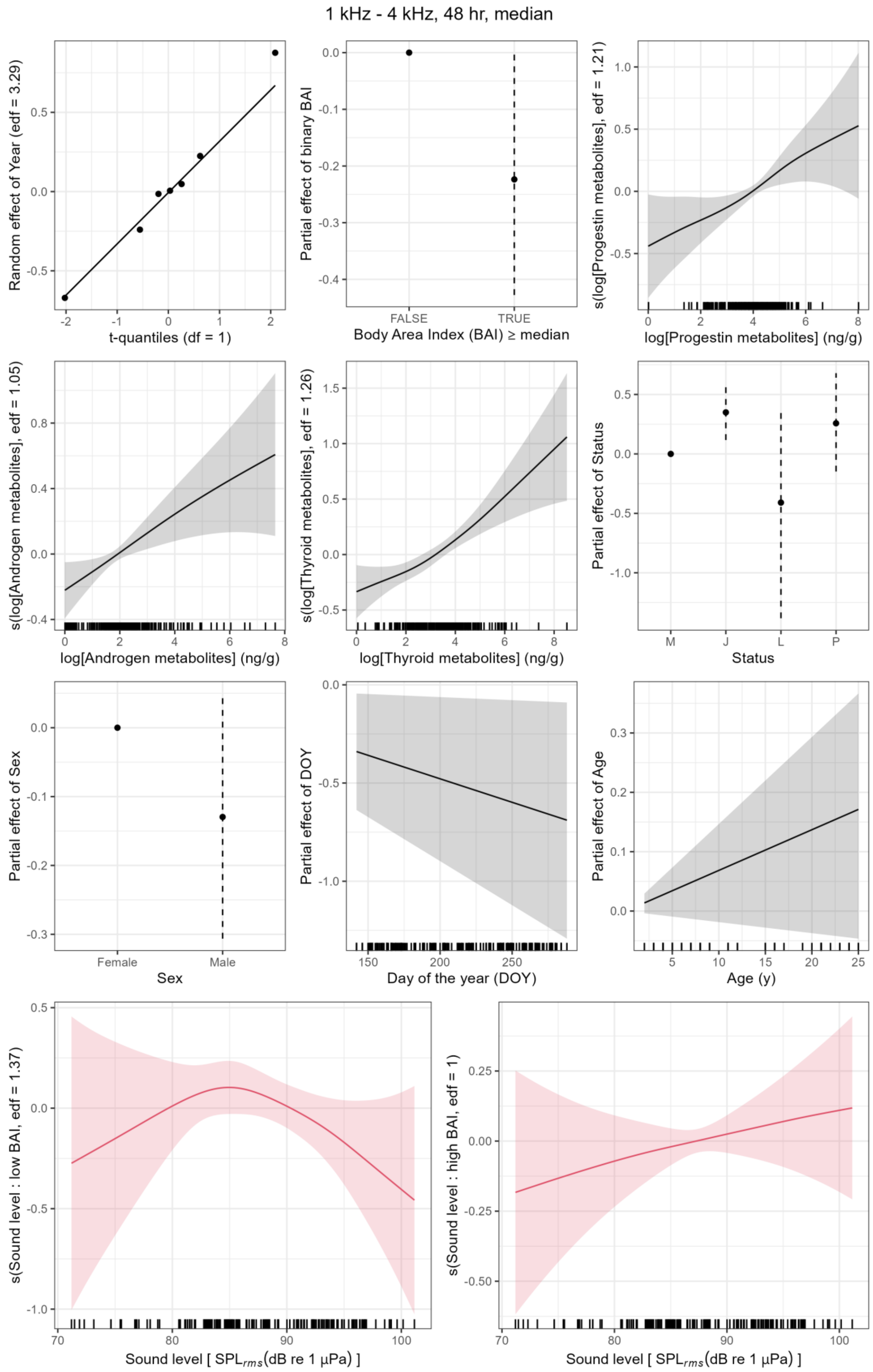
1 kHz - 4 kHz, 48 hr, median
Random effect of Year (edf = 3.29)
t-quantiles (df = 1)
Partial effect of binary BAI
FALSE
TRUE
Body Area Index (BAI) ≥ median
s(log[Progestin metabolites], edf = 1.21)
log[Progestin metabolites] (ng/g)
s(log[Androgen metabolites], edf = 1.05)
log[Androgen metabolites] (ng/g)
s(log[Thyroid metabolites], edf = 1.26)
log[Thyroid metabolites] (ng/g)
Partial effect of Status
M
J
L
P
Status
Partial effect of Sex
Female
Male
Sex
Partial effect of DOY
Day of the year (DOY)
Partial effect of Age
Age (y)
s(Sound level : low BAI, edf = 1.37)
Sound level [SPL$_{rms}$(dB re 1 µPa)]
s(Sound level : high BAI, edf = 1)
Sound level [SPL$_{rms}$(dB re 1 µPa)]

1 kHz - 4 kHz, 48 hr, 95th percentile

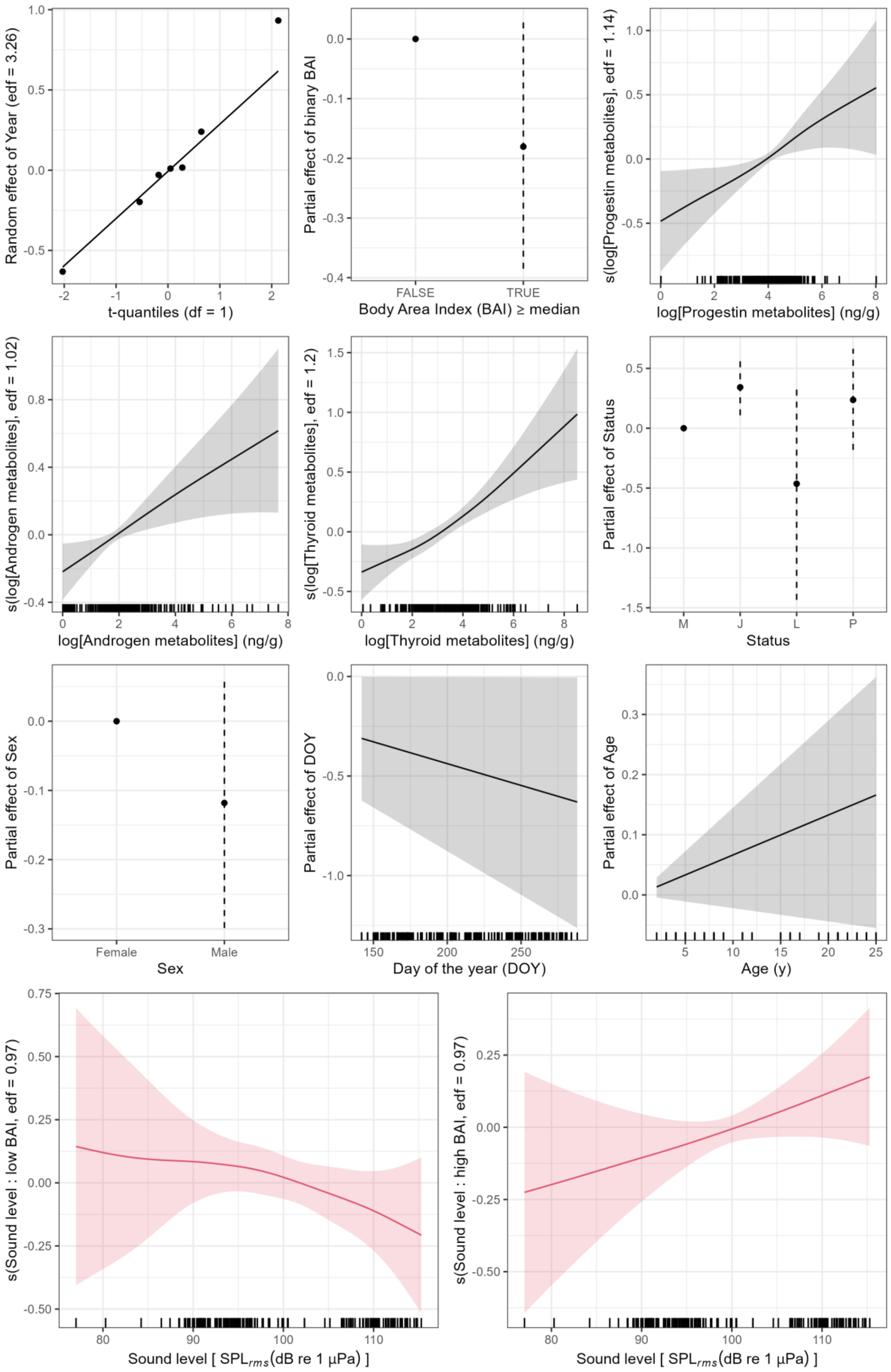

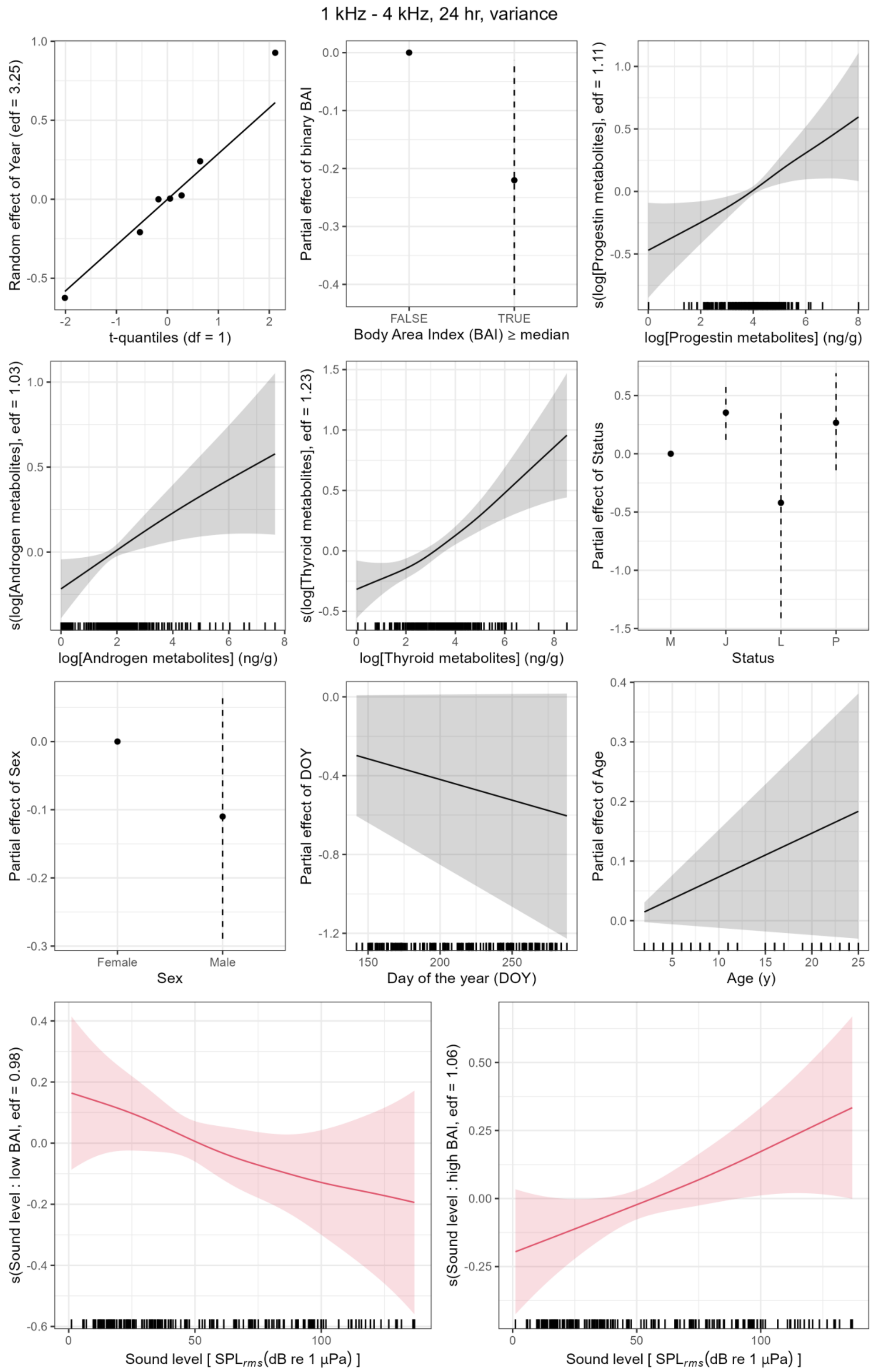
1 kHz - 4 kHz, 24 hr, variance
Random effect of Year (edf = 3.25)
t-quantiles (df = 1)
Partial effect of binary BAI
FALSE
TRUE
Body Area Index (BAI) ≥ median
s(log[Progestin metabolites], edf = 1.11)
log[Progestin metabolites] (ng/g)
s(log[Androgen metabolites], edf = 1.03)
log[Androgen metabolites] (ng/g)
s(log[Thyroid metabolites], edf = 1.23)
log[Thyroid metabolites] (ng/g)
Partial effect of Status
M
J
L
P
Status
Partial effect of Sex
Female
Male
Sex
Partial effect of DOY
Day of the year (DOY)
Partial effect of Age
Age (y)
s(Sound level : low BAI, edf = 0.98)
Sound level [SPL$_{rms}$(dB re 1 µPa)]
s(Sound level : high BAI, edf = 1.06)
Sound level [SPL$_{rms}$(dB re 1 µPa)]

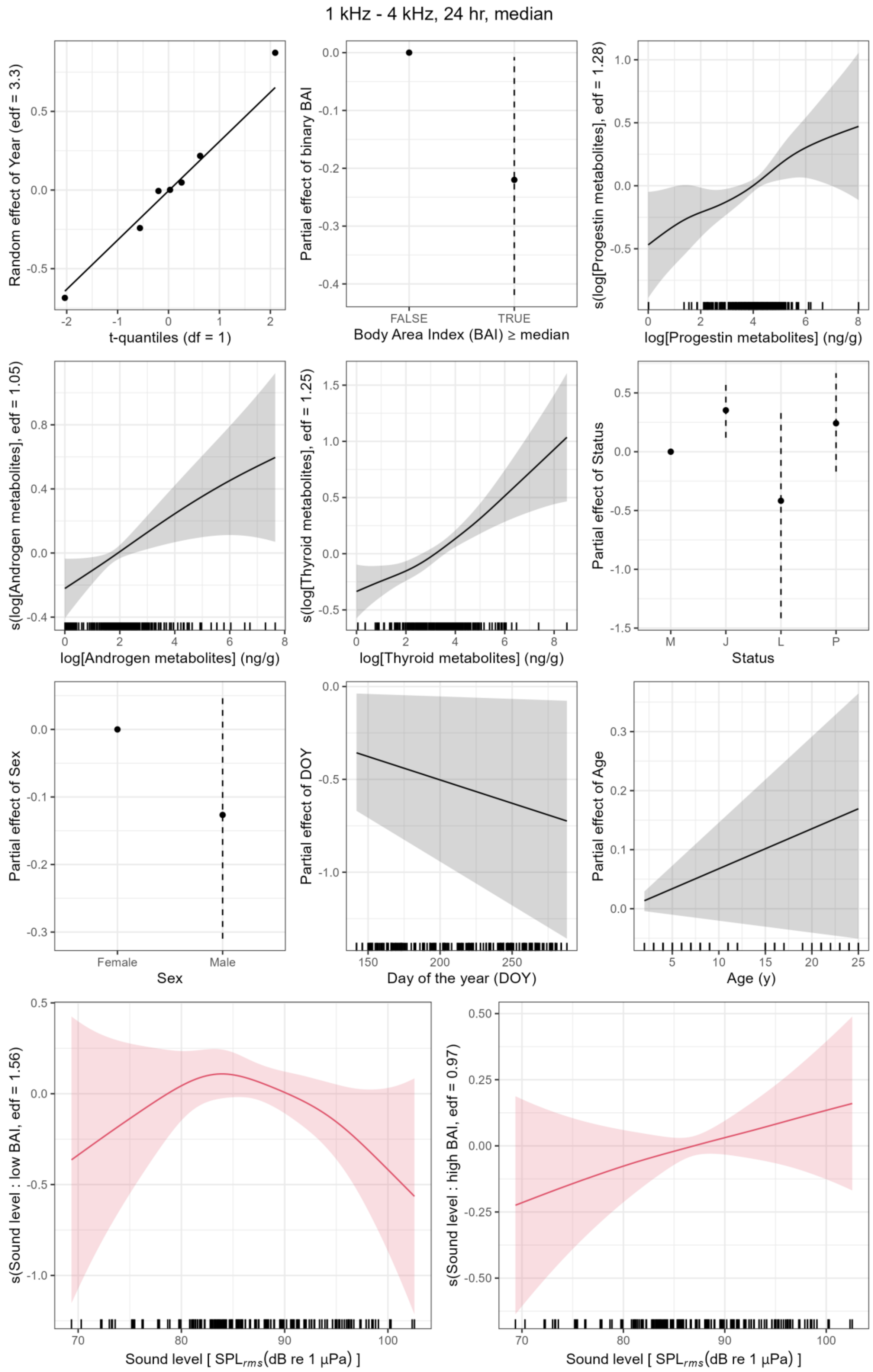

1 kHz - 4 kHz, 24 hr, median
Random effect of Year (edf = 3.3)
t-quantiles (df = 1)
Partial effect of binary BAI
FALSE
TRUE
Body Area Index (BAI) ≥ median
s(log[Progestin metabolites], edf = 1.28)
log[Progestin metabolites] (ng/g)
s(log[Androgen metabolites], edf = 1.05)
log[Androgen metabolites] (ng/g)
s(log[Thyroid metabolites], edf = 1.25)
log[Thyroid metabolites] (ng/g)
Partial effect of Status
M
J
L
P
Status
Partial effect of Sex
Female
Male
Sex
Partial effect of DOY
Day of the year (DOY)
Partial effect of Age
Age (y)
s(Sound level : low BAI, edf = 1.56)
Sound level [SPL$_{rms}$(dB re 1 µPa)]
s(Sound level : high BAI, edf = 0.97)
Sound level [SPL$_{rms}$(dB re 1 µPa)]

1 kHz - 4 kHz, 24 hr, 95th percentile

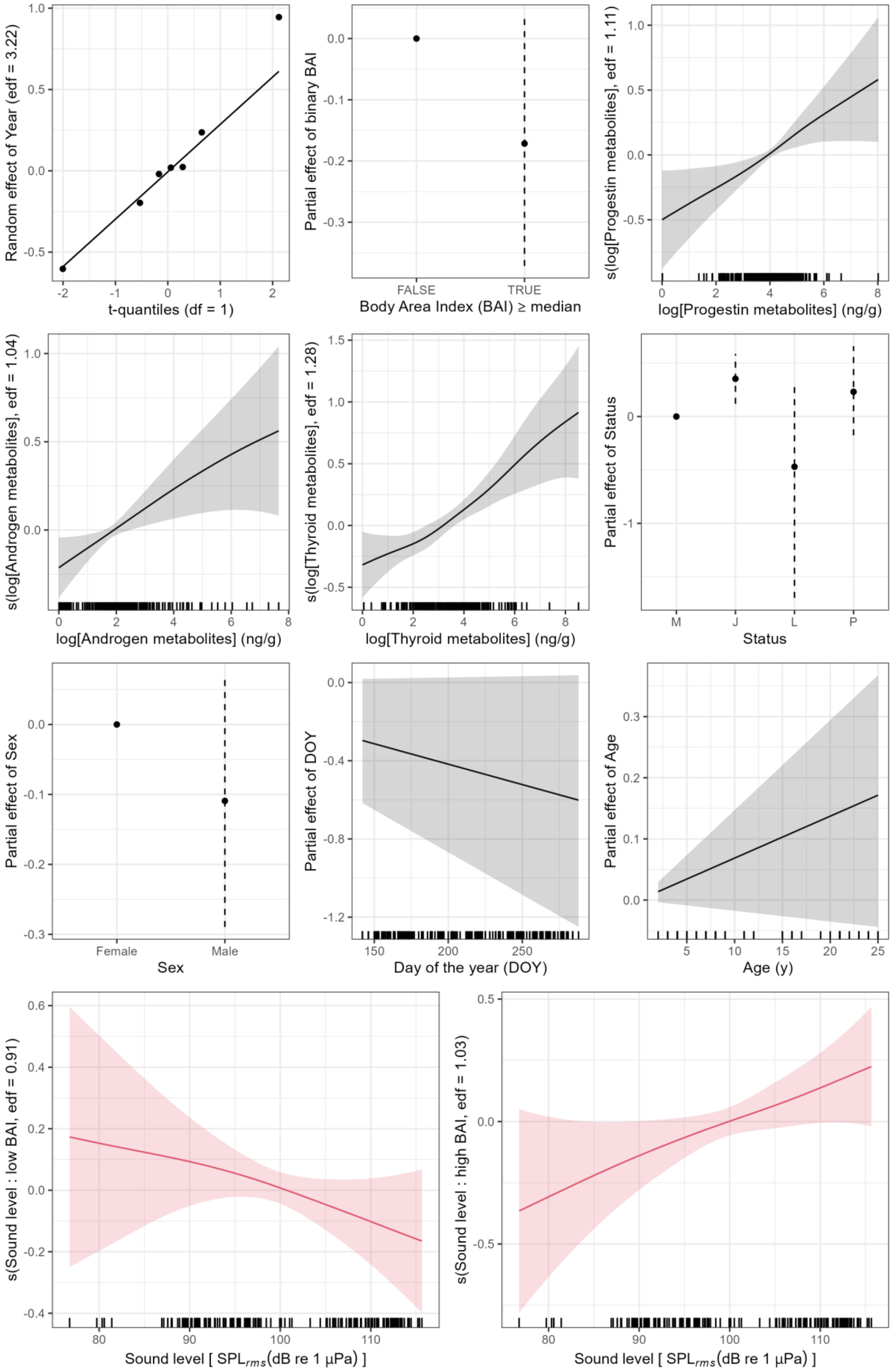

50 Hz - 1 kHz, 5 AM - 6 PM, variance

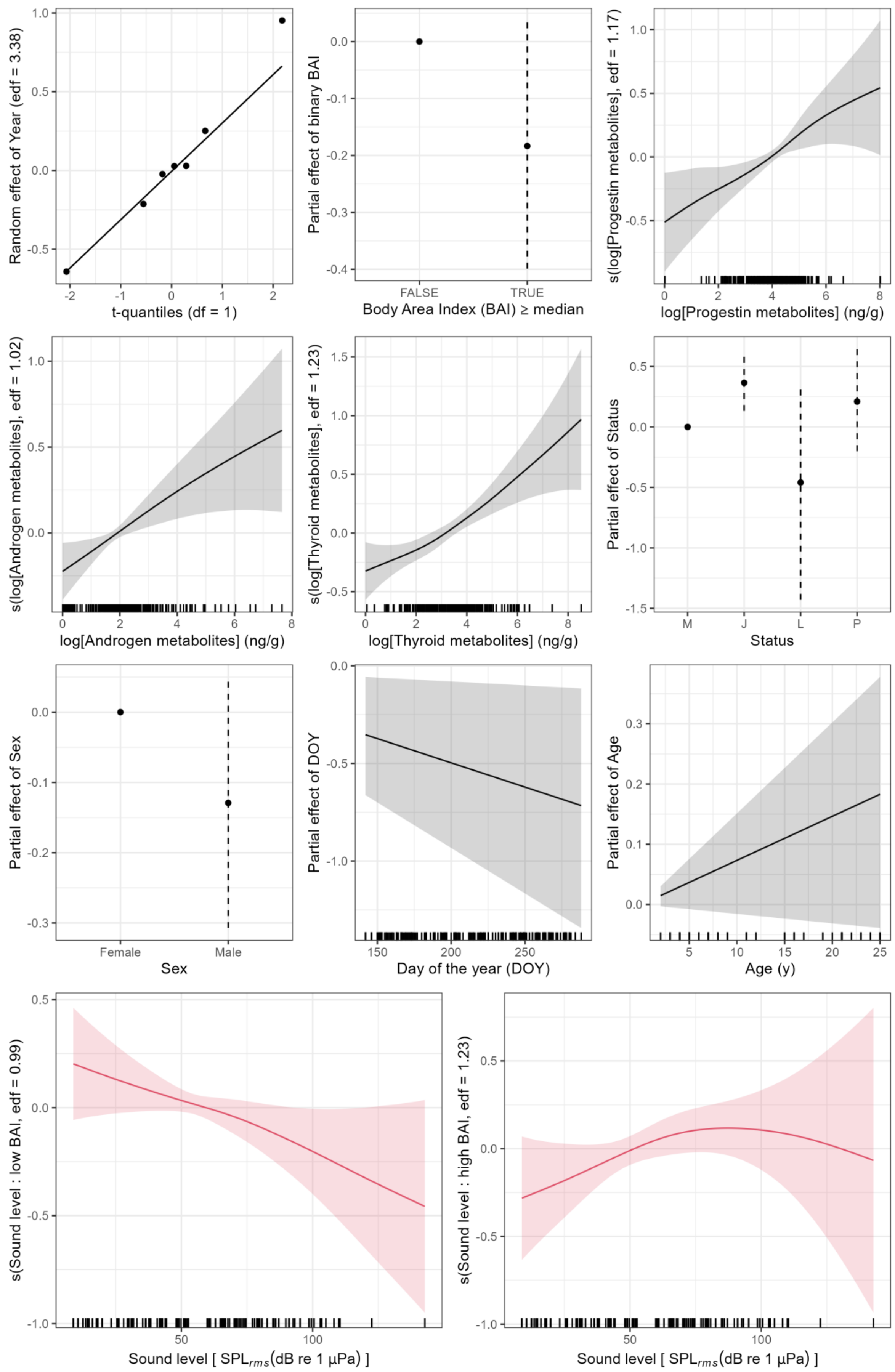

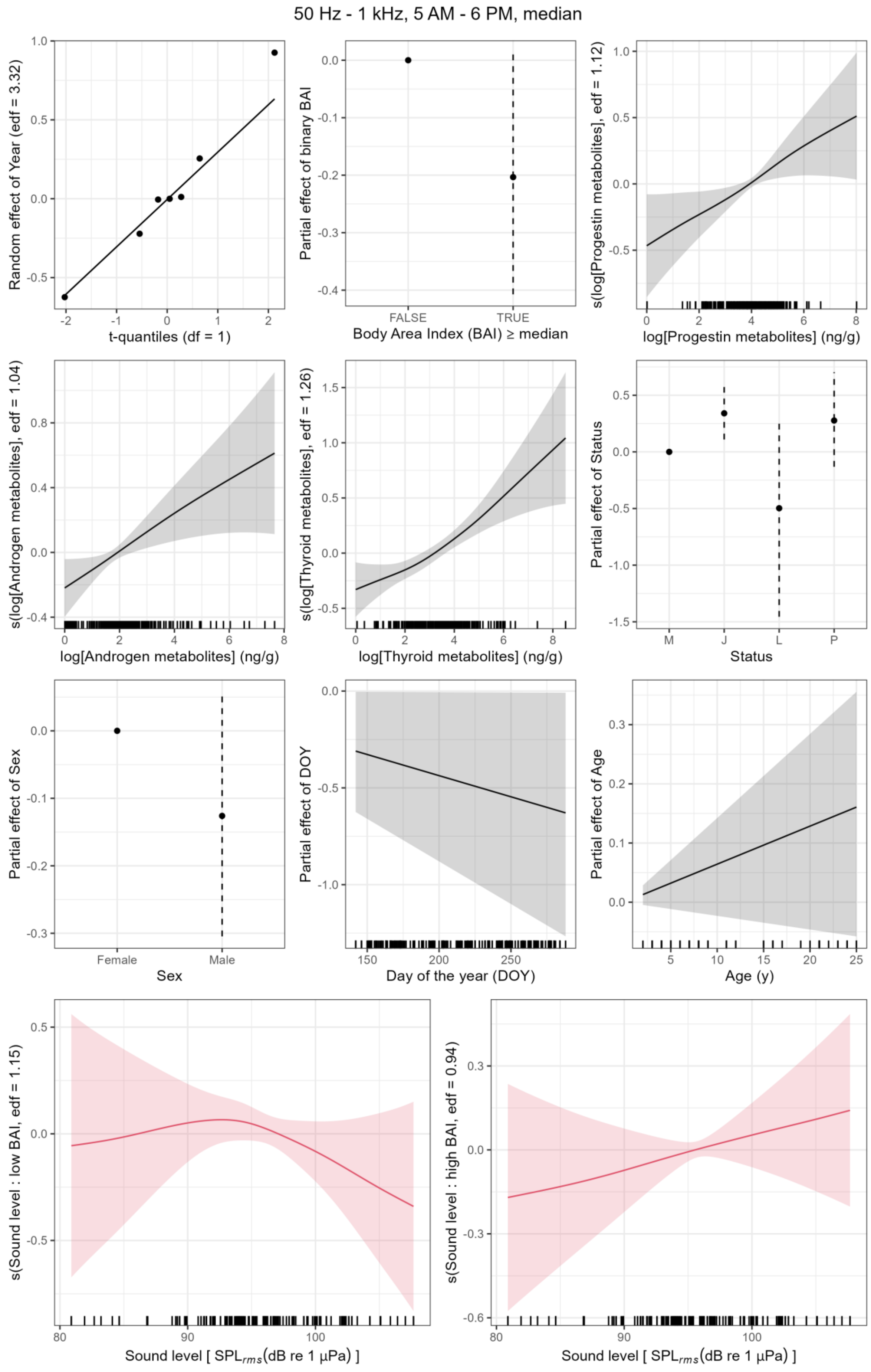
50 Hz - 1 kHz, 5 AM - 6 PM, median
Random effect of Year (edf = 3.32)
t-quantiles (df = 1)
Partial effect of binary BAI
FALSE
TRUE
Body Area Index (BAI) ≥ median
s(log[Progestin metabolites], edf = 1.12)
log[Progestin metabolites] (ng/g)
s(log[Androgen metabolites], edf = 1.04)
log[Androgen metabolites] (ng/g)
s(log[Thyroid metabolites], edf = 1.26)
log[Thyroid metabolites] (ng/g)
Partial effect of Status
M
J
L
P
Status
Partial effect of Sex
Female
Male
Sex
Partial effect of DOY
Day of the year (DOY)
Partial effect of Age
Age (y)
s(Sound level : low BAI, edf = 1.15)
Sound level [SPLrms(dB re 1 μPa)]
s(Sound level : high BAI, edf = 0.94)
Sound level [SPLrms(dB re 1 μPa)]

50 Hz - 1 kHz, 5 AM - 6 PM, 95th percentile

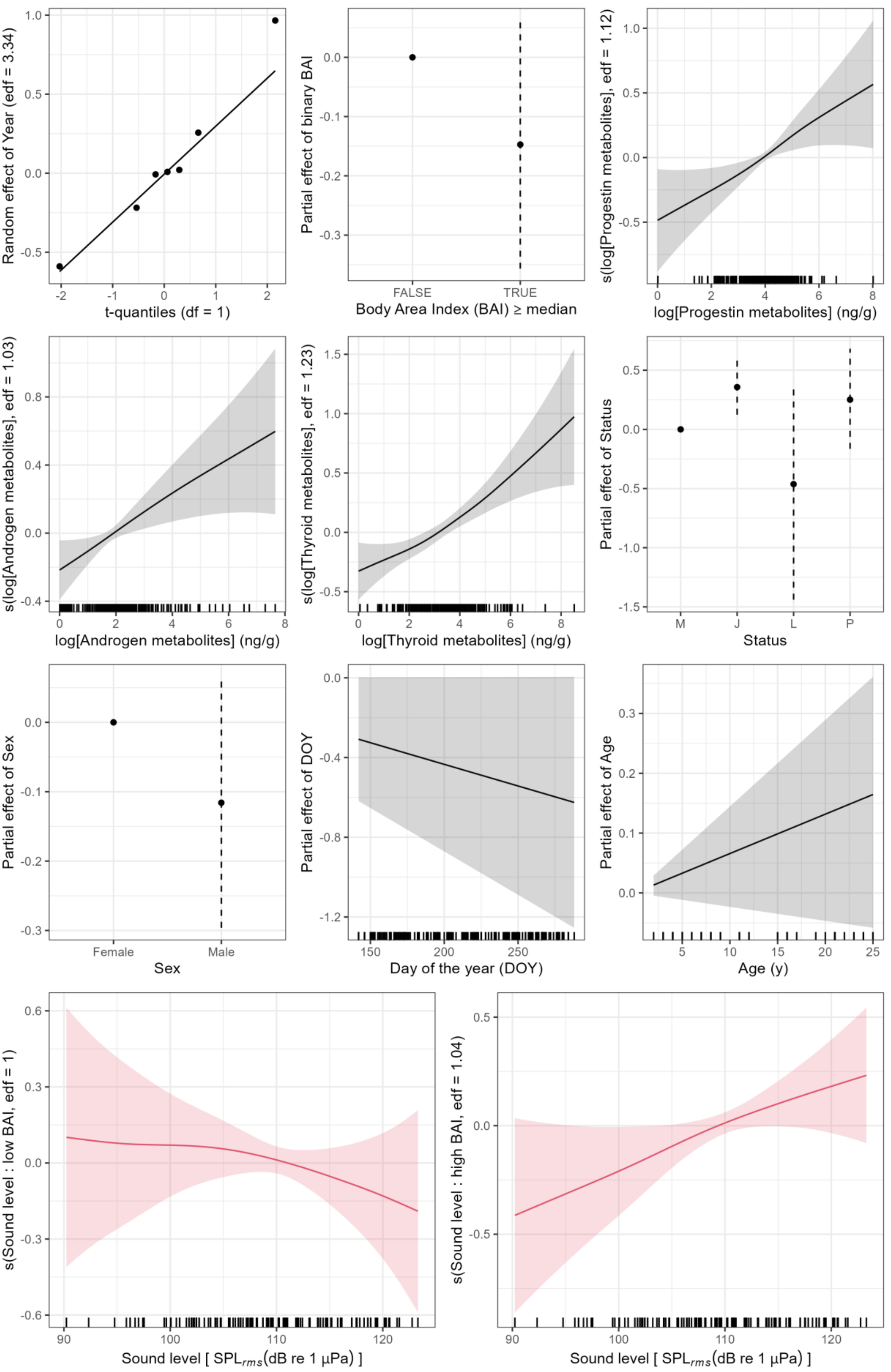

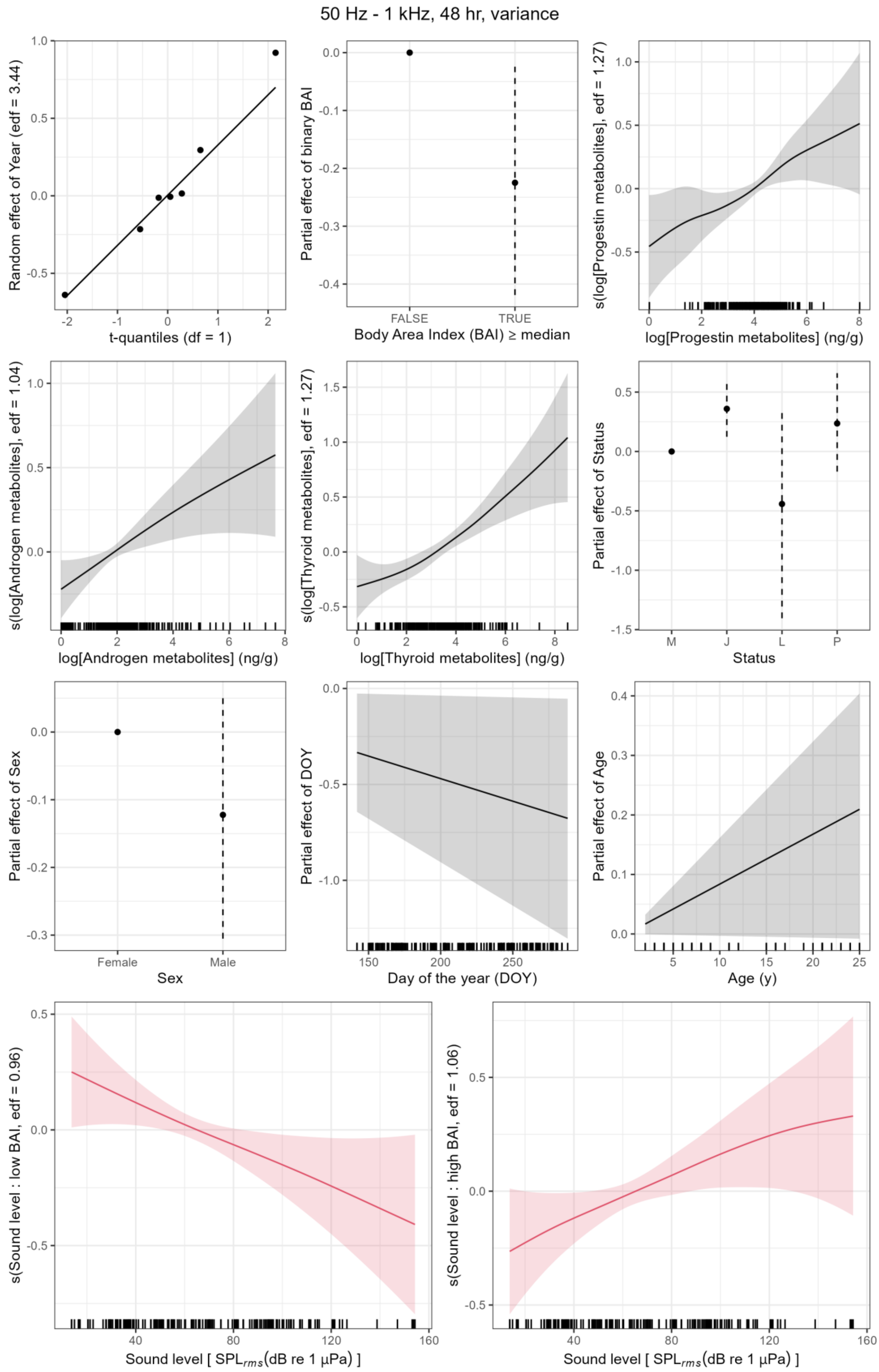
50 Hz - 1 kHz, 48 hr, variance
Random effect of Year (edf = 3.44)
t-quantiles (df = 1)
Partial effect of binary BAI
FALSE
TRUE
Body Area Index (BAI) ≥ median
s(log[Progestin metabolites], edf = 1.27)
log[Progestin metabolites] (ng/g)
s(log[Androgen metabolites], edf = 1.04)
log[Androgen metabolites] (ng/g)
s(log[Thyroid metabolites], edf = 1.27)
log[Thyroid metabolites] (ng/g)
Partial effect of Status
M
J
L
P
Status
Partial effect of Sex
Female
Male
Sex
Partial effect of DOY
Day of the year (DOY)
Partial effect of Age
Age (y)
s(Sound level : low BAI, edf = 0.96)
Sound level [SPL$_{rms}$(dB re 1 µPa)]
s(Sound level : high BAI, edf = 1.06)
Sound level [SPL$_{rms}$(dB re 1 µPa)]

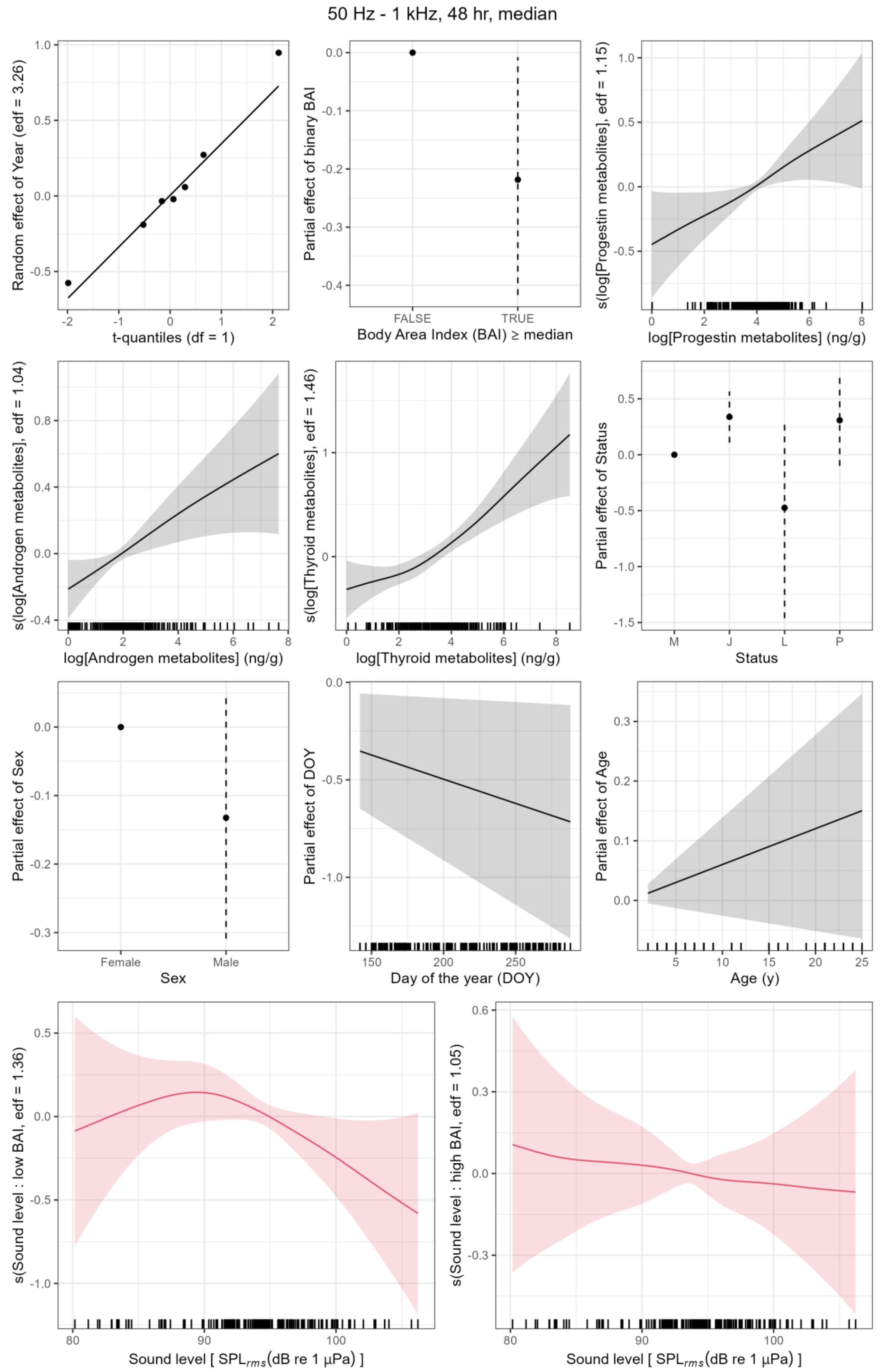

50 Hz - 1 kHz, 48 hr, median
Random effect of Year (edf = 3.26)
t-quantiles (df = 1)
Partial effect of binary BAI
FALSE
TRUE
Body Area Index (BAI) ≥ median
s(log[Progestin metabolites], edf = 1.15)
log[Progestin metabolites] (ng/g)
s(log[Androgen metabolites], edf = 1.04)
log[Androgen metabolites] (ng/g)
s(log[Thyroid metabolites], edf = 1.46)
log[Thyroid metabolites] (ng/g)
Partial effect of Status
M
J
L
P
Status
Partial effect of Sex
Female
Male
Sex
Partial effect of DOY
Day of the year (DOY)
Partial effect of Age
Age (y)
s(Sound level : low BAI, edf = 1.36)
Sound level [SPL$_{rms}$(dB re 1 µPa)]
s(Sound level : high BAI, edf = 1.05)
Sound level [SPL$_{rms}$(dB re 1 µPa)]

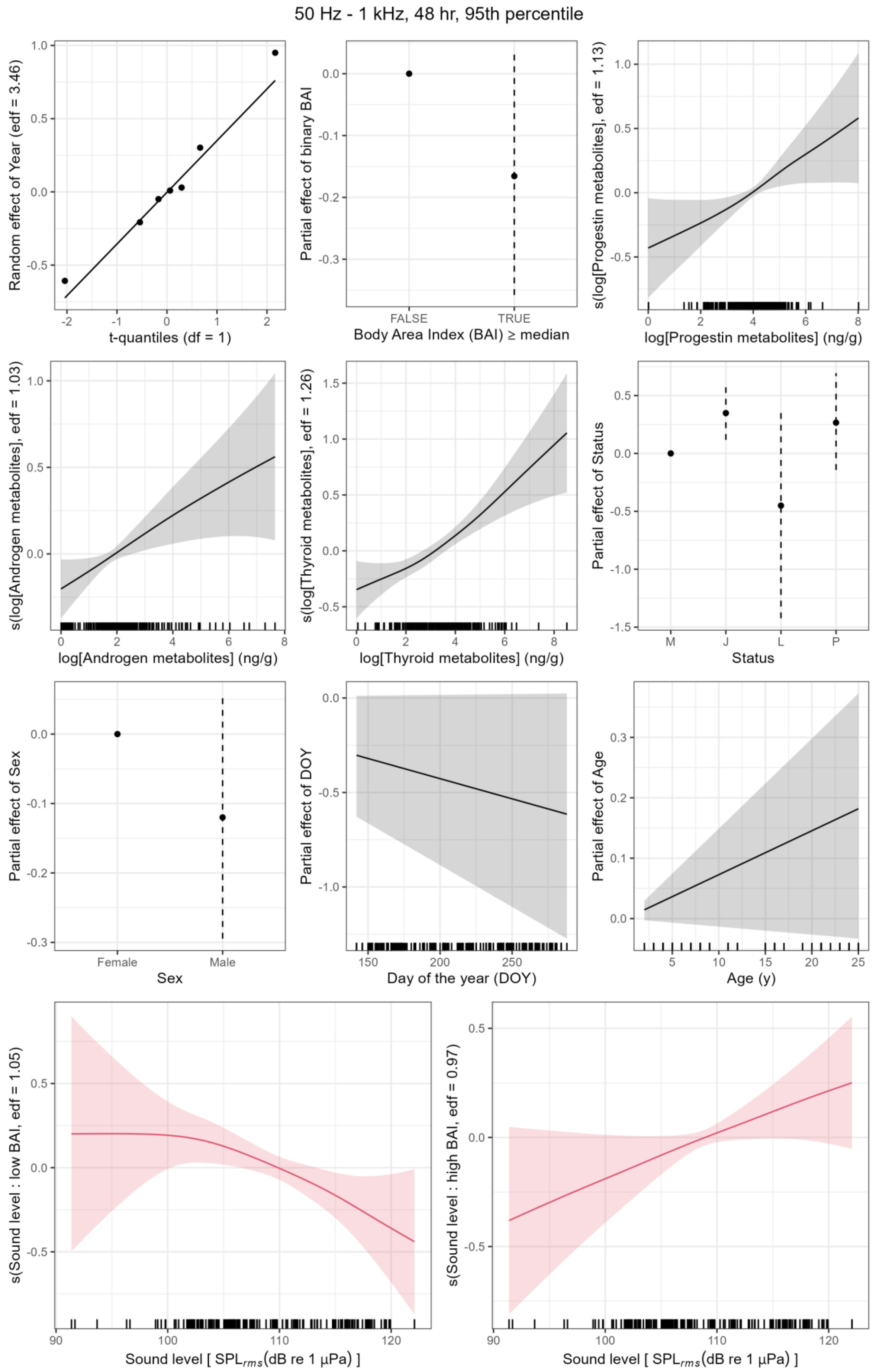
50 Hz - 1 kHz, 48 hr, 95th percentile
Random effect of Year (edf = 3.46)
t-quantiles (df = 1)
Partial effect of binary BAI
FALSE
TRUE
Body Area Index (BAI) ≥ median
s(log[Progestin metabolites], edf = 1.13)
log[Progestin metabolites] (ng/g)
s(log[Androgen metabolites], edf = 1.03)
log[Androgen metabolites] (ng/g)
s(log[Thyroid metabolites], edf = 1.26)
log[Thyroid metabolites] (ng/g)
Partial effect of Status
M
J
L
P
Status
Partial effect of Sex
Female
Male
Sex
Partial effect of DOY
Day of the year (DOY)
Partial effect of Age
Age (y)
s(Sound level : low BAI, edf = 1.05)
Sound level [SPLrms(dB re 1 µPa)]
s(Sound level : high BAI, edf = 0.97)
Sound level [SPLrms(dB re 1 µPa)]

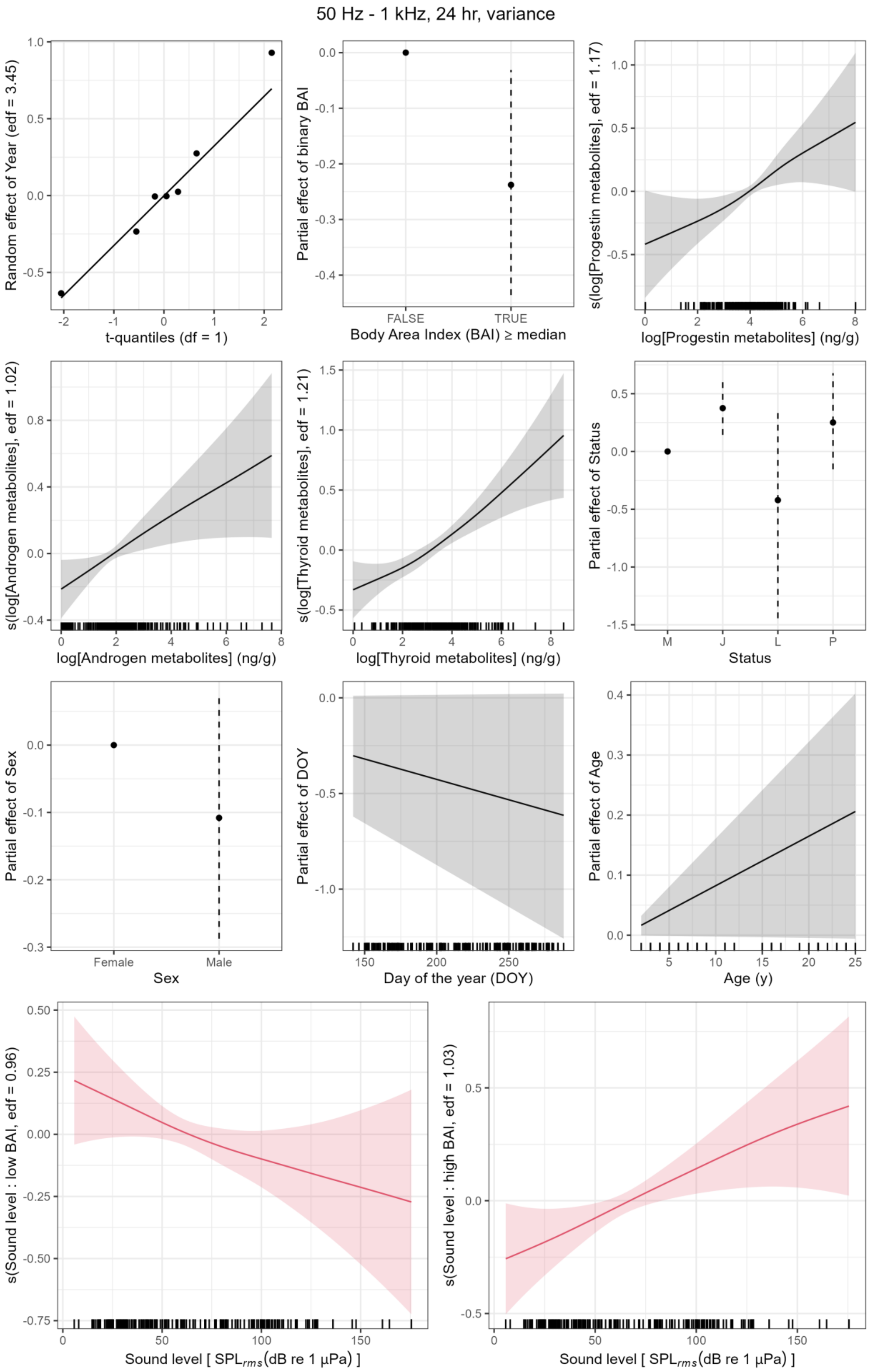

50 Hz - 1 kHz, 24 hr, variance
Random effect of Year (edf = 3.45)
t-quantiles (df = 1)
Partial effect of binary BAI
FALSE
TRUE
Body Area Index (BAI) ≥ median
s(log[Progestin metabolites], edf = 1.17)
log[Progestin metabolites] (ng/g)
s(log[Androgen metabolites], edf = 1.02)
log[Androgen metabolites] (ng/g)
s(log[Thyroid metabolites], edf = 1.21)
log[Thyroid metabolites] (ng/g)
Partial effect of Status
M
J
L
P
Status
Partial effect of Sex
Female
Male
Sex
Partial effect of DOY
Day of the year (DOY)
Partial effect of Age
Age (y)
s(Sound level : low BAI, edf = 0.96)
Sound level [SPL_{rms}(dB re 1 μPa)]
s(Sound level : high BAI, edf = 1.03)
Sound level [SPL_{rms}(dB re 1 μPa)]

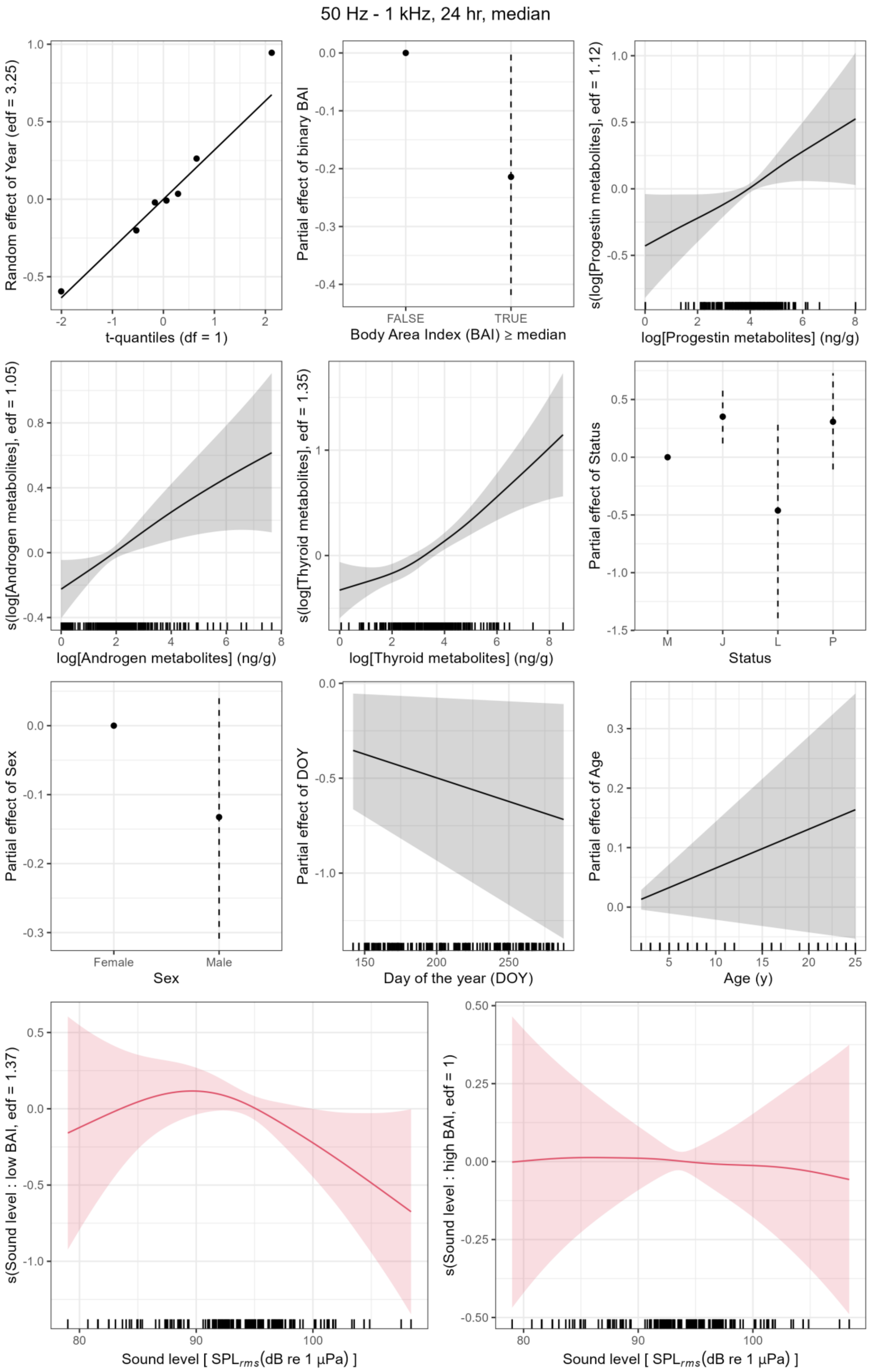
50 Hz - 1 kHz, 24 hr, median
Random effect of Year (edf = 3.25)
t-quantiles (df = 1)
Partial effect of binary BAI
FALSE
TRUE
Body Area Index (BAI) ≥ median
s(log[Progestin metabolites], edf = 1.12)
log[Progestin metabolites] (ng/g)
s(log[Androgen metabolites], edf = 1.05)
log[Androgen metabolites] (ng/g)
s(log[Thyroid metabolites], edf = 1.35)
log[Thyroid metabolites] (ng/g)
Partial effect of Status
M
J
L
P
Status
Partial effect of Sex
Female
Male
Sex
Partial effect of DOY
Day of the year (DOY)
Partial effect of Age
Age (y)
s(Sound level : low BAI, edf = 1.37)
Sound level [SPL$_{rms}$(dB re 1 μPa)]
s(Sound level : high BAI, edf = 1)
Sound level [SPL$_{rms}$(dB re 1 μPa)]

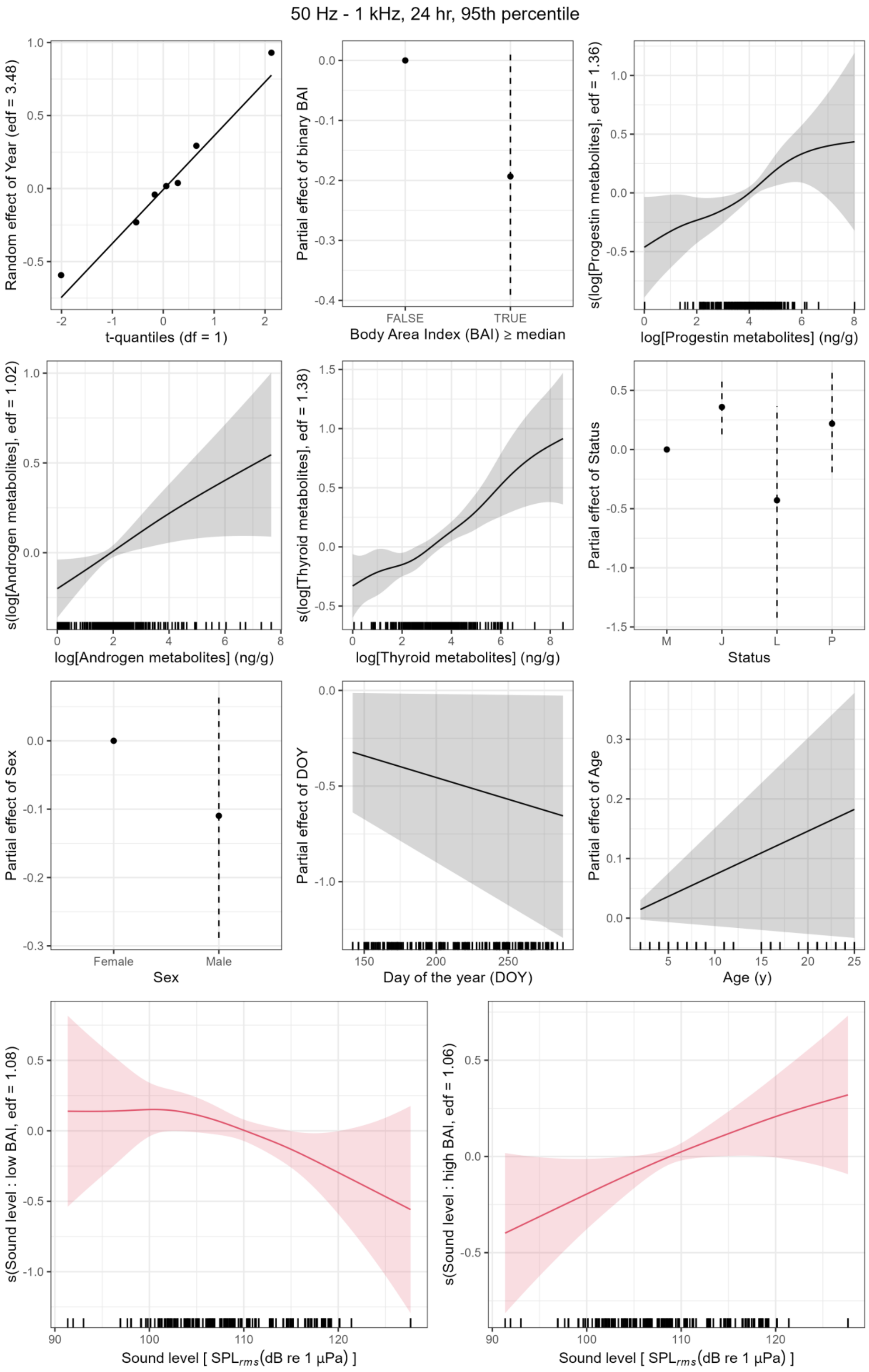
50 Hz - 1 kHz, 24 hr, 95th percentile
Random effect of Year (edf = 3.48)
t-quantiles (df = 1)
Partial effect of binary BAI
FALSE
TRUE
Body Area Index (BAI) ≥ median
s(log[Progestin metabolites], edf = 1.36)
log[Progestin metabolites] (ng/g)
s(log[Androgen metabolites], edf = 1.02)
log[Androgen metabolites] (ng/g)
s(log[Thyroid metabolites], edf = 1.38)
log[Thyroid metabolites] (ng/g)
Partial effect of Status
M
J
L
P
Status
Partial effect of Sex
Female
Male
Sex
Partial effect of DOY
Day of the year (DOY)
Partial effect of Age
Age (y)
s(Sound level : low BAI, edf = 1.08)
Sound level [SPLrms(dB re 1 µPa)]
s(Sound level : high BAI, edf = 1.06)
Sound level [SPLrms(dB re 1 µPa)]

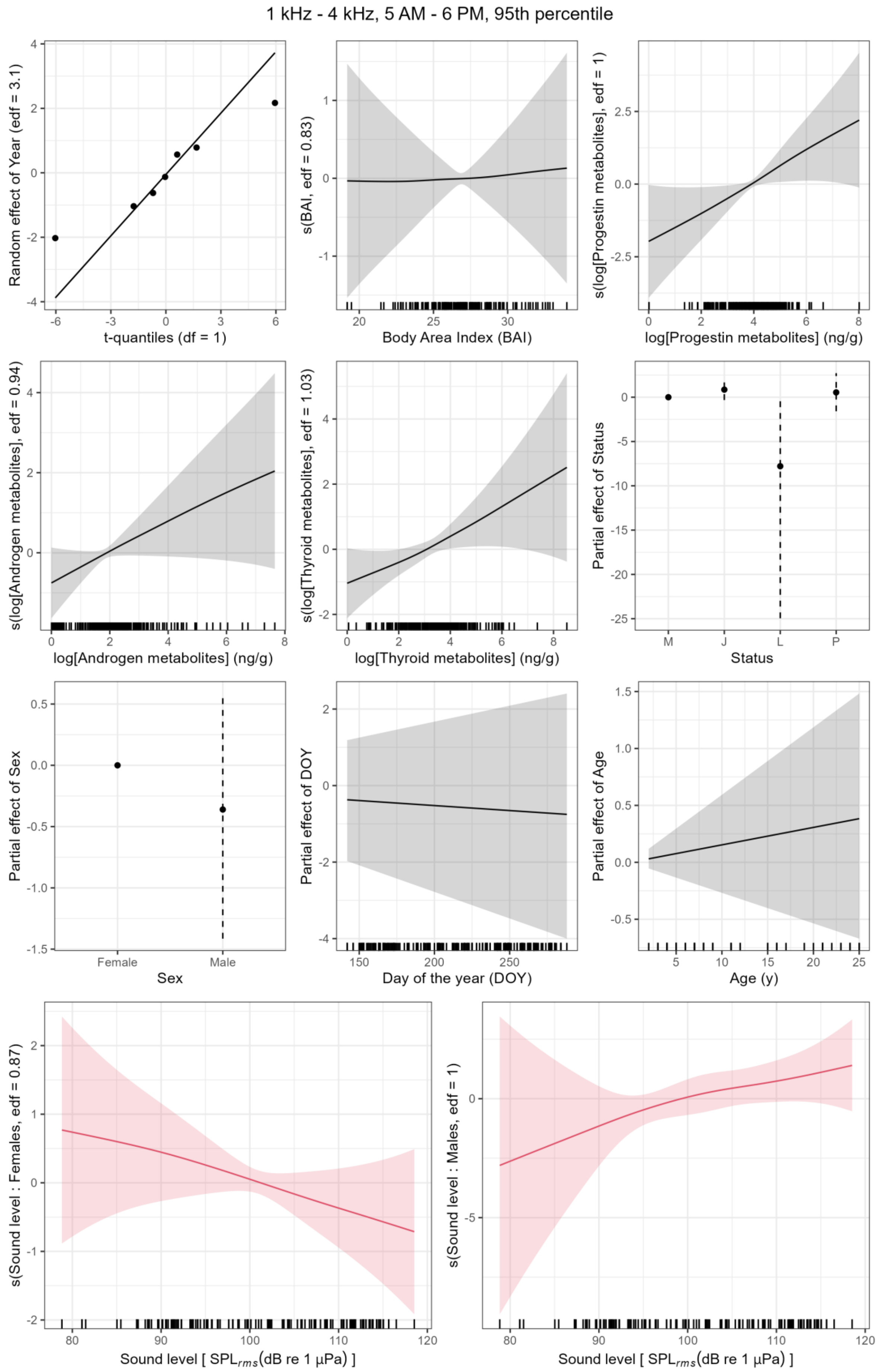
1 kHz - 4 kHz, 5 AM - 6 PM, 95th percentile
Random effect of Year (edf = 3.1)
t-quantiles (df = 1)
s(BAI, edf = 0.83)
Body Area Index (BAI)
s(log[Progestin metabolites], edf = 1)
log[Progestin metabolites] (ng/g)
s(log[Androgen metabolites], edf = 0.94)
log[Androgen metabolites] (ng/g)
s(log[Thyroid metabolites], edf = 1.03)
log[Thyroid metabolites] (ng/g)
Partial effect of Status
Status
M J L P
Partial effect of Sex
Sex
Female Male
Partial effect of DOY
Day of the year (DOY)
Partial effect of Age
Age (y)
s(Sound level : Females, edf = 0.87)
Sound level [SPLrms(dB re 1 μPa)]
s(Sound level : Males, edf = 1)
Sound level [SPLrms(dB re 1 μPa)]

2 days, total

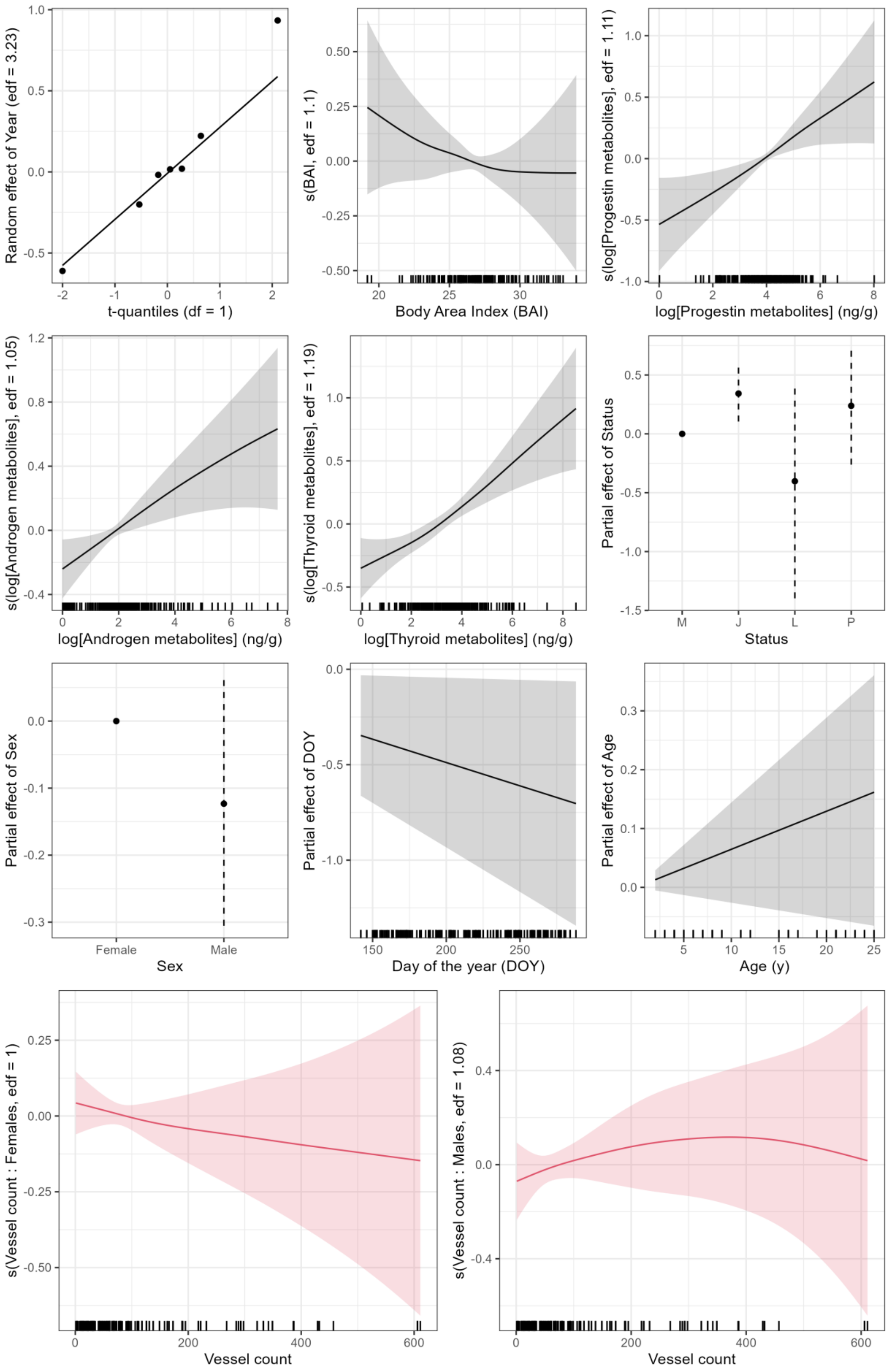
Random effect of Year (edf = 3.23)
t-quantiles (df = 1)
s(BAI, edf = 1.1)
Body Area Index (BAI)
s(log[Progestin metabolites], edf = 1.11)
log[Progestin metabolites] (ng/g)
s(log[Androgen metabolites], edf = 1.05)
log[Androgen metabolites] (ng/g)
s(log[Thyroid metabolites], edf = 1.19)
log[Thyroid metabolites] (ng/g)
Partial effect of Status
Status
M
J
L
P
Partial effect of Sex
Sex
Female
Male
Partial effect of DOY
Day of the year (DOY)
Partial effect of Age
Age (y)
s(Vessel count : Females, edf = 1)
Vessel count
s(Vessel count : Males, edf = 1.08)
Vessel count

2 days, private

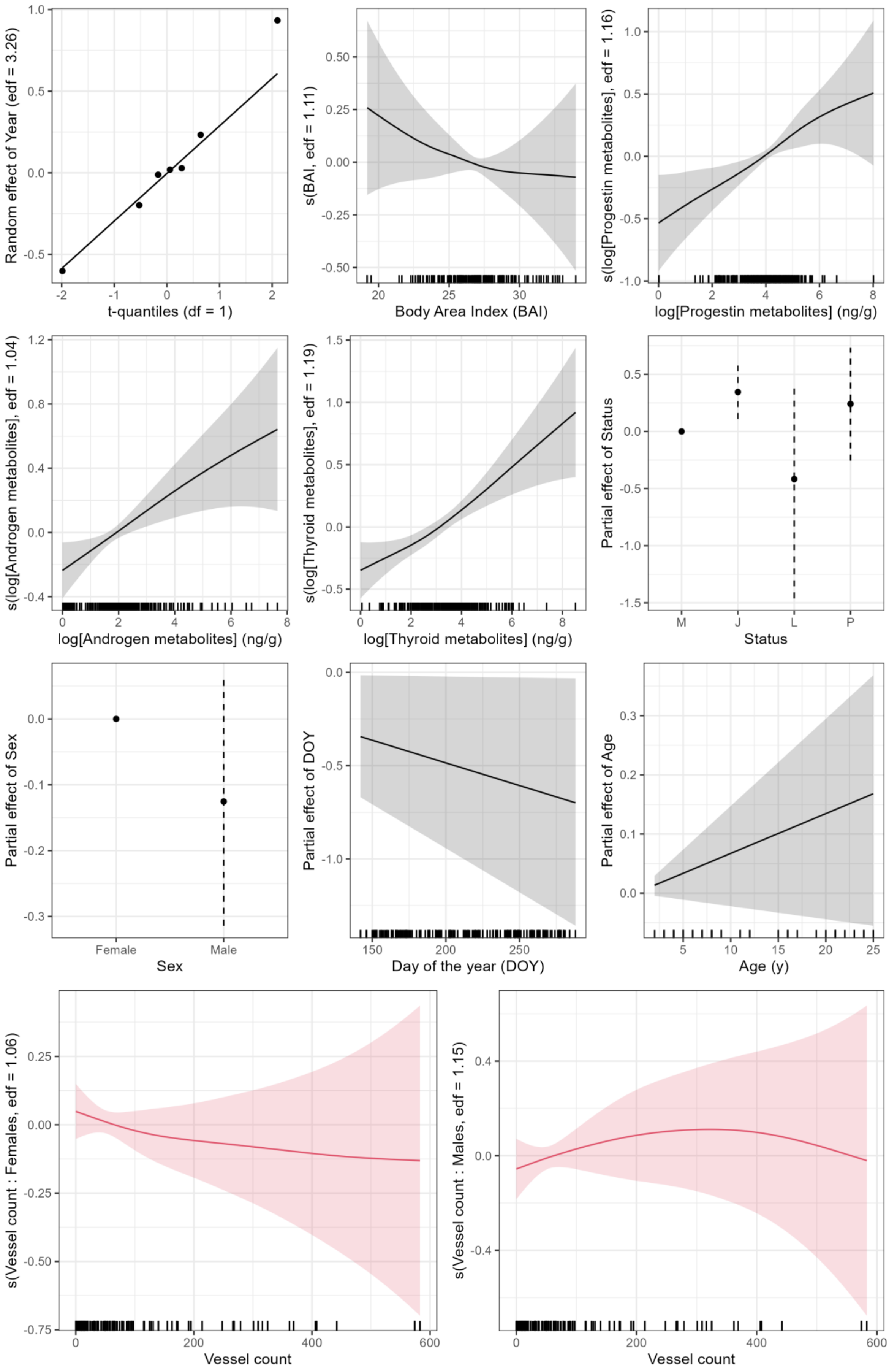

2 days, charters

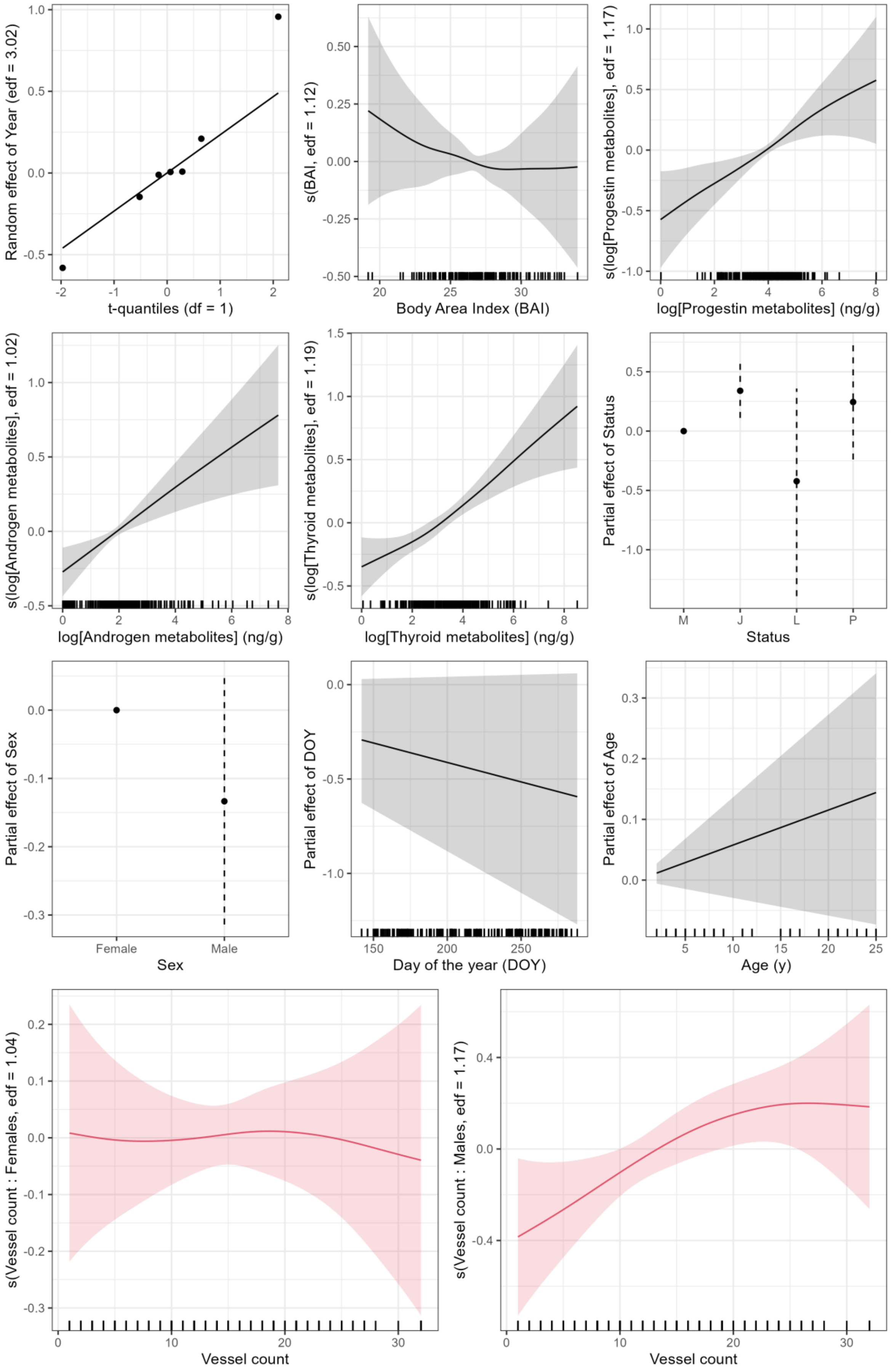

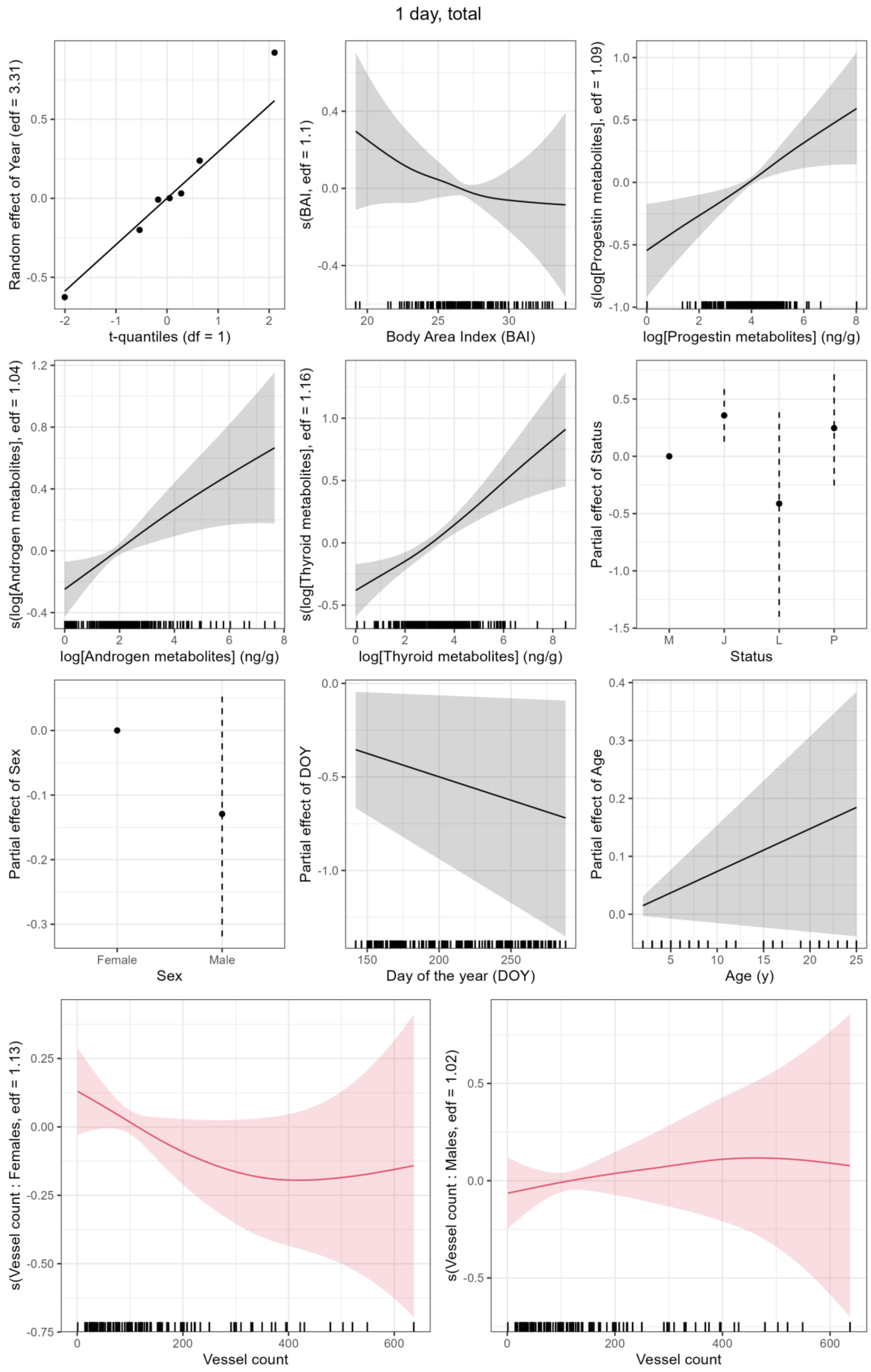
1 day, total
Random effect of Year (edf = 3.31)
t-quantiles (df = 1)
s(BAI, edf = 1.1)
Body Area Index (BAI)
s(log[Progestin metabolites], edf = 1.09)
log[Progestin metabolites] (ng/g)
s(log[Androgen metabolites], edf = 1.04)
log[Androgen metabolites] (ng/g)
s(log[Thyroid metabolites], edf = 1.16)
log[Thyroid metabolites] (ng/g)
Partial effect of Status
Status
M
J
L
P
Partial effect of Sex
Sex
Female
Male
Partial effect of DOY
Day of the year (DOY)
Partial effect of Age
Age (y)
s(Vessel count : Females, edf = 1.13)
Vessel count
s(Vessel count : Males, edf = 1.02)
Vessel count

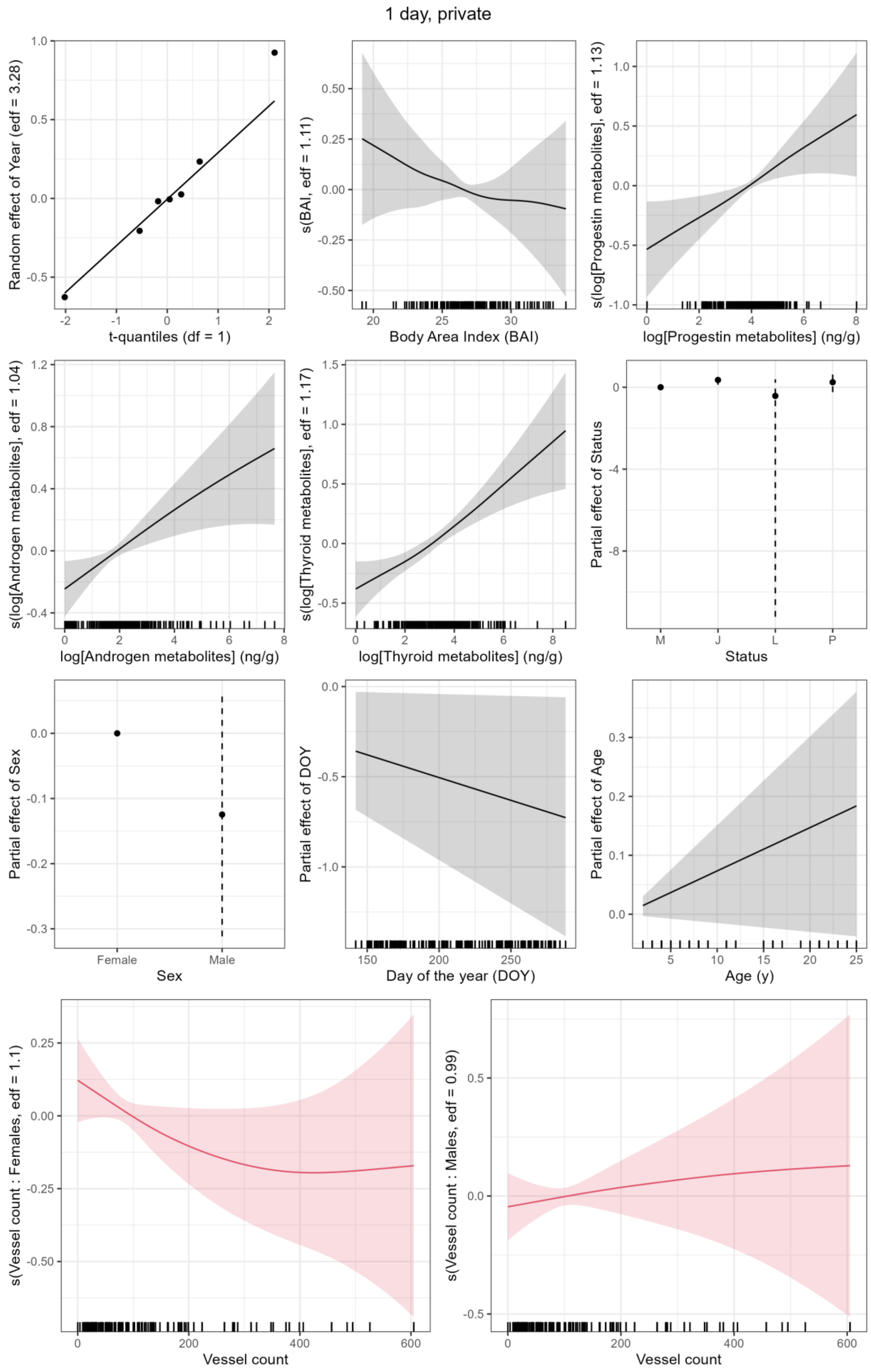
1 day, private
Random effect of Year (edf = 3.28)
t-quantiles (df = 1)
s(BAI, edf = 1.11)
Body Area Index (BAI)
s(log[Progestin metabolites], edf = 1.13)
log[Progestin metabolites] (ng/g)
s(log[Androgen metabolites], edf = 1.04)
log[Androgen metabolites] (ng/g)
s(log[Thyroid metabolites], edf = 1.17)
log[Thyroid metabolites] (ng/g)
Partial effect of Status
M
J
L
P
Status
Partial effect of Sex
Female
Male
Sex
Partial effect of DOY
Day of the year (DOY)
Partial effect of Age
Age (y)
s(Vessel count : Females, edf = 1.1)
Vessel count
s(Vessel count : Males, edf = 0.99)
Vessel count

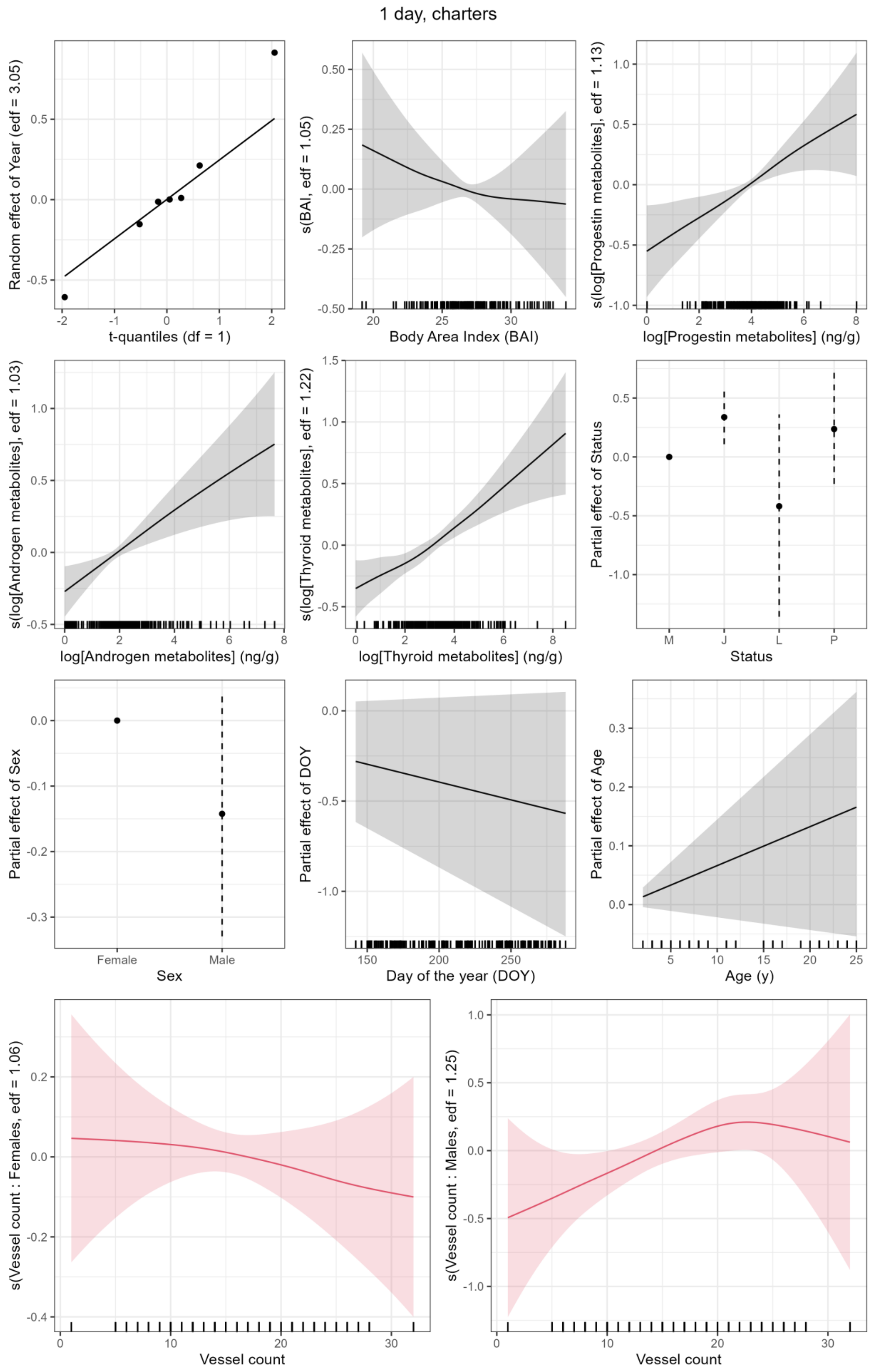
1 day, charters
Random effect of Year (edf = 3.05)
t-quantiles (df = 1)
s(BAI, edf = 1.05)
Body Area Index (BAI)
s(log[Progestin metabolites], edf = 1.13)
log[Progestin metabolites] (ng/g)
s(log[Androgen metabolites], edf = 1.03)
log[Androgen metabolites] (ng/g)
s(log[Thyroid metabolites], edf = 1.22)
log[Thyroid metabolites] (ng/g)
Partial effect of Status
Status
M
J
L
P
Partial effect of Sex
Sex
Female
Male
Partial effect of DOY
Day of the year (DOY)
Partial effect of Age
Age (y)
s(Vessel count : Females, edf = 1.06)
Vessel count
s(Vessel count : Males, edf = 1.25)
Vessel count

2 days, total

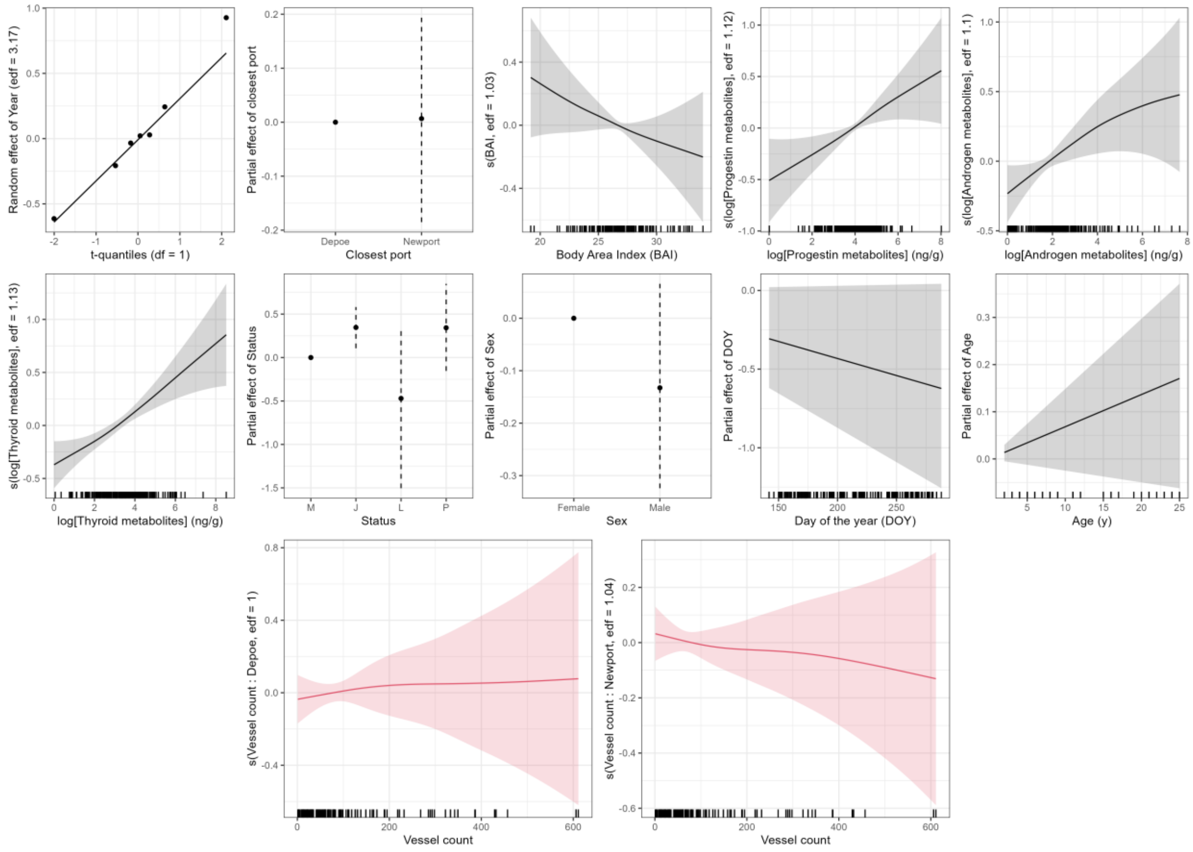

2 days, private

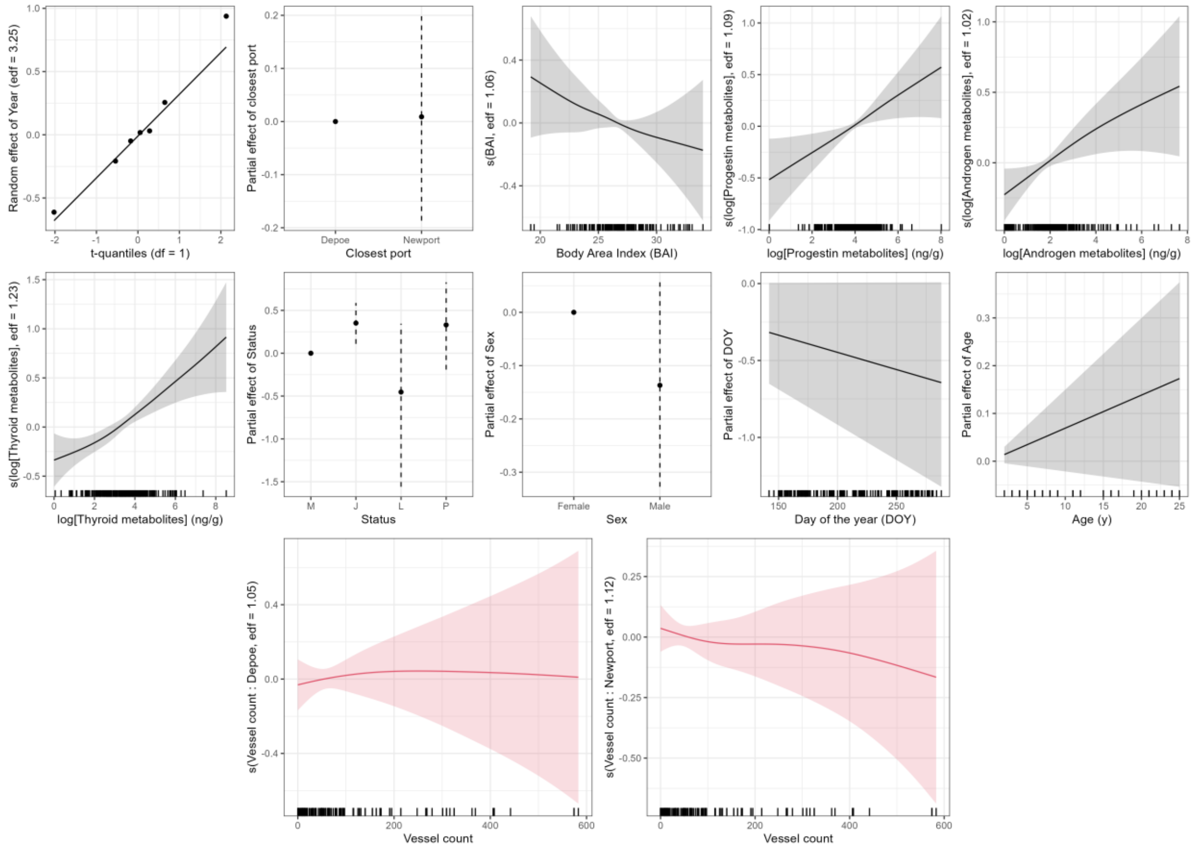

2 days, charters

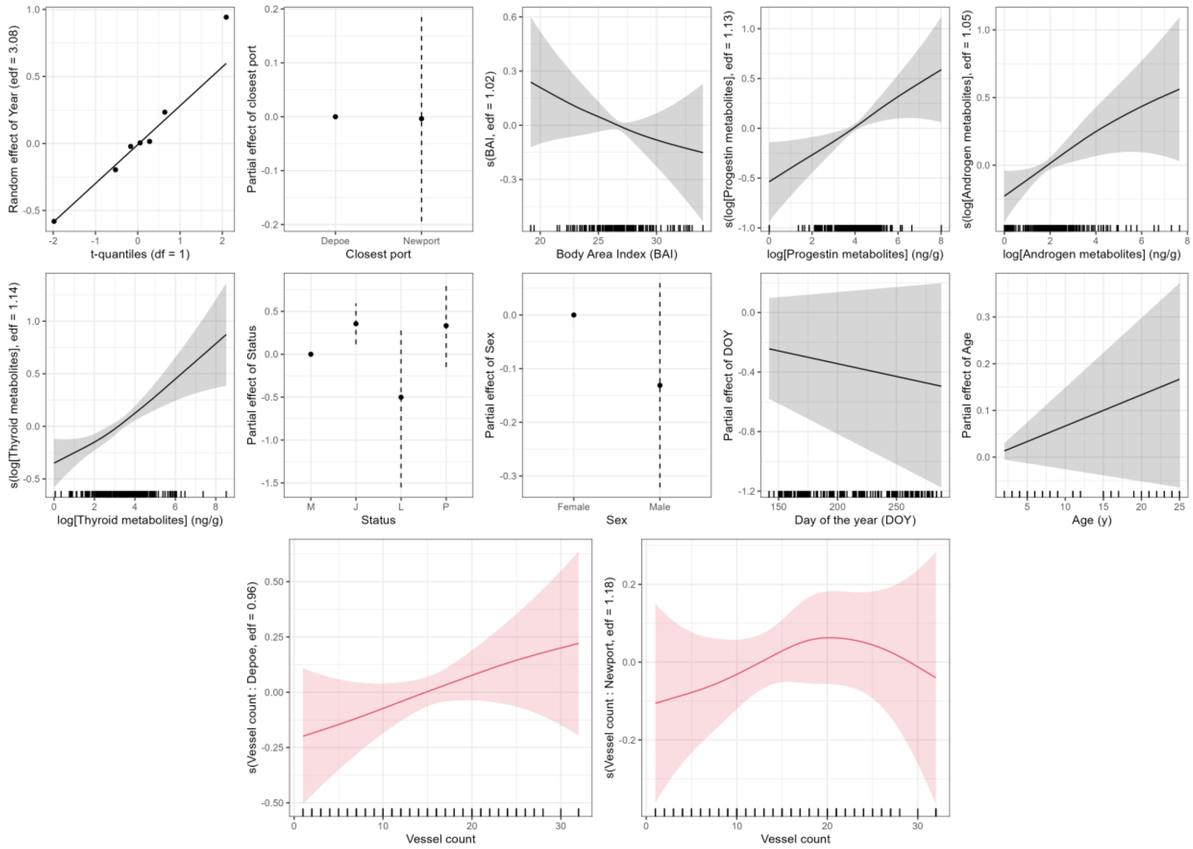

1 day, total

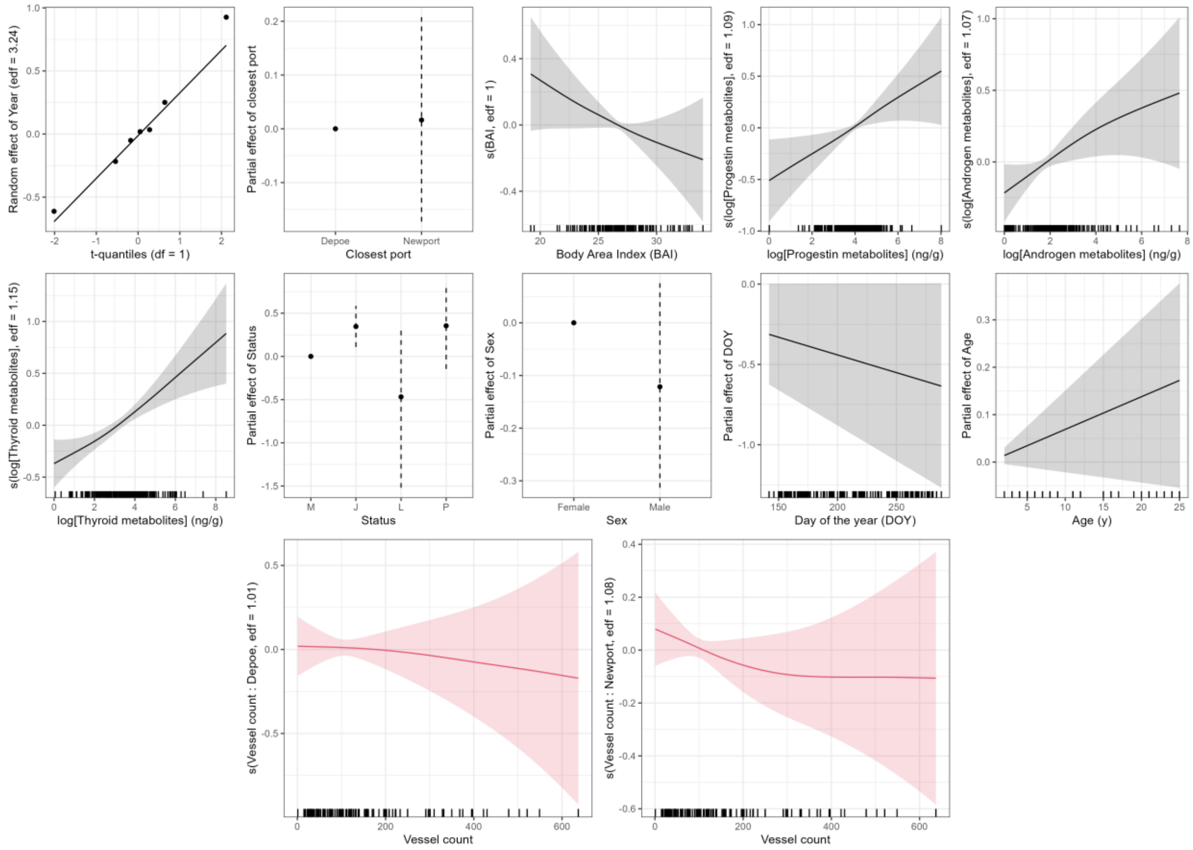

1 day, private

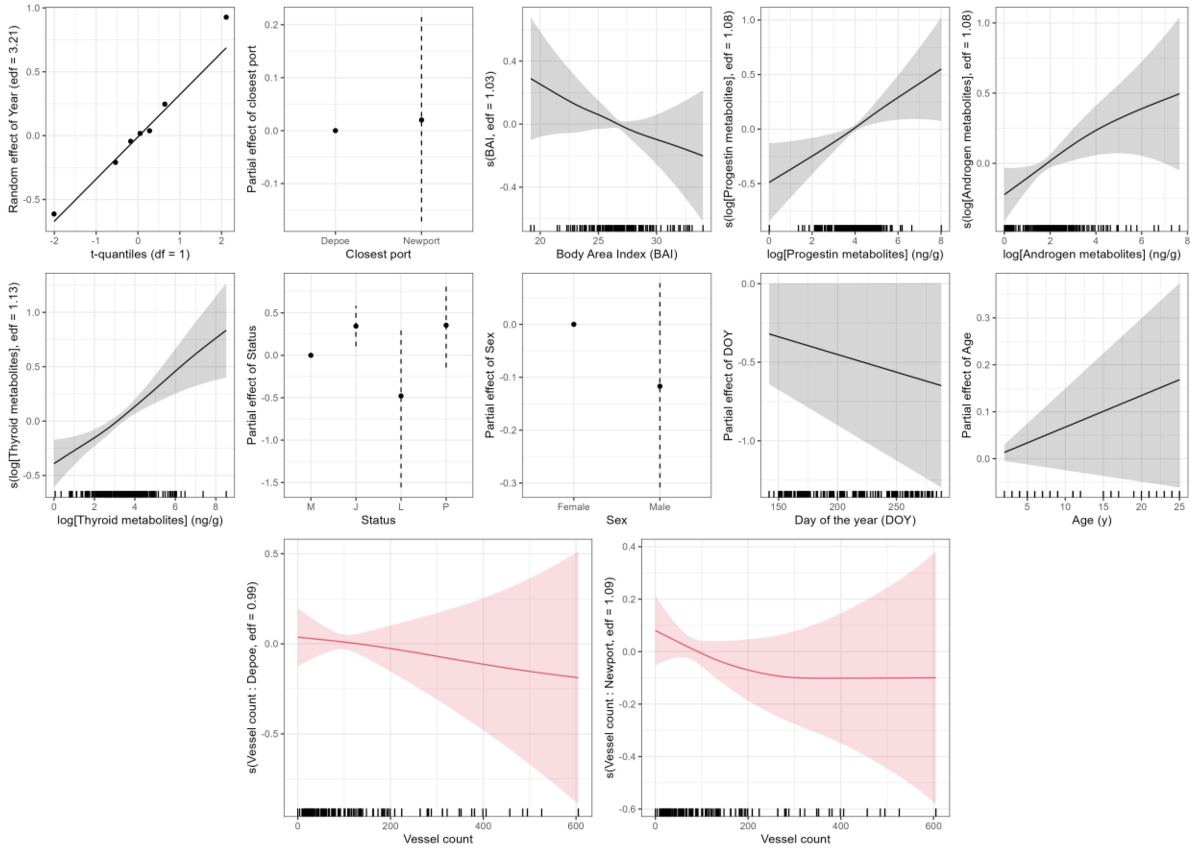

1 day, charters

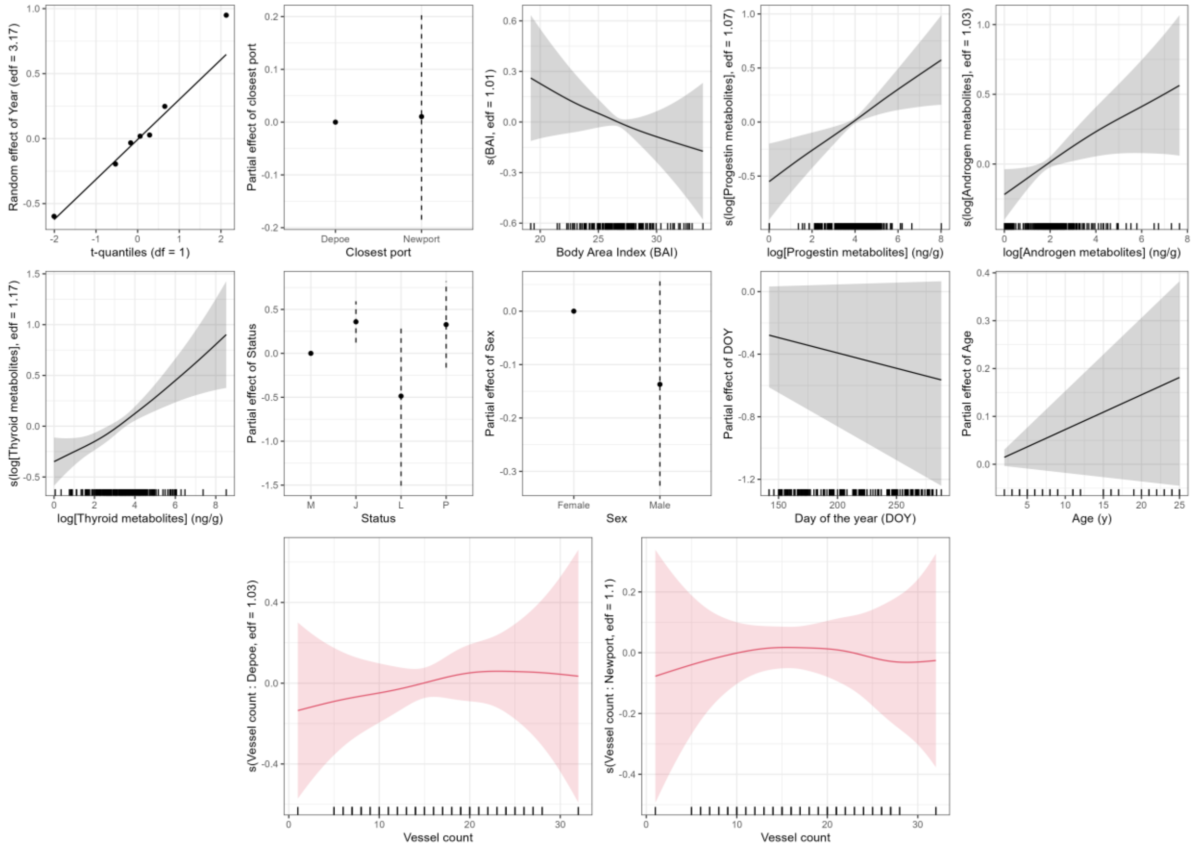

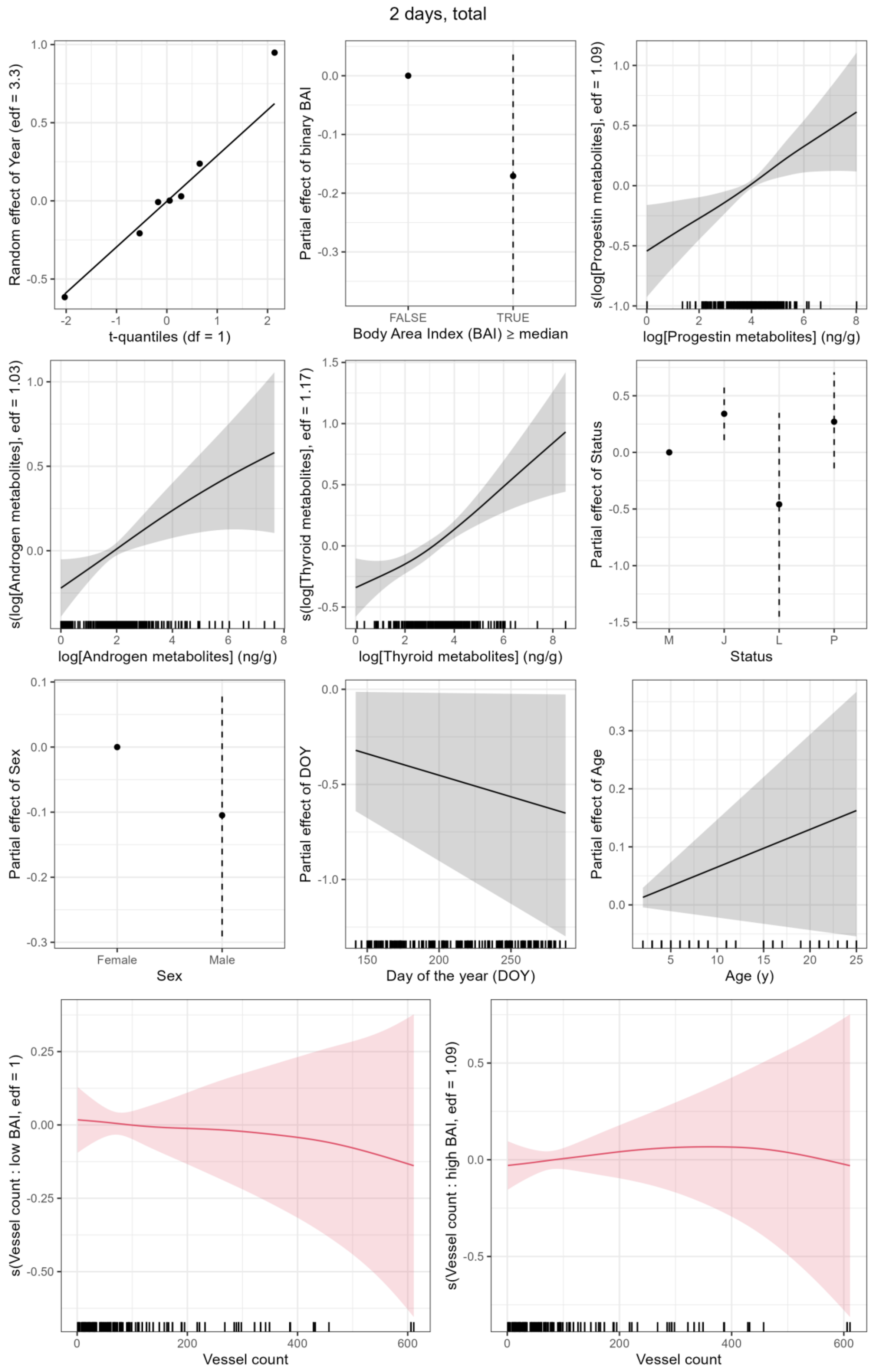

2 days, total
Random effect of Year (edf = 3.3)
t-quantiles (df = 1)
Partial effect of binary BAI
FALSE
TRUE
Body Area Index (BAI) ≥ median
s(log[Progestin metabolites], edf = 1.09)
log[Progestin metabolites] (ng/g)
s(log[Androgen metabolites], edf = 1.03)
log[Androgen metabolites] (ng/g)
s(log[Thyroid metabolites], edf = 1.17)
log[Thyroid metabolites] (ng/g)
Partial effect of Status
M
J
L
P
Status
Partial effect of Sex
Female
Male
Sex
Partial effect of DOY
Day of the year (DOY)
Partial effect of Age
Age (y)
s(Vessel count : low BAI, edf = 1)
Vessel count
s(Vessel count : high BAI, edf = 1.09)
Vessel count

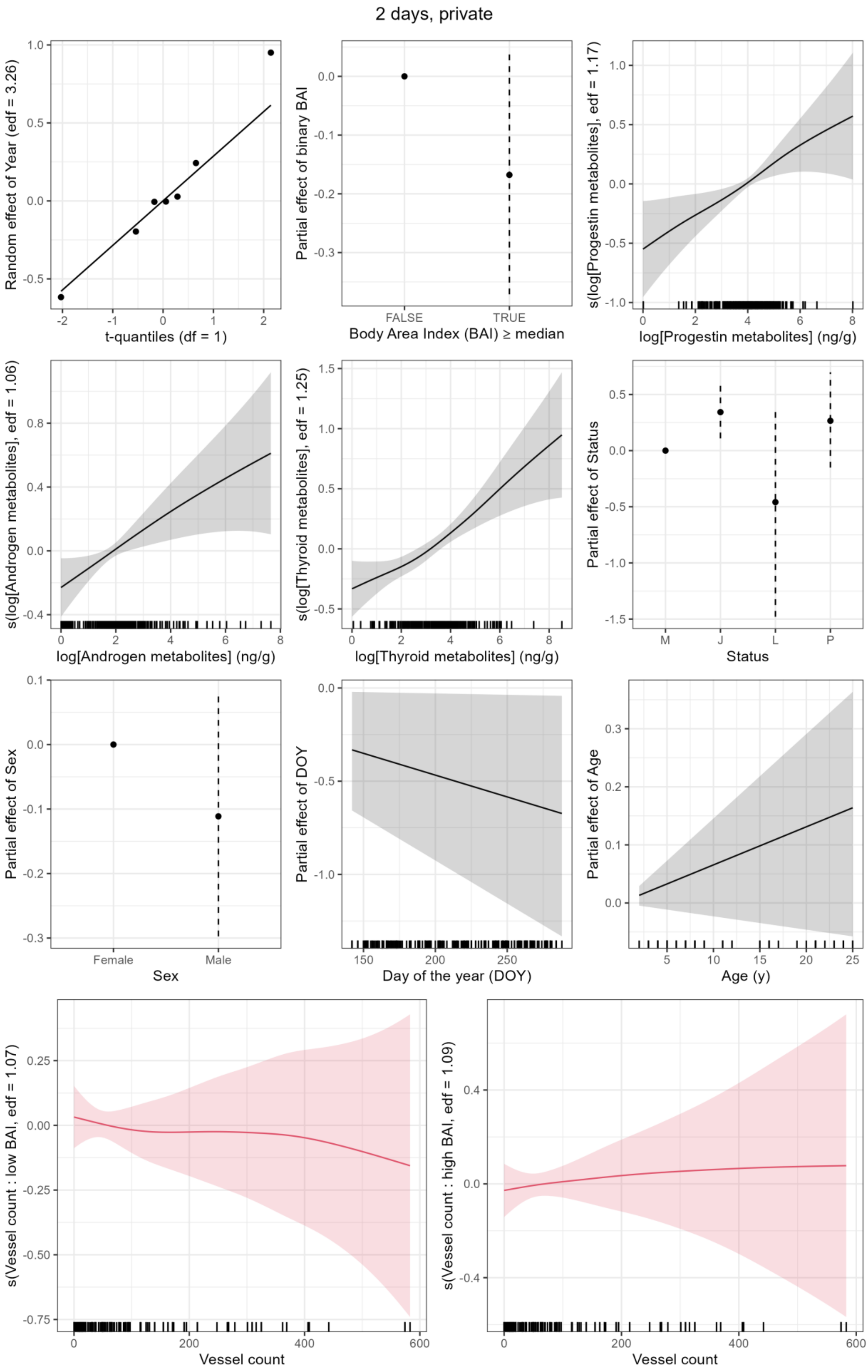
2 days, private
Random effect of Year (edf = 3.26)
t-quantiles (df = 1)
Partial effect of binary BAI
FALSE
TRUE
Body Area Index (BAI) ≥ median
s(log[Progestin metabolites], edf = 1.17)
log[Progestin metabolites] (ng/g)
s(log[Androgen metabolites], edf = 1.06)
log[Androgen metabolites] (ng/g)
s(log[Thyroid metabolites], edf = 1.25)
log[Thyroid metabolites] (ng/g)
Partial effect of Status
M
J
L
P
Status
Partial effect of Sex
Female
Male
Sex
Partial effect of DOY
Day of the year (DOY)
Partial effect of Age
Age (y)
s(Vessel count : low BAI, edf = 1.07)
Vessel count
s(Vessel count : high BAI, edf = 1.09)
Vessel count

2 days, charters

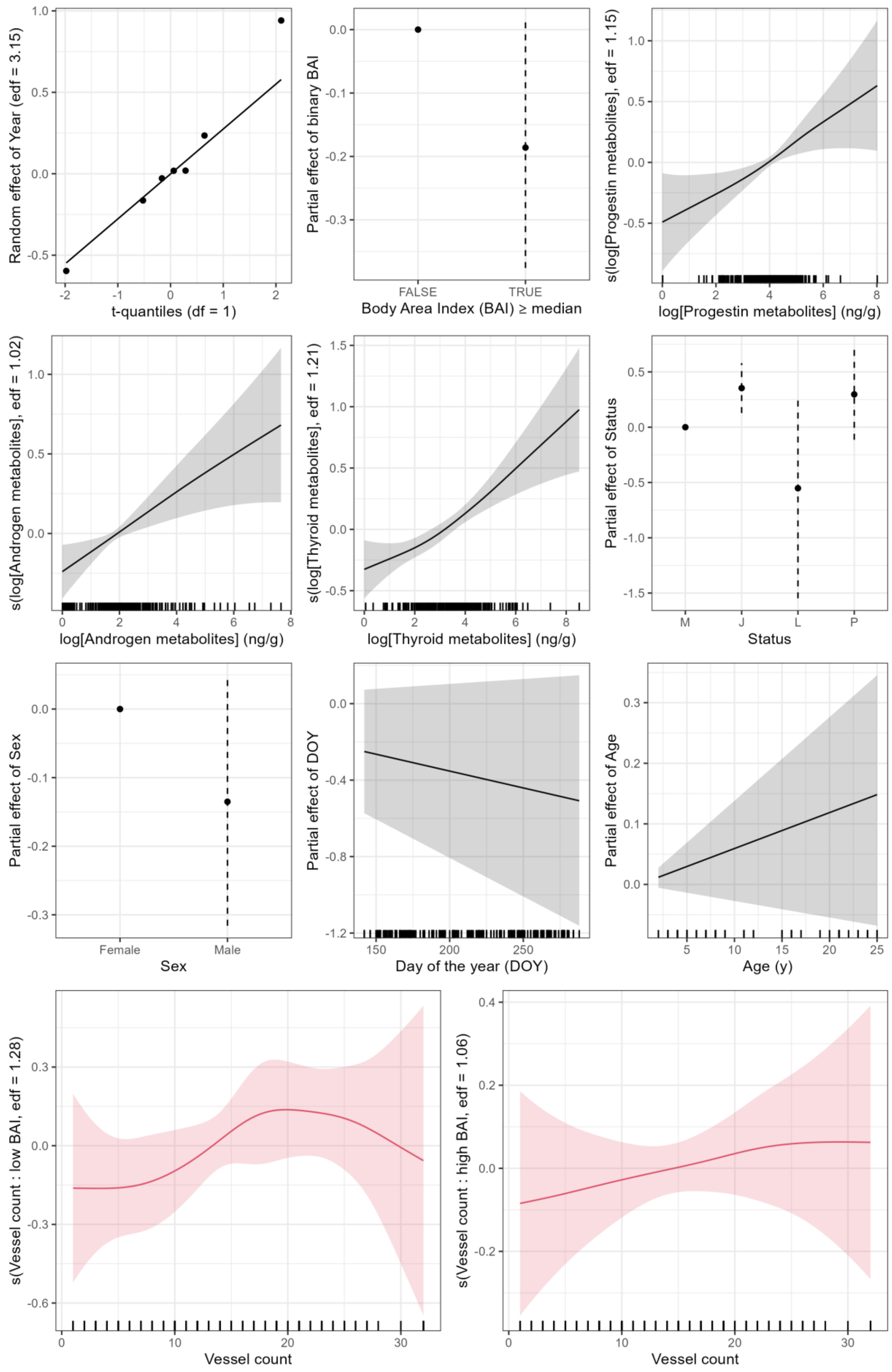

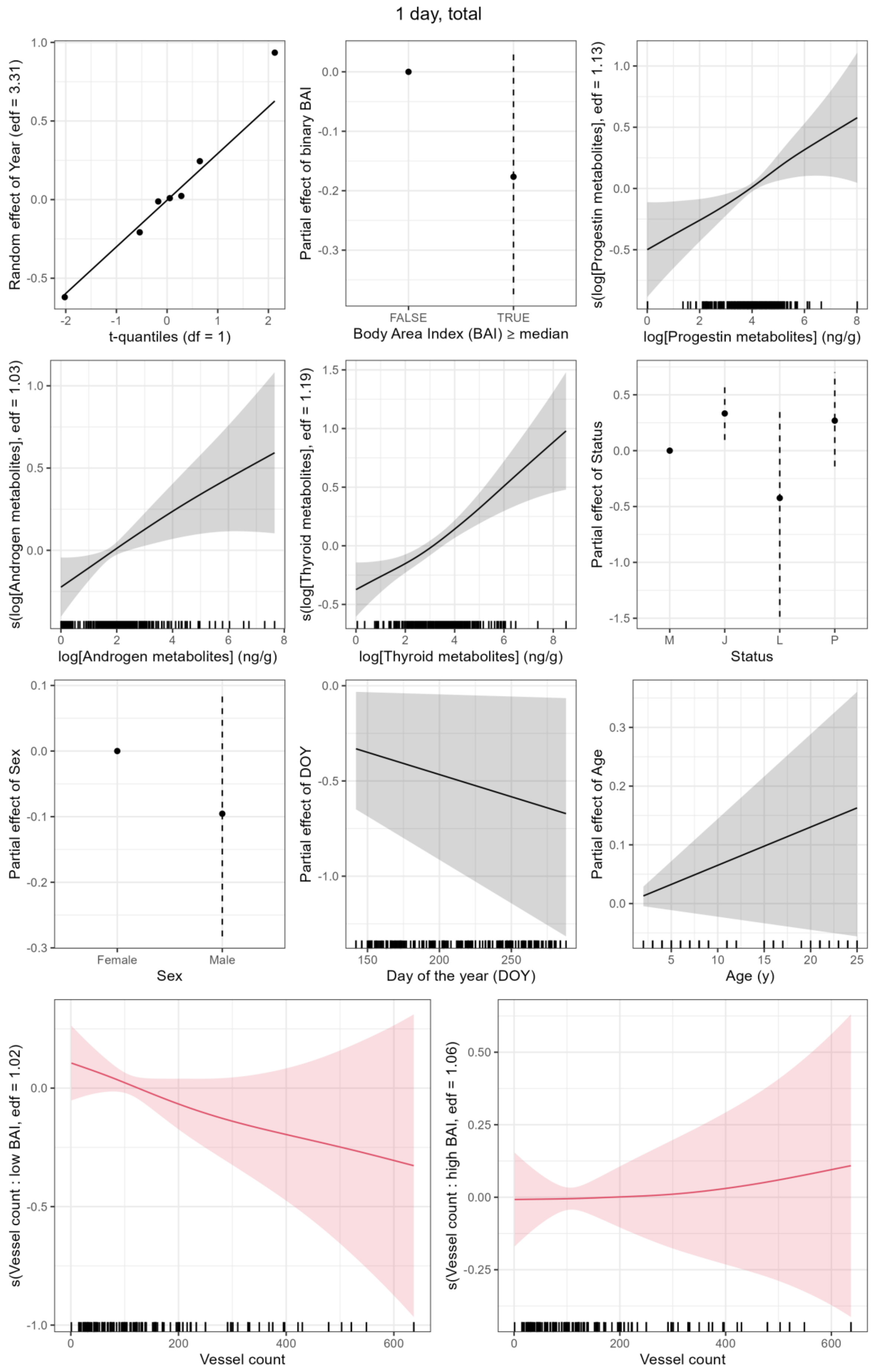
1 day, total
Random effect of Year (edf = 3.31)
t-quantiles (df = 1)
Partial effect of binary BAI
FALSE
TRUE
Body Area Index (BAI) ≥ median
s(log[Progestin metabolites], edf = 1.13)
log[Progestin metabolites] (ng/g)
s(log[Androgen metabolites], edf = 1.03)
log[Androgen metabolites] (ng/g)
s(log[Thyroid metabolites], edf = 1.19)
log[Thyroid metabolites] (ng/g)
Partial effect of Status
M
J
L
P
Status
Partial effect of Sex
Female
Male
Sex
Partial effect of DOY
Day of the year (DOY)
Partial effect of Age
Age (y)
s(Vessel count : low BAI, edf = 1.02)
Vessel count
s(Vessel count : high BAI, edf = 1.06)
Vessel count

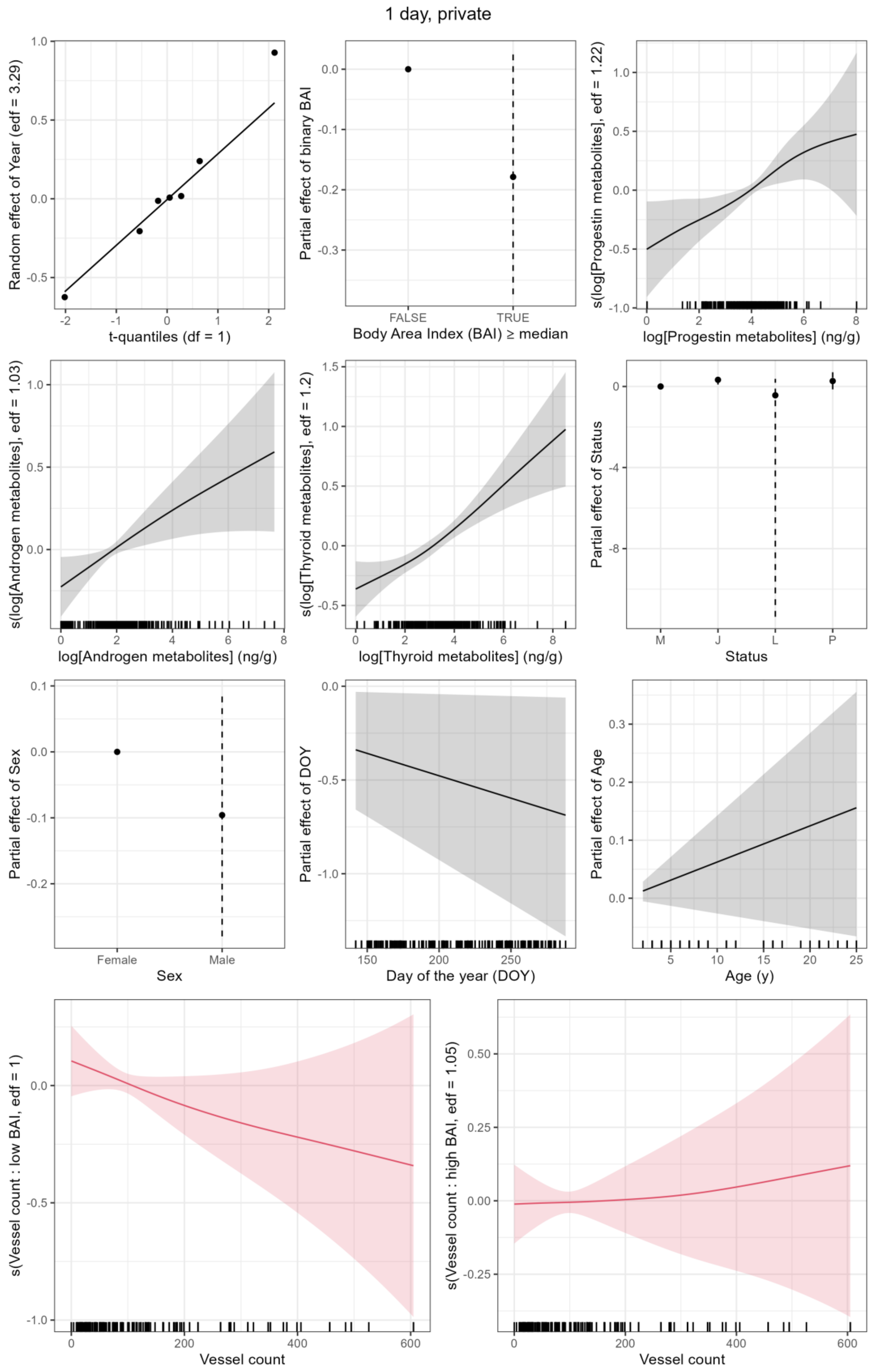
1 day, private
Random effect of Year (edf = 3.29)
t-quantiles (df = 1)
Partial effect of binary BAI
FALSE
TRUE
Body Area Index (BAI) ≥ median
s(log[Progestin metabolites], edf = 1.22)
log[Progestin metabolites] (ng/g)
s(log[Androgen metabolites], edf = 1.03)
log[Androgen metabolites] (ng/g)
s(log[Thyroid metabolites], edf = 1.2)
log[Thyroid metabolites] (ng/g)
Partial effect of Status
M
J
L
P
Status
Partial effect of Sex
Female
Male
Sex
Partial effect of DOY
Day of the year (DOY)
Partial effect of Age
Age (y)
s(Vessel count : low BAI, edf = 1)
Vessel count
s(Vessel count : high BAI, edf = 1.05)
Vessel count

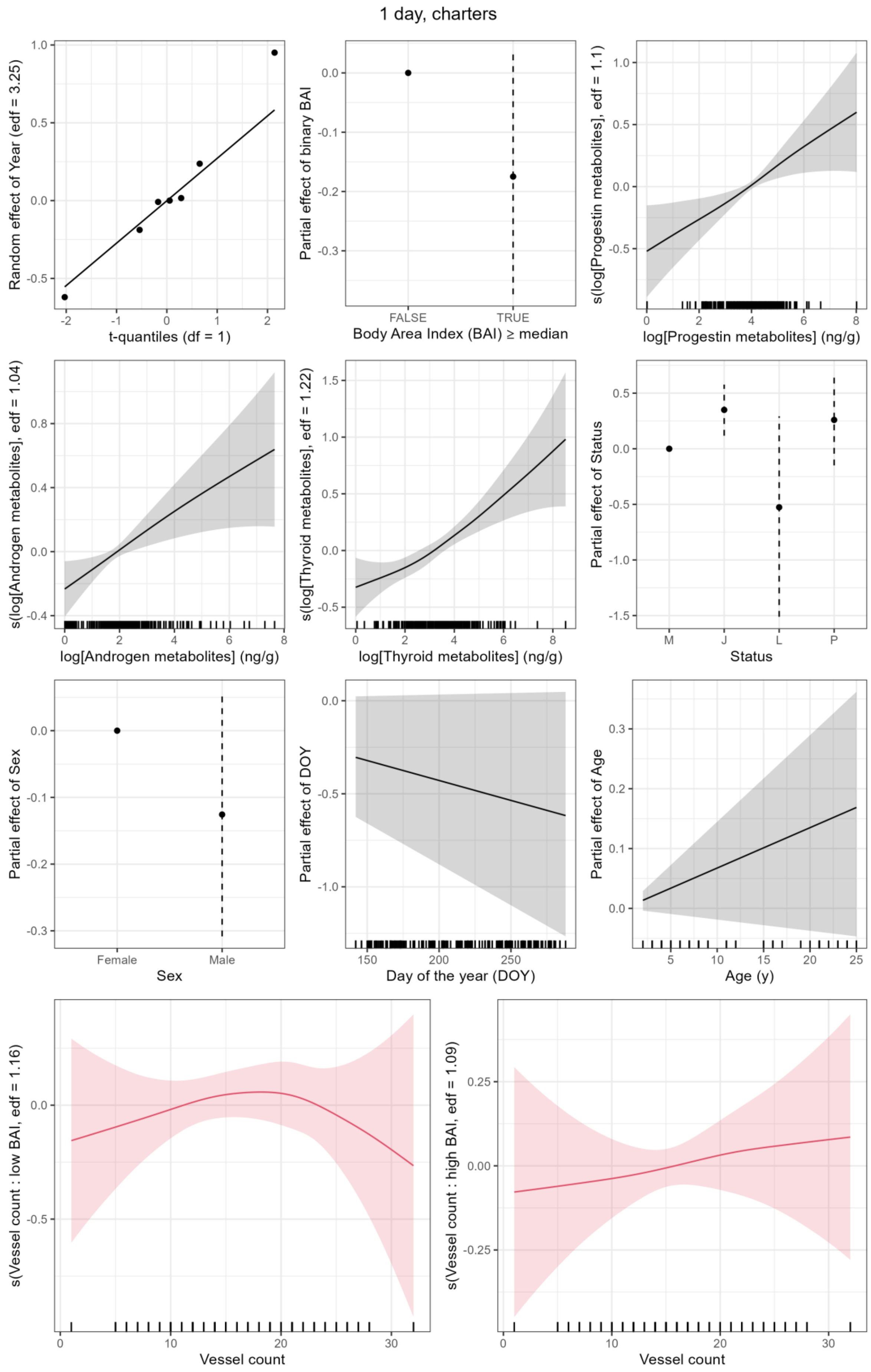
1 day, charters
Random effect of Year (edf = 3.25)
t-quantiles (df = 1)
Partial effect of binary BAI
FALSE
TRUE
Body Area Index (BAI) ≥ median
s(log[Progestin metabolites], edf = 1.1)
log[Progestin metabolites] (ng/g)
s(log[Androgen metabolites], edf = 1.04)
log[Androgen metabolites] (ng/g)
s(log[Thyroid metabolites], edf = 1.22)
log[Thyroid metabolites] (ng/g)
Partial effect of Status
M
J
L
P
Status
Partial effect of Sex
Female
Male
Sex
Partial effect of DOY
Day of the year (DOY)
Partial effect of Age
Age (y)
s(Vessel count : low BAI, edf = 1.16)
Vessel count
s(Vessel count : high BAI, edf = 1.09)
Vessel count

Example of observational dose-response function

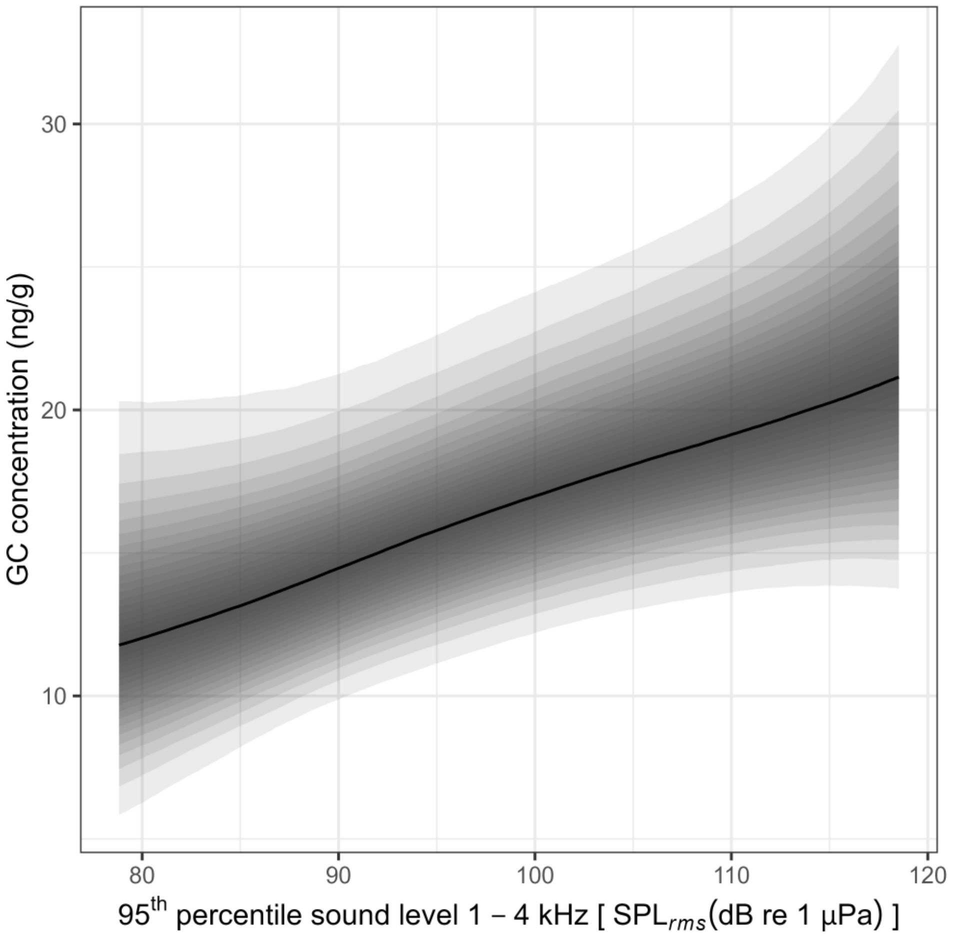

Figure S24. Example of dose-response function derived from the results of the model including the interaction between sex and the 95th percentile of sound levels in the high frequency band (1 – 4 kHz) between 5 AM and 6 PM in the day prior to sampling. This function represents the relationship between sound level and the median faecal GC concentration in mature males, with all other contextual variables in the model set to their mean value. The shaded area indicates the 95% credible interval. This relationship should be considered observational, i.e., it was not derived using a formal experimental design measuring individuals' responses before and after the exposure to the stressor.

References

Bierlich KC, Schick RS, Hewitt J, Dale J, Goldbogen JA, Friedlaender AS, Johnston DW (2021) Bayesian approach for predicting photogrammetric uncertainty in morphometric measurements derived from drones. *Mar Ecol Prog Ser* 673: 193–210.

Dawson SM, Bowman MH, Leunissen E, Sirguey P (2017) Inexpensive aerial photogrammetry for studies of whales and large marine animals. *Front Mar Sci* 4: 366.

Haver S, Haxel J, Dziak R, Roche L, Matsumoto H, Hvidsten C, Torres L (2023) The variable influence of anthropogenic noise on summer season coastal underwater soundscapes near a port and marine reserve. *Mar Pollut Bull* 194: 115406.

Lemos LS, Haxel JH, Olsen A, Burnett JD, Smith A, Chandler TE, Nieukirk SL, Larson SE, Hunt KE, Torres LG (2022) Effects of vessel traffic and ocean noise on gray whale stress hormones. *Sci Rep* 12: 18580.

Rice D, Wolman A (1971) The Life History and Ecology of the Gray Whale: *Eschrichtius Robustus*. American Society of Mammalogists. Special Publication No. 3. Stillwater, Oklahoma.
